# Economic analyses of supported employment programmes for people with mental health conditions: A systematic review

**DOI:** 10.1192/j.eurpsy.2022.2309

**Published:** 2022-08-19

**Authors:** A-La Park, Miles Rinaldi, Beate Brinchmann, Eoin Killackey, Nils Abel P. Aars, Arnstein Mykletun, David McDaid

**Affiliations:** 1 Nordland Hospital Trust, Centre for Work and Mental Health, Bodø, Norway; 2 Department of Community Medicine, UiT – The Arctic University of Norway, Tromsø, Norway; 3 Care Policy and Evaluation Centre, Department of Health Policy, London School of Economics and Political Science, London, United Kingdom; 4 South West London & St George’s Mental Health NHS Trust, London, United Kingdom; 5 Orygen, The National Centre of Excellence in Youth Mental Health, Parkville, Victoria, Australia; 6 Centre for Youth Mental Health, The University of Melbourne, Melbourne, Victoria, Australia; 7 Centre for Research and Education in Forensic Psychiatry and Psychology, Haukeland University Hospital, Bergen, Norway; 8 Division of Mental Health, Norwegian Institute of Public Health, Oslo, Norway

**Keywords:** Cost-effectiveness, economic evaluation, individual placement and support, supported employment

## Abstract

**Background:**

Employment is intrinsic to recovery from mental health conditions, helping people live independently. Systematic reviews indicate supported employment (SE) focused on competitive employment, including individual placement and support (IPS), is effective in helping people with mental health conditions into work. Evidence is limited on cost-effectiveness. We comprehensively reviewed evidence on the economic case for SE/IPS programmes.

**Methods:**

We searched PubMed/MEDLINE, EMBASE, PsycINFO, CINAHL, IBSS, Business Source Complete, and EconLit for economic and return on investment analyses of SE/IPS programmes for mental health conditions. Traditional vocational rehabilitation, sheltered work, and return to work initiatives after sickness absence of less than 1 year were excluded. Studies were independently screened by two reviewers. We assessed quality using the Consolidate Health Economic Evaluation Reporting Standards checklist. The protocol was preregistered with PROSPERO-CRD42020184359.

**Results:**

From 40,015 references, 28 studies examined the economic case for IPS, four IPS augmented by another intervention, and 24 other forms of SE. Studies were very heterogenous, quality was variable. Of 41 studies with quality scores over 50%, 10 reported cost per quality-adjusted life year gained, (8 favourable to SE/IPS), 14 net monetary benefits (12 positive), 5 return on investment (4 positive), and 20 cost per employment outcome (14 favorable, 5 inconclusive, 1 negative). Totally, 24 of these 41 studies had monetary benefits that more than outweighed the additional costs of SE/IPS programmes.

**Conclusions:**

There is a strong economic case for the implementation of SE/IPS programmes. The economic case is conservative as evidence on long-term impacts of programmes is limited.

## Introduction

Good employment is intrinsic to recovery from mental health conditions, improving quality of life, and empowering people to live independently [1]. Lost employment represents the majority of costs of mental health conditions to society [2]. Supported employment (SE) programmes help people to enter or return to employment. Programmes vary but are characterised by a “place and train” approach with a focus on competitive employment as the goal with ongoing support to retain employment [3]. The individual placement and support (IPS) approach is one form of SE, underpinned by eight evidence-based principles, including employment specialists integrated into mental health services, playing an intermediate role to match jobseekers preferences with employers in a competitive job market, and initiating rapid search for competitive employment [4, 5]. Systematic reviews indicate SE interventions, particularly IPS, are highly effective in helping people with mental health conditions into work [6, 7]. Despite this, policy decisions and implementation of evidenced-based nonpharmacological structured interventions can be slow (at best). This is the case for SE/IPS.

The economic case for SE/IPS is potentially strong as it has merit to reduce long-term disability related to illness and may catalyse policy decisions and implementation. Reviews have focused on the effectiveness of SE/IPS, rather than the economic case; only one systematic review of economic evaluations was identified and restricted to trial-based evaluations of IPS for people with severe mental illness (SMI) [8]. It identified seven studies published between 1998 and 2017, generally favouring IPS. Another review of supported employment for people with disabilities reported 6 economic evaluations, again all focused on IPS, the most recent of which was published in 2014 [9]. A narrative review looking at the social costs of expanding access to SE/IPS identified 27 studies on the costs of interventions, but only included 5 economic evaluations [10]. Consequently, this review aims to comprehensively review evidence on economic evaluations of SE/IPS programmes.

## Methods

A systematic review was registered with PROSPERO (CRD42020184359) to identify economic evaluations of SE/IPS programmes using a “place and train” approach to support working-age individuals to find and stay in employment in the competitive labour market. Traditional vocational rehabilitation and sheltered work programmes, as well as education, training, and initiatives to promote return to work after sick leave of less than 1 year were excluded.

Although the PROSPERO review covers people with all health conditions/disabilities, this article focuses solely on interventions for people with any mental health condition, regardless of severity, including learning disabilities but excluding dementia. We searched PubMed/MEDLINE, EMBASE, PsycINFO, CINAHL, Business Source Complete, IBSS, and EconLit. Because of the volume of records found, and examination of previous reviews which identified only one economic evaluation between 2000 and 2009 [11], one deviation from the protocol was to search for literature published between January 2009, rather than January 2000 to August 2021 (see search strategies in Supplementary S1). The multicountry EQOLISE study on supported employment had just been published in 2008 [12]; this had an economic evaluation fully embedded that we felt would act as a catalyst for later studies. Another deviation was that we were also unable to search two social science databases, PAIS International and ASSIA as their subscriptions lapsed. Reference lists of relevant papers were checked and Google Scholar searched. There were no language restrictions. Title/abstracts and full texts of papers were independently screened by two reviewers. Disagreements were resolved through discussion and, if necessary, input from a third reviewer.

The economic outcomes in identified studies were likely to be very heterogenous and in the protocol, we anticipated finding studies with many different outcomes, including cost-effectiveness studies that report incremental costs per additional unit of outcome (employment) achieved between two or more interventions, such as cost per job, or day of work. In this case, the amount policymakers are willing to pay for better outcomes is a value judgment varying across countries.

However, these cost-effectiveness studies are of limited value as their relative cost-effectiveness cannot be compared easily with other health-related interventions, e.g. investment in support for carers of people living with dementia. To overcome this, health economists often measure cost per quality-adjusted life year (QALY) gained, where a year spent in perfect quality of life represents 1 QALY. Quality of Life can be elicited in different ways, for instance asking study respondents to complete the EQ-5D [13], an instrument often used in health economic studies. A monetary value can also be placed on a QALY, but this varies across countries, reflecting differences in societal willingness to pay for a QALY. Other anticipated measures included cost-benefit analyses where both costs and outcomes are valued monetarily to generate net monetary benefits (NMBs), and return on investment (ROI) analyses where the cost of investing in SE/IPS is compared to the monetary value of additional employment and costs averted.

Extracted information included country, population, intervention, study design, economic evaluation type, timeframe, perspectives, key impacts, costs related to mental health and work, and summary economic findings. We used the Consolidate Health Economic Evaluation Reporting Standards (CHEERS) checklist to judge the quality of economic evaluation methodologies [14]. Recent new CICERO appraisal guidance on economic reviews highlights this as one recommended tool [15]. We added one additional item to the existing checklist on whether labour market outcomes, as well as health outcomes, were reported. The checklist has 23 or 25 items depending on whether it was a single study or model-based economic evaluation; we allocated one point per item with scores as a percentage indicating strength of evidence. All costs in the text have been converted to purchasing power parity adjusted 2020 US Dollars, using the CCEMG – EPPI-Centre Cost Converter [16]. We report original currency values in tables.

## Results

**Figure 1.**
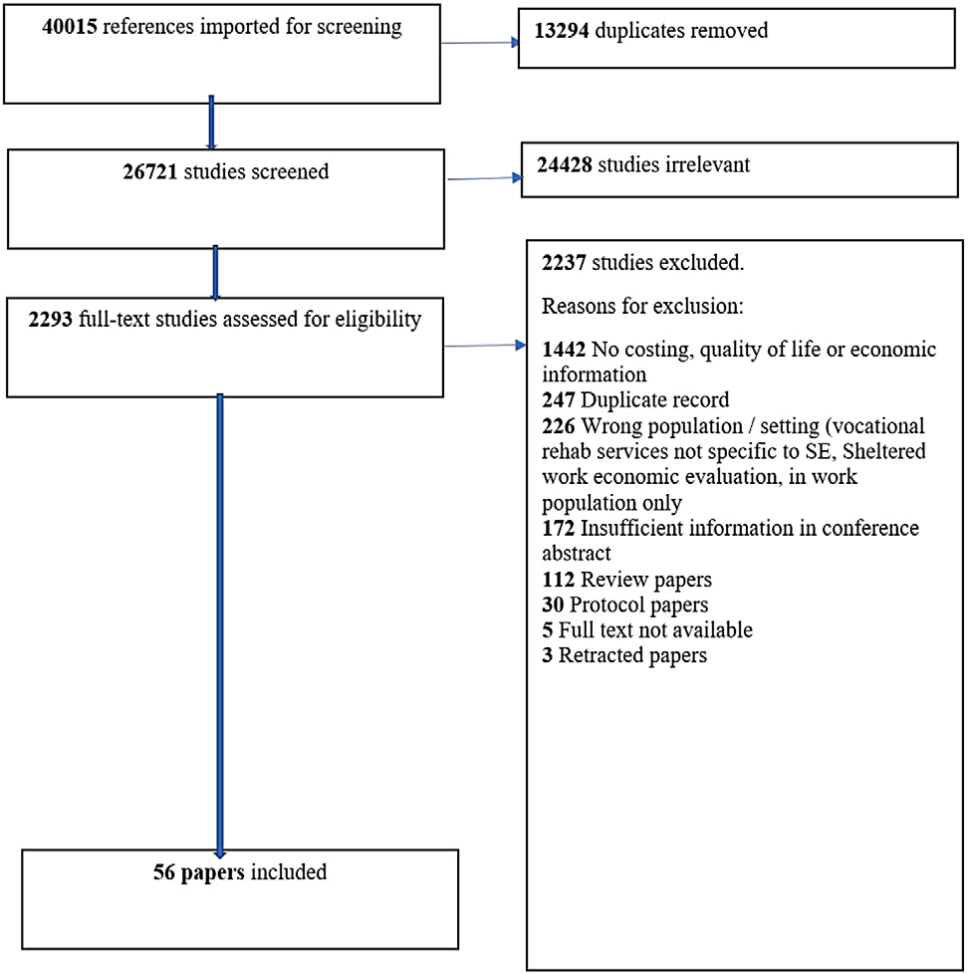
PRISMA flow diagram.

Figure 1 shows a PRISMA (Preferred Reporting Items for Systematic Reviews and Meta-Analyses) diagram with review results. From 40,015 references, 56 papers covering 54 economic studies were identified (see Supplementary S2 for a list of all studies). A total of 43 studies (79%) reported a positive economic case for SE/IPS with three (5%) being negative and eight (15%) inconclusive. Studies are very heterogenous; 13 used multiple evaluation methods. A total of 23 included a cost–benefit analysis, 11 cost per QALY gained, 5 ROI and 25 cost per employment outcome achieved, measured as job gained, hour, day, or week worked. Five were cost-consequence studies where multiple outcomes and costs are recorded but no synthesis is conducted. Overall, 28 studies examined the economic case for IPS, four IPS augmented by another intervention, and 24 other forms of SE. Studies ranged from under 10 to 173,000 participants, with time horizons from 6 weeks to 50 years. Other than one economic evaluation of a six-country trial in Europe [17], all were single-country studies. In total, 17 (31%) were set in the UK, 18 (33%) in the USA, and 8 (15%) in the Nordic countries.

Quality was variable; the mean quality score was 63% (range 30–96%) (See quality checks in Supplementary Table S1). We focus here on 41 studies with quality scores over 50%, including 24 with scores over 70% (Table 1). Ten studies in Table 1 reported cost per QALY gained, (8 favourable to SE/IPS), 16 NMBs (14 positive), 5 ROI (4 positive), and 20 cost per employment outcome (14 favourable, 5 inconclusive, 1 negative). Sixteen of the 24 higher-quality evaluations were linked to empirical studies, mainly randomised controlled trials (RCTs). The remainder synthesised information from multiple sources on costs and benefits of SE/IPS.

**Table 1.** Summary results for studies with quality scores of 50% and above.

Study	Country	Study design/Economic evaluation	Outcome	Perspective	Main economic finding	Net intervention cost and funder of SE	Which sectors benefit?^a^	Time horizon	Quality score
IPS studies									
Christensen et al. [18]	Denmark	RCT/CEA, CUA	QALYs (EQ-5D), Employment	Societal	96% probability of being cost-effective at €35,000 WTP	€914, NS	All sectors: €10,457 LGovt (LM): 29%	1.5 years	95%
Deloitte [19]	UK	Model/CBA	NMB	Health, public purse and service users	Net benefits of at least £7,870 per service user if employment sustained for 3 months	£921, HC	All sectors: £1,436 HC: 26% WF: 16% SOC: 23% OTH: 35%	1 year	70%
Hellström et al. [20]	Denmark	RCT/CEA, CUA	QALYs (EQ-5D), Employment	Societal	83–95% probability of being cost-effective at €30,000 WTP. Not cost-effective if cost per hour worked used	€1,183, NS	Overall: No significant difference WF: €4,262 SOC: −€3,376	1 year	78%
Heslin et al. [21]	UK	RCT/CEA	Employment	Health care	Higher rates of employment; cost-savings associated with IPS	NS, NS	Overall: No significant difference	2 years	56%
Hoffmann et al. [22]	Switzerland	RCT/ROI	Employment	Societal	Improved employment; Social ROI: 132%	CHF:14,917, NS	SOC: CHF 29,884	5 years	52%
Holmås et al. [23]	Norway	RCT/CBA	NMB	Societal	Higher rates of employment; cost savings associated with IPS	NOK 100,000, WF, HC	Total: NOK 317,000 WF: 67% SOC: 33%	3.6 years	65%
Howard et al. [24]	UK	RCT/CEA	Employment	Health care	No difference in employment, lower cost associated with IPS	£291, NS	HC: £1,880	1 year	56%
Khalifa et al. [25]	UK	RCT/CEA	Employment	Health care	9% higher rate of employment; No significant change in costs	NS, NS	No significant difference	1 year	70%
Knapp et al. [17]	Netherlands, UK, Italy, Bulgaria, Switzerland, and Germany	RCT/CBA, CEA	Employment, NMB	Societal	IPS associated with lower health care costs and higher rates of employment	£-4,022, HC	CEA: HC: £5,233 NMB: SOC: £17,005	1.5 years	91%
Mavranezouli et al. [26]	UK	Model/CEA, CUA	QALYs (SF-6D)	Health care	80% probability of being cost-effective at £30,000 WTP	£2,302, HC	HC: £1,700	8 years	92%
Mental Health Reform [27]	Ireland	Model/CEA	Employment	Provider	Cost per job was €8,374	€2,451, NS	WF: €1,614 per job	2 years	61%
Parlettaa and Waghorn [28]	Australia	Cohort study/CBA	Employment	Provider, public purse	Higher rate of employment; higher net revenue associated with IPS. Lower cost to government	$A 779, Government	Govt total expenditure per 26 week employment outcome: $A 3,855	1.5 years	52%
Perkins et al. [29]	UK	Model/ROI	Employment	Societal	ROI = 1.72 assuming 56% improved job rate in IPS group	£1,333, Govt	SOC: £2,319	2 years	56%
Rosenheck et al. [30]	USA	RCT/CBA, CUA	QALYs (QLS), NMB	Health care	94% probability of being cost-effective at $40,000 WTP. 80% probability of positive net monetary benefit if QALY valued at $20,000	NS, HC	No significant difference	2 years	87%
Saha et al. [31]	Sweden	RCT/CEA, CUA	QALYs (EQ-5D, MANSA)	Societal	Improved quality of life; cost savings associated with IPS	€-1,299, NS	SOC: €5,948	1 year	74%
Sambo [32]	Canada	RCT/CEA, CUA	Employment, QALYs (EQ-5D, QLS)	Health care	Improved employment; nonsignificantly higher quality of life, cost savings associated with IPS	$CAN 321.34, HC	No significant difference	1 year	91%
Shi [33]	Canada	RCT/CEA	Employment	Societal	Higher rate of employment and higher wages; cost savings associated with IPS	$CAN 4,778, HC	Three overlapping sets of benefits HC: $CAN 5,752 Govt: $CAN 5,739 SOC: $5,442	1.5 years	87%
Stant et al. [34] and van Busschbach et al. [35]	Netherlands	RCT/CEA, CUA	Employment, QALYs (MANSA)	Societal	80% probability of being cost-effective at €2,000 WTP per additional 1% employed. No difference in quality of life	€529, NS	Overall: No significant difference	2.5 years	83%
Stroupe et al. [36]	USA	RCT/CEA, ROI	Employment	Societal	95% probability of being cost-effective at $81 WTP per additional hour worked. ROI 0.329 for IPS versus 0.296 for transitional work	$3,839, HC	Overall: No significant difference	1.5 years	87%
Szplit [37]	UK	Observational study/CBA, ROI	Employment, NMB	Employment	ROI = 5.01; net present value: £449,063	£1,729, HC	Total: £11,709 WF: 51% HC: 15% Employers: 1% SOC: 33%	5 years	57%
van Stolk et al. [38]	UK	Model/CBA	NMB	Public purse	Benefit–cost ratio of £1.41 for every £1 spent to achieve employment outcome	£75,000 (per employment specialist), Govt	Total £118,913 HC: 9% Govt: 91%	1 year	70%
Washington State Institute for Public Policy [39]	USA	Model/CBA	NMB	Societal	Benefit–cost ratio of $7.70. 80% probability of positive NMB	$849, NS	All sectors $7,014 Govt: 30% HC: 0.002% SOC: 69.9%	50 years	74%
Whitworth [40]	UK	Model/CBA	NMB	Health care	Benefit–cost ratio of 0.32–7.47 depending on scenario	£1,200–£3,500, Govt	No cost breakdown provided	10 years	70%
IPS plus studies									
Christensen et al. [18]	Denmark	RCT/CEA, CUA	QALYs (EQ-5D)	Societal	95% probability of being cost-effective at €35,000 WTP	€2,543, NS	All sectors: €9,831 LGovt: 30% HC: 51%	1.5 years	95%
Reme et al. [41]	Norway	RCT/CBA, CUA	QALYs (EQ-5D), NMB	Societal	Improved quality of life. Positive NMB of NOK 7,694 per person per year for long-term welfare dependent	NOK: 28,043, WF	All sectors: NOK 35,737 WF: 11% HC: 10% SOC: 79%	1 year	87%
Schneider et al. [42]	UK	RCT/CEA	Employment	Health care	Only if WTP per wage hour reaches £100 is there a 50% probability of being cost-effective	£136, HC	HC: £86.8, 100%	1 year	74%
Yamaguchi et al. [43]	Japan	RCT/CEA	Cognition, Employment	Health and social care	Higher rate of employment. 78% probability of being cost-effective at $0 WTP	$1,287, NS	Overall: No significant difference HC: $2,682 100%	1 year	87%
Other supported employment									
Cimera [44]	USA	Cohort Study/CEA	Employment	Public purse	Higher costs per hour worked in supported employment than SW	$-252, Govt	NS	NS	61%
Cimera et al. [45]	USA	Matched cohort study/CEA	Employment	Public purse	Better weekly earnings, lower service costs for non-SW than the SW group	$-3,624, Govt	SOC: $62 per week	5 years	53%
Cimera [46]	USA	Matched cohort study/CEA	Employment	Public purse	Lower cost per dollar earned for the SE group than SW	$-3,352, Govt	SOC: $19 per week	8.5 years	56%
Cimera [47]	USA	Cohort study/CBA	NMB	Public purse	Benefit–cost ratios ranged from 0.63 to 2.77	$636 per month, Govt	WF: $770 per month	1 year	56%
Cimera [48]	USA	Match cohort study/CBA	NMB	Public purse	Benefit–cost ratios of 0.46, 0.56, 0.73 for individuals in the no transition, school transition and community transition groups respectively	$941–$1,345 per month, State Govt	WF: $54–$189 State Govt: $497	NS	56%
Cimera [49]	USA	Cohort study/CBA	NMB	Public purse	Benefit to cost ratio of 1.46 from a taxpayer perspective for all service users	$544, Govt	WF: $796	1 year	65%
Dattilo [50]	USA	Cohort study/CBA	NMB	Public purse	There were net benefits of $9,165 and $2,093 in the on-site and off-site groups	On-site $1,732, Off-site $284, State Govt (Rehabilitation)	On-site only HC: $10,897	0.5 year	56%
Evensen et al. [51]	Norway	Matched cohort/CUA	QALYs (EQ-5D)	Health and social care	85% probability of being cost-effective at €62,000 WTP	€9,131, WF	HC: €10,621	4 years	78%
Fogelgren et al. [3]	Sweden	Model/CBA	NMB	Societal	Gains from supported employment exceed costs after 12 years	€764–5900, Employment Services	SOC: €50 per month of employment	12 years	61%
Hagen 2018 [52]	Switzerland	Model/CBA	NMB	Public purse	Benefit cost ratio of 1.9–6.5 under different scenarios	CHF 8,819, Disability Insurance	WF: Between CHF 16,819 and €57,119	20 years	70%
Indecon [53]	Ireland	Cohort study/CEA	Employment	Public purse	Cost per job sustained €13,582	Monthly expenditure per client: €222–228, WF	NS	4 years	61%
Schneider et al. [54]	UK	Cohort study/CCA	Employment	Societal	The cohort who started work reduced their consumption of mental health services by an average of £23.93	3 month costs ranged from £31 to $238, HC	Per week: SOC: if working £70.53, not working £11.09 WF: if working £25, not working £12	1 year	65%
Sultan-Taib et al. [55]	Canada	Cohort study/CUA	QALYs (EQ-5D)	Health care	No impact on quality of life. Health care costs lower in comparison social firm group	NS, HC	HC: $CAN −$1,924	1 year	70%
Tholen et al. [56]	Sweden	Registry data /Model/CBA, ROI	NMB	Public purse	Benefit cost ratio of 3.09–4.99 under different scenarios	SEK 20 Million (69 young people), Local government	Municipality: SEK 21,128 Million	7.5 years	70%

Abbreviations: CCA, cost consequences analysis; CEA, cost-effectiveness analysis; Govt, government; HC, health care sector; LGovt, local government; MANSA, Manchester short assessment of quality of life; NMB, net monetary benefits; NS, not stated; OTH, other; QALYs, quality-adjusted life years; QLS, quality of life scale; RCT, randomised controlled trial; SOC, society; SW, sheltered workshop; WF, welfare sector; WTP, willingness to pay.

a Only significant differences for sectors reported.

## Individual Placement and Support

Of the 28 IPS studies focused on individuals with SMI (Supplementary Table S2), 23 had quality scores above 50% (Table 2). Of these, 6 studies reported cost per QALY gained, (5 favourable to SE/IPS), 8 NMBs (all positive), 4 ROI (3 positive), and 13 cost per employment outcome (9 favourable, 3 inconclusive, 1 negative). In total, 18 studies included impacts on health outcomes and/or service utilisation, as well employment [17–22, 24–26, 30–36, 38, 39]. Table 2 shows 16 of these 23 studies had a short timeframe (maximum 2 years) [17–21, 24, 25, 27–33, 36, 38], but there is growing evidence on longer-term economic impacts [22, 23, 26, 34, 35, 37, 39, 40]. For example, one Norwegian RCT compared IPS to traditional vocational rehabilitation for 327 people with moderate to severe mental illness [23]. Using registry data on employment and earnings it followed individuals for 43 months, reporting sustained increased levels of employment, equivalent to two full-time months per participant. Health service utilisation was lower although this was not significant. The cost–benefit analysis was limited in detail but reported net-benefits of $27,670 per participant. Cost-savings were due to increased time in work and reduced use of traditional active labor market programmes.

**Table 2.** Detailed data extraction for IPS economic studies (quality scores above 50% only).

			Economic analysis		Outcomes and key findings			
References and country	Setting and study population (age, sex, and size)	Intervention details (study design, description of intervention, comparator, and type of intervention)	Perspective Price year Currency	Type of economic analysis	Effect on mental health	Work-related outcome	Economic or financial outcome	Quality score
Christensen et al. [18] Denmark	482 people with severe mental illness: schizophrenia, schizotypal, or delusional disorders (F20–F29) 75.7 or 77.8% or bipolar disorder (F31) 13.2 or 10.5%, or recurrent depression (F33) 11.1 or 11.7% according to ICD 10 in both IPS and SAU groups. Mean age 33 in both IPS and SAU groups. 61.3 and 60.3% male in IPS and SAU groups, respectively. Data collected between 2012 and 2018	RCT comparing IPS (*n* = 243) compared with service as usual (SAU) (*n* = 239). Duration 1.5 years	Societal 2016 Euros	CEA, CUA	QALY gains were nonsignificantly higher in the intervention group than the control group (0.0329 vs. 0.0074, *p* = 0.2960). Mental health hospital care costs were lower in the IPS group €14,549 versus €18,279. (*p* = 0.0901). No significant difference in somatic hospital, primary health care or prescription costs	IPS participants earned an average of €1,792 more than the control group. Productivity gains in the intervention group −€7214 versus −€5422, *p* = 0.205). Labour market intervention costs were significantly lower in the IPS group. €403 versus €3,395 (*p* < 0.0001)	IPS was less costly, with nonsignificantly improved QALY gains compared to SAU. Overall costs, including productivity losses were significantly lower by a mean of €9,543 in the IPS group (*p* = 0.001). If there was a societal threshold of €35,000 for willingness-to-pay for a QALY, there is a probability of 95.6% of IPS being cost-effective compared to SAU. IPS also dominated with significantly lower costs and nonsignificantly higher hours spent in work or education	95%
Deloitte [19] UK	126 adults with unspecified mental illness using IPS services in Glasgow, Scotland. Age unspecified	Modelling study drawing on published literature, IPS data and expert opinion. The intervention was IPS and the comparator traditional vocational schemes (TVS). Duration: 1 year	Health, Public purse and service users combined 2016 UK pounds	CBA	40–60% reductions in Community Psychiatric Nurse (CPN) appointments and three less psychiatric appointments after 1 year after having secured employment. Total costs avoided of £96,710. NHS costs avoided per service user with more than 3 months competitive employment £1,981	Total service user benefit due to increased earnings £84,020	The annual cost of the IPS service was £116,000. Benefits of IPS: increased earnings £84,020, health care costs avoided £96,710, welfare benefits avoided £59,210. Net benefits £123,940. Additional net costs of TVS avoided £57,030 Total net benefit £180,970 Net benefits per user if employment sustained for less than or more than 3 months: £2,300 and £7,870	70%
Hellström et al. [20] Denmark	326 people aged 18–60 with an anxiety or affective disorder recruited from mental health centres and private psychiatrists in Copenhagen. Gender not reported. Participants should not have been in contact with mental health services for more than 3 years.	The intervention was IPS intervention modified for people with mood and anxiety disorders (IPS-MA) (162 people) compared to services as usual (SAU) (164 people). These could be social services (e.g., group therapy or psycho-social support interventions) or labour market services. Duration of study 2 years but only 12 month outcomes used in economic analysis	Societal 2016 Euros	CUA, CEA	When imputed cases data included mean QALYs gained were 0.056 and −0.17 in the IPS-MA and SAU groups (*p* < 0.05). The difference was not significant for complete cases only. 1 year mental health service use in the IPS-MA group was €5,489 compared to €8,161 in the SAU group (*p* = 0.078) Overall health care costs were not significantly different between the two groups	Mean wage earnings in the IPS-MA group were significantly lower, €5,034 versus €8,410 (*p* = 0.017) Labour market service costs were significantly lower in the IPS-MA group, €1,329 versus €5,591 (*p* = 0.009)	IPS-MA had a mean cost per person per year of €1,183. Overall, there was no significant difference in costs between the two groups, although costs were lower in the IPS-MA group €5,485 versus €7,706. *p* = 0.423. There was between an 83 and 95% chance at €30,000 per QALY gained of IPS-MA being cost-effective versus SAU. If cost per hour of worked gained was used instead the intervention would not be cost-effective with significantly lower levels of hours worked than SAU	78%
Heslin et al. [21] UK	219 individuals, with severe mental illness recruited between November 2004 and September 2006. Mean age 38, 66–69% male, 41 and 34% white, in IPS and control groups. 69 and 76% had a psychotic disorder. 31 and 24% had a mood disorder	RCT comparing IPS (*n* = 109) with treatment as usual/local vocational services (TAU) (*n* = 110). Duration: 2 years	Health care 2006/2007 UK pounds	CEA	There were no differences between intervention and control groups at follow-up on any clinical measures. Over 24 months, health care costs in intervention group were lower than controls (£9,571 versus £11,932), *p*-value not reported	Intervention had a significantly higher proportion in competitive employment than control group (22% vs. 11%, *p* = 0.041)	With lower costs and higher outcomes IPS was dominant. In probabilistic sensitivity analysis there was a 90% chance of IPS being the most cost-effective option	56%
Hoffmann et al. [22] Switzerland	100 individuals aged 18–64 with severe mental illness including schizophrenia spectrum, affective disorder), male (65%). Mean age 33.5 and 34.1 in intervention and support groups	RCT comparing IPS (*n* = 46) with traditional vocational rehabilitation (TVR) (*n* = 54). Duration: 5 years	Society Price year not stated Swiss Francs (CHF)	ROI	Intervention group had significantly less hospitalisation (21% vs. 46.7% *p* = 0.015), fewer psychiatric hospital admissions (0.4 vs. 1.1 *p* = 0.026) and spent fewer days in the hospital (38.6 vs. 96.8 *p* = 0.027)	Intervention group had higher rates of competitive work than traditional vocational rehabilitation (65% vs. 33%, *p* = 0.002), worked more hours per year (689 vs. 392, *p* = 0.023), earned more wages (CHF 11,826 vs. CHF 6,885, *p* = 0.004), had longer job tenures (104.8 vs. 35.5, *p* < 0.001)	Earning per client over 5 years CHF 66,977 versus CHF 37,093 in IPS and TVR. Mental health treatment costs per client CHF 25,484 versus 40,093. Vocational programme costs CHF 80,917 and CHF 43,701 Social ROI: 132.2%	52%
Holmås et al. [23] Norway	327 individuals (mean age = 35) with moderate (depression and anxiety disorders) to severe mental illness (psychotic or bipolar disorder with or without comorbid substance abuse/dependency), women (50%), from regional primary and secondary mental health care settings for 43 months	Original study based on RCT comparing IPS (*n* = 184) with treatment as usual (*n* = 143) 327 people before and after the IPS intervention. Duration: 3.6 years	Societal 2016 Norwegian kroner	CBA	Not reported	During 43 months, the intervention group had 8.8% higher rates of regular employment than in the control group, and 5% higher in regular employment with a half-time job or more (16.5% vs. 10.7%)	Net social benefit: NOK 217,000 (gain in productivity = 65,000 + cost-savings from traditional VR programme costs = 211,000 − programme cost(100,000) + cost-savings from excess burden of taxes 41,000, so 65,000 + 211,000000 − 100,000 + 41,000 = 217,000	65%
Howard et al. [24] UK	150 individuals (mean age = 38) with psychotic or chronic affective disorder in South London	RCT comparing IPS (*n* = 109) with local traditional vocational service, TAU (*n* = 110). Duration: 1 year	Health care	CEA	Psychiatric inpatient costs were lower in the intervention group (£719 vs. £2241), also lower costs for community mental health nurse costs than the control group (£49 versus £65)	There were no significant differences between the treatment as usual and intervention groups in obtaining competitive employment (13% in the intervention group and 7% in controls; *p* = 0.15), nor in secondary outcomes	Total costs were £2176 significantly higher in the control group (bootstrapped 95% CI £445–£4168). No significant differences in outcomes During the 1-year follow-up, two-thirds of the intervention group received input from employment workers at mean cost of £296. There were no substantial differences in the number of people using other services. However, control group participants who were admitted spent substantially more days in hospital than inpatients in the intervention group. This resulted in a difference in inpatient costs of £1522.	56%
Khalifa et al. [25] UK	18 individuals (mean age = 39.2) with schizophrenia, depression, personality disorder, with offending histories in community forensic settings over 12 months, male (88.9%)	RCT comparing IPS (*n* = 11) with Treatment as usual (*n* = 7). Duration: 1 year	Health care 2016 UK pounds	CEA	Brief Psychiatric Rating Scale scores were higher in the intervention group (34 vs. 25.5, *p*-value not reported), SF-12 scores in mental health were higher (53.1 vs. 43.8), EQ-5D (64.3 vs. 70), *p*-values not reported. Health care costs not reported separately from cost of IPS	Intervention group had higher rates in open employment at 12 months (9.1 and 0%) than TAU	Mean baseline costs £29,444 in IPS group versus £1,898 in TAU group. IPS less costly than TAU at 12 month follow up £1,799 vs. 1,940, significance not tested. Sample too small to draw conclusions on cost-effectiveness	70%
Knapp et al. [17] Netherlands, UK, Italy, Bulgaria, Switzerland, and Germany	312 individuals with SMI (schizophrenia and schizophrenia-like disorders, bipolar disorder, or depression with psychotic features, using ICD-10 criteria for 18 months in six European cities: Groningen (Netherlands), London (UK), Rimini (Italy), Sofia (Bulgaria), Ulm-Gunzburg (Germany), and Zurich (Switzerland)	RCT comparing IPS (*n* = 156) with standard vocational services (*n* = 156). Duration: 1.5 years	Health and social care Societal 2003 UK pounds	CBA, CEA	Readmission rates were lower in IPS than the control group (13% vs. 20%). Mean health care costs in the IPS group at first 6-month follow up were significantly lower than for control £4,688 versus £6,926 (Mean difference £2,720 95% CI −£4,624, −£813. Costs were lower in the following two 6-month follow ups but the difference was not significant	Over 18 months, IPS had higher rates of being at least 1 day in employment (55% vs. 28%) than those in vocational services	Mean costs for IPS across all sites were £18,877 versus £25,455 in controls (Mean difference £7,880 95% CI −£12,249, −£3151. Costs were significantly lower in three of the six sites: London, Ulm, and Zurich and the intervention was dominant, with lower costs and better outcomes in all areas except Groningen. In Groningen the additional cost per additional 1% of people working at least 1 day was £30 and additional cost per additional day worked was £10 Boostrapped mean net monetary benefits comparing the costs of intervention with value of employment achieved for both IPS and control were favourable at £17,005 in the IPS group	91%
Mavranezouli et al. [26] UK	Model drew on previous study of people with formal diagnosis of autism and IQ ≥70. Mean IQ score 98.8 (Wechsler Adult Intelligence Scale)	Markov modelling study comparing IPS versus standard care (day services). Duration: 8 years	Health care 2012 UK pounds	CEA, CUA	Mean QALYs gained over 8 years were 5.42 in the IPS group and 5.31 in the control group Secondary analysis including other health and social care costs, including mental health-care costs, other primary and secondary care costs and local authority costs revealed mean health care costs of £16,005 and £16,663 in the IPS and control groups	Over 8 years mean weeks in employment were 136 and 102 for the IPS and control groups	For the primary analysis, just including the costs of IPS and day care, the cost per QALY gained was £5,600 and cost per extra week of employment was £18. In probabilistic sensitivity analysis there was a 67 and 75.2% chance of being cost-effective at £20,000 or £30,000 per QALY gained. In secondary analysis including health and social care costs, the IPS intervention was dominant with lower costs and better outcomes. In probabilistic sensitivity analysis, there was an 80% chance of being cost-effective at £30,000 per QALY gained	92%
Mental Health Reform [27] Ireland	95 adults with severe and enduring mental health problems, not in paid employment who received IPS services from 2015 to 2017	A pilot IPS study, control group not reported. Duration: 2 years	Provider Price year not reported Euros	CCA	Not reported	In the project, 36% had at least one job placement. The average number of hours worked per week by successful applicants was 21 h, the average weekly wage for successful participants was €230. The combined staff and project costs totaled €276,326, with a cost per participant of €2,909	Cost per job outcome was €8,374. If start-up costs and project management excluded the cost per participant was €2,451 and the cost per job outcome was €7,057	61%
Parlettaa and Waghorn [28] Australia	175 individuals aged 15–64 (47% male) with schizophrenia or bipolar affective disorder, major depression, anxiety disorders, Posttraumatic Stress Disorder, personality disorder and substance abuse disorder	Observational cohort study comparing IPS (*n* = 68) with pre-IPS cohort (*n* = 107). Duration: 1.5 years	Provider and public purse	CBA	Not reported	Intervention group had significantly higher rates of job starts than pre-IPS services (67.6% vs. 56.1%) (Significance not reported).	Net revenues were higher in IPS than pre-IPS groups. The IPS enhanced service achieved higher gross revenue per participant ($9,062) than pre-IPS services ($7,514). The IPS enhanced programme generated more net revenue (gross revenue less direct costs) per participant compared to pre-IPS services ($6,929 versus $6,161), Cost per 26 week employment outcome achieved to government was $38,958 compared to $42,813 for pre IPS group. For higher severity group cost per 26 week employment outcome in IPS enhanced group was $48,693 vs. $167,199 in pre IPS group	52%
Perkins et al. [29] UK	Hypothetical 135,000 new IPS participants each year with unspecified mental health problems	Modelling study comparing IPS with Traditional service or no intervention. Duration: 2 years	Public Purse Price year not stated UK pounds	ROI	Not reported	Unpublished survey data for study involving employment workers across private, public and voluntary sectors was used to assume all clients would receive support for 6 months, with 35% continuing for 1 year and 25% for 2 years	Total cost of the programme £180 million per annum. 27,000 jobs would need to be created for the service to break even. This would mean a cost per job before fiscal benefits of £6,600; if 47,000 jobs were created the return on investment would be 1.72	56%
Rosenheck et al. [30] USA	404 individuals aged 15–40 with First Episode Psychosis, less than 6 months on lifetime antipsychotics in clinical treatment clinics. Demographic information not provided	RCT comparing IPS: Navigate (NAV), a comprehensive, multidisciplinary, team-based treatment approach for first episode psychosis, including IPS (*n* = 223) with community care (CC) (*n* = 181). Duration: 2 years	Health care US dollars	CBA, CUA	The NAV group had significantly greater improvement in PANSS total scores and improved significantly more on as a one standard deviation change on the Quality of Life Scale (QLS-SD) (*p* < 0.02). There was no significant difference in overall costs between the two groups. However, the intervention group had higher outpatient mental health ($1870 vs. 1379, *p* = 0.05), antipsychotic medication costs ($1739 vs. 1060. *p* = 0.01) Costs for all mental health and medical surgical inpatient care were lower in the intervention group but no significant difference ($3,694 vs. $3,780, *p* = 0.91)	Not reported	The incremental cost-effectiveness ratio was $12,081/QLS-SD, with a 94% probability that NAV was more cost-effective than CC at $40,000/QLS-SD. When converted to monetised quality-adjusted life years, NAV benefits exceeded costs, especially using future generic drug prices	87%
Saha et al. [31] Sweden	55 individuals with unemployed, depressive episodes, recurrent depression or bipolar disorder. Demographic information not reported	RCT comparing IES (individual enabling and support) (an IPS intervention) vs. TVR (traditional vocational rehabilitation) Duration: 1 year	Societal 2014 Euros	CEA, CUA	Intervention group was more effective using Manchester Short Assessment of Quality of Life (MANSA), but not EQ-5D. There were no significant differences in QALY improvement between groups. But quality of life measured by the MANSA scale significantly improved over the study period in IE. Health care costs were not included	The value of productivity gains was higher in the intervention group (€6059) than the control group (€111), *p*-value not reported	The cost of IES was €7247 lower per person per year, compared to TVR. The total cost for IES were €528 per person per year compared to €7775 for TVR. Intervention was dominant with no change in quality of life but lower costs	74%
Sambo [32] Canada	109 individuals aged 18–30 (mean age = 23) with Schizophrenia, psychosis, bipolar disorder, male (45%)	RCT comparing IPS: IPS + Early intervention for psychosis (TAU) (*n* = 56) with TAU (*n* = 53). Duration: 1 year	Health care Public payer 2016 Canadian dollars	CCA, CUA	Although the sample was small due to data collection issues, the EQ-5D-5L index scores were consistently higher for those in the TAU group compared with those in the IPS+ group. Scores on the QLS were consistently higher for the IPS+ TAU group compared with those in the TAU group. Overall health care costs, including primary care as well as specialist mental health care were nonsignificantly higher in the TAU group $3,884 vs. $3,656	At 12 months, proportion employed (IPS+ TAU vs. TAU):60% versus 50% but not significant. Nonsignificantly increased working days in intervention group: mean 8.38 more days, 57.24 vs. 48.86 days.	Total costs per patient in the IPS + TAU group were lower than those in TAU (mean difference $228, *p* = 0.823, 95% CI, $-2,261 to $1,806). Also improvements in employment outcomes in IPS group and quality of life but not significant	91%
Shi [33] Canada	149 individuals aged 18–64 (mean age = 40 or 41, female 37.3% and 39.2% in IPS or control groups 40.6) with severe mental illness: psychosis, bipolar disorder, major depression; and using outpatient psychiatric hospital services between 2001–2004	RCT comparing IPS (*n* = 75) with usual vocational services, including sheltered workshops, creative workshops, a consumer-run boutique, horticultural programs, job-finding-skills training and psychosocial intervention (*n* = 74) Duration of study: 1.5 years	Health Care, Public Purse, and Societal 2010–11 Canadian dollars	CEA	No significant differences in mental health care costs between the two groups: Inpatient costs ($1,421 versus $6,443, *p* = 0.2258), other mental health service ($639 versus $1,286, *p* = 0.9032), out-of-pocket costs of psychologist services ($11 vs. $10, *p* = 0.9921)	Over 12 months, significantly longer hours in competitive employment in IPS than the control group. Mean 126 h versus 72 h, *p* = 0.0004), higher wages in competitive employment ($935 vs. $514, *p* = 0.004)	Overall costs were lower for IPS compared to controls from all three perspectives: health and social care perspective: $25,709 (IPS) vs. $26,683 (UC) (*p* = 0.011). Public Purse: $32,984 versus $33,945, Society: $27,014 versus $27,678. The IPS programme was less expensive while improving outcomes, which means IPS dominates usual services	87%
Stant et al. [34] and van Busschbach et al. [35] Netherlands	151 with severe and long-term mental disorders who want to work. In IPS and control groups respectively: 55 and 64% psychosis, 17 and 10% mood disorders, 22 and 23% personality disorders, mean age 34.1 and 35.6, male 73 and 75%	RCT comparing IPS (*n* = 71) versus regular vocational rehabilitation (*n* = 80) Duration of study: 2.5 years	Health care Societal 2008 Euros	CEA, CUA	There were no differences between groups in quality of life at any time point measured using the MANSA – Manchester Short Assessment of Quality of Life. Mental health and general health care cost were higher but significance not reported. Overall mean costs including health, net of productivity gains were €57,285 and €43,819 in the IPS and control groups	After 2.5 years significantly more people in the IPS group were in regular paid work. Paid work during the study was significantly higher in the IPS group (44% vs. 25%) *p* < 0.05. More hours were also worked in the IPS group. Mean rehabilitation costs, including the cost of intervention were greater in the IPS group €1,705 versus €1,176	IPS has higher costs and better outcomes than regular vocational rehabilitation. The cost per additional 1 percent of individuals in paid work was €1,084. However, averted social welfare costs due to increased work participation are not included in the cost-effectiveness ratio. Here is an 80% probability of being cost-effective if society is willing to pay €2,000 per additional 1% in employment. The incremental cost per additional 1 point on the MANSA scale is €76,359	83%
Stroupe et al. [36] USA	541 military veterans (mean age = 41.2) with PTSD, men (81.7%)	RCT comparing IPS (*n* = 271) with transitional work (TW) programmes (*n* = 270) Duration: 1.5 years	Health care Societal 2019 US dollars	ROI, CEA	Mental health costs were insignificantly higher for IPS than TW ($1687 vs. $1498, *p* = 0.75) The annual mean cost per person of outpatient care were $3970 higher for IPS compared to TW ($23,245 vs. $19,276, *p* = 0.004) Overall mean health care costs including costs of vocational rehabilitation were significantly higher in the IPS group $29,691 vs. $23,298	The average number of hours worked in competitive employment per person per year was significantly higher in the IPS than the TW group (632 h vs. 458 h, *p* = 0.002). The mean annual income from competitive employment was higher in the IPS than the TW group ($9,762 vs. $7,326, *p* = 0.02)	IPS is more costly and more effective. 95% probability of being cost-effective if willing to pay $81 per additional hour worked. The average total costs per person per year were similar between groups ($29,828 vs. $26,772, *p* = 0.17). The incremental cost-effectiveness was $28 per additional hour of competitive employment. The return on investment (excluding TW income) was 32.9% for IPS ($9762 mean income/$29,691 mean total costs) and 29.6% for TW ($7326 mean income/$24,781 mean total costs)	87%
Szplit [37] UK	45 individuals with moderate to severe mental health problems in collaboration with community mental health teams from April 2010 to March 2011	1 year observational study, with longer term impacts modelled. Duration: 5 years	Employment 2010/11 UK pounds	CBA, ROI	Not reported	For 1 year, 40% (18 out of 45) of participants secured permanent employment	For every £1 invested with IPS there would be a return ranging between £5.01 and £6.77 in social added value The total present value (PV) of IPS for 2010/2011 is valued at £526,885. The total investment is £77,822 Total net present value less total investment figure (NPV) is £449,063	57%
van Stolk et al. [38] UK	People with depression, anxiety (common mental disorders) including employment, but also some people on sick leave	Modelling study comparing vocational support based on the Individual Placement and Support (IPS) model in IAPT or other suitable psychological therapy services. Duration: 1 year	Healthcare Public purse 2011 UK pounds	CBA	Assumes IPS service would support 120 clients per year. 35 would have reduced healthcare utilisation costs of £300 per year, including savings from fewer GP visits and limited use of secondary care	24.5 people would stop claiming job seekers allowance of £3,900	Positive cost benefit ratio = 1.41 from IPS; note this assumes that savings from reduced statutory sick leave so cost benefit ratio for long term unemployed alone not stated	70%
Washington State Institute for Public Policy [39] USA	Those with severe mental illness	Modelling study (Monte Carlo Simulation analysis for risk/uncertainty analysis) IPS versus traditional vocational rehabilitation. Duration: 50 years	Taxpayers, Participants	CBA	Net health care costs for psychiatric hospitalisation reduced by $8 per participant	Not reported. Net programme cost per participant: $849. Additional taxes from additional earnings to taxpayers $2,090 and to participants $4,910	Total positive benefits net of deadweight costs: $5,741. Benefit cost ratio = $7.7. Chance the program will produce benefits greater than costs 80%	74%
Whitworth [40] UK	Hypothetical 5,000 IPS programme starts over 30 months. Time limited to maximum 15 months. Assumed to have mental and physical health problems	Modelling study for IPS comparing alternative modified scenarios, the control group not reported. Duration: 10 years	Public purse Price year not reported UK pounds	CBA	Impacts on health care costs not included in analysis	Not detailed but costs averted include welfare benefits avoided, including council tax benefit and universal credit. Average annual earnings from employment assumed to be £11,800	ROI = 0.32 to 7.47, depending on models at 10 years. ROI at 5 years ranges from 0.19 to 4.53 depending on model scenario	70%

Some modelling analyses, synthesising evidence from multiple sources, have explored longer-term impacts. The Washington State Institute for Public Policy has generated economic models for many mental health interventions, including a 50-year model for IPS [39], reporting a benefit-to-cost ratio for IPS of 7.7:1.

A UK modelling study [40] examined potential costs and monetary benefits for up to 10 years, by varying assumptions about the degree of “modifiable” IPS fidelity items using two approaches, a “discrete” approach within secondary mental health services versus a “networked” approach, partnering with wider health, housing, debt, and employment services. Limiting IPS to 15 months maximum, caseloads per employment specialist varied between 20 and 30, with 12 scenarios showing a return on investment between 0.19 and 4.53 after 5 years and 0.32–7.47 after 10 years. By adopting a broader perspective in economic analyses, cost–benefits were greater and the longer the time horizon, the higher the expected returns.

Six IPS studies included QALY outcomes and the results were mainly positive. For example, the potential costs of IPS for people with autism were modelled over 8 years in the UK [26], with a cost per QALY of $9,231, well below the $28,964 (£20,000) to $43,042 (£30,000) threshold recommended by the National Institute for Health and Care Excellence, the body responsible for producing guidance on cost-effective interventions in England [57]. In a Danish RCT [18], IPS for people with SMI was less costly, with better quality of life measured using the EQ-5D. A Swedish RCT for affective disorders [31], found IPS less costly and more effective using the MANSA instrument. A Canadian RCT found positive impacts on quality of life measured with the EQ-5D, but data were only available for 30% of participants [32]. However, another Danish study using IPS for mood and anxiety disorders reported neither a significant change in quality of life nor in costs and was not considered cost-effective [20].

Nine economic evaluations alongside RCTs showed IPS was less costly as well as more effective than usual support, including traditional vocational rehabilitation. [17, 18, 21–25, 31, 33]. For example, a multicentre trial across six European cities reported IPS had lower mean health care costs at first 6-month follow up than controls, $9,414 versus $13,909 (Mean difference $5,462, 95% CI −$9,286, −$1,682) [17]. In other words, IPS can be considered as a dominant strategy, compared with usual care.

A 5-year trial in Switzerland reported a 132% return on investment from IPS, taking increased earnings and lower health costs for participants into account [22]. In addition, the 2-year Supported Work and Needs (SWAN) RCT in the UK [24] showed total costs for usual care were significantly higher by $4040, compared to IPS, given no significant difference in outcomes. In other words, IPS had significantly lower costs than treatment as usual with similar effects. This can also be interpreted as a good investment.

Only two IPS studies reported significantly higher health care costs for IPS participants. In the Netherlands, while health care costs were higher, the authors argued IPS could still be cost-effective given significantly improved employment outcomes [35]. There was also significantly increased use of outpatient mental health services for people with PTSD using IPS in the US over 18 months [36], but improved work outcomes offset these increased costs.

## IPS Plus Psychological Therapies

Meta-analyses have shown additional benefits of adding Cognitive Remediation (CR) and Cognitive Behavioural Therapy to vocational interventions [58]. Four studies looked at IPS augmented with these interventions, all with quality scores above 50% (Table 3). Two reported cost per QALY gained, (both favourable to SE/IPS), 1 positive NMBs, and 3 cost per employment outcomes (2 favourable, 1 inconclusive). In three RCTs studies, CR in Denmark [18] and Japan [43] and/or CBT [41] in Norway were added to IPS. Impacts on health and social care service use were reported in all studies. In Japan adding psychological therapies to IPS was dominant with lower health care service costs and better employment rates and cognitive functioning within 1 year [43]. The Danish study reported mental health care costs in the IPS plus CR group of $18,950 versus $25,205 (*p* = 0.0426) in the usual care group over 18 months, with better employment outcomes and a cost per QALY gained of $46,817 [18]. This would be considered cost-effective in Denmark, with an accepted societal willingness-to-pay threshold of $48,261 [18]. The Norwegian study also had lower health service costs, better quality of life, and positive net monetary benefits for participants on long-term disability benefits [41]. However, a small UK feasibility RCT [42] of IPS plus work-focused counseling CBT compared to IPS alone, was more costly and unlikely to be cost-effective.

**Table 3.** Detailed data extraction for IPS+ psychological therapies economic studies.

			Economic analysis		Outcomes and key findings			
References and country	Setting and study population (age, sex, and size) Dates of study	Intervention details (study design and duration, description of intervention, comparator, and type of intervention)	Perspective Price Year Currency	Type of economic analysis	Effect on mental health	Work-related outcome	Economic or financial outcome	Quality score
Christensen et al. [18] Denmark	477 people with severe mental illness schizophrenia, schizotypal, or delusional disorders(F20–F29) 76.1 or 77.8% or bipolar disorder (F31) 30 or 25%, or recurrent depression (F33) 27 or 28% according to ICD 10 in both IPS + CR and SAU groups. Mean age 33 in both IPS + CR and SAU groups. 63.5 and 60.3% male in IPS + SE and SAU groups, respectively. Data collected between 2012 and 2018	RCT comparing IPS supplemented with cognitive remediation and social skills training (IPS + SE) (*n* = 238) compared with service as usual (SAU) (*n* = 239). Duration 1.5 years	Societal 2016 Euros	CEA, CUA	QALY gains using the EQ-5D-5L in IPSE and SAU groups respectively were 0.0329 and 0.0074 (*p* = 0.0146) Mental health hospital care costs were significantly lower in the IPSE group €13,743 versus €18,279. (*p* = 0.0426) Prescription medication costs also significantly lower. No significant difference in somatic hospital or primary health care costs	IPSE group earned €756 more than the SAU group leading to mean productivity gain of €6418 in the intervention group Labour market intervention costs were significantly lower in the IPSE group. €415 versus €3,395 (*p* < 0.0001)	IPSE group had €4,545 lower costs for psychiatric hospital care compared to SAU group. (*p* = 0.0426). Overall costs, including productivity losses were significantly lower by a mean of €7,288 in the IPSE group (*p* = 0.0106). With better quality of life and lower costs IPSE was dominant over SAU. With a societal threshold of €0 for willingness-to-pay for a QALY, there is a probability of 88.3% of IPSE being cost-effective. At a societal willingness to pay of €35,000, the probability is more than 95% IPSE also dominated with significantly lower costs and nonsignificantly higher hours spent in work or education	95%
Reme et al. [41] Norway	1193 individuals aged 18–60 with common mental disorders on sick leave, at risk of going on sick leave or on long-term benefits in Norway. Mean age = 40.4 years, 33% male. 21.7% were on long-term benefits (>12 months sick leave) and 7.9% were unemployed	RCT comparing IPS (individual job support) + work-related cognitive behavioural therapy (*n* = 630) with usual care (*n* = 563). Duration: 1 year	Societal 2011 Norwegian Krone (NOK)	CBA, CUA	Intervention had a significant reduction in depression (*p* ≤ 0.001) and anxiety symptoms *p* = 0.012). Significant improvement in quality of life (EQ-5D scores 65.64 vs. 61.57 *p* = 0.026) for intervention compared with usual care. Annual health service costs were lower in the intervention group NOK 2287 versus NOK 6020 (significance not reported)	At 12 months, intervention group had higher job retention compared to the control group (44.2% vs. 37.2%, *p* = 0.015) and this difference persisted at 18 months (*p* = 0.018). The difference between the intervention and control group was largest for those on long-term benefits at baseline, 30% increased or maintained work participation versus 11% at 18-month follow-up	Mean costs for the IPS service were NOK 28,043 per service user per year. Overall additional costs in the intervention group were NOK 28,454. Overall benefits did not outweigh costs with net present value of gains being NOK -3,681 per person However, for those on long-term benefits only at baseline there would be positive net benefit of NOK 7,694 per person per year.	87%
Schneider et al. [42] UK	74 individuals, 37 in intervention and control groups, and aged 18–60 with severe mental illness within Nottinghamshire Healthcare NHS Trust in 2010–2012. 70% were male; 70 and 64% in intervention and control groups were White. Mean ages were 30.48 and 29.48. 65 and 68% had psychotic disorders, 11 and 19% had bipolar disorders, 16 and 11% had depression	RCT of IPS only (control) or IPS combined with work-focused counselling (intervention). Duration: 1 year	Health care 2012 UK Pounds	CEA	There were no statistically significant differences between the two groups at any time point on the mental health outcomes (*p*-value not reported for the between group differences) Mean total health care costs in the intervention and control groups were £1,507 and £1,112 (*p* = 0.739)	The intervention showed no difference in the average number of hours in paid employment (2.1 vs. 3.7 h per week) in the two groups (*p* = 0.681)	Total intervention costs (£2397) were higher than the control group (£1880) but not significant (*p* = 0.290). Costs of the work-focused intervention were estimated at £136 per person on average. In probabilistic sensitivity analysis only if the decision-maker is willing to pay about £100 per paid hour does the intervention reach a 50% chance of being cost-effective	74%
Yamaguchi et al. [43] Japan	111 individuals aged 20–45 with schizophrenia, depression, bipolar from hospital outpatient settings in four Japanese prefectures: Tokyo, Chiba, Miyagi and Kyoto. Approximately 60% were males, and over 80% were diagnosed with schizophrenia. Mean age was 35	RCT comparing IPS+ Cognitive remediation (a social skills training): CR + IPS, (*n* = 57) with traditional vocational services TVS (*n* = 54). Duration: 1 year	Health and Social Care 2015 US Dollars	CEA	Intervention group showed better cognition in Brief Assessment of Cognition in Schizophrenia Japanese language version, BACS-J, *p* < 0.001), and no significant differences in depression (*p* = 0.739) Medical costs were significantly lower in the CR + IPS group $3,394, SD $3655 versus $6616, SD $10,646. *p* = 0.042, due to lower use of inpatient services	At 12 months, the CR + IPS group had higher rates of competitive employment than the TVS group (28% vs. 9%, *p* < 0.001). Mean length of employment was also longer (78.62 versus 24.87, *p* = 0.001)	Total mean cost in the CR + IPS group were lower than the TVS group ($9823, SD $6372 vs. $11,063, SD $11,263) (*p* = 0.412). The CR + IPS group was therefore dominant over TVS, with better outcomes and no significant difference in costs In probabilistic sensitivity analysis with a willingness to pay of zero there was a 70% likelihood of CR + IPS being more cost-effective than TVS in gaining an additional 1% of people working. There was also a 78% chance of CR + SE being more cost-effective than TVS per unit improvement on the BACS-J scale	87%

## Other Supported Employment Interventions

Of the 24 non-IPS SE studies (Supplementary Table S3) 14 had quality scores above 50% (Table 4), including 8 whose populations included some people with learning disabilities [3, 44, 45, 47–49, 53, 56]. Two studies in Table 4 reported cost per QALY gained, (one favourable), seven NMBs (five positive), one positive ROI, and four cost per employment outcome (three favourable and one inconclusive).

**Table 4.** Detailed data extraction for supported employment economic studies (quality scores above 50% only).

References and country	Setting and study population (age, sex, and size) Dates of study	Intervention details (study design and duration, description of intervention, comparator, and type of intervention)	Economic analysis		Outcomes and key findings			Quality score
			Perspective Price Year Currency	Type of economic analysis	Effect on mental health	Work-related outcome	Economic or financial outcome	
Cimera [44] USA	40,118 supported employees in the United States whose cases were closed in 2013. 60.4% were male, 70.6% were White. Primary impairments: learning disabilities 51%, mental disorders 36%. Age not reported	Analysis of electronic records in the US Rehabilitation Services Administration 911 database. The intervention was supported employment services. There was no comparison group but costs of service use were compared with those for sheltered workshop users. Duration: not stated	Public purse 2013 US Dollars	CEA	Not reported	53% of services users were in employment and had their cases closed. The mean cost per month of supported employment was $342.07 (SD $418.45). The mean cost per month for sheltered workshops reported in literature was $728.58. 12.8% of supported employees had mean costs per month in excess of mean sheltered workshop costs	Mean cost to vocational rehabilitation services per hour worked was $7.23 (SD $15.46). The mean cost reported in literature to sheltered workshops per hour worked was $9.94. 20.4% of supported employees had mean costs per hour worked in excess of mean sheltered workshop costs per hour worked. There was substantial variation in costs per hour worked across vocational rehabilitation agencies	61%
Cimera et al. [45] USA	215 supported employees with autistic spectrum disorders who participated in sheltered workshops (SW) prior to becoming supported employees and 215 supported employees with autistic spectrum disorders who did not participate in sheltered workshops (NSW) prior to becoming supported employees. Mean age of the SW and NSW groups was 31.12 and 37.75; 80% in both groups were male. 78.5 and 83.3% of the SW and NSW groups were White. 74.8% in both groups had a secondary disability. Data were taken from records from 2002 to 2006	Analysis of electronic records in the US Rehabilitation Services Administration 911 database. The intervention was supported employment, with or without prior sheltered workshop experience. Duration: 5 years	Public purse Price year not reported US Dollars	CEA	Not reported	45.6% of the former SW group were employed when their cases were closed compared with 39.5% of the NSW group workers. The SW group worked a mean 23.5 h (SD = 11.4) per week; compared with 25.0 (SD = 12.3) hours in the NSW group. Neither difference was significant. Former SW who became competitively employed earned a mean $129.36 (SD $89.66) per week, compared with $191.42 (SD = US$118.83) (*p* = 0.001) for NSWs. Former SWs had mean service costs of $6065.08 (SD 9879.33) per person. Costs for the NSW were significantly lower at $2440.60; SD $4585.63) (*p* < 0.001). Mean service costs for the employed in the NSW group were also significantly lower than those for the employed in the SW group: $4212.24 (SD $5088.11) compared to $8364.39 (SD $11,420.70) (*p* = 0.001)	Individuals from the NSW group had better outcomes—weekly earnings, while service costs were significantly lower than the group with prior SW used	53%
Cimera [46] USA	112 individuals who were in supported employment and/or sheltered work programmes. Age and gender not reported. Data from service use between January 2000 and June 2008. Disability breakdown not provided	Cohorts drawn from dataset for all sheltered and supported employees in a US State. 20 participants were in both supported and sheltered employment programme. 92 were in matched pairs in either sheltered work or supported employment. Duration:8.5 years	Public purse 2008 US Dollars	CEA	Not reported	In the matched pair analysis the SE cohort received services for 46 months (SD 26.71) compared to 70.02 (SD 31.28) months in the SW group (*p* < 0.001). SE worked fewer hours per month than the SW group—60.55 (SD 16.18) hours versus 65.41 (SD 34.65) hours but this was not significant. The cost per month of services received was $496.41.(SD $399.13 in the SE group versus $602.36 ($327.98) in the SW group. This difference was not significant. The SE group had significantly higher monthly wages of $403.34 versus $159.77 in the SW group (*p* < 0.001). Including data from the 20 individuals who participated in both programmes did not change findings	For the matched pairs the SE cohort had a nonsignificantly lower cost per hour worked of $10.83 (SD $15.35) compared with $14.13 (SD $14.46) The cost per dollar earned for the SE group was $2.01 (SD $4.33) versus $12.24 (SD $20.03) (*p* < 0.001) for SW employees. Including data from the 20 individuals who participated in both programmes did not change findings	56%
Cimera [47] USA	104,213 individuals with intellectual disabilities funded by state vocational rehabilitation agencies throughout the entire United States and its territories from 2002 to 2007 who wished to be enrolled in supported employment. 56.9% were male. Mean age was 33.89, 71.8% were White and 47.5% had a secondary diagnosis. Data were taken from records between 2002 and 2007 inclusive	Analysis of electronic records in the US Rehabilitation Services Administration 911 database. The intervention was supported employment services received through state vocational rehabilitation agencies. There was no comparator, but it was assumed that individuals who did not obtain competitive employment would receive sheltered employment. Duration: 1 year	Public Purse 2008 US Dollars	CBA	Not reported	For all service users the mean gross monthly costs of providing supported employment were $636.45, while gross net monthly benefits to the taxpayer were $769.54. Impacts on employment not reported	Net monthly benefits were $133.10, with a benefit–cost ratio of 1.21. Service users with or without secondary conditions had similar net benefits of $113.03 and $128.24 with benefit: cost ratios of 1.19 and 1.23, respectively. Benefit–cost ratios across US states vary greatly between 2.77 in Nebraska and Illinois in 0.63	56%
Cimera [48] USA	246 supported employees who completed at least one job cycle (i.e., they obtained and eventually separated from a job in the community). 185 received no transition services when they were in high school. 31 individuals received special education including transition planning only. 30 received community-based transition services in high school. Mean age of no transition, school-transition and community-transition groups was 36.24, 25.59, and 23.86. Males accounted for 50.3, 61.3, and 63.3%, respectively. Primary disability: mental health 32.4, 12.9,and 6.7%, respectively; mild learning difficulties 47.6, 45.2, and 36.7%, moderate learning disabilities 6.5, 25.8, and 36.7%, severe learning disabilities 10% in community transition group only. 48.7, 35.7,and 37.9%, respectively had a secondary diagnosis. Study dates not provided	Analysis of cohort of supported employees who had either: (a) received no transition services in high school, (b) had community-based work experiences in high school, and (c) individuals who had individualised education programmes (IEPs) in high school but experienced only in-school transition services. It was assumed that if an individual was not in supported employment they would have been in sheltered workshops. Secondary analysis matched pairs of individuals to compare no transition versus community transition and no transition versus school transition. Duration: not stated	Public Purse 2008 US Dollars	CBA	Not reported	No transition services generated mean per capita gross monthly benefit to taxpayers of $619.4 and mean per capita gross monthly cost of $1,345.02. School transition service group had mean per capita gross monthly benefit of $551.27 and gross monthly cost of $979.02. Community-based transition services group had $686.10 in gross benefits and $940.95 in gross costs For 21 matched pairs in the no transition versus community transition groups, months employed were 3.24 and 7.32 *p* = 0.001 respectively. For 17 matched pairs in school transition versus community transition months employed were 4.70 and 8.10 (*p* = 0.0006)	Individuals in the no transition, school transition and community transition groups had benefit–cost ratio of 0.46, 0.56, and 0.73, respectively For matched pairs in the no transition versus community transition group the respective benefit–cost ratios were 0.41 and 0.61. In 85.7% of cases, individuals with community-based transition services were more cost-efficient For matched pairs in the school transition versus community transition group the respective benefit–cost ratios were 0.37 and 0.59. In 88.2% of cases, individuals with community-based transition services were more cost-efficient	56%
Cimera [49] USA	All 231,204 supported employees from 2002 to 2007 who were served by vocational rehabilitation (VR) throughout the entire United States and its territories. 57.2% Male, 74.1% White, Mean Age 32.2. Primary condition: 40.3% learning disabilities, 29.6% mental illness.48.7% had secondary condition. Data were taken from records between 2002 and 2007 inclusive	Analysis of electronic records in the US Rehabilitation Services Administration 911 database. The intervention was supported employment services received through state vocational rehabilitation agencies. There was no comparator, but it was assumed that individuals who did not obtain competitive employment would receive sheltered employment. Duration: 1 year	Public purse 2008 US Dollars	CBA	Not reported	For all service users, the mean gross monthly costs of providing supported employment were $544.31, while gross net monthly benefits to the taxpayer were $795.65. Net benefits were $251.34. For mental health service users the mean gross monthly costs of providing supported employment were $481.76, while gross net monthly benefits to the taxpayer were $807.69. Net benefits were $325.92. For learning disability service users mean gross monthly costs of providing supported employment were $651.47, while gross net monthly benefits to the taxpayer were $781.21. Net benefits were $129.74. Impacts on employment not reported	From a taxpayer perspective for all service users the benefit: cost ratio was 1.46. For mental health and learning disability service users it was 1.68 and 1.20, respectively. For mental health and learning disability service users with or without secondary conditions benefit cost ratios were 1.67 or 1.69 and 1.17 and 1.22, respectively	65%
Dattilo [50] USA	65 people with mental health conditions referred by California State Department of Rehabilitation to Caminar’s Jobs Plus programme at San Mateo County within the 2017–2019 fiscal years. Age and gender not stated	Analysis of data from a supported employment agency, Jobs Plus. On-site versus off-site job coaching support as part of a supported employment programme Jobs Plus using the IPS model. 22 people chose off-site coaching and 42 people chose on-site coaching. Duration: 180 days	Public purse Not stated US Dollars	CBA	On-site coaching had a greater reduction in hospitalisation days, from 5.12 days before referral to 1.79 days after. For off-site this was 3.05 days to 2.32 days. Significance not reported	Those using off-site coaching had an average of 8.41 weeks on the job during the 90-day probationary period, while those using on-site coaching had an average of 10.29 weeks on the job. Significance not reported. 71.43% of on-site group had cases successfully closed versus 54.55% in off-site group.	There were statistically significant reductions in health care costs of $10,897 in the on-site group post intervention. This compared with a reduction of $2,377 in the off-site group. Mean costs of providing coaching in the off-site and onsite groups were $284 and $1,732. There were net benefits of $9,165 and $2,093 in the onsite and off-site groups	56%
Evensen et al. [51] Norway	169 individuals aged between 18 and 65 with a broad schizophrenia spectrum disorder. Mean age 33.2 and 34.9 for intervention (JUMP) and treatment as usual (TAU) groups. 65% of both groups were male. 87 and 100% of JUMP and TAU groups had schizophrenia	Analysis of registry data linked to participants in a multisite vocational rehabilitation (VR) programme (JUMP programme) for adults with schizophrenia spectrum disorders. The programme provided 10 months of standard VR services in competitive or sheltered workplaces. This was augmented with cognitive behavioural therapy (CBT) or Cognitive Remediation (CR). The comparator was TAU. Data were analysed 2 years before (T0) and 2 years after entering JUMP (T1). Duration: 4 years	Health and social care system 2015 Euros	CUA	For JUMP group at T0 mean QALYs were 0.8003 (SD 0.024) and 0.8043 (SD 0.024) at T1. Specialist mental health costs reduced significantly in the JUMP group both for those who gained paid employment (*n* = 21; mean = € −80,776; 95% CI -140,112, −21,467; *p* = 0.010) and those who had work placement or sheltered work (*n* = 42; mean = € −90,885; 95% CI −153,873, − 27,897; *p* = 0.006). JUMP group had 25.5 inpatient days at T1 compared with 64.6 in TAU group (*p* = 0.003)	23.2and 21.4% of JUMP and TAU groups were in employment at 2 year follow-up. Mean months of competitive employment in JUMP group were 3.10 (*N* = 69, SD 7.27) at T0 and 4.30 (*N* = 69, SD 7.33) at T1. Authors assumed stable 10.2% employment rate in the TAU group	Mean cost of JUMP intervention was € 9131 (SD 2123) per participant. Mean duration of JUMP was 26.52 weeks (SD 5.89). Total mean costs for the JUMP group (inclusive of intervention costs and adjusted for baseline differences) were € 10,621 lower than for TAU (95% CI: −29,979, 8735; *p* = 0.282). With QALY gains and lower costs compared to TAU—JUMP was considered cost-effective. It had an 85% likelihood of being cost-effective at willingness to pay threshold of €62,000 in probabilistic sensitivity analysis	78%
Fogelgren et al. [3] Sweden	1,062 young adults on disability pensions across 25 Swedish municipalities. Mean age was 25. Between 45 and 51% were female. 73% had mental health conditions, 17% learning disabilities and 10% other conditions. The study took place between November 2014 and December 2016	Modelling study based on randomised trial lasting 1.5 years comparing supported employment (SE) with regular vocational rehabilitation (VR) which includes “in-house” work preparation and work training, as well as case management. Duration: at least 12 years	Societal Price year not stated €	CBA	Not reported	At 18 month follow up 32% of the SE group were employed versus 22% for VR. This was statistically significant, but the definition of employment includes subsidized competitive employment	The costs per participant for SE group were €2,781 compared to €2017 for VR using a bottom up costing approach. SE costs were €5,900 more expensive per participant using a top down costing approach Based on an average difference in costs between interventions (€3,350) and assuming 0.33 of difference in employment probabilities between SE and VR is permanent, gains from supported employment exceed costs after 12 years	61%
Hagen [52] Switzerland	908 adults who had been on long term disability benefits who received intervention and matched controls from a sample of 14,878 who did not have intervention. Data collected between 2009 and 2011. Mean age 43.7, 53% male. 55.7% had one or more mental disorders	Economic modelling analysis using administrative linked datasets. The intervention was placement coaching by individually assigned advisers/coaches. Participants received active support in, and practical tips on, their search for suitable jobs for up to 12 months. Once in a job they also received support from coaches for a further 12 months. Duration 20 years	Public purse Not stated Swiss Francs (CHF)	CBA	Not reported	Significant reductions in claims for disability insurance for intervention group compared to matched controls, with up to an 8% reduction or 146 CHF per month 4 years after intervention start. (*p* < 0.01). Income earned from paid employment increased by up to 13.2% three years after intervention start or additional 4,017 CHF per annum	Total cost per participant was CHF 8,819. Four long term modeling scenarios created. Under all scenarios there are positive net benefits comparing change in disability benefit and increase in taxes paid with programme costs. Expected mean long-run benefits exceed the mean costs by 1.9–6.5 times, or between CHF 8,000 and CHF 48,300 per participant	70%
Indecon [53] Ireland	3,151 adults referred to 23 EmployAbility services in Ireland, each having a specific geographical remit. Data were collected between 2010–2014	Analysis of official data on service use for The EmployAbility service, formerly known as the Supported Employment Programme (SEP), a national employment service dedicated to improving employment outcomes for jobseekers with a disability. Duration: 4 years	Public purse Not stated Euros	CEA	Not reported	46.9% of clients exited programme while in employment and 83.3% of these then had 6 months of employment without support. Only 28% of all exits sustained at least 6 months of employment	Average monthly expenditure per client varied from €222 to €258 per month over 2010–2014. Mean cost per client supported between €3,996 and €4,644 over this period. Mean spend per client exit to employment has fallen from €19,032 in 2012 to €11,433 in 2014. Mean service expenditure relative to employment sustained was €13,582 over the period 2013–2014	61%
Schneider et al. [54] UK	141 users of support employment services. Three groups: already working and remained in same job, those who obtained work just prior to baseline or 12-month follow-up and those who remained unemployed throughout year. 43.3% were female, 83% White, 25.5% schizophrenia, 24.1% anxiety, 31.9% depression, 15.6% bipolar disorder.	Cohort analysis. Intervention was supported employment services that were close to IPS model. Comparison made between those who obtained work just prior to baseline or 12-month follow-up and those who remained unemployed throughout year Duration: 1 year.	Public purse and societal Price year not stated UK Pounds	CCA	For employed <12 months mental health service weekly costs were significantly lower at follow up compared to baseline. £14.30(SD £23.97) versus £36.71 (SD £45.76) *p* < 0.001. Similarly, for employed >12 months baseline £43.35 (SD £82.12) versus £23.19 (SD £46.33) at follow up *p* < 0.01. There was no change for the unemployed group. Overall weekly costs were significantly lower at follow up in the <12 months group £30.34 (SD £69.20) versus £40.00 (SD £45.51). Weekly overall health costs were also lower in the >12 months group £28.31 versus £47.86 but this was not quite significant	Individuals entering work increased net mean earnings by £59 per week *p* = 0.02. This was much greater than welfare benefits and allowances lost as a result of employment	Costs of supported employment increased significantly for people who had worked for less than 1 year (*p* < 0.04) while they declined for those who remained unemployed (*p* < 0.001) as well as for those who had been working for longer (*p* < 0.002). The median weekly cost of supported employment input was £15.75, and the cohort who started work reduced their consumption of mental health services by an average of £23.93 (range: £4.93–£52.78, *p* = 0.103)	65%
Sultan-Taib et al. [55] Canada	122 employees working in 19 Social Firms (SFs) across Quebec and 64 individuals participating in 2 supported employment programmes (SEP) in Montreal. 74 and 46% of participants were male. Mean age was 46 and 39.9. Primary mental health diagnosis: Schizophrenia 58 and 39%, Bipolar 9 and 6%, Major Depression 20 and 5%, Others 34 and 14%	Cohort analysis. Noncompetitive employment in social Firms was compared with supported employment programmes. Duration: 1 year	Healthcare 2015 Canadian dollars	CUA	There was no significant difference in mean EQ-5D-5L (quality of life) scores 76.87 versus 72.6 in the SF and SEP groups. Mean annual healthcare costs were $3,600 (SD $4,304) in the SF group and $9,403 (SD 11,888.9) in the SEP group. Mean costs for outpatient visits, inpatient stays, medications were all significantly lower in the SF group		There was a difference in health care costs of $5,803 (95% Confidence Interval [CI]: 3,433.2–8,173.3; *p* < 0.001) in favour of SF. Controlling for covariates including EQ‐5D‐5L score, severity of psychiatric symptoms, gender and age The multivariable analysis showed that the annual costs per patient were significantly lower for the group of participants working in SFs $2,580 versus $4,774 a ($1,923.9 lower, 95% CI: 1,146.3–3,127.7; *p* = 0.004)	70%
Tholen et al. [56] Sweden	118 former pupils with learning disabilities, 69 of whom previously took part in supported employment programme in three upper secondary special schools in Örebro, Sweden. 56% were male. Data were collected between 2006 and 2013	Modelling study drawing on data from before and after analysis using Registry data on employment status up to 4 years after leaving school. The intervention (Job In Sight – JIS) was supported employment was very similar to IPS but used internships as an intermediate step before full employment. A control group of 49 pupils left school before the JIS programme started. Duration: 4 years	Local government 2013 prices Swedish Krona	CBA, ROI	Not reported	Employment rates in the JIS group increased from 35% in the first year (*T*) to 67% in the last year (*T* + 3). Employment rates in the control group increased from 10% in the first year (*T*) to 35% in the last year (*T* + 3) In the model an increase of the work share by one percentage unit was assumed to reduce the share of former pupils in day-activity programmes by one percentage unit	The cost per pupil of intervention was 290,000 SEK for 3 years of support, equivalent to 114% of the cost of a full school year. JIS would have a positive benefit cost ratio. Depending on discount rate (3 or 3.5%) used the benefit cost ratio was 4.987 or 3.09, respectively. There would be a net present value of 0.592 million SEK It would take 7.5 years to generate a positive return on investment	70%

Only five SE studies looked at health outcomes and/or service utilisation [50, 51, 54, 55, 59]. Four reported lower healthcare costs [50, 51, 54, 59], for example, a Norwegian RCT comparing SE plus CBT/CR to usual care had better QALY gains with lower healthcare costs after 2 years [51]. In contrast, a Canadian analysis reported significantly lower health care costs for people working in social firms, rather than in supported employment [55]. It argued this may have been due to the therapeutic nature of social firm support.

Five out of seven cost–benefit analyses of SE interventions had positive net benefits [3, 47, 49, 52, 56], for instance, $6,627 to $40,012 per participant in Switzerland under different scenarios over 20 years [52]. A US study on SE for autism, intellectual disabilities, and other mental health conditions, from a public purse perspective, had various benefit-to-cost ratios, depending the presence of comorbid conditions (without secondary diagnosis = 1.46 versus with secondary diagnosis = 1.49), with the highest net benefits of 2.2 shown for learning disabilities over 6 years [49]. Another US study of SE for intellectual disabilities [47] estimated that every $1 investment led to $1.21 of monetary benefits from taxes paid and programme costs forgone. However, there were wide geographical variations across states in benefit–cost ratios ranging from 0.63 in Illinois to 2.77 in Nebraska. In another US analysis, the monthly benefit–cost ratio for supported employment for people living mainly with mental health conditions or mild learning disabilities was at best 0.73:1 from a taxpayer perspective [48].

Modelling long-term outcomes of a RCT of a Swedish SE service adherent to most IPS principles, but delivered by public employment services, was found to have positive monetary benefits [3]. However, uncertainty over long-term costs and sustaining employment meant the time to achieve positive benefits might be between seven and 12 years. Another Swedish intervention, similar to IPS but using employment internships as an intermediate step to employment, was targeted at pupils in their final school year [56]. Registry data confirmed participation was associated with increased rates of future employment, generating positive net economic benefits after 7 years. In the US a long-term (up to 8.5 years) observational analysis of 112 people in SE or sheltered workshops also found the former had six-fold lower costs per dollar earned [46].

## Discussion

To our knowledge, this is the most comprehensive systematic review on economic evaluations of SE/IPS to date, covering modelling and trial-based studies for all mental health conditions. Our systematic review reveals 56 papers covering 54 economic evaluations examining the economic case for SE/IPS for people living with all mental health conditions. Other reviews revealed only a handful of studies, because of their narrower scope on SMI [8, 60].

Our findings are consistent with commentaries looking at earlier studies in the 2000s and 1990s [10, 61]. SE/IPS, when well-implemented can lead to significantly improved work-related outcomes and/or reductions in welfare payments at least in the short term, which partially or even completely offset the costs of intervention. Well-designed RCTs also demonstrate cost-effectiveness from a healthcare perspective; the economic case can be strengthened further when multiple impacts across employment/welfare, health, and other sectors are considered.

In total, 43 (79%) of our included studies reported a positive economic case for intervention with just three (5%) being negative and eight (15%) inconclusive or dependent on subjective judgment. We assessed the quality of these economic studies using the CHEERS checklist; 41 had quality scores above 50%, including 24 with quality scores over 70%. Twelve studies that scored 70% or more on quality focused on IPS, were linked to RCTs, and concluded that it was cost-effective [17, 18, 20, 25, 30, 31, 33–36, 41–43]. Only two IPS studies were from the USA [30, 36]; most IPS studies were European. Moreover, SE/IPS studies have grown particularly in the Nordic countries [3, 18, 20, 23, 31, 41, 51, 56], where IPS services have been implemented and there is good access to welfare and health registry data to follow-up service users.

### Implications for policy and practice

Despite evidence on the effectiveness of SE/IPS strategies helping people with mental health conditions to obtain competitive employment, the availability of services remains limited in many countries, where traditional “train and then place” services continue to dominate [62]. One barrier to the scale-up of services is fragmentation of funding and responsibility for SE/IPS services, leading to budgetary silos. As Table 1 indicates, usually only one sector, such as health, is responsible for funding services, whilst there is a perception that most of the documented economic gains accrue to other sectors, such as through reduced welfare payments, less need for active labour market programmes, and higher earnings. Another barrier is the lack of sustainability. SE/IPS programmes as alternatives to traditional vocational rehabilitation services may not have secure mainstream funding [63, 64].

Overcoming these challenges requires more intersectoral collaborations between health and welfare sectors. Potentially this could include pooled budgets and joint delivery contracts to overcome budgetary silo issues that impede implementation [65]. Economic evidence can help inform contracting arrangements across sectors, quantifying the economic costs and value of gains to different sectors, as well as the time required for positive economic gains to be achieved. As Table 1 indicates, 24 of the 41 studies had total benefits that covered the additional costs of SE/IPS programmes; societal, government, welfare, and health benefits alone would completely cover these costs for 11, 6, 6, and 8 programmes, respectively. By knowing more about which sectors pay and which sectors gain, it is possible to demonstrate that multiple sectors make gains from SE/IPS, or alternatively estimate the level of financial risk sharing needed across sectors to incentivise implementation. To do this requires more analysis of the impacts across key sectors, including health, as well as a clear indication in all studies of which budget holders are responsible for funding SE/IPS services.

### Implications for future research

We put forward a number of suggestions for future research. These include more assessment of long-term cost-effectiveness, making use of registry data, and/or modelling. Cost-effectiveness of implementation of SE/IPS through evaluation under naturalistic conditions rather than just in trials, as well as more assessment of combined interventions and those targeted at specific risk groups is also needed. The interpretation of economic evaluation can be strengthened by more mixed methods research that looks at the context and fidelity under which SE/IPS are implemented.

While economic studies in this review have shown the superior cost-effectiveness of SE/IPS compared to usual care, only six looked at outcomes beyond 5 years. 75% of mental health conditions occur by age 24 [66], adversely impacting future life chances and leading to long-term disability benefits [67, 68]. If SE/IPS has an effect on this trajectory over the life-course, it will strengthen the economic case further. This could be assessed by linking service participation with long-term social security and welfare data, as seen in Norway [23], to look at sustainability of outcomes, impacts on long-term earnings and need for welfare benefits.

Although we found some modelling analyses [26, 39, 40, 52] looking at the effectiveness of SE/IPS under different conditions and estimating long-run cost-effectiveness, there is scope for more modelling work. They could extrapolate short-term findings over longer-time periods making different assumptions on long-term employment. Evidence could also be adapted in models to estimate costs and benefits of SE/IPS in comparable countries where no primary economic evaluation exists.

More economic evaluations as part of naturalistic implementation studies, as well as studies determining the economic value of augmenting SE/IPS with additional measures such as psychological therapies, or looking at approaches that are more or less targeted to specific populations, would help inform policy and practice. For example, in the UK, one ongoing evaluation explores whether IPS will reach and support more people with mental health needs, if it is part of primary health services rather than linked to specialist mental health services [64]. We also found no studies directly comparing cost-effectiveness by severity of illness or dual diagnosis, or studies on high-risk populations including refugees or those with adverse childhood experiences. Comparisons between programmes that support young people to enter higher education as an alternative/complement to immediate employment are missing.

The results of qualitative research, surveys, and process evaluations on implementation and contextual issues can be incorporated into economic evaluations, for instance, to look at how well programmes engage potential service-users. Referrals to SE/IPS services may be impacted by the attitudes of people in the health and employment sectors, as well as employers [69]. Different mechanisms to recruit and retain employment specialists, as well as differences in caseloads, could be considered [40]. Another gap concerns the impact of adherence to fidelity in service delivery on the cost-effectiveness of programmes; good but less than perfect fidelity has been shown as most cost-effective for first-episode psychosis services [63]. Finally, another gap is the lack of economic studies in low-income countries, although costs of delivering SE/IPS have been examined in South Africa [70].

### Strengths and limitations

We believe our systematic review to be the most comprehensive to date, covering multiple databases in health, psychology, social science, and economics, plus additional searches of gray literature and citation snowballing. While there were no language restrictions, we did not search non-English language databases. So we may have missed literature, for instance in mainland China, where there is emerging evidence on SE/IPS services [71]. Although we assessed 24 economic evaluations as being high-quality, we may have undervalued the quality of some studies as no existing health economic appraisal checklist is a perfect fit for employment-related studies, where health is usually not the primary focus of evaluation, and studies often are cost–benefit analyses, less used by health economists [15].

Some caution in the generalisability of findings across countries is also needed. Our results cover a very heterogeneous set of studies, using many different comparators, where “usual care” is not always be clearly defined. Country-specific factors, such as generosity of welfare safety nets and employment conditions, might impact on potential cost-effectiveness, although a recent systematic review indicated IPS can be effective, regardless of the generosity of country welfare support [6].

## Conclusion

Our review indicates considerable evidence on the cost-effectiveness of IPS and other types of SE in high-income countries, particularly in Europe and North America. The economic case, while strong, is conservative; the short duration of most studies means that long-term economic benefits of being in work, accrued over the life-course, were not captured. It is time to implement SE/IPS, it is a good investment for society.

## Data Availability

All the studies included in this article are available on the quoted databases (PubMed/MEDLINE, EMBASE, PsycINFO, CINAHL, Business Source Complete, IBSS, and EconLit). The search strategies for all databases are available in Supplementary S1 and all extracted data from the studies are available in Supplementary Tables S2 and S3 as well as in Table 3.

## Supplementary material

For supplementary material accompanying this paper visit https://doi.org/10.1192/j.eurpsy.2022.2309. click here to view supplementary material
