# Supplementary material for: Economic analyses of supported employment programmes for people with mental health conditions: A systematic review
Source: Eur Psychiatry. 2022 Aug 19;65(1):e51. doi: 10.1192/j.eurpsy.2022.2309 (PMC9491084; doi:10.1192/j.eurpsy.2022.2309)
Supplement: Supplementary file 1 [file S0924933822023094sup001.docx]

**Online-Only Supplements**

**Economic analyses of supported employment programmes for people with mental health conditions: a systematic review**

[1. S1: Search Strategies in bibliographic databases 2](#_Toc107946522)

[2. S2: List of included studies 6](#_Toc107946523)

[4 Table S1. Quality assessment 10](#_Toc107946524)

[5. Table S2. Data Extraction for systematic review of IPS economic studies 18](#_Toc107946525)

[6. Table S3 Data Extraction for systematic review of supported employment economic studies 35](#_Toc107946526)

[7. List of excluded studies at full text stage 57](#_Toc107946527)

# S1: Search Strategies in bibliographic databases

**MEDLINE search strategy**

The search terms for MEDLINE are listed below. This search strategy was adapted for the other electronic databases. We explicitly did not include costing terms in most database searches as we wanted to maximise the number of papers to examine on supported employment and IPS. This was to ensure that papers without overt economic terms might also contain economic analysis.

1. exp employment, supported/
2. exp return to work/
3. “individual support”.tw.
4. ips.tw. AND employment.tw.
5. “supported employment”.tw.
6. “individual placement”.tw
7. (“return to work” OR “return-to-work” OR “work participation”).tw.
8. (“work disability” OR “work rehabilitation” OR “work retention”).tw.
9. exp disabled persons/
10. exp work/
11. exp occupations/

12. (occupation$ OR vocational$).tw.

13. OR/ 10-12

14. 9 AND 13

15. ("Job coach” OR “Employment coach” OR “Employment advis?r” OR“ Employment specialist”).tw

16 7 AND 10

17 OR /1-8

18 14 OR 16 OR 17

19. Limit 18 to 20090101 202110818

**Business Source Complete (EBSCOhost database)**

1. DE "VOCATIONAL rehabilitation" OR DE "SUPPORTED employment"
2. (DE "RETURN to work programs") OR (DE "EMPLOYMENT reentry")
3. "individual support".tw.
4. IPS.tw. AND employment.tw.
5. "SUPPORTED EMPLOYMENT".tw.
6. "INDIVIDUAL PLACEMENT".tw.
7. "Return to work".tw. OR "return-to-work" OR “work participation”
8. "WORK DISABILITY".tw. OR "WORKREHABILITATION" OR "work retention" OR "vocational rehabilitation"
9. DE "EMPLOYMENT of the mentally ill" OR DE "EMPLOYMENT of people with disabilities"
10. "Job coach” OR “Employmentcoach” .tw. OR “Employment advis?r” OR“ Employment specialist”
11. 1 OR 2 OR 3 OR 4 OR 5 OR 6 OR 8
12. DE "EMPLOYMENT of the mentally ill" OR DE "EMPLOYMENT of people with disabilities"
13. (DE "EMPLOYMENT of the mentally ill" OR DE "EMPLOYMENT of people with disabilities") AND (7 OR 8)
14. 7 OR 8
15. 9 AND 14
16. 1 OR 2 OR 3 OR 4 OR 5 OR 6 OR 10
17. 15 OR 16
18. Limit 17 to 20090101 202110818

**CINAHL**

1. (MH “Rehabilitation, Vocational+”)
2. (MH "Job Re-entry")
3. TX "individual support"
4. TX IPS AND TX employment
5. TX "SUPPORTED EMPLOYMENT"
6. TX "INDIVIDUAL PLACEMENT"
7. TX "Return to work" OR "return-to-work" OR “work participation”
8. TX "WORK DISABILITY" OR "WORK REHABILITATION" OR "work retention" OR "vocational rehabilitation"
9. MH "Employment of Disabled+") OR (MH "Disability Management") OR (MH "Employee, Disabled+")
10. (MH "Work+")
11. (MH "Occupations+")
12. TX occupation* OR vocational*
13. 10 OR 11 OR 12
14. (10 OR 11 OR 12) AND (9 AND 13)
15. TX "Job coach” OR “Employment coach” OR “Employment advis?r” OR “Employment specialist”
16. 7 AND 10
17. 1 OR 2 OR 3 OR 4 OR 5 OR 6 OR 8
18. 14 OR 16 OR 17
19. Limit 18 to 20090101 202110818

**EMBASE**

| 1 | exp supported employment/ |
| --- | --- |
| 2 | (supported adj1 employment).mp. [mp=title, abstract, heading word, drug trade name, original title, device manufacturer, drug manufacturer, device trade name, keyword, floating subheading word, candidate term word] |
| 3 | (individual adj1 placement).mp. [mp=title, abstract, heading word, drug trade name, original title, device manufacturer, drug manufacturer, device trade name, keyword, floating subheading word, candidate term word] |
| 4 | (individual adj1 support).mp. [mp=title, abstract, heading word, drug trade name, original title, device manufacturer, drug manufacturer, device trade name, keyword, floating subheading word, candidate term word] |
| 5 | (IPS and employment).mp. [mp=title, abstract, heading word, drug trade name, original title, device manufacturer, drug manufacturer, device trade name, keyword, floating subheading word, candidate term word] |
| 6 | exp Return to work/ |
| 7 | (return adj1 to adj1 work).mp. [mp=title, abstract, heading word, drug trade name, original title, device manufacturer, drug manufacturer, device trade name, keyword, floating subheading word, candidate term word] |
| 8 | (work adj1 participation).mp. [mp=title, abstract, heading word, drug trade name, original title, device manufacturer, drug manufacturer, device trade name, keyword, floating subheading word, candidate term word] |
| 9 | 6 or 7 or 8 |
| 10 | exp work/ |
| 11 | exp disabled person/ |
| 12 | (work adj1 disability).mp. [mp=title, abstract, heading word, drug trade name, original title, device manufacturer, drug manufacturer, device trade name, keyword, floating subheading word, candidate term word] |
| 13 | (work adj1 rehabilitation).mp. [mp=title, abstract, heading word, drug trade name, original title, device manufacturer, drug manufacturer, device trade name, keyword, floating subheading word, candidate term word] |
| 14 | (work adj1 retention).mp. [mp=title, abstract, heading word, drug trade name, original title, device manufacturer, drug manufacturer, device trade name, keyword, floating subheading word, candidate term word] |
| 15 | (vocational adj1 rehabilitation).mp. [mp=title, abstract, heading word, drug trade name, original title, device manufacturer, drug manufacturer, device trade name, keyword, floating subheading word, candidate term word] |
| 16 | (job adj1 coach).mp. [mp=title, abstract, heading word, drug trade name, original title, device manufacturer, drug manufacturer, device trade name, keyword, floating subheading word, candidate term word] |
| 17 | (employment adj1 coach).mp. [mp=title, abstract, heading word, drug trade name, original title, device manufacturer, drug manufacturer, device trade name, keyword, floating subheading word, candidate term word] |
| 18 | (employment adj1 advis?r).mp. [mp=title, abstract, heading word, drug trade name, original title, device manufacturer, drug manufacturer, device trade name, keyword, floating subheading word, candidate term word] |
| 19 | (employment adj1 specialist).mp. [mp=title, abstract, heading word, drug trade name, original title, device manufacturer, drug manufacturer, device trade name, keyword, floating subheading word, candidate term word] |
| 20 | 1 or 2 or 3 or 4 or 5 or 12 or 13 or 14 or 15 or 16 or 17 or 18 or 19 |
| 21 | 9 and 11 |
| 22 | 10 and 11 |
| 23 | 20 or 21 or 22 |
| 24 | limit 23 to yr="2000 - 2021" |
|  |  |

**EconLit**

1. DE "VOCATIONAL rehabilitation" OR DE "SUPPORTED employment"

2. (DE "RETURN to work programs") OR (DE "EMPLOYMENT reentry")

3. "individual support".tw.

4. IPS.tw. AND employment.tw.

5. "SUPPORTED EMPLOYMENT".tw.

6. "INDIVIDUAL PLACEMENT".tw.

7. "Return to work".tw. OR "return-to-work" OR “work participation”

8. "WORK DISABILITY".tw. OR "WORKREHABILITATION" OR "work retention" OR "vocational rehabilitation"

9. DE "EMPLOYMENT of the mentally ill" OR DE "EMPLOYMENT of people with disabilities"

10. "Job coach” OR “Employment coach” .tw. OR “Employment advis?r” OR“ Employment specialist”

11. 1 OR 2 OR 3 OR 4 OR 5 OR 6 OR 8

12. DE "EMPLOYMENT of the mentally ill" OR DE "EMPLOYMENT of people with disabilities"

13. (DE "EMPLOYMENT of the mentally ill" OR DE "EMPLOYMENT of people with disabilities") AND (7 OR 8)

14. 7 OR 8

15. 9 AND 14

16. 1 OR 2 OR 3 OR 4 OR 5 OR 6 OR 10

17. 15 OR 16

18. Limit 17 to 20090101 202110818

**IBSS (International Bibliography of the Social Sciences**

1. MAINSUBJECT.EXACT("Vocational rehabilitation")"
2. "SUPPORTED employment" (All fields)

2. "RETURN to work program* OR RETURN to work programme* (All fields)

3. “Individual Placement and Support” (All field)

4. "SUPPORTED EMPLOYMENT" (All Fields)

6. "INDIVIDUAL PLACEMENT" (All Fields)

7. "Job coach” OR “Employment coach” OR “Employment advis?r” OR“ Employment specialist” (All Fields)

8. Limit 7 to 20090101 202110818

**PsycInfo**

1. exp vocational rehabilitation/

2. supported adj1 employment).mp. [mp=title, abstract, heading word, table of contents, key concepts, original title, tests & measures, mesh]

3. (individual adj1 placement).mp. [mp=title, abstract, heading word, table of contents, key concepts, original title, tests & measures, mesh]

4. (individual adj1 support).mp. [mp=title, abstract, heading word, table of contents, key concepts, original title, tests & measures, mesh]

5. (IPS and employment).mp. [mp=title, abstract, heading word, table of contents, key concepts, original title, tests & measures, mesh]

6. exp Reemployment/

7. (return adj1 to adj1 work).mp. [mp=title, abstract, heading word, table of contents, key concepts, original title, tests & measures, mesh]

8. (work adj1 participation).mp. [mp=title, abstract, heading word, table of contents, key concepts, original title, tests & measures, mesh]

9. 6 or 7 or 8

10. exp disabilities/

11. exp employment status/

12. 10 and 11

13. (work adj1 disability).mp. [mp=title, abstract, heading word, table of contents, key concepts, original title, tests & measures, mesh]

14. (work adj1 rehabilitation).mp. [mp=title, abstract, heading word, table of contents, key concepts, original title, tests & measures, mesh]

15. (work adj1 retention).mp. [mp=title, abstract, heading word, table of contents, key concepts, original title, tests & measures, mesh]

16. (vocational adj1 rehabilitation).mp. [mp=title, abstract, heading word, table of contents, key concepts, original title, tests & measures, mesh]

17. (job adj1 coach).mp. [mp=title, abstract, heading word, table of contents, key concepts, original title, tests & measures, mesh]

18. (employment adj1 coach).mp. [mp=title, abstract, heading word, table of contents, key concepts, original title, tests & measures, mesh]

19. (employment adj1 advis?r).mp. [mp=title, abstract, heading word, table of contents, key concepts, original title, tests & measures, mesh]

20. (employment adj1 specialist).mp. [mp=title, abstract, heading word, table of contents, key concepts, original title, tests & measures, mesh]

21. 9 and 11

22. 1 or 2 or 3 or 4 or 5 or 6

23. 13 or 14 or 15 or 16 or 18 or 19 or 20

24. 12 or 21 or 22 or 23

25. limit 24 to (all journals and yr="2000 - 2021")

# S2: List of included studies

1. Booth D, Francis S, McIvor N, Hinson P, Barton B. Severe mental illness & employment: cost-benefit analysis and dynamics of decision making. Mental Health & Social Inclusion. 2014;18(4):215-23.

2. Chow CM, Croft B, Cichocki B. Evaluating the potential cost-savings of job accommodations among individuals with psychiatric disability. Journal of Vocational Rehabilitation. 2015;43(1):67-74.

3. Christensen TN, Kruse M, Hellstrom L, Eplov LF. Cost-utility and cost-effectiveness of individual placement support and cognitive remediation in people with severe mental illness: Results from a randomized clinical trial. Eur Psychiatry. 2020;64(1):e3.

4. Cimera RE. Supported employment's cost-efficiency to taxpayers: 2002 to 2007. Research & Practice for Persons with Severe Disabilities. 2009;34(2):13-20.

5. Cimera RE. The national costs of supported employment to Vocational Rehabilitation: 2002 to 2006. Journal of Vocational Rehabilitation. 2009;30(1):1-9.

6. Cimera RE. The monetary benefits and costs of hiring supported employees: A pilot study. Journal of Vocational Rehabilitation. 2009;30(2):111-9.

7. Cimera RE. National cost efficiency of supported employees with intellectual disabilities: 2002 to 2007. American Journal On Intellectual And Developmental Disabilities. 2010;115(1):19-29.

8. Cimera RE. The national cost-efficiency of supported employees with intellectual disabilities: the worker's perspective. Journal of Vocational Rehabilitation. 2010;33(2):123-31.

9. Cimera RE. Can community-based high school transition programs improve the cost-efficiency of supported employment? Career Development for Exceptional Individuals. 2010;33(1):4-12.

10. Cimera RE. Supported versus sheltered employment: Cumulative costs, hours worked, and wages earned. Journal of Vocational Rehabilitation. 2011;35(2):85-92.

11. Cimera RE. Does being in sheltered workshops improve the employment outcomes of supported employees with intellectual disabilities? Journal of Vocational Rehabilitation. 2011;35(1):21-7.

12. Cimera RE. Agency Setting as a Factor in the Effectiveness of Supported Employment Programs. Journal of Rehabilitation. 2014;80(2):41-6.

13. Cimera RE. A comparison of the cost-ineffectiveness of supported employment versus sheltered work services by state and demographics of program participants. Journal of Vocational Rehabilitation. 2016;45(3):281-94.

14. Cimera RE, Burgess S, Bedesem PL. Does Providing Transition Services by Age 14 Produce Better Vocational Outcomes for Students With Intellectual Disability? Research & Practice for Persons with Severe Disabilities. 2014;39(1):47-54.

15. Cimera RE, Wehman P, West M, Burgess S. Do sheltered workshops enhance employment outcomes for adults with autism spectrum disorder? Autism: The International Journal Of Research And Practice. 2012;16(1):87-94.

16. Dattilo J. Cost Effectiveness and Hospitalization Rates of Those with Mental Health Issues Using On-Site versus Off-Site Job Coaching. 2020.

17. Deloitte. Individual Placement and Support Programme - Economic Impact Assessment. Scottish Association for Mental Health. London: Deloitte; 2017.

18. Drake RE, Skinner JS, Bond GR, Goldman HH. Social security and mental illness: reducing disability with supported employment. Health Affairs (Project Hope). 2009;28(3):761-70.

19. Evensen S, Wisloff T, Lystad JU, Bull H, Martinsen EW, Ueland T, et al. Exploring the potential cost-effectiveness of a vocational rehabilitation program for individuals with schizophrenia in a high-income welfare society. BMC Psychiatry. 2019;19.

20. Fogelgren M, Ornstein P, Rödin M, Thoursie PS. Is Supported Employment Effective for Young Adults with Disability Pension? Evidence from a Swedish Randomized Evaluation. Journal of Human Resources. 2021:0319-10105R2.

21. Gadenne V, Maglicic M, Nolan D, Wright H, Frerichs J. Individual Placement Support: A Social Impact Bond Model. London: Behavioural Insights Team; 2021.

22. Greig R, Chapman P, Eley A, Watts R, Love B, Bourlet G. The cost effectiveness of employment support for people with disabilities. NDTi, March; 2014.

23. Hagen T. Evaluation of a Placement Coaching Program for Recipients of Disability Insurance Benefits in Switzerland. Journal of Occupational Rehabilitation. 2019;29(1):72-90.

24. Hamilton S, Kotecha-Hazzard R, Pinfold V. Project 100: Evaluation of a model of Individual Placement Support delivered through DWP employment services. London: McPin; 2016.

25. Hellström L, Kruse M, Christensen TN, Trap Wolf R, Eplov LF. Cost-effectiveness analysis of a supported employment intervention for people with mood and anxiety disorders in Denmark - the IPS-MA intervention. Nord J Psychiatry. 2021:1-8.

26. Heslin M, Howard L, Leese M, McCrone P, Rice C, Jarrett M, et al. Randomized controlled trial of supported employment in England: 2 year follow-up of the Supported Work and Needs (SWAN) study. World Psychiatry: Official Journal Of The World Psychiatric Association (WPA). 2011;10(2):132-7.

27. Hoffmann H, Jäckel D, Glauser S, Mueser KT, Kupper Z. Long-term effectiveness of supported employment: 5-year follow-up of a randomized controlled trial. The American Journal Of Psychiatry. 2014;171(11):1183-90.

28. Holmås TH, Monstad K, Reme SE. Regular employment for people with mental illness - An evaluation of the individual placement and support programme. Soc Sci Med. 2021;270:113691.

29. Howard LM, Heslin M, Leese M, McCrone P, Rice C, Jarrett M, et al. Supported employment: randomised controlled trial. The British Journal Of Psychiatry: The Journal Of Mental Science. 2010;196(5):404-11.

30. Indecon. Evaluation of EmployAbility (Supported Employment) Service. Dublin: Department of Social Protection; 2016.

31. Khalifa N, Talbot E, Barber S, Schneider J, Bird Y, Attfield J, et al. A Feasibility Cluster Randomized Controlled Trial of Individual Placement and Support (IPS) for Patients With Offending Histories. Front Psychiatry. 2019;10:952.

32. Kilsby M, Beyer S. A Financial Cost:Benefit Analysis of Kent Supported Employment: A Framework for Analysis. Canterbury: Kent County Council; 2011.

33. Knapp M, Patel A, Curran C, Latimer E, Catty J, Becker T, et al. Supported employment: cost-effectiveness across six European sites. World Psychiatry: Official Journal Of The World Psychiatric Association (WPA). 2013;12(1):60-8.

34. Lockett H, Elwin W, Haines N, Conneely J. Employment as a health intervention - A good return on investment. Australian and New Zealand Journal of Psychiatry. 2012;46(SUPPL. 1):46-7.

35. Mavranezouli I, Megnin-Viggars O, Cheema N, Howlin P, Baron-Cohen S, Pilling S. The cost-effectiveness of supported employment for adults with autism in the United Kingdom. Autism: The International Journal Of Research And Practice. 2014;18(8):975-84.

36. Mental Health R. Steps into Work: Integrating Employment and Mental Health Supports Project Final Report. Dublin: Mental Health Reform; 2018.

37. Parlettaa VA, Waghorn G. The financial viability of evidence-based supported employment for people with mental illnesses in a blended funding system. Journal of Vocational Rehabilitation. 2016;44(2):227-41.

38. Parsonage M. Commissioning what works. The economic and financial case for supported employment. London: Sainsbury Centre for Mental Health; 2009.

39. Perkins R, Farmer P, Litchfield P. Realising ambitions: Better employment support for people with a mental health condition. London: Department for Work and Pensions; 2009.

40. Reme SE, Grasdal AL, Løvvik C, Lie SA, Øverland S. Work-focused cognitive-behavioural therapy and individual job support to increase work participation in common mental disorders: a randomised controlled multicentre trial. Occupational And Environmental Medicine. 2015;72(10):745-52.

41. Rosenheck R, Leslie D, Sint K, Lin H, Robinson DG, Schooler NR, et al. Cost-Effectiveness of Comprehensive, Integrated Care for First Episode Psychosis in the NIMH RAISE Early Treatment Program. Schizophr Bull. 2016;42(4):896-906.

42. Saha S, Bejerholm U, Gerdtham U-G, Jarl J. Cost-effectiveness of supported employment adapted for people with affective disorders. Nordic Journal Of Psychiatry. 2018;72(3):236-9.

43. Sambo T. Economic evaluation in the context of schizophrenia and related psychotic conditions: A systematic review and cost-consequence analysis. 2020.

44. Schneider J, Akhtar A, Boycott N, Guo B, Latimer E, Cao Z, et al. Individual placement and support versus individual placement and support enhanced with work-focused cognitive behaviour therapy: Feasibility study for a randomised controlled trial. British Journal of Occupational Therapy. 2016;79(5):257-69.

45. Schneider J, Boyce M, Johnson R, Secker J, Slade J, Grove B, et al. Impact of supported employment on service costs and income of people with mental health needs. Journal of Mental Health. 2009;18(6):533-42.

46. Shi Y. Cost‐effectiveness of the individual placement and support model of supported employment for people with severe mental illness: Results from a Canadian randomized trial. 2011.

47. *Stant A, Van Busschbach J, Vugt M, Michon H. A rehabilitation intervention to help people with severe mental illness obtain and keep a paid job: The economic evaluation. Value in Health. 2012;15(7):A337.

48. *Stant AD, Van Busschbach JT, Van Vugt M, Michon H. A rehabilitation intervention to help people with severe mental illness obtain and keep a paid job: The economic evaluation. Journal of Mental Health Policy and Economics. 2013;16(SUPPL. 1):S32.

49. Stroupe KT, Jordan N, Richman J, Bond GR, Pogoda TK, Cao L, et al. Cost-Effectiveness of Individual Placement and Support Compared to Transitional Work Program for Veterans with Post-traumatic Stress Disorder. Adm Policy Ment Health. 2021.

50. Sultan-Taïeb H, Villotti P, Berbiche D, Dewa CS, Desjardins É, Fraccaroli F, et al. Can social firms contribute to alleviating the economic burden of psychiatric disabilities for the public healthcare system? Health & Social Care In The Community. 2019;27(5):1311-20.

51. Szplit K. Social Return on Investment (SROI) Forecast Analysis. Individual Placement and Support (IPS) for Period April 2010 to March 2011. Remploy; 2013.

52. Tholén SL, Hultkrantz L, Persson M. Economic Evaluation of Supported-Employment Inspired Program for Pupils With Intellectual Disabilities. Nordic Journal of Working Life Studies. 2017;7(1):69-86.

53. van Busschbach JT, Michon H, van Vugt M, Stant A, Aerts-Roorda MmvLC, van Erp N. Effectiviteit van Individuele Plaatsing en Steun in Nederland. Eindverslag van een gerandomiseerde gecontroleerde effectstudie Deel 2: Bevindingen na 30 maanden follow-up. Utrecht: Trimbos-instituut; 2011.

54. van Stolk C, Hofman J, Hafner M, Janta B. Psychological Wellbeing and Work. Improving Service Provision and Outcomes. Cambridge: RAND Europe; 2014.

55. Washington State Institute for Public P. Individual Placement and Support (IPS) for individuals with serious mental illness. Benefit-cost results. Seattle: Washington State Institute for Public Policy; 2019.

56. Whitworth A. The economic case for well-considered investment in health-related employment support: Costs and savings of alternative modified Individual and Placement Support (IPS) models. Disability And Health Journal. 2018;11(4):568-75.

57. Yamaguchi S, Sato S, Horio N, Yoshida K, Shimodaira M, Taneda A, et al. Cost-effectiveness of cognitive remediation and supported employment for people with mental illness: a randomized controlled trial. Psychological Medicine. 2017;47(1):53-65.

*** Same abstract**

# Table S1. Quality assessment

**eTable1 (a): Quality appraisal using modified CHEERS checklist**

| **Section/topic** | **#** | **Booth (2014)^1^** | **Chow (2015)^2^** | **Christensen (2020)^3^** | **Cimera (2009)^4^** | **Cimera (2009)^5^** | **Cimera (2009) ^6^** | **Cimera (2010)^7^** |
| --- | --- | --- | --- | --- | --- | --- | --- | --- |
| **TITLE AND ABSTRACT** | |  |  |  |  |  |  |  |
| Title | 1 | Y | N | Y | Y | N | Y | Y |
| Abstract | 2 | N | N | Y | Y | N | N | N |
| **INTRODUCTION** | |  |  |  |  |  |  |  |
| Background and objectives | 3a | Y | Y | Y | Y | Y | Y | Y |
|  | 3b | Y | Y | Y | Y | Y | Y | Y |
| **METHODS** | |  |  |  |  |  |  |  |
| Target population and subgroups | 4 | N | Y | Y | Y | Y | Y | Y |
| Setting and location | 5 | Y | Y | Y | Y | Y | Y | Y |
| Study perspective | 6 | N | Y | Y | Y | Y | Y | Y |
| Comparators | 7 | N | Y | Y | N | N | N | Y |
| Time horizon | 8 | Y | Y | Y | Y | N | N | Y |
| Discount rate | 9 | N | N | Y | N | N | N | N |
| Choice of health outcomes | 10a | N | N | Y | N | N | N | N |
| Choice of other outcomes | 10b | Y | Y | Y | Y | Y | Y | Y |
| Measurement of effectiveness | 11a | N | N | Y | N | N | N | Y |
|  | 11b | N | N | N | Y | Y | N | N |
| Measurement and valuation of preference based outcomes | 12 | N | N | N | N | N | N | N |
| Estimating resources and costs | 13a | N | N | Y | Y | N | Y | Y |
|  | 13b | N | N | N | N | N | N | N |
| Currency, price date, and conversion | 14 | N | N | Y | Y | N | N | Y |
| Choice of model | 15 | N | N | N | N | N | N | N |
| Assumptions | 16 | N | N | N | N | N | N | N |
| Analytical models | 17 | N | N | N | N | N | N | N |
| **RESULTS** | |  |  |  |  |  |  |  |
| Study parameters | 18 | N | N | Y | N | N | N | N |
| Incremental costs and outcomes | 19 | Y | N | Y | N | N | Y | N |
| Characterising uncertainty | 20a | N | N | Y | N | N | N | N |
|  | 20b | N | N | N | N | N | N | N |
| Characterising heterogeneity | 21 | N | N | Y | Y | N | N | N |
| **Section/topic** |  |  |  |  |  |  |  |  |
| **DISCUSSION** | |  |  |  |  |  |  |  |
| Study findings, limitations, generalisability, and current knowledge | 22 | Y | Y | Y | Y | Y | Y | Y |
| **Other** | |  |  |  |  |  |  |  |
| Source of funding | 23 | N | N | Y | N | N | N | N |
| Conflicts of interest | 24 | N | N | Y | N | N | N | N |

**eTable1 (b): Quality appraisal using modified CHEERS checklist**

| **Section/topic** | **#** | **Cimera (2010)^8^** | **Cimera (2010) ^9^** | **Cimera (2011)^10^** | **Cimera (2011)^11^** | **Cimera (2014)^12^** | **Cimera (2016)^13^** | **Cimera (2014)^14^** |
| --- | --- | --- | --- | --- | --- | --- | --- | --- |
| **TITLE AND ABSTRACT** | |  |  |  |  |  |  |  |
| Title | 1 | Y | Y | Y | N | N | Y | N |
| Abstract | 2 | N | N | Y | Y | N | N | N |
| **INTRODUCTION** | |  |  |  |  |  |  |  |
| Background and objectives | 3a | Y | Y | Y | N | Y | Y | Y |
|  | 3b | Y | Y | Y | Y | Y | Y | Y |
| **METHODS** | |  |  |  |  |  |  |  |
| Target population and subgroups | 4 | Y | Y | N | Y | Y | Y | Y |
| Setting and location | 5 | Y | Y | Y | Y | Y | Y | Y |
| Study perspective | 6 | Y | Y | Y | N | N | Y | N |
| Comparators | 7 | N | Y | Y | Y | Y | Y | Y |
| Time horizon | 8 | Y | Y | N | N | N | N | N |
| Discount rate | 9 | N | N | N | N | N | N | N |
| Choice of health outcomes | 10a | N | N | N | N | N | N | N |
| Choice of other outcomes | 10b | Y | Y | Y | Y | Y | Y | Y |
| Measurement of effectiveness | 11a | N | N | Y | Y | N | Y | Y |
|  | 11b | Y | N | N | N | N | N | N |
| Measurement and valuation of preference based outcomes | 12 | N | N | N | N | N | N | N |
| Estimating resources and costs | 13a | N | Y | Y | Y | N | Y | Y |
|  | 13b | N | N | N | N | N | N | N |
| Currency, price date, and conversion | 14 | N | N | Y | N | N | Y | N |
| Choice of model | 15 | N | N | N | N | N | N | N |
| Assumptions | 16 | N | N | N | N | N | N | N |
| Analytical models | 17 | N | N | N | N | N | N | N |
| **RESULTS** | |  |  |  |  |  |  |  |
| Study parameters | 18 | N | N | N | N | N | N | N |
| Incremental costs and outcomes | 19 | N | Y | Y | Y | N | N | Y |
| Characterising uncertainty | 20a | N | N | N | N | N | N | N |
|  | 20b | N | N | N | N | N | N | N |
| Characterising heterogeneity | 21 | Y | Y | N | N | N | Y | N |
| **Section/topic** |  |  |  |  |  |  |  |  |
| **DISCUSSION** | |  |  |  |  |  |  |  |
| Study findings, limitations, generalisability, and current knowledge | 22 | Y | Y | Y | Y | Y | Y | Y |
| **Other** | |  |  |  |  |  |  |  |
| Source of funding | 23 | N | N | N | N | N | N | N |
| Conflicts of interest | 24 | N | N | N | N | N | Y | N |

**eTable1 (c): Quality appraisal using modified CHEERS checklist**

| **Section/topic** | **#** | **Cimera (2012) ^15^** | **Dattilo (2020) ^16^** | **Deloitte (2017) ^17^** | **Drake (2009)^18^** | **Evensen (2019)^19^** | **Fogelgren (2021)^20^** | **Gadenne (2020)^21^** |
| --- | --- | --- | --- | --- | --- | --- | --- | --- |
| **TITLE AND ABSTRACT** | |  |  |  |  |  |  |  |
| Title | 1 | N | Y | Y | N | Y | N | N |
| Abstract | 2 | Y | N | Y | N | Y | N | N |
| **INTRODUCTION** | |  |  |  |  |  |  |  |
| Background and objectives | 3a | Y | Y | Y | Y | Y | Y | Y |
|  | 3b | Y | Y | Y | Y | Y | Y | Y |
| **METHODS** | |  |  |  |  |  |  |  |
| Target population and subgroups | 4 | Y | N | N | Y | Y | Y | N |
| Setting and location | 5 | Y | Y | Y | Y | Y | Y | Y |
| Study perspective | 6 | Y | Y | Y |  | Y | Y | N |
| Comparators | 7 | Y | Y | Y | Y | Y | Y | N |
| Time horizon | 8 | N | Y | Y | Y | Y | Y | N |
| Discount rate | 9 | N | N | N |  | N | N | N |
| Choice of health outcomes | 10a | N | Y | N | Y | Y | N | N |
| Choice of other outcomes | 10b | Y | Y | Y | N | Y | Y | Y |
| Measurement of effectiveness | 11a | Y | Y | N | N | Y | Y | Y |
|  | 11b | N | N | N | N | N | N | N |
| Measurement and valuation of preference based outcomes | 12 | N | N | N | N | N | N | N |
| Estimating resources and costs | 13a | Y | Y | N | N | N | Y | Y |
|  | 13b | N | N | Y | N | N | N | N |
| Currency, price date, and conversion | 14 | N | N | N | N | Y | N | N |
| Choice of model | 15 | N | N | N | N | N | N | N |
| Assumptions | 16 | N | N | Y | N | N | N | N |
| Analytical models | 17 | N | N | N | N | N | N | N |
| **RESULTS** | |  |  |  |  |  |  |  |
| Study parameters | 18 | N | N | N | N | N | N | N |
| Incremental costs and outcomes | 19 | Y | Y | Y | N | Y | Y | Y |
| Characterising uncertainty | 20a | N | N | N | N | Y | N | N |
|  | 20b | N | N | Y | N | N | N | N |
| Characterising heterogeneity | 21 | N | N | Y | N | N | N | Y |
| **Section/topic** |  |  |  |  |  |  |  |  |
| **DISCUSSION** | |  |  |  |  |  |  |  |
| Study findings, limitations, generalisability, and current knowledge | 22 | Y | Y | Y | Y | Y | Y | Y |
| **Other** | |  |  |  |  |  |  |  |
| Source of funding | 23 | N | N | Y | Y | Y | Y | Y |
| Conflicts of interest | 24 | N | N | N | Y | Y | N | N |

**eTable1 (d): Quality appraisal using modified CHEERS checklist**

| **Section/topic** | **#** | **Greig (2014)^22^** | **Hagen (2019)^23^** | **Hamilton (2016)^24^** | **Hellstrom (2021)^25^** | **Heslin (2011)^26^** | **Hoffmann (2014)^27^** | **Holmas (2021)^28^** |
| --- | --- | --- | --- | --- | --- | --- | --- | --- |
| **TITLE AND ABSTRACT** | |  |  |  |  |  |  |  |
| Title | 1 | Y | N | N | Y | N | N | N |
| Abstract | 2 | N | Y | Y | Y | Y | Y | N |
| **INTRODUCTION** | |  |  |  |  |  |  |  |
| Background and objectives | 3a | Y | Y | Y | Y | Y | Y | Y |
|  | 3b | Y | Y | Y | Y | Y | Y | Y |
| **METHODS** | |  |  |  |  |  |  |  |
| Target population and subgroups | 4 | N | Y | Y | Y | Y | Y | Y |
| Setting and location | 5 | Y | Y | Y | Y | Y | Y | Y |
| Study perspective | 6 | N | Y | N | Y | N | N | Y |
| Comparators | 7 | Y | Y | Y | Y | Y | Y | Y |
| Time horizon | 8 | Y | Y | Y | Y | Y | Y | Y |
| Discount rate | 9 | N | Y | N | N | N | N | Y |
| Choice of health outcomes | 10a | N | N | N | Y | N | N | N |
| Choice of other outcomes | 10b | Y | Y | Y | N | Y | Y | Y |
| Measurement of effectiveness | 11a | N | N | Y | Y | Y | Y | Y |
|  | 11b | N | N | N | N | N | N | N |
| Measurement and valuation of preference based outcomes | 12 | N | N | N | N | N | N | N |
| Estimating resources and costs | 13a | N | Y | N | Y | Y | N | Y |
|  | 13b | N | N | N | N | N | N | N |
| Currency, price date, and conversion | 14 | N | N | N | Y | N | N | Y |
| Choice of model | 15 | N | N | N | N | N | N | N |
| Assumptions | 16 | N | Y | N | N | N | N | N |
| Analytical models | 17 | N | N | N | N | N | N | N |
| **RESULTS** | |  |  |  |  |  |  |  |
| Study parameters | 18 | N | N | N | N | N | N | N |
| Incremental costs and outcomes | 19 | N | N | N | Y | N | N | N |
| Characterising uncertainty | 20a | N | N | N | Y | Y | N | N |
|  | 20b | N | Y | N | N | N | N | N |
| Characterising heterogeneity | 21 | N | Y | N | N | N | N | N |
| **Section/topic** |  |  |  |  |  |  |  |  |
| **DISCUSSION** | |  |  |  |  |  |  |  |
| Study findings, limitations, generalisability, and current knowledge | 22 | Y | Y | Y | Y | Y | Y | Y |
| **Other** | |  |  |  |  |  |  |  |
| Source of funding | 23 | Y | N | Y | Y | Y | Y | Y |
| Conflicts of interest | 24 | N | Y | N | Y | N | Y | Y |

**eTable1 (e): Quality appraisal using modified CHEERS checklist**

| **Section/topic** | **#** | **Howard (2010)^29^** | **Indecon (2016)^30^** | **Khalifa**  **(2019)^31^** | **Kilsby**  **(2011)^32^** | **Knapp**  **(2013)^33^** | **Lockett**  **(2012)^34^** | **Mavranezouli**  **(2014)^35^** |
| --- | --- | --- | --- | --- | --- | --- | --- | --- |
| **TITLE AND ABSTRACT** | |  |  |  |  |  |  |  |
| Title | 1 | N | N | N | N | Y | N | Y |
| Abstract | 2 | Y | Y | N | N | Y | Y | Y |
| **INTRODUCTION** | |  |  |  |  |  |  |  |
| Background and objectives | 3a | Y | Y | Y | Y | Y | Y | Y |
|  | 3b | Y | Y | Y | Y | Y | Y | Y |
| **METHODS** | |  |  |  |  |  |  |  |
| Target population and subgroups | 4 | Y | Y | Y | Y | Y | N | N |
| Setting and location | 5 | Y | Y | Y | Y | Y | Y | Y |
| Study perspective | 6 | N | Y | Y | Y | Y | Y | Y |
| Comparators | 7 | Y | Y | Y | N | Y | N | Y |
| Time horizon | 8 | Y | N | Y | Y | Y | N | Y |
| Discount rate | 9 | N | N | N | N | Y | N | Y |
| Choice of health outcomes | 10a | N | N | Y | N | Y | Y | Y |
| Choice of other outcomes | 10b | Y | Y | Y | Y | Y | Y | Y |
| Measurement of effectiveness | 11a | Y | Y | Y | N | Y | Y | N |
|  | 11b | N | N | N | N | N | N | Y |
| Measurement and valuation of preference based outcomes | 12 | N | N | N | N | N | N | N |
| Estimating resources and costs | 13a | Y | Y | Y | N | Y | N | N |
|  | 13b | N | N | N | Y | N | N | Y |
| Currency, price date, and conversion | 14 | N | N | N | Y | N | N | Y |
| Choice of model | 15 | N | N | N | N | N | N | Y |
| Assumptions | 16 | N | N | N | N | N | N | Y |
| Analytical models | 17 | Y | N | N | N | Y | N | Y |
| **RESULTS** | |  |  |  |  |  |  |  |
| Study parameters | 18 | N | N | Y | N | Y | N | Y |
| Incremental costs and outcomes | 19 | Y | Y | Y | N | Y | N | Y |
| Characterising uncertainty | 20a | N | N | N | N | Y | N | N |
|  | 20b | N | N | N | N | N | N | Y |
| Characterising heterogeneity | 21 | N | N | N | N | Y | N | N |
| **Section/topic** |  |  |  |  |  |  |  |  |
| **DISCUSSION** | |  |  |  |  |  |  |  |
| Study findings, limitations, generalisability, and current knowledge | 22 | N | Y | Y | Y | Y | Y | Y |
| **Other** | |  |  |  |  |  |  |  |
| Source of funding | 23 | Y | Y | Y | Y | Y | N | Y |
| Conflicts of interest | 24 | N | Y | Y | N | N | N | Y |

**eTable1 (f): Quality appraisal using modified CHEERS checklist**

| **Section/topic** | **#** | **MentalHealthReform**  **(2018)^36^** | **Parlettaa**  **(2016)^37^** | **Parsonage**  **(2009)^38^** | **Perkins**  **(2009)^39^** | **Reme**  **(2015)^40^** | **Rosenheck**  **(2016)^41^** | **Saha**  **(2018)^42^** |
| --- | --- | --- | --- | --- | --- | --- | --- | --- |
| **TITLE AND ABSTRACT** | |  |  |  |  |  |  |  |
| Title | 1 | N | N | N | N | N | Y | Y |
| Abstract | 2 | N | Y | N | N | Y | Y | Y |
| **INTRODUCTION** | |  |  |  |  |  |  |  |
| Background and objectives | 3a | Y | Y | Y | Y | Y | Y | Y |
|  | 3b | Y | Y | Y | Y | Y | Y | Y |
| **METHODS** | |  |  |  |  |  |  |  |
| Target population and subgroups | 4 | Y | N | N | N | Y | Y | N |
| Setting and location | 5 | Y | Y | Y | Y | Y | Y | Y |
| Study perspective | 6 | Y | Y | Y | Y | Y | Y | Y |
| Comparators | 7 | N | Y | N | Y | Y | Y | Y |
| Time horizon | 8 | Y | Y | N | N | Y | Y | N |
| Discount rate | 9 | N | N | N | N | Y | N | N |
| Choice of health outcomes | 10a | Y | N | N | N | Y | Y | Y |
| Choice of other outcomes | 10b | Y | Y | Y | Y | Y | N | Y |
| Measurement of effectiveness | 11a | Y | Y | Y | N | Y | Y | Y |
|  | 11b | N | N | N | Y | N | N | N |
| Measurement and valuation of preference based outcomes | 12 | N | N | N | N | N | N | N |
| Estimating resources and costs | 13a | Y | N | Y | N | Y | Y | Y |
|  | 13b | N | N | N | Y | N | N | N |
| Currency, price date, and conversion | 14 | N | N | N | Y | Y | Y | N |
| Choice of model | 15 | N | N | N | N | N | N | N |
| Assumptions | 16 | Y | Y | N | N | N | Y | Y |
| Analytical models | 17 | N | N | N | N | N | Y | N |
| **RESULTS** | |  |  |  |  |  |  |  |
| Study parameters | 18 | Y | N | N | Y | N | N | Y |
| Incremental costs and outcomes | 19 | N | N | N | N | Y | Y | Y |
| Characterising uncertainty | 20a | N | N | Y | N | Y | Y | Y |
|  | 20b | N | N | N | Y | N | N | N |
| Characterising heterogeneity | 21 | N | N | N | N | Y | Y | N |
| **Section/topic** |  |  |  |  |  |  |  |  |
| **DISCUSSION** | |  |  |  |  |  |  |  |
| Study findings, limitations, generalisability, and current knowledge | 22 | Y | Y | Y | Y | Y | Y | Y |
| **Other** | |  |  |  |  |  |  |  |
| Source of funding | 23 | Y | Y | Y | Y | Y | Y | Y |
| Conflicts of interest | 24 | N | N | N | N | Y | N | N |

**eTable1 (g): Quality appraisal using modified CHEERS checklist**

| **Section/topic** | **#** | **Sambo**  **(2020)^43^** | **Schneider**  **(2016)^44^** | **Schneider**  **(2009)^45^** | **Shi**  **(2011)^46^** | **Stant**  **(2013)^47^** | **Stroupe**  **(2021)^49^** | **Sultan-Taïeb**  **(2019)^50^** |
| --- | --- | --- | --- | --- | --- | --- | --- | --- |
| **TITLE AND ABSTRACT** | |  |  |  |  |  |  |  |
| Title | 1 | Y | N | N | Y | Y | Y | N |
| Abstract | 2 | N | Y | N | Y | Y | Y | N |
| **INTRODUCTION** | |  |  |  |  |  |  |  |
| Background and objectives | 3a | Y | Y | Y | Y | Y | Y | Y |
|  | 3b | Y | Y | Y | Y | Y | Y | Y |
| **METHODS** | |  |  |  |  |  |  |  |
| Target population and subgroups | 4 | Y | Y | Y | Y | N | Y | Y |
| Setting and location | 5 | Y | Y | Y | Y | Y | Y | Y |
| Study perspective | 6 | Y | Y | Y | Y | Y | Y | Y |
| Comparators | 7 | Y | Y | Y | Y | Y | Y | Y |
| Time horizon | 8 | Y | Y | Y | Y | Y | Y | N |
| Discount rate | 9 | Y | N | N | N | N | N | N |
| Choice of health outcomes | 10a | Y | Y | Y | Y | Y | N | Y |
| Choice of other outcomes | 10b | Y | Y | Y | Y | Y | Y | N |
| Measurement of effectiveness | 11a | Y | Y | Y | Y | Y | Y | Y |
|  | 11b | N | N | N | N | N | N | N |
| Measurement and valuation of preference based outcomes | 12 | N | N | N | N | N | N | N |
| Estimating resources and costs | 13a | Y | Y | Y | Y | Y | Y | Y |
|  | 13b | N | N | N | N | N | N | N |
| Currency, price date, and conversion | 14 | Y | Y | N | Y | N | Y | Y |
| Choice of model | 15 | N | N | N | N | N | N | N |
| Assumptions | 16 | Y | N | N | N | N | N | N |
| Analytical models | 17 | Y | N | N | N | N | N | Y |
| **RESULTS** | |  |  |  |  |  |  |  |
| Study parameters | 18 | N | N | N | Y | N | Y | Y |
| Incremental costs and outcomes | 19 | Y | N | Y | Y | Y | Y | N |
| Characterising uncertainty | 20a | Y | Y | N | Y | Y | Y | Y |
|  | 20b | N | N | N | N | N | N | N |
| Characterising heterogeneity | 21 | Y | N | Y | Y | N | N | Y |
| **Section/topic** |  |  |  |  |  |  |  |  |
| **DISCUSSION** | |  |  |  |  |  |  |  |
| Study findings, limitations, generalisability, and current knowledge | 22 | Y | Y | Y | Y | Y | Y | Y |
| **Other** | |  |  |  |  |  |  |  |
| Source of funding | 23 | Y | Y | Y | Y | Y | Y | Y |
| Conflicts of interest | 24 | N | Y | Y | N | N | Y |  |

**eTable1 (h): Quality appraisal using modified CHEERS checklist**

| **Section/topic** | **#** | **Szplit**  **(2013)^51^** | **Tholen**  **(2017)^52^** | **Van Busschbach**  **(2011)^53^** | **van Stolk**  **(2014)^54^** | **WSIPP**  **(2019)^55^** | **Whitworth**  **(2018)^56^** | **Yamaguchi**  **(2017) ^57^** |
| --- | --- | --- | --- | --- | --- | --- | --- | --- |
| **TITLE AND ABSTRACT** | |  |  |  |  |  |  |  |
| Title | 1 | Y | Y | N | N | N | Y | Y |
| Abstract | 2 | Y | Y | Y | N | N | N | Y |
| **INTRODUCTION** | |  |  |  |  |  |  |  |
| Background and objectives | 3a | Y | Y | Y | Y | Y | Y | Y |
|  | 3b | Y | Y | Y | Y | Y | Y | Y |
| **METHODS** | |  |  |  |  |  |  |  |
| Target population and subgroups | 4 | Y | Y | Y | N | N | N | Y |
| Setting and location | 5 | Y | Y | Y | Y | Y | Y | Y |
| Study perspective | 6 | Y | Y | Y | Y | Y | Y | Y |
| Comparators | 7 | Y | Y | Y | N | Y | Y | Y |
| Time horizon | 8 | Y | Y | Y | Y | Y | Y | Y |
| Discount rate | 9 | Y | Y | Y | N | Y | N | N |
| Choice of health outcomes | 10a | N | N | Y | Y | Y | N | Y |
| Choice of other outcomes | 10b | Y | Y | Y | Y | Y | Y | Y |
| Measurement of effectiveness | 11a | N | Y | Y | N | N | N | Y |
|  | 11b | N | N | N | Y | Y | Y | N |
| Measurement and valuation of preference based outcomes | 12 | N | N | N | N | N | N | N |
| Estimating resources and costs | 13a | N | N | Y | N | N | N | Y |
|  | 13b | N | Y | N | Y | Y | Y | N |
| Currency, price date, and conversion | 14 | N | Y | Y | Y | N | N | Y |
| Choice of model | 15 | N | N | N | N |  | N | N |
| Assumptions | 16 | N | N | N | Y | Y | N | N |
| Analytical models | 17 | N | N | N | N | N | N | N |
| **RESULTS** | |  |  |  |  |  |  |  |
| Study parameters | 18 | N | N | Y | Y | Y | Y | Y |
| Incremental costs and outcomes | 19 | Y | N | Y | N | Y | N | Y |
| Characterising uncertainty | 20a | N | N | Y | N | N | N | Y |
|  | 20b | N | Y | N | Y | Y | Y | N |
| Characterising heterogeneity | 21 | N | N | N | Y | Y | Y | N |
| **Section/topic** |  |  |  |  |  |  |  |  |
| **DISCUSSION** | |  |  |  |  |  |  |  |
| Study findings, limitations, generalisability, and current knowledge | 22 | Y | Y | Y | Y | Y | Y | Y |
| **Other** | |  |  |  |  |  |  |  |
| Source of funding | 23 | N | Y | Y | Y | N | Y | Y |
| Conflicts of interest | 24 | N | N | N | N | N | Y | Y |

# Table S2. Data Extraction for systematic review of IPS economic studies

| **Author**  **(year)**  **/country** | **Setting and study population**  **(age, sex, size)** | **Intervention details (study design, description of intervention, comparator, and type of intervention)** | **Economic analysis** | | **Outcomes and key findings** | | | **Quality Score** |
| --- | --- | --- | --- | --- | --- | --- | --- | --- |
|  |  |  | **Perspective**  **Price Year**  **Currency** | **Type of economic analysis** | **Effect on mental health** | **Work-related outcome** | **Economic or financial outcome** |  |
| (Booth et al 2014)  UK | 262 individuals with severe mental illness who were referred to IPS in several sites in the north of England. Demographic data were not reported.  Data were collected in 2010 and 2011. | Modelling study for IPS using literature review, qualitative interviews and national/local databases. Comparator services assumed to be sheltered employment, training colleges, supported employment programmes and a programme of grants, information and support (Access to Work) . Duration 1 year. | Public Purse  2012  UK pounds | CBA | Not reported | During 2010/2011, 79 (30%) of IPS clients obtained paid employment. 7 (3%) in employment of 4 hours or less, 16 (6%) for 5-16 hours, 56 (21%) for 16 hours or more. Marginal employment rate compared to no action estimated as 73%. | The individual service cost is estimated to be around £3,800. Estimated to be a positive return on investment to public purse of £1·04 for every £ spent. | 35% |
| (Christensen et al 2020)  Denmark | 482 people with severe mental illness: schizophrenia, schizotypal, or delusional disorders(F20–F29) 75·7% or 77·8% or bipolar disorder (F31) 13·2% or 10·5%, or recurrent depression (F33) 11·1% or 11·7% according to ICD 10 in both IPS and SAU groups. Mean age 33 in both IPS and SAU groups. 61·3% and 60·3% male in IPS and SAU groups respectively. Data collected between 2012 and 2018. | RCT comparing IPS (n=243) compared with service as usual (SAU) (n=239). Duration 1·5 years | Societal  2016  Euros | CEA, CUA | QALY gains were non-significantly higher in the intervention group than the control group (0·0329 vs. 0·0074, p=0·2960).  Mental health hospital care costs were lower in the IPS group €14,549 versus €18,279. (p=0·0901).  No significant difference in somatic hospital, primary health care or prescription costs. | IPS participants earned an average of €1,792 more than the control group.  Productivity gains in the intervention -€7214 vs. -€5422, p=0·205).  Labour market intervention costs were significantly lower in the IPS group. €403 versus €3,395 (p<0·0001) | IPS was less costly, with non- significantly improved QALY gains compared to SAU. Overall costs, including productivity losses were significantly lower by a mean of €9,543 n the IPS group (P=0·001).  If there was a societal threshold of €35,000 for willingness-to-pay for a QALY, there is a probability of 95·6.% of IPS being cost-effective compared to SAU. | 95% |
| (Deloitte 2017)  UK | 126 adults with unspecified mental illness using IPS services in Glasgow, Scotland. Age unspecified | Modelling study drawing on published literature, IPS data and expert opinion. The intervention was IPS and the comparator traditional vocational schemes (TVS). Duration: 1 year. | Health, Public Purse and Service Users Combined  2016  UK pounds | CBA | 40%-60% reductions in Community Psychiatric Nurse (CPN) appointments and 3 less psychiatric appointments after 1 year after having secured employment.  Total costs avoided of £96,710. NHS costs avoided per service user with more than 3 months competitive employment £1,981. | Total service user benefit due to increased earnings £84,020. | The annual cost of the IPS service was £116,000. Benefits of IPS: increased earnings £84,020, health care costs avoided £96,710, welfare benefits avoided £59,210. Net benefits £123,940. Additional net costs of TVS avoided £57,030.  Total net benefit £180,970.  Net Benefits per user if employment sustained for less than or more than 3 months: £2,300 and £7,870 | 70% |
| (Gadenne et al 2021)  UK | 1926 individuals aged 18-65 (mean age=39, 41% to 49% female) with severe and enduring mental health conditions. Data collected between March 2016 and September 2019. | Impact evaluation of delivery of IPS in six sites in England. Funding on basis of Social Impact Bond model where payment depends on outcomes achieved. No comparator group. Duration: 6 months. | Providers  2011-12  UK pounds | CEA | Not reported | 31% of participants engaged (n=590) achieved a job start, 22% maintaining work for at least 6 weeks, 75% working 16 hours or more per week. | Three of four IPS sites where data were available made net losses, i.e. their costs were greater than their contract income.  Cost per job start achieved £4,400 (range £3,600 to £5,300) | 43% |
| (Hamilton et al 2016)  UK | 63 people with bipolar disorder, schizophrenia, or psychosis. Male (63%), female (35%), transgender (2%). Mean age 39, 38% White,  Data collected between March 2015 until the end of July 2016. | Mixed methods analysis using routine data and qualitative interviews comparing IPS modified to be delivered through public employment services (Job Centre Plus). No comparator group. Duration: 1 year | Public Purse  Price year not stated  UK pounds | CEA | Not reported | 11 participants gained employment during pilot study (17·2%). During the pilot overall 18 jobs were obtained, with some participants having more than one job during this period. Mean increase in pre-tax income for these individuals was £3528 (range: £510 -£11,266) | Staff costs for the project during the final year were £57,533. From a public purse perspective the total cost after reduction in disability benefits was £40,567, with a net cost per person in employment of £3,688. Individuals would need to be employed for over two years to generate net savings. | 48% |
| Hellström et al (2021)  Denmark | 326 people aged 18 – 60 with an anxiety or affective disorder recruited from mental health centres and private psychiatrists in Copenhagen. Gender not reported. Participants should not have been in contact with mental health services for more than 3 years. | The intervention was IPS intervention modified for people with mood and anxiety disorders (IPS-MA) (162 people) compared to services as usual (SAU) (164 people). These could be social services (e.g. group therapy or psycho-social support interventions) or labour market services. Duration of study two years but only 12 month outcomes used in economic analysis. | Societal  2016  Euros | CUA, CEA | When imputed cases data included mean QALYs gained were 0·056 and -0·17 in the IPS-MA and SAU groups (p<0·05). The difference was not significant for complete cases only.  1 year mental health service use in the IPS-MA group was €5,489 compared to €8,161 in the SAU group (p=0·078) Overall health care costs were not significantly different between the two groups. | Mean wage earnings in the IPS-MA group were significantly lower, €5034 versus €8410 (p=0·017)  Labour market service costs were significantly lower in the IPS-MA group, €1,329 versus €5,591 (p=0·009). | IPS-MA had a mean cost per person per year of €1,183. Overall, there was no significant difference in costs between the two groups, although costs were lower in the IPS-MA group €5,485 versus €7,706. P=0·423.  There was between an 83% and 95% chance at €30,000 per QALY gained of IPS-MA being cost effective versus SAU. If cost per hour of worked gained were used instead the intervention would not be cost effective with significantly lower levels of hours worked than SAU. | 78% |
| (Heslin et al 2011)  UK | 219 individuals, with severe mental illness recruited between November 2004 and September 2006. Mean age 38, 66% to 69% male, 41% and 34% white, in IPS and control groups. 69% and 76% had a psychotic disorder. 31% and 24% had a mood disorder. | RCT comparing IPS (n=109) with treatment as usual/ local vocational services (TAU) (n=110). Duration: 2 years. | Health care  2006/7  UK pounds | CEA | There were no differences between intervention and control groups at follow-up on any clinical measures.  Over 24 months, health care costs in intervention group were lower than controls (£9,571 vs £11,932), p-value not reported | Intervention had a significantly higher proportion in competitive employment than control group (22% vs. 11%, p=0·041). | With lower costs and higher outcomes IPS was dominant. In probabilistic sensitivity analysis there was a 90% chance of IPS being the most cost effective option. | 56% |
| (Hoffmann et al 2014)  Switzerland | 100 individuals aged 18-64 with severe mental illness including schizophrenia spectrum, affective disorder), male (65%). Mean age 33·5 and 34·1 in intervention & support groups. | RCT comparing  IPS (n= 46) with traditional vocational rehabilitation (TVR) (n=54). Duration: 5 years. | Society  Price year not stated  Swiss Francs (CHF) | ROI | Intervention group had significantly less hospitalisation (21% versus 46·7% p=0·015),  fewer psychiatric hospital admissions (0·4 versus 1·1 p=0·026) and spent fewer days in the hospital (38·6 versus 96·8 p=0·027). | Intervention group had higher rates of competitive work than traditional vocational rehabilitation (65% vs 33%, p=0·002), worked more hours per year (689 vs. 392, p=0·023), earned more wages (CHF 11,826 vs CHF 6,885, p=0·004), had longer job tenures (104·8 vs. 35·5, p<0·001) | Earning per client over 5 years 66,977 versus 37,093 in IPS and TVR. Mental health treatment costs per client CHF 25,484 versus 40,093. Vocational programme costs CHF 80,917 and CHF 43,701.  Social ROI: 132·2% | 52% |
| (Holmås et al 2021)  Norway | 327 individuals (mean age=35) with moderate (depression and anxiety disorders) to severe mental illness (psychotic or bipolar dis-order with or without comorbid substance abuse/dependency), women (50%), from regional primary and secondary mental health care settings for 43 months | Original study based on RCT comparing  IPS (n= 184) with treatment as usual (n=143) 327 people before and after the IPS intervention. Duration: 3·6 years | Societal  2016  Norwegian kroner | CBA | Not reported | During 43 months, the intervention group had 8·8% higher rates of regular employment than in the control group, and 5% higher in regular employment with a half-time job or more (16·5% vs 10·7%). | Net social benefit: NOR 217 ( gain in productivity=65+cost-savings from traditional VR programme costs=211- programme cost(100)+ cost-savings from excess burden of taxes 41, so 65+211-100+41=217. | 65% |
| (Howard et al 2010)  UK | 150 individuals (mean age=38) with psychotic or chronic affective disorder in South London | RCT comparing  IPS (n=109) with local traditional vocational service, TAU (n=110). Duration: 1 year | Health care | CEA | Psychiatric inpatient costs were lower in the intervention group (£ 719 vs £2241), also lower costs for community mental health nurse costs than the control group (£49 vs. £65) | There were no significant differences between the treatment as usual and intervention groups in obtaining competitive employment (13% in the intervention group and 7% in controls; p= 0·15), nor in secondary outcomes. | CS: Total costs were £2176 significantly higher in the control group (bootstrapped 95% CI £445–4168)., No significant differences in outcomes.  During the 1-year follow-up, two-thirds of the intervention group received input from employment workers at mean cost of £296. There were no substantial differences in the number of people using other services. However, control group participants who were admitted spent substantially more days in hospital than inpatients in the intervention group. This resulted in a difference in in-patient costs of £1522. Total costs were £2176 significantly higher in the control group (bootstrapped 95% CI £445–£4168). | 56% |
| (Khalifa et al 2019)  UK | 18 individuals (mean age=39·2) with schizophrenia, depression, personality disorder, with offending histories in community forensic settings over 12 months, male (88·9%) | RCT comparing  IPS (n=11) with Treatment as usual (n=7). Duration: 1 year | Health care  2016  UK pounds | CEA | Brief Psychiatric Rating Scale scores were higher in the intervention group (34 vs 25·5, p-value not reported), SF-12 in mental health were higher (53·1 vs 43·8), EQ-5D (64·3 vs 70), p-values not reported. Health care costs not reported separately from cost of IPS. | Intervention group had higher rates in open employment at 12 months (9·1% and 0%) than TAU | IPS less costly than TAU. £: 1,799 vs.1,940 at 12 months. | 70% |
| (Knapp et al 2013)  Netherlands, UK, Italy, Bulgaria, Switzerland, Germany | 312 individuals with SMI (schizophrenia and schizophrenia-like disorders, bipolar disorder, or depression with psychotic features, using ICD-10 criteria for 18 months in six European  cities: Groningen (Netherlands), London (UK), Rimini  (Italy), Sofia (Bulgaria), Ulm-Gunzburg (Germany), and  Zurich (Switzerland | RCT comparing  IPS (n=156) with standard vocational services (n=156). ). Duration :1·5 years. | Health and Social Care  Societal  2003  UK pounds | CBA, CEA | Readmission rates were lower in IPS than the control group (13% vs 20%).  Mean health care costs in the IPS group at first six month follow up were significantly lower than for control £4,688 versus £6,926 (Mean difference £2,720 95% CI -£4,624, -£813. Costs were lower in the following two six month follow ups but the difference was not significant. | Over 18 months, IPS had higher rates of being at least 1 day in employment (55% vs. 28% ) than those in vocational services. | Mean costs for IPS across all sites were £18,877 versus £25,455 in controls (Mean difference £7,880 95% CI -£12,249, -£3151. Costs were significantly lower in 3 of the six sites: London, Ulm and Zurich and the intervention was dominant, with lower costs and better outcomes in all areas except Groningen. In Groningen the additional cost per additional 1% of people working at least 1 day was £30 and additional cost per additional day worked was £10.  Boostrapped mean net monetary benefits comparing the costs of intervention with value of employment achieved for both IPS and control were favourable at £17,005 in the IPS group. | 91% |
| (Lockett 2012)  New Zealand | 1400 individuals with severe mental illness | Retrospective analysis of data for those receiving IPS, control group not reported. Duration: 4 years. | Health care  Welfare sector  Price year not reported  New Zealand dollars | CBA | Every $1NZD spent from Health is returned in full within year one through reduced contact time with clinical teams and reduced hospital admissions. | Not reported | For every $1 spent by Welfare, $1·11 is returned in year one alone from taxes paid and reduced welfare payments. | 39% |
| (Mavranezouli et al)  UK | Model drew on previous study of people with formal diagnosis of autism and IQ>= 70. Mean IQ score 98·8 (Wechsler Adult Intelligence Scale) | Markov modelling study comparing IPS versus standard care (day services). Duration: 8 years. | Health care  2012  UK pounds | CEA, CUA | Mean QALYs gained over 8 years were 5·42 in the IPS group and 5·31 in the control group.  Secondary analysis including other health and social care costs, including mental health-care costs, other primary and secondary care costs and local authority costs revealed mean health care costs of £16,005 and £16,663 in the IPS and control groups | Over 8 years mean weeks in employment were 136 and 102 for the IPS and control groups. | For the primary analysis, just including the costs of IPS and day care, the cost per QALY gained was £5,600 and cost per extra week of employment was £18. In probabilistic sensitivity analysis there was a 67% and 75·2% chance of being cost effective at £20,000 or £30,000 per QALY gained.  In secondary analysis including health and social care costs, the IPS intervention was dominant with lower costs and better outcomes. In probabilistic sensitivity analysis there was an 80% chance of being cost effective at £30,000 per QALY gained. | 92% |
| (Mental Health Reform 2018)  Ireland | 95 adults with severe and enduring mental health problems, not in paid employment who received IPS services from 2015 to 2017 | A pilot IPS study, control group not reported. Duration: 2 years | Provider  Price year not reported  Euros | CEA | Not reported | In the project, 36% had at least one job placement, The average number of hours worked per week by successful applicants was 21 hours, the average weekly wage for successful participants was €230.  The combined staff and project costs totalled €276,326, with a cost per participant of €2,909. | Cost per job outcome was €8,374. If start-up costs and project management excluded the cost per participant was €2,451 and the cost per job outcome was €7,057. | 61% |
| (Parlettaa 2016)  Australia | 175 individuals aged 15-64 (47% male) with schizophrenia or bipolar affective disorder, major depression, anxiety disorders, Posttraumatic Stress Disorder, personality disorder and substance abuse disorder. | Observational cohort study comparing IPS (n=68) with pre-IPS cohort (n=107). Duration: 1·5 years | Provider and public purse | CBA | Not reported | Intervention group had significantly higher rates of job starts than pre-IPS services (67·6% vs 56·1%) (Significance not reported). | Net revenues were higher in IPS than pre-IPS groups. The IPS enhanced service achieved higher gross revenue per participant ($9,062) than pre-IPS services ($7,514). The IPS enhanced pro-gramme generated more net revenue (gross revenue less direct costs) per participant compared to pre-IPS services ($6,929 vs. $6,161) | 52% |
| (Parsonage 2009)  UK | 330 individuals with (unspecified) mental health problems | Observational study for IPS participants. Support for 330 people per year including 165 new referrals. Duration: 1 year | Public purse  2003  UK pounds | CCA | Assumed rehospitalisation rates for IPS lower than control group (20% vs. 31%) drawing on literature. Estimated saving of £6,000 per client in inpatient costs over 18 months, based on the average cost of psychiatric inpatient care in England (figures not stated). | 100 out of 187 clients supported by the Mid-Surrey Employment Advice Service achieved paid employment outcomes (53·5%). A further 78 clients were supported into unpaid employment, training and education. Among those finding employment, 67% worked for 16 or more hours a week. | The total cost of service £280,000 a year. Nationally at the level of provision recommended in government commissioning guidance on vocational services estimated at £67 million a year (or £440,000 per average PCT or £2,000 per client). Current spending on day and employment services is around £184 million a year. IPS could divert resources from less effective services. | 43% |
| (Perkins & Rinaldi 2009)  UK | Hypothetical 135,000 new IPS participants each year with unspecified mental health problems. | Modelling study comparing  IPS with Traditional service or no intervention. Duration: 2 years. | Public Purse  Price year not stated  UK pounds | ROI | Not reported | Unpublished survey data for study involving employment workers across private, public and voluntary sectors was used to assume all clients would receive support for 6 months, with 35% continuing for one year and 25% for two years. | Total cost of the programme £180 million per annum. 27,000 jobs would need to be created for the service to break even. This would mean a cost per job before fiscal benefits of £6,600; if 47,000 jobs were created the return on investment would be 1·72. | 56% |
| (Rosenheck et al 2016)  USA | 404 individuals aged 15-40 with First Episode Psychosis, less than 6 months on lifetime antipsychotics in clinical treatment clinics. Demographic information not provided. | RCT comparing IPS: Navigate (NAV), a comprehensive, multidisciplinary, team-based treatment approach for first episode psychosis, including IPS (n=223) with community care (CC) (n=181). Duration: 2 years | Health care  US dollars | CBA, CUA | The NAV group had significantly greater improvement in PANSS total scores and improved significantly more on the QLS as a one standard deviation change on the Quality of Life Scale (QLS-SD) (p<0·02). There was no significant difference in overall costs between the two groups. However, the intervention group had higher outpatient mental health ($1870 vs 1379, p=0·05), antipsychotic medication costs ($1739 vs 1060. P=0·01).  Costs for all mental health and medical surgical inpatient care were lower in the intervention group but no significant difference ($3694 vs $3780, p=0·91) | Not reported | The incremental cost-effectiveness ratio was $12 081/QLS-SD, with a 94 % that NAV was more cost-effective than CC at $40 000/QLS-SD. When converted to monetized Quality Adjusted Life Years, NAV benefits exceeded costs, especially at future generic drug prices. | 87% |
| (Saha et al 2018)  Sweden | 55 individuals with unemployed, depressive episodes, recurrent depression or bipolar disorder. Demographic information not reported. | RCT comparing IES (individual enabling and support) (an IPS intervention) vs. TVR (traditional vocational rehabilitation).  Duration: 1 year | Societal  2014  Euros | CEA, CUA | Intervention group was more effective using Manchester Short Assessment of Quality of Life (MANSA), but not EQ-5D. There were no significant differences in QALY improvement between groups. But quality of life measured by the MANSA scale significantly improved over the study period in IE. Health care costs were not included. | The value of productivity gains were higher in the intervention group (€6059) than the control group (€111), p-value not reported. | The cost of IES was €7247 lower per person per year, compared to TVR. TVR unit costs taken from the Knapp et al study^13^ The total cost for IES were €528 per person per year compared to €7775 for TVR. Intervention was dominant with no change in quality of life but lower costs. | 74% |
| (Sambo 2020)  Canada | 109 individuals aged 18-30 (mean age=23) with Schizophrenia, psychosis, bipolar disorder, male (45%). | RCT comparing  IPS: IPS+Early intervention psychosis (TAU) (n=56) with TAU (n=53). Duration: 1 year | Health care  Public payer  2016  Canadian dollars | CEA, CUA | Although the sample was small due to data collection issues, the EQ-5D-5L index scores were consistently higher for those in the TAU group compared with those in the IPS+ group. Scores on the QLS were consistently higher for the IPS+ TAU group compared with those in the TAU group. Overall health care costs, including primary care as well as specialist mental health care were non-significantly higher in the TAU group $3,884 vs $3,656. | At 12 months, proportion employed (IPS+ TAU vs. TAU):60% versus 50% but not significant. Non significantly increased working days in intervention group: mean 8·38 more days, 57·24 vs 48·86 days. | Total costs per patient in the IPS+TAU group were lower those in TAU (mean difference $228, p = 0·823, 95% CI, $-2,261 to $1,806). Also improvements in employment outcomes in IPS group and quality of life but not significant. | 91% |
| (Shi 2011)  Canada | 149 individuals aged 18-64 (mean age=40 or 41, female 37·3% and 39·2% in IPS or control groups 40·6) with severe mental illness: psychosis,  bipolar disorder, major depression; and using outpatient psychiatric hospital services between 2001-2004. | RCT comparing IPS (n=75) with usual vocational services, including sheltered workshops, creative workshops, a  consumer-run boutique, horticultural programs, job-finding-skills training and psychosocial intervention (n=74) Duration of study :1·5 years. | Health Care, Public Purse, and Societal  2010-11  Canadian dollars | CEA | No significant differences in mental health care costs between the two groups: Inpatient costs ($1421 vs. $6443, p=0·2258), other mental health service ($639 vs. $1286, p=0·9032), out-of-pocket costs of psychologist services ($11 vs $10, p=0·9921) | Over 12 months, significantly longer hours in competitive employment in IPS than the control group. Mean 126 hours vs. 72 hours on average, p=0·0004), higher wages in competitive employment ($935 vs $514, p=0·004) | Overall costs were lower for IPS compared to controls from all three perspectives: health and social care perspective: $25,709 ( IPS) vs $26,683 (UC) (P=0·011). Public Purse: $32,984 vs. $33,945, Society: $27,014 vs. $27,678. The IPS program was less expensive while improving outcomes, which means IPS dominates usual services. | 87% |
| (Stant et al 2013) and  (vanBusschbach et al 2011)  Netherlands | 151 with severe and long-term mental disorders who want to work. In IPS and control groups respectively: 55% and 64% psychosis, 17% and 10% mood disorders, 22% and 23% personality disorders, mean age 34·1 and 35·6, male 73% and 75%. | RCT comparing IPS n=(71) vs. regular vocational rehabilitation (n=80).  Duration of study: 2·5 years | Health care  Societal  2008  Euros | CEA, CUA | There were no differences between groups in quality of life at any time point measured using the MANSA – Manchester Short Assessment of Quality of Life. Mental health and general health care cost were higher but significance not reported. Overall mean costs including health, net of productivity gains were €57,285 and €43,819 in the IPS and control groups. | After 2·5 years significantly more people in the IPS group were in regular paid work with paid work during the study was significantly higher in the IPS group (44% versus 25%) P<0·05. More hours were also worked in the IPS group. Mean rehabilitation costs, including the cost of intervention were greater in the IPS group €1,705 versus €1,176. | IPS has higher costs and better outcomes than regular vocational rehabilitation. The cost per additional 1 percent of individuals in paid work was €1, 084. However, averted social welfare costs due to increased work participation are not included in the cost effectiveness ratio. here is an 80% probability of being cost effective if society is willing to pay €2,000 per additional 1% in employment. The incremental cost per additional 1 point on the MANSA scale is €76,359. | 83% |
| (Stroupe et al 2021)  USA | 541 military veterans (mean age= 41·2) with PTSD, men (81·7%) | RCT comparing IPS (n=271) with transitional work (TW) programmes (n=270).  Duration: 1.5 years | Health care  Societal  2019  US dollars | CEA, ROI | Mental health costs were insignificantly higher for IPS than TW ($1687 vs $1498, p=0·75)  The annual mean cost per person of outpatient were $3970 higher for IPS compared to TW ($23,245 vs. $19,276, p= 0·004).  Overall mean health care costs including costs of vocational rehabilitation were significantly higher in the IPS group $29,691 vs $23,298 | The average number of hours worked in competitive employment per person per year was significantly higher in the IPS than the TW (632 hours vs 458 hours, p= 0·002). The mean annual income from competitive employment was higher in the IPS than the TW ($9762 vs $7326, p= 0·02) | IPS is more costly, more effective.  95% probability that IPS is cost-effective if willing to pay is $81 per additional hour worked.  The average total costs per person per year were similar between groups ($29,828 vs. $26,772, p= 0·17). The incremental cost-effectiveness was $28 per additional hour of competitive employment.  The return on investment (excluding TW income) was 32·9% for IPS ($9762 mean income/$29,691 mean  total costs) and 29·6% for TW ($7326 mean income/$24,781 mean total costs). | 87% |
| (Szplit 2013)  UK | 45 individuals with moderate to severe mental health problems in collaboration with community mental health teams from April 2010 to March 2011 | 1 year observational study, with longer term impacts modelled. Duration: 5 years. | Employment  20110/11  UK pounds | CBA, ROI | Not reported | For 1 year, 40% (18 out of 45) of participants secured permanent employment | For every £1 invested with IPS there would be a return ranging between £5·01 and £6·77 in social added value.  The total present value (PV) of IPS for 2010/2011 is valued at £526,885. The total investment is £77,822 Total present value less total investment figure (NPV) is £449,063. | 57% |
| (van Stolk et al 2014)  UK | People with depression, anxiety (common mental disorders) including employment, but also some people on sick leave | Modelling study comparing vocational support based on the Individual Placement and Support (IPS) model in IAPT or other suitable psychological therapy services. Duration: 1 year | Healthcare  Public purse  2011  UK pounds | CBA | Assumes IPS service would support 120 clients per year. 35 would have reduced healthcare utilisation costs of £300 per year, including savings from fewer GP visits and limited use of secondary care. | 24.5 people would stop claiming job seekers allowance £3,900 each. | Positive cost benefit ratio = 1·41 from IPS; note this assumes that savings from reduced statutory sick leave so cost benefit ratio for long term unemployed alone not stated. | 70% |
| (Washington State Institute for Public Policy 2019)  USA | Those with severe mental illness | Modelling study (Monte Carlo Simulation analysis for risk/uncertainty analysis)  IPS vs. traditional vocational rehabilitation. Duration: 50 years. | Taxpayers,  Participants | CBA | Net health care costs for psychiatric hospitalisation reduced by $8 per participant. | Not reported. Net program cost per participant: $849. Additional taxes from additional earnings to taxpayers $2,090 and to participants $4,910 | Total positive benefits net of deadweight costs: $5,741. Benefit cost benefit ratio=$7·7. Chance the program will produce Benefits greater than costs 80%. | 74% |
| (Whitworth 2018)  UK | Hypothetical 5000 IPS programme starts over 30 months. Time limited to maximum 15 months. Assumed to have mental and physical health problems | Modelling study for IPS comparing alternative modified scenarios, the control group not reported. Duration: 10 years. | Public purse  Price year not reported  UK pounds | CBA | Impacts on health care costs not included in analysis | Not detailed but costs averted include welfare benefits avoided, including council tax benefit and universal credit. Average annual earnings from employment assumed to be £11,800 | ROI=0·32 to 7·47, depending on models at 10 years. ROI at 5 years ranges from 0.19 to 4.53 depending on model scenario. | 70% |

# Table S3 Data Extraction for systematic review of supported employment economic studies

| **Author**  **(year)**  **/country** | **Setting and study population**  **(age, sex, size)**  **Dates of study** | **Intervention details (study design & duration, description of intervention, comparator, and type of intervention)** | **Economic analysis** | | **Outcomes and key findings** | | | **Quality Score** |
| --- | --- | --- | --- | --- | --- | --- | --- | --- |
|  |  |  | **Perspective**  **Price Year**  **Currency** | **Type of economic analysis** | **Effect on mental health** | **Work-related outcome** | **Economic or financial outcome** |  |
| (Chow et al 2015)  USA | 987 community dwelling adults with severe mental illness recruited from outpatient psychiatric clinics across 8 US states who were in receipt of Supplemental Security Income (SSI). Mean age 40·76 Female 44·9%; includes 62·9% with diagnosis of schizophrenia. Data collected between February 1996 and May 2000. | Secondary econometric analysis of data from a RCT comparing supported employment programmes (including some IPS) to usual vocational rehabilitations services. Focus in this analysis on job accommodation versus no job accommodation in the trial. Duration: 2 years | Welfare system  2000  US Dollars | Cost Offset | Not Reported | Individuals with job accommodations worked additional mean 7·87 hours per month (p≤0·001) but earned a mean $0·56 less per hour (p≤0·001). | Marginal monthly SSI costs averted were $11·73 higher with job accommodations (p≤0·05). Potential SSI savings for individuals with accommodations 68% higher than those without accommodations (p≤0·01). | 39% |
| (Cimera 2016)  USA | 40,118 supported employees in the United States whose cases were closed in 2013. 60·4% were male, 70·6% were White. Primary impairments: learning disabilities 51%, mental disorders 36%. Age not reported. | Analysis of electronic records in the US Rehabilitation Services Administration 911 database. The intervention was supported employment services. There was no comparison group but costs of service use were compared for those for sheltered workshop users. Duration: not stated. | Public purse  2013  US Dollars | CEA | Not reported | 53% of services users were in employment and had their cases closed. The mean cost per month of supported employment was $342·07 (SD $418·45). The mean cost per month for sheltered workshops reported in literature was $728·58. 12·8% of supported employees had mean costs per month in excess of mean sheltered workshop costs. | Mean cost to vocational rehabilitation services per hour worked was $7·23 (SD $15·46). The mean cost reported in literature to sheltered workshops per hour worked was $9·94. 20·4% of supported employees had mean costs per hour worked in excess of mean sheltered workshop costs per hour worked. There was substantial variation in costs per hour worked across vocational rehabilitation agencies. | 61% |
| (Cimera et al 2014)  USA | 62 people receiving supported employment services. 77·4% had learning difficulties. Age and gender not reported. | Analysis of data from matched cohorts of individuals from supported employment services that also have sheltered workshops compared with supported employment services that do not also provide sheltered workshops. Duration: not stated but at least 11 months. | Public purse  Price year not stated  US Dollars | CCA | Not reported | Supported employees in services that also provide sheltered workshops (SW group) provided a mean 10·84 months of support per individual compared to 8·32 months per individual for the supported employment services who do not also offer sheltered workshops (NSW group). This difference was not significant. | Mean cost per month of service received was $399·43 in the SW group compared to $684·38 in the NSW group (p=0·050) | 30% |
| (Cimera et al 2014)  USA | 15,040 young adults with learning disabilities receiving vocational rehabilitation services who previously had a requirement for transition services to be addressed in individualised educational plans at age 14 or age 16. The mean age was 20·3 and 56·5% were male. 32·7% and 35·1% had multiple disabilities in the age 14 or 16 groups. 52·1% and 53·7% were White in the two groups respectively. Data were taken from records from 2006 to 2009 | Analysis of electronic records in the US Rehabilitation Services Administration 911 database. Matched groups of young adult vocational rehabilitation service users with differential prior timing of transition plans in individualised educational plans (either age 14 or age 16) who subsequently received vocational rehabilitation services. Duration: 4 years. | Public purse  Price year not stated  US Dollars | CCA | Not reported | Early transition was associated with higher levels of employment 58·8% versus 45·6%. For each year from 2006 to 2009 inclusive differences in employment rates were significant (P<0.001). There was no significant difference in hours worked or wages earned. Annual costs of supported employment services did not vary significantly between the early and later transition groups ($3,542 versus $3,750). | Individuals who received transition services by age 14 were significantly more likely to be employed after they enter vocational rehabilitation programmes than individuals receiving transition services by 16 (p<0.001). There were no differences in service costs. | 43% |
| (Cimera et al 2012)  USA | 215 supported employees with autistic spectrum disorders who participated in sheltered workshops (SW) prior to becoming supported employees and 215 supported employees with autistic spectrum disorders who did not participate in sheltered workshops (NSW) prior to becoming supported employees. Mean age of the SW and NSW groups was 31·12 and 37·75; 80% in both groups were male. 78·5% and 83·3% of the SW and NSW groups were White. 74·8% in both groups had a secondary disability. Data were taken from records from 2002 to 2006 | Analysis of electronic records in the US Rehabilitation Services Administration 911 database. The intervention was supported employment, with or without prior sheltered workshop experience. Duration: 5 years | Public purse  Price year not reported  US Dollars | CEA | Not reported | 45·6% of the former SW group were employed when their cases were closed compared with 39·5% of the NSW group workers. The SW group worked a mean 23·5 hours (SD = 11·4) per week; compared with 25·0 (SD = 12·3) hours in the NSW group. Neither difference was significant. Former SW who became competitively employed earned a mean $129·36 (SD $89·66) per week, compared with $191·42 (SD = US$118·83) (p = .001) for NSWs. Former SWs had mean service costs of $6065·08 (SD 9879·33) per person. Costs for the NSW were significantly lower at $2440·60; SD $4585·63) (p <.001). Mean service costs for the employed in the NSW group were also significantly lower than those for the employed in the SW group: $4212·24 (SD $5088·11) compared to $8364·39 (SD $11,420·70) (p = .001). | Individuals from the NSW group had better outcomes – weekly earnings, while service costs were significantly lower than the group with prior SW used. | 53% |
| (Cimera 2011)  USA | 112 individuals who were in supported employment and/or sheltered work programmes. Age and gender not reported. Data from service use between January 2000 and June 2008. Disability breakdown not provided. | Cohorts drawn from dataset for all sheltered and supported employees in a US State. 20 participants were in both supported and sheltered employment program. 92 were in matched pairs in either sheltered work or supported employment. Duration:8.5 years | Public purse  2008  US Dollars | CEA | Not reported | In the matched pair analysis the SE cohort received services for 46 months (SD 26·71) compared to 70·02 (SD 31·28) months in the SW group (P<0·001). SE worked fewer hours per month than the SW group - 60·55 (SD 16·18) hours versus 65·41 (SD 34·65) hours but this was not significant. The cost per month of services received was $496·41.(SD $399·13 in the SE group versus $602·36 ($327·98) in the SW group. This difference was not significant. The SE group had significantly higher monthly wages of $403·34 versus $159·77 in the SW group (p<0·001). Including data from the 20 individuals who participated in both programmes did not change findings. | For the matched pairs the SE cohort had a non- significantly lower cost per hour worked of $10·83 (SD $15·35) compared with $14·13 (SD $14·46) The cost per dollar earned for the SE group was $2·01 (SD $4·33) versus $12·24 (SD $20·03) (p <0·001)for SW employees. Including data from the 20 individuals who participated in both programmes did not change findings. | 56% |
| (Cimera 2011)  USA | 9808 supported employees with intellectual disabilities who participated in sheltered workshops (SW) prior to becoming supported employees and 4,904 supported employees with intellectual disabilities who did not participate in sheltered workshops (NSW) prior to becoming supported employees. Mean age of the SW and NSW groups was 38·93 and 31·56; 58·3% in both groups were male. 78·3% and 71·5% of the SW and NSW groups were White. 49·6% in both groups had a secondary disability. Data were taken from records from 2002 to 2006 | Matched cohorts drawn from electronic records in the US Rehabilitation Services Administration 911 database. The intervention was supported employment, with or without prior sheltered workshop experience. Duration: 5 years. | Public purse  Price year not stated  US Dollars | CEA | Not reported | Supported employees from the NSW group were just as likely to be employed as supported employees from the SW group (60·4% versus 59·6%). NSW group employees earned $137·20 (SD $82·29) per week compared to $118·55 (SD $74·56) per week in the SW group. (P<0·001). They also worked more hours (24·78 (SD 10·06) vs 22·44 (SD 10·71) p=0·006. The NSW group also cost less in services ($4,542·65 SD $6,141·63 versus $7,894·63 (SD $11,643·03) (p<0·001). Similarly for the cost of services (employed sample only) NSW group $5,399·26 SD $5,847·08 compared to $8,659·44 SD $10,895·56) in the SW group .(p<0·001) | The NSW group worked longer hours, earned more per week and had lower supported employment costs than the SW group. | 43% |
| (Cimera 2010)  USA | 104,213 individuals with intellectual disabilities funded by state vocational rehabilitation agencies throughout the entire United States and its territories from 2002 to 2007 who wished to be enrolled in supported employment. 56·9% were male. Mean age was 33·89, 71·8% were white and 47·5% had a secondary diagnosis. Data were taken from records between 2002 and 2007 inclusive. | Analysis of electronic records in the US Rehabilitation Services Administration 911 database. The intervention was Supported employment services received through state vocational rehabilitation agencies. There was no comparator, but it was assumed that individuals who did not obtain competitive employment would receive sheltered employment. Duration: 1 year | Service User  2008  US Dollars | CBA | Not Reported | 64,692 individuals (62·0%) were successfully employed at time their cases were closed. Mean hours worked per week were 21·80. 64% of job seekers without secondary conditions and 59·2% of job seekers with secondary conditions were successfully employed. | Employees had a mean monthly net benefit of $475·35 and a mean benefit cost ratio of 4·20. Benefit-cost ratios, ranged from 3·85 in 2002 to 4·45 in 2004. Net mean monthly benefit was $454·51, benefit cost ratio of 4·07 for service users with secondary conditions. Without secondary conditions mean net monthly benefit was $489·83 with a benefit cost ratio of 4·27. Rates of successful employment varied from 97·33% in Washington to 37·14% in Oklahoma. Mean benefit cost ratios across US states ranged from 13·54 in Washington State to 1·86 in Wisconsin. | 48% |
| (Cimera 2010)  USA | 104,213 individuals with intellectual disabilities funded by state vocational rehabilitation agencies throughout the entire United States and its territories from 2002 to 2007 who wished to be enrolled in supported employment. 56·9% were male. Mean age was 33·89, 71·8% were white and 47·5% had a secondary diagnosis. Data were taken from records between 2002 and 2007 inclusive. | Analysis of electronic records in the US Rehabilitation Services Administration 911 database. The intervention was Supported employment services received through state vocational rehabilitation agencies. There was no comparator, but it was assumed that individuals who did not obtain competitive employment would receive sheltered employment. Duration: 1 year | Public Purse  2008  US Dollars | CBA | Not reported | For all service users the mean gross monthly costs of providing supported employment were $636·45, while gross net monthly benefits to the taxpayer were $769·54. Impacts on employment not reported. | Net monthly benefits were $133·10, with a benefit-cost ratio of 1·21. Service users with or without secondary conditions had similar net benefits of $113·03 and $128·24 with benefit : cost ratios of 1·19 and 1·23 respectively. Benefit-cost ratios across US states vary greatly between 2·77 in Nebraska and Illinois in 0·63. | 56% |
| (Cimera 2010)  USA | 246 supported employees who completed at least one job cycle (i.e., they obtained and eventually separated from a job in the community). 185 received no transition services when they were in high school. 31 individuals received special education including transition planning only. 30 received community-based transition services in high school. Mean age of no transition, school-transition and community-transition groups was 36·24, 25·59 and 23·86. Males accounted for 50·3%, 61·3% and 63·3% respectively. Primary disability: mental health 32·4%, 12·9% and 6·7% respectively; mild learning difficulties 47·6%, 45·2% and 36·7%, moderate learning disabilities 6·5%, 25·8% and 36·7%, severe learning disabilities 10% in community transition group only. 48·7%, 35·7% and 37·9% respectively had a secondary diagnosis. Study dates not provided. | Analysis of cohort of supported employees who had either : (a) received no transition services in high school, (b) had community-based work experiences in high school , and (c) individuals who had individualised education programs (IEPs) in high school but experienced only in-school transition services. It was assumed that if an individual was not in supported employment they would have been in sheltered workshops. Secondary analysis matched pairs of individuals to compare no transition versus community transition and no transition versus school transition. Duration: not stated. | Public Purse  2008  US Dollars | CBA | Not reported | No transition services generated mean per capita gross monthly benefit to taxpayers of $619·4 and mean per capita gross monthly cost of $1,345·02. School transition service group had mean per capita gross monthly benefit of $551·27 and gross monthly cost of $979·02. Community-based transition services group had $686·10 in gross benefits and $940·95 in gross costs.  For 21 matched pairs in the no transition versus community transition groups, months employed were 3·24 and 7·32 p=0·001 respectively. For 17 matched pairs in school transition versus community transition months employed were 4·70 and 8·10 (p=0·0006). | Individuals in the no transition, school transition and community transition groups had benefit-cost ratio of 0·46, 0·56 and 0·73 respectively.  For matched pairs in the no transition versus community transition group the respective benefit-cost ratios were 0·41 and 0·61. In 85·7% of cases, individuals with community-based transition services were more cost-efficient.  For matched pairs in the school transition versus community transition group the respective benefit-cost ratios were 0·37 and 0·59. In 88·2% of cases, individuals with community-based transition services were more cost-efficient. | 56% |
| (Cimera 2009)  USA | All 231,204 supported employees from 2002 to 2007 who were served by vocational rehabilitation (VR) throughout the entire United States and its territories. 57·2% Male, 74·1% White, Mean Age 32·2. Primary condition: 40·3% learning disabilities, 29·6% mental illness.48·7% had secondary condition. Data were taken from records between 2002 and 2007 inclusive. | Analysis of electronic records in the US Rehabilitation Services Administration 911 database. The intervention was Supported employment services received through state vocational rehabilitation agencies. There was no comparator, but it was assumed that individuals who did not obtain competitive employment would receive sheltered employment. Duration: 1 year | Public purse  2008  US Dollars | CBA | Not Reported | For all service users the mean gross monthly costs of providing supported employment were $544·31, while gross net monthly benefits to the taxpayer were $795·65. Net benefits were $251·34. For mental health service users the mean gross monthly costs of providing supported employment were $481·76, while gross net monthly benefits to the taxpayer were $807·69. Net benefits were $325·92. For learning disability service users mean gross monthly costs of providing supported employment were $651·47, while gross net monthly benefits to the taxpayer were $781·21. Net benefits were $129·74. Impacts on employment not reported. | From a taxpayer perspective for all service users the benefit : cost ratio was 1·46. For mental health and learning disability service users it was 1·68 and 1·20 respectively. For mental health and learning disability service users with or without secondary conditions benefit cost ratios were1·67 or 1·69 and 1·17 and 1·22 respectively. | 65% |
| (Cimera 2009)  USA | All 192,756 supported employees from 2002 to 2006 who were served by vocational rehabilitation (VR) throughout the entire United States and its territories. Males ranged from 56·7% to 57·4%; White 72·4% to 75·3%; mean age ranged from 31·8 to 33·4. Primary diagnosis: Mental Illness 29·0% to 31·0%; learning disabilities from 38·7% to 42·7%. Between 47·1% and 50·5% had a secondary condition. Data were taken from records between 2002 and 2006 inclusive. | Analysis of electronic records in the US Rehabilitation Services Administration 911 database. The intervention was supported employment services received through state vocational rehabilitation agencies. There was no comparator. Duration: 1 week. | Public purse  2006  US Dollars | CEA | Not reported | 113,002 of 192,756 service users were in supported employment during study period (58·6%). All supported employees averaged 23·44 working hours per week. For services users with a primary diagnosis of mental illness or learning disability mean weekly working hours were 24·0 and 22·0 respectively. For services users with a primary diagnosis of mental illness or learning disability mean weekly working hours were 24·0 and 22·0 respectively. All supported employees averaged wages of $176·87 per week.  Overall, supported employees obtained services costing vocational rehabilitation services an average of $4,524·92 (SD = $7,147·44). (Note: period that costs cover is not explicitly stated and could conceivably be either costs per week or costs per year) For services users with a primary diagnosis of mental illness or learning disability these costs were $3,351 (SD = $4,315) and $5,134 (SD = $6,799) respectively. | Author states that when the average cost of services is divided by the average hours worked per week, the result indicates that $193·05 was spent by vocational rehabilitation for every hour the supported employee worked, and $25·58 was paid for every $1 earned. For services users with a primary diagnosis of mental illness these costs were $139·63 spent by vocational rehabilitation for every hour the supported employee worked, and $18·45 was paid for every $1 earned. For services users with a primary diagnosis of learning disabilities these costs were $233·36 was spent by vocational rehabilitation for every hour the supported employee worked, and $37·31 was paid for every $1 earned. | 34% |
| (Cimera 2009)  USA | Three male supported employees aged 18, 20 and 31 working at a McDonald's restaurant and three employees, 2 males and 1 female aged 16, 17 and 67, without disabilities with comparable job functions working at the restaurant. Dates of data collection not stated. | Pilot study matching workers hired from a local supported employment agency now in competitive employment. Matched with employees in same workplace with no disabilities and no use of supported employment. Duration: at least 972 days | Employer  Price year not stated  US Dollars | CBA | Not reported | Adam remained employed significantly longer than Zach (972 days versus 267 days). Bart maintained his job 3·11 times longer than did Yedda (413 days versus133 days). Calvin was employed for 326 days, compared to Xenos who was employed only 92 days (3·54 times longer). | Comparing the three employees Adam, Zach and Bart with their matched workers without disabilities there were net benefits to the employer of $892·83, $114·59 and $691·74 respectively. Supervision costs were $64·15 lower for Adam but greater for Bart $597·79 and Calvin $128·38 compared to matched controls. There were lower employee turnover costs for all three ($828·68, $708·02 and $805·92) because they remained employed longer than matched controls. | 43% |
| (Dattilo 2020)  USA | 65 people with mental health conditions referred by California State Department of Rehabilitation to Caminar’s Jobs Plus program at San Mateo County within the 2017-2019 fiscal years. Age and gender not stated. | Analysis of data from a supported employment agency, Jobs Plus. On-site versus off-site job coaching support as part of a supported employment programme Jobs Plus using the IPS model. 22 people chose off-site coaching and 42 people chose on-site coaching. Duration: 180days | Public purse  Not stated  US Dollars | CBA | On-site coaching had a greater reduction in hospitalisation days, from 5·12 days before referral to 1·79 days after. For off-site this was 3·05 days to 2·32 days. Significance not reported  . | Those using off-site coaching had an average of 8·41 weeks on the job during the 90-day probationary period, while those using on-site coaching had an average of 10·29 weeks on the job. Significance not reported. 71·43% of on-site group had cases successfully closed versus 54·55% in off-site group. | There were statistically significant reductions in health care costs of $10,897 in the on-site group post intervention. This compared with a reduction of $2,377 in the offsite group. Mean costs of providing coaching in the offsite and onsite groups were $284 and $1,732. There were net benefits of $9,165 and $2,093 in the onsite and offsite groups | 56% |
| (Drake et al 2009)  USA | 3·3 million US adult population of working age with mental health conditions who are enrolled in the US Security Disability Insurance (SSDI) or Supplemental Security Income (SSI) programmes in 2006. New potential entrants to programmes also included. | Modelling analysis drawing on published literature and national statistical sources, looking at economic benefits of participating in supported employment programmes. Model assumes 30% of people with low severity of mental illness and 40% of all those with moderate and high severity of mental illness will participate. Duration: 1 year. | Public purse and service user  2006  US Dollars | CBA | Model assumes a reduction of $5,000 per annum in Medicaid treatment costs for high severity group in supported employment. | Assuming a 30% enrolment into supported employment programmes of yearly new potential entrants to the US Social Security Disability Insurance (SSDI) and Supplemental Security Income (SSI) programme (173,000 people with mental health conditions) would lead to an increase in earnings of $107 million per annum in US. Additional 200 and 167 hours of work per annum for low and moderate severity groups. Little gain for severe group. Increased annual earnings of $679, $466 and $343 million for the low, moderate and high severity groups. | Overall net saving of $368 million per annum to the public purse. This consists of net costs to govt of $124 million and $262 million for low and moderate severity groups, but $706 million saving for the high severity group mainly due to health care savings.  From service user perspective increased annual earnings of $1·596 billion from supported employment enrolment. | 43% |
| (Evensen et al 2019)  Norway | 169 individuals aged between 18 and 65 and a broad schizophrenia spectrum disorder. Mean age 33·2 and 34·9 for intervention (JUMP) and treatment as usual (TAU) groups. 65% of both groups were male. 87% and 100% of JUMP and TAU groups had schizophrenia. | Analysis of registry data linked to participants in a multi-site vocational rehabilitation (VR) programme (JUMP programme) for adults  with schizophrenia spectrum disorders. The programme provided 10 months  of standard VR services in competitive or sheltered  workplaces. This was augmented with cognitive behavioural therapy (CBT) or Cognitive Remediation (CR). The comparator was TAU. Data were analysed two years before (T0) and two years after entering JUMP (T1).  Duration: 4 years. | Health and social care system  2015  Euros | CUA | For JUMP group at T0 mean QALYs were  0·8003 (SD .024) and 0·8043 (SD .024) at T1.  Specialist mental health costs reduced significantly in the JUMP group both for  those who gained paid employment (n = 21; mean = €  -80,776; 95% CI -140,112, − 21,467; p = .010) and those who had work placement or sheltered work (n = 42;  mean = € -90,885; 95% CI -153,873, − 27,897; p = .006). JUMP group had 25·5 inpatient days at T1 compared with 64·6 in TAU group (p=0·003) | 23·2% and 21·4% of JUMP and TAU groups were in employment at 2 year follow-up. Mean months of competitive employment in JUMP group were 3·10 (N = 69, SD 7·27) at T0 and 4·30 (N = 69, SD 7·33) at T1. Authors assumed stable 10·2% employment rate in the TAU group. | Mean cost of JUMP intervention was € 9131 (SD 2123) per participant. Mean duration of JUMP was 26·52 weeks (SD 5·89). Total mean costs for the JUMP group (inclusive of intervention costs and adjusted for baseline differences) were € 10,621 lower than for TAU (95% CI: -29,979, 8735; p = .282). With QALY gains and lower costs compared to TAU – JUMP was considered cost effective. It had an 85% likelihood of being cost effective in probabilistic sensitivity analysis. | 78% |
| (Fogelgren  2020)  Sweden | 1,062 young adults on disability pensions across 25 Swedish municipalities. Mean age was 25. Between 45% and 51% were female. 73% had mental health conditions, 17% learning disabilities and 10% other conditions. The study took place between November 2014 and December 2016. | Modelling study based on randomised trial lasting 1·5 years comparing supported employment (SE) with regular vocational rehabilitation (VR) which includes ‘in-house’ work preparation and work training, as well as case management. Duration: at least 12 years | Societal  Price year not stated  € | CBA | Not reported | At 18 month follow up 32% of the SE group were employed versus 22% for VR. This was statistically significant, but the definition of employment includes subsidised competitive employment. | The costs per participant for SE group were €2,781 compared to €2017 for VR using a bottom up costing approach. SE costs were €5,900 more expensive  per participant using a top down costing approach.  Based on an average difference in costs between interventions (€3,350) and assuming 0·33 of difference in employment probabilities between SE and VR is permanent, gains  from supported employment exceed costs after 12 years  . | 61% |
| (Greig et al 2014)  UK | Users of supported employment services across England. Data were collected in 2012 and 2013. | Mixed methods analysis of 70 supported employment services across England. 32 learning disability focused, 31 mental health focused. Plus in depth qualitative data collected in 11 local sites in England. Duration: 1 year | Health and social care  Price year not stated  UK Pounds | CEA | Not reported | 38% of all service users secured a job, of which 61% obtained a new job and 36% retained a job. 43% and 34% of people using learning disability and mental health services obtained employment. In 10 IPS services with good fidelity and 3 learning disability services following good practice 43% of users obtained employment. | The average cost per person supported was £1,730, £1,948 and £1,485 for all sites, learning disability services and mental health services respectively. Average cost per job was £8,217, £8,218 and £8,024 respectively. Average cost per job outcome in 10 IPS services with good fidelity and 3 learning disability services following good practice were much lower at £2,818 | 39% |
| (Hagen 2018)  Switzerland | 908 adults who had been on long term disability benefits who received intervention and matched controls from a sample of 14,878 who did not have intervention. Data collected between 2009 and 2011. Mean age 43·7, 53% male. 55·7% had one or more mental disorders. | Economic modelling analysis using administrative linked datasets. The intervention was placement coaching  by individually assigned advisers/coaches. Participants received active support in, and practical tips on, their search for suitable jobs for up to 12 months. Once in a job they also received support from coaches for a further 12 months. Duration 20 years. | Public purse  Not stated  Swiss Francs (CHF) | CBA | Not reported | Significant reductions in claims for disability insurance for intervention group compared to matched controls, with up to an 8% reduction or 146 CHF per month four years after intervention start. (p<0·01). Income earned from paid employment increased by up to 13·2% three years after intervention start or additional 4,017 CHF per annum. | Total cost per participant was CHF 8,819. Four long term modelling scenarios created. Under all scenarios there are positive net benefits comparing change in disability benefit and increase in taxes paid with programme costs. Expected mean long-run benefits exceed the mean costs by 1·9–6·5 times, or between CHF 8,000 and CHF 48,300 per participant | 70% |
| (Indecon 2016)  Ireland | 3,151 adults referred to 23 EmployAbility services in Ireland, each having a specific geographical remit. Data were collected between 2010 – 2014. | Analysis of official data on service use for The EmployAbility service, formerly known as the Supported Employment Programme (SEP), a national employment service dedicated to improving employment outcomes for jobseekers with a disability. Duration: 4 years | Public purse  Not stated  Euros | CEA | Not reported | 46·9% of clients exited programme while in employment and 83·3% of these then had 6 months of employment without support. Only 28% of all exits sustained at least 6 months of employment. | Average monthly expenditure per client varied from €222 to €258 per month over 2010-2014. Mean cost per client supported between €3,996 and €4,644 over this period. Mean spend per client exit to employment has fallen from €19,032 in 2012 to €11,433 in 2014. Mean service expenditure relative to employment sustained was €13,582 over the period 2013-2014 | 61% |
| (Kilsby et al 2011)  UK | 105 people with learning disabilities who were in work, or who had gained work, from 1st April 2010 to 31st March 2011 in the county of Kent, England. | Analysis of data provided by Kent County Council on use of their supported employment services compared with no intervention, which assumed use of day care services. Duration: 1 year | Service user and public purse  2012  UK Pounds | CBA | Not reported | Gross weekly income for employees from all sources after employment was £151·75 (wages + retained welfare benefits + working tax credits), an average increase on pre-work income of £71·18 per week. | The cost of per year per supported employee in employment was £10,252, compared with £12,792 for day services. From the taxpayer perspective KSE provides a net saving of £1,121 per person per annum compared to the day service alternative, with a cost benefit ratio of 0·12. Workers gained £71·18 per week.  . | 48% |
| (Schneider et al 2009)  UK | 141 users of support employment services. Three groups: already working and remained in same job, those who obtained work just prior to baseline or 12-month follow-up and those who remained unemployed throughout year. 43·3% were female, 83% White, 25·5% schizophrenia, 24·1% anxiety, 31·9% depression, 15·6% bipolar disorder. | Cohort analysis. Intervention was supported employment services that were close to IPS model. Comparison made between those who obtained work just prior to baseline or 12-month follow-up and those who remained unemployed throughout year  Duration: 1 year. | Public purse and societal  Price year not stated  UK Pounds | CCA | For employed < 12 months mental health service weekly costs were significantly lower at follow up compared to baseline. £36·71 (SD £45·76) versus £14·30 (SD £23·97) p<0·001. Similarly, for employed >12 months baseline £43·35 (SD £82·12) versus £23·19 (SD £46·33). p<0·01. There was no change for the unemployed group.  Overall weekly costs were significantly lower at follow up in the <12 months group £40·00 (SD £45·51) versus £30·34 (SD £69·20). Weekly overall health costs were also lower in the >12 months group £47·86 versus £28·31 but this was not quite significant. | Individuals entering work increased net mean earnings by £59 per week p=0·0.2. This was much greater than welfare benefits and allowances lost as a result of employment. | Costs of supported employment increased significantly for people who had worked for less than one year (p < 0·04) while they declined for those who remained unemployed (p < 0·001) as well as for those who had been working for longer (p <0·002). The median weekly cost of supported employment input was £15·75, and the cohort who started work reduced their consumption of mental health services by an average of £23·93 (range: £4·93–£52·78, p = 0·103) | 65% |
| (Sultan-Taib et al 2019)  Canada | 122 employees working in 19 Social Firms (SFs) across Quebec and 64 individuals participating in 2 supported employment programmes (SEP) in Montreal. 74% and 46% of participants were male. Mean age was 46 and 39·9. Primary mental health diagnosis : Schizophrenia 58% and 39%, Bipolar 9% and 6%, Major Depression 20% and 5%, Others 34% and 14%. | Cohort analysis. Non-competitive employment in social Firms was compared with supported employment programmes. Duration: 1 year. | Healthcare  2015  Canadian dollars | CUA | There was no significant difference in mean EQ-5D-5L (quality of life ) scores 76·87 vs 72·6 in the SF and SEP groups. Mean annual healthcare costs  were $3,600 (SD $4,304) in the SF group and $9,403 (SD 11,888·9) in the SEP group. Mean costs for outpatient visits, inpatient stays, medications were all significantly lower in the SF group. |  | There was a difference in health care costs of $5,803 (95% Confidence Interval [CI]: 3,433·2–8,173·3; p  < 0·001) in favour of SF. Controlling for covariates including EQ‐5D‐5L score, severity of psychiatric symptoms, gender and age  the multivariable analysis showed that the annual costs  per patient were significantly lower for the group of participants working in SFs $2,580 versus $4,774 a ($1,923·9 lower, 95% CI: 1,146·3–3,127·7; p = 0·004) | 70% |
| (Tholen et al 2017)  Sweden | 118 former pupils with learning disabilities, 69 of whom previously took part in supported employment programme in three upper secondary special schools in Örebro, Sweden. 56% were male. Data were collected between 2006 and 2013. | Modelling study drawing on data from before and after analysis using Registry data on employment status up to four years after leaving school. The intervention (Job In Sight – JIS) was supported employment was very similar to IPS but used internships as an intermediate step before full employment. A control group of 49 pupils left school before the JIS programme started. Duration: 4 years. | Local government  2013 prices  Swedish Krona | CBA, ROI | Not reported | Employment rates in the JIS group increased from 35% in the first year (T) to 67% in the last year (T+3).  Employment rates in the control group increased from 10% in the first year (T)  to 35% in the last year (T+3)  In the model an increase of the work share by one percentage unit was assumed to reduce the share of former pupils in day-activity programs by one percentage unit | The cost per pupil of intervention was 290,000 SEK for 3 years of support, equivalent to 114 % of the cost of a full school year. JIS would have a positive benefit cost ratio. Depending on discount rate (3% or 3·5%) used the benefit cost ratio was 4·987 or 3·09 respectively. There would be a be a net present value of 0·592 million SEK It would take 7·5 years to generate a positive return on investment. | 70% |

# List of excluded studies at full text stage

1. Supported employment: helping members achieve recovery and economic independence: Thresholds Supported Employment Program, Chicago, Illinois. Psychiatric Services (Washington, DC). 2009;60(10):1392-4.

2. VA to survey veterans about Vocational Rehabilitation and Employment program. Contemporary Rehab. 2009;65(5):7-.

3. Adding social value. Supply Management. 2009;14(17):26-7.

4. VR&E Has Strengthened Its Focus on Employment through the Five-Track Employment Process, but Has Not Updated Its Incentive Structure to Align with Its Mission. GAO Reports. 2009:8-.

5. VR&E Has Implemented Its Five-Track Employment Process and Strengthened Its Focus on Employment. GAO Reports. 2009:8-11.

6. MENTAL HEALTH SUPPORT. Equal Opportunities Review. 2009(195):7-.

7. Identifying strengths, goals key in supported employment arena. Mental Health Weekly. 2011;21(24):4-5.

8. Supported employment raises potential for clients in San Diego system. Mental Health Weekly. 2011;21(20):1-8.

9. JOBS NY assists people with psychiatric disabilities find sustainable employment. Contemporary Rehab. 2011;67(2):18-.

10. The Poppy Factory builds on employment support. Design Week (Online Edition). 2011:7-.

11. The Factors Influence upon Job Maintenance of the Mentally Disabled with Job Experience. Korean Journal of Occupational Health Nursing. 2012;21(1):1p-p.

12. Corrigendum...Becker DR, Drake RE, Bond GR. Benchmark outcomes in supported employment. American Journal of Psychiatric Rehabilitation. 2011;14(3):230–236. American Journal of Psychiatric Rehabilitation. 2012;15(2):238-.

13. Updates from the national rehabilitation association job placement & development (NRAJPD) division. Contemporary Rehab. 2012;68(3):10-.

14. Vocational Rehabilitation Programs. Exceptional Parent. 2012;42(1):84-5.

15. Job support lacking from mental health services. RoSPA Occupational Safety & Health Journal. 2012;42(11):4-.

16. Little Is Known about the Effectiveness of Fragmented and Overlapping Programs. GAO Reports. 2012:1-85.

17. Vocational Rehabilitation Programs. Exceptional Parent. 2013;43(1):85-6.

18. Twenty-five years of APSE: The Association of People Supporting EmploymentFirst. Journal of Vocational Rehabilitation. 2013;38(3):157-62.

19. Final priority; Rehabilitation Training: Rehabilitation Long-Term Training program--rehabilitation specialty areas. Final priority. Federal Register. 2014;79(141):42680-3.

20. Supported employment benefits significant beyond five years. Mental Health Weekly. 2014;24(34):5-6.

21. Vocational Rehabilitation Programs. Exceptional Parent. 2014;44(1):81-2.

22. Final Priority and Definitions; Demonstration and Training Program: Career Pathways for Individuals With Disabilities. Final priority and definitions. Federal Register. 2015;80(151):46799-804.

23. Final Priority; Rehabilitation Training: Vocational Rehabilitation Technical Assistance Center--Youth With Disabilities. Final priority. Federal Register. 2015;80(146):45423-8.

24. Final Priority and Definitions--Rehabilitation Training: Vocational Rehabilitation Technical Assistance Center-Target Communities. Final priority and definitions. Federal Register. 2015;80(157):48696-702.

25. Final Priority. Rehabilitation Training: Vocational Rehabilitation Workforce Innovation Technical Assistance Center. Final priority. Federal Register. 2015;80(156):48443-9.

26. Bazelon Center publishes fact sheets on supported employment. Mental Health Weekly. 2015;25(22):8-.

27. Vocational Rehabilitation Programs. Exceptional Parent. 2015;45(1):83-4.

28. Social Return on Investment of an Innovative Employment Option for Persons with Developmental Disabilities. John Wiley & Sons, Inc.; 2015. p. 209-28.

29. PAVE Power. PN. 2016;70(4):18-9.

30. 'Reverse jobs fair' to get disabled people into work. Equal Opportunities Review. 2016(264):6-.

31. Training in adult social care. Fenman Ltd.; 2016. p. 12-3.

32. Notice of Retraction...Summative evaluation of the Employment First State Leadership Mentoring Project 2015 program year. Journal of Vocational Rehabilitation, 47(1)(2017), 25-45. IOS Press; 2017. p. 259-.

33. NHS ROLLS OUT HEALTH EMPLOYMENT SPECIALIST SCHEME. Healthcare Counselling & Psychotherapy Journal. 2018;18(4):5-.

34. VOCATIONAL REHABILITATION: Additional Federal Information Could Help States Serve Employers and Find Jobs for People with Disabilities. GAO Reports. 2018:i-66.

35. Statement of retraction: individual placement and support in Europe: the EQOLISE trial. International Review Of Psychiatry (Abingdon, England). 2019;31(5-6):543-.

36. Statement of retraction: individual placement and support in Europe: the EQOLISE trial...Burns T, White SJ, Catty J et al. The Eqolise Group, Individual Placement and Support in Europe: The EQOLISE trial, International Review of Psychiatry, 20:6, 2008, 498–502. International Review of Psychiatry. 2019;31(5/6):543-.

37. Error in Text. JAMA Psychiatry. 2019;76(12):1319-.

38. NHS mental health job coaches. Healthcare Counselling & Psychotherapy Journal. 2019;19(4):4-.

39. Aakvik A, Holmås TH, Kjerstad E. Prioritization and the elusive effect on welfare - a Norwegian health care reform revisited. Social Science & Medicine (1982). 2015;128:290-300.

40. Aas RW, Haveraaen LA, Brouwers EPM, Skarpaas LS. Who among patients with acquired brain injury returned to work after occupational rehabilitation? The rapid-return-to-work-cohort-study. Disability And Rehabilitation. 2018;40(21):2561-70.

41. Aas RW, Raanaas RK, Shaw L. Unifying and diversifying workplace-based efforts for promoting health and preventing disability. Work. 2016;53(1):3-7.

42. Aasdahl L, Foldal VS, Standal MI, Hagen R, Johnsen R, Solbjør M, et al. Motivational interviewing in long-term sickness absence: study protocol of a randomized controlled trial followed by qualitative and economic studies. BMC Public Health. 2018;18(1):756-.

43. Abdel-Baki A, Létourneau G, Morin C, Ng A. Resumption of work or studies after first-episode psychosis: the impact of vocational case management. Early Intervention In Psychiatry. 2013;7(4):391-8.

44. Abraham KM, Chang M-UM, Van T, Resnick SG, Zivin K. Employment After Vocational Rehabilitation Predicts Decreased Health Care Utilization in Veterans With Mental Health Diagnoses. Military medicine. 2021.

45. Abraham KM, Ganoczy D, Yosef M, Resnick SG, Zivin K. Receipt of employment services among Veterans Health Administration users with psychiatric diagnoses. Journal Of Rehabilitation Research And Development. 2014;51(3):401-14.

46. Abraham KM, Yosef M, Resnick SG, Zivin K. Competitive Employment Outcomes Among Veterans in VHA Therapeutic and Supported Employment Services Programs. Psychiatric Services (Washington, DC). 2017;68(9):938-46.

47. Abrams B, Forman L. Opportunities for success. Developmental Medicine and Child Neurology. 2010;52(SUPPL. 5):98.

48. Adamecz-Völgyi A, Lévay PZ, Bördős K, Scharle Á. Impact of a personalised active labour market programme for persons with disabilities. Scandinavian Journal Of Public Health. 2018;46(19_suppl):32-48.

49. Adams S, Ned L, Kahonde C. Exploring possibilities for the effective transitioning of persons with intellectual disabilities into supported employment. Journal of Intellectual Disability Research. 2019;63(7):868.

50. Addington J. Psychosocial treatment in the RAISE-ETP study. Early Intervention in Psychiatry. 2014;8(SUPPL. 1):1.

51. Addington J, Piskulic D, Marshall C, Addington AB. Psychosocial treatments for schizophrenia. Current Directions in Psychological Science. 2010;19(4):260-3.

52. Agovino M, Rapposelli A, Agovino AB. Disability and work: A two-stage empirical analysis of Italian evidence at provincial level in providing employment for disabled workers. Social Indicators Research. 2016;125(2):635-48.

53. Ahonle Z, Romero S, Barnes M, Sorrells A. Vocational Rehabilitation as a Public Health Intervention for Individuals with Traumatic Brain Injury. Archives of Physical Medicine and Rehabilitation. 2019;100(12):e211.

54. Ahonle ZJ, Barnes M, Romero S, Sorrells AM, Brooks GI. State-Federal Vocational Rehabilitation in Traumatic Brain Injury: What Predictors Are Associated With Employment Outcomes? Rehabilitation Counseling Bulletin. 2020;63(3):143-55.

55. Aida T, Yamada T. Effective professional process of vocational rehabilitation for clients with higher brain dysfunctions. Brain Injury. 2016;30(5-6):754.

56. Aida T, Yamada T. Effect of medical welfare cooperation of "vocational rehabilitation planning sheet for people with cognitive disorders after acquired brain injury". Neurorehabilitation and Neural Repair. 2018;32(4-5):368-9.

57. Aisia JBL, Yu JMS, Da L, Tsang HWH. Implementing Integrated Supported Employment in Mainland China: A Case Vignette. Journal of Rehabilitation. 2015;81(3):51-7.

58. Aizawa K, Hisanaga F. Employment Support Services in Japan. International Journal of Mental Health. 2012;41(2):48-60.

59. Aksnes SY. Engaging employers in vocational rehabilitation: Understanding the new significance of knowledge brokers. Journal of Vocational Rehabilitation. 2019;50(1):73-84.

60. Aksnes SY, Becker BB. Engaging employers in vocational rehabilitation: Understanding the new significance of knowledge brokers. Journal of Vocational Rehabilitation. 2019;50(1):73-84.

61. Al-Abdulmunem M, Drake RE, Carpenter-Song E. Evidence-Based Supported Employment in the Rural United States: Challenges and Adaptations. Psychiatric Services (Washington, DC). 2021;72(6):712-5.

62. Alexander L, Cooper K, Mitchell D. Effectiveness of vocational rehabilitation on work par-ticipation in adults with musculoskeletal disorders: An umbrella review. Physiotherapy (United Kingdom). 2017;103(Supplement 1):e102.

63. Alexander L, Cooper K, Mitchell D, MacLean C. Effectiveness of vocational rehabilitation on work participation in adults with musculoskeletal disorders: an umbrella review protocol. JBI Database Of Systematic Reviews And Implementation Reports. 2017;15(6):1518-21.

64. Allaire SJ, Niu J, Zhu Y, Brett B. Providing Effective Early Intervention Vocational Rehabilitation at the Community Level. Rehabilitation Counseling Bulletin. 2011;54(3):154-63.

65. Allott K. Cat in first-episode psychosis: Feasibility, acceptability and potential to enhance vocational recovery. Schizophrenia Bulletin. 2018;44(Supplement 1):S32.

66. Allott K, Bartholomeusz C, Thompson A, Wood S, Killackey E. Bolstering work: Potential benefits of cognitive and social cognitive interventions to employment interventions for people with early psychosis. Schizophrenia Research. 2012;136(SUPPL. 1):S38.

67. Allott K, Cotton S, Killackey E. The relationship between neurocognition, social cognition and vocational engagement in first-episode psychosis. Early Intervention in Psychiatry. 2014;8(SUPPL. 1):81.

68. Allott KA, Chinnery GL, Cotton SM, Jackson HJ, Killackey EJ. Does individual placement and support compensate for neurocognitive deficit in first-episode psychosis? Early Intervention in Psychiatry. 2012;6(SUPPL.1):14.

69. Allott KA, Cotton SM, Chinnery GL, Baksheev GN, Massey J, Sun P, et al. The relative contribution of neurocognition and social cognition to 6-month vocational outcomes following Individual Placement and Support in first-episode psychosis. Schizophrenia Research. 2013;150(1):136-43.

70. Allott KA, Turner LR, Chinnery GL, Killackey EJ, Nuechterlein KH. Managing disclosure following recent-onset psychosis: utilizing the individual placement and support model. Early Intervention In Psychiatry. 2013;7(3):338-44.

71. Almalki S. A qualitative study of supported employment practices in Project SEARCH. International Journal of Developmental Disabilities. 2019.

72. Almalki S, Brooke CD. A qualitative study of supported employment practices in project search. International Journal of Developmental Disabilities. 2019:No-Specified.

73. Al-Rashaida M, López-Paz JF, Amayra I, Lázaro E, Martínez O, Berrocoso S, et al. Factors affecting the satisfaction of people with disabilities in relation to vocational rehabilitation programs: A literature review. Journal of Vocational Rehabilitation. 2018;49(1):97-115.

74. Alsaman MA, Lee C-L. Employment Outcomes of Youth With Disabilities in Vocational Rehabilitation. Rehabilitation Counseling Bulletin. 2017;60(2):98-107.

75. Alsaman MA, Lee C-L, Austin BB. Employment outcomes of youth with disabilities in vocational rehabilitation: A multilevel analysis of RSA-911 data. Rehabilitation Counseling Bulletin. 2017;60(2):98-107.

76. Alvarez JC, Tourino R, Abelleira C, Fernandez J, Baena E, Giraldez A, et al. Social cognition and schizophrenia: Differences between users of psychosocial rehabilitation day center and from a supported employment program. Rehabilitacion Psicosocial. 2013;10(2):4-9.

77. Alverson CY, Yamamoto SH. VR Employment Outcomes of Individuals with Autism Spectrum Disorders: A Decade in the Making. Journal of Autism & Developmental Disorders. 2018;48(1):151-62.

78. Alverson CY, Yamamoto SH, Alverson AB. Employment outcomes of vocational rehabilitation clients with autism spectrum disorders. Career Development and Transition for Exceptional Individuals. 2017;40(3):144-55.

79. Amado I. Early detection and rehabilitation. A specific device for care and integration in the community. Annales Medico-Psychologiques. 2018;176(1):80-3.

80. Amado I, Abdel-Baki. Early detection and rehabilitation. A specific device for care and integration in the community. Detection precoce et rehabilitation La place d'un dispositif specifique. 2018;176(1):80-3.

81. Andersén Å, Larsson K, Lytsy P, Berglund E, Kristiansson P, Anderzén I. Strengthened General Self-Efficacy with Multidisciplinary Vocational Rehabilitation in Women on Long-Term Sick Leave: A Randomised Controlled Trial. Journal Of Occupational Rehabilitation. 2018;28(4):691-700.

82. Andersén Å, Larsson K, Pingel R, Kristiansson P, Anderzén I. The relationship between self-efficacy and transition to work or studies in young adults with disabilities. Scandinavian Journal Of Public Health. 2018;46(2):272-8.

83. Andersén Å, Ståhl C, Anderzén I, Kristiansson P, Larsson K. Positive experiences of a vocational rehabilitation intervention for individuals on long-term sick leave, the Dirigo project: a qualitative study. BMC Public Health. 2017;17:1-10.

84. Andersen MF, Nielsen KM, Brinkmann S, Aas AA. Meta-synthesis of qualitative research on return to work among employees with common mental disorders. Scandinavian Journal of Work, Environment & Health. 2012;38(2):93-104.

85. Anderson CB, Smart JF, Arias AABBBCDF-PFFFFFFFGHHHHKKKKLLMMM. Improving transition outcomes for culturally and linguistically diverse VR consumers. Journal of Applied Rehabilitation Counseling. 2010;41(4):3-10.

86. Andrén D, Svensson M. Part-Time Sick Leave as a Treatment Method for Individuals with Musculoskeletal Disorders. Journal of Occupational Rehabilitation. 2012;22(3):418-26.

87. Anema JR, Schellart AJM, Cassidy JD, Loisel P, Veerman TJ, van der Beek AJ. Can cross country differences in return-to-work after chronic occupational back pain be explained? An exploratory analysis on disability policies in a six country cohort study. Journal Of Occupational Rehabilitation. 2009;19(4):419-26.

88. Angell B, Matthews E, Barrenger S, Watson AC, Draine J, Binswanger BCCCCDDDDDFGGHHHHKKKLLLLLLMMMM. Engagement processes in model programs for community reentry from prison for people with serious mental illness. International Journal of Law and Psychiatry. 2014;37(5):490-500.

89. Angothu H, Jayarajan D, Prasad K, Sivakumar T. Models of vocational rehabilitation for persons with severe mental illness-nimhans experiences and challenges. Indian Journal of Psychiatry. 2018;60(5 Supplement 1):S35.

90. Anonymous. "The feasibility of implementing cognitive remediation for work in community based psychiatric rehabilitation programs": Correction to McGurk et al. (2017). Psychiatric rehabilitation journal. 2017;40(2):206.

91. Anonymous. RETRACTED ARTICLE: Individual Placement and Support in Europe: The EQOLISE trial (International Review of Psychiatry, (2008), 20, 6, (498-502), 10.1080/09540260802564516). International Review of Psychiatry. 2019;31(5-6):543.

92. Anonymous. Correction: Effects of individual placement and support supplemented with cognitive remediationand work-focused social skills training for people with severe mental illness: A randomized clinical trial (JAMA Psychiatry (2019) DOI: 10.1001/jamapsychiatry.2019.2291). JAMA Psychiatry. 2019;76(12):1319.

93. Anwar Z, Grover P, Ahmed S, Das D. Journey of an early intervention in psychosis service. Early Intervention in Psychiatry. 2012;6(SUPPL.1):109.

94. Arbesman M, Logsdon DW. Occupational therapy interventions for employment and education for adults with serious mental illness: a systematic review. The American Journal Of Occupational Therapy: Official Publication Of The American Occupational Therapy Association. 2011;65(3):238-46.

95. Arbesman M, Logsdon DW, Anzai ABBBBBCCCCDDDEFGGGHHIKKKLiLMMM. Occupational therapy interventions for employment and education for adults with serious mental illness: A systematic review. Special Issue: Effectiveness of occupational therapy services in mental health practice. 2011;65(3):238-46.

96. Arblaster K, Urlic K, McCluskey A. Critically appraised papers. Organisational changes leading to use of Assertive Community Treatment and supported employment improve outcomes for people with severe mental illness. Australian Occupational Therapy Journal. 2009;56(5):362-4.

97. Areberg C, Bejerholm U. The effect of IPS on participants' engagement, quality of life, empowerment, and motivation: a randomized controlled trial. Scandinavian Journal Of Occupational Therapy. 2013;20(6):420-8.

98. Areberg C, Björkman T, Bejerholm U. Experiences of the individual placement and support approach in persons with severe mental illness. Scandinavian Journal Of Caring Sciences. 2013;27(3):589-96.

99. Arends I, Bruinvels DJ, Rebergen DS, Nieuwenhuijsen K, Madan I, Neumeyer-Gromen A, et al. Interventions to facilitate return to work in adults with adjustment disorders. The Cochrane Database Of Systematic Reviews. 2012;12:CD006389.

100. Arikawa M, Goto H, Mineno K. Job support by occupational therapists for people with developmental disabilities: two case studies. Work (Reading, Mass). 2013;45(2):245-51.

101. Atterbury K. Rethinking Why We Do What We Do: Individual Placement and Support. Psychiatric Services (Washington, DC). 2021:appips202000654.

102. Au DWH, Tsang HWH, So WWY, Bell MD, Cheung V, Yiu MGC, et al. Effects of integrated supported employment plus cognitive remediation training for people with schizophrenia and schizoaffective disorders. Schizophrenia Research. 2015;166(1-3):297-303.

103. Audhoe SS, Hoving JL, Sluiter JK, Frings-Dresen MHW. Vocational Interventions for Unemployed: Effects on Work Participation and Mental Distress. A Systematic Review. Journal of Occupational Rehabilitation. 2009:1-13.

104. Audhoe SS, Hoving JL, Sluiter JK, Frings-Dresen MHW. Vocational interventions for unemployed: effects on work participation and mental distress. A systematic review. Journal Of Occupational Rehabilitation. 2010;20(1):1-13.

105. Audhoe SS, Nieuwenhuijsen K, Hoving JL, Sluiter JK, Frings-Dresen MHW. The effectiveness of the "Brainwork Intervention" in reducing sick leave for unemployed workers with psychological problems: design of a controlled clinical trial. BMC Public Health. 2015;15:377-.

106. Aust B, Nielsen MBD, Grundtvig G, Buchardt HL, Ferm L, Andersen I, et al. Implementation of the Danish return-to-work program: process evaluation of a trial in 21 Danish municipalities. Scandinavian Journal Of Work, Environment & Health. 2015;41(6):529-41.

107. Austin BS, Chun-Lung L. A Structural Equation Model of Vocational Rehabilitation Services: Predictors of Employment Outcomes for Clients with Intellectual and Co-occurring Psychiatric Disabilities. Journal of Rehabilitation. 2014;80(3):11-20.

108. Austin BS, Fleming A, Chun-Lung L, Sukyeong P. Vocational Rehabilitation Outcomes for Individuals with Intellectual Disabilities and Co-Occurring Psychiatric Disorders. Journal of Rehabilitation. 2019;85(4):14-23.

109. Autret K, Zouker J, Albanese J-B, Berthier T, Durufle A, Le Meur C, et al. Return to work after brain injury: a retrospective study of 85 patients followed by an occupational reintegration unit. Annals Of Physical And Rehabilitation Medicine. 2015;58(5):308-11.

110. Awsumb JM, Balcazar FE, Alvarado F. Vocational Rehabilitation Transition Outcomes of Youth With Disabilities From a Midwestern State. Rehabilitation Research, Policy & Education. 2016;30(1):48-64.

111. Axe J, Childs E, Manion K. In search of employment: Tackling youth homelessness and unemployment. Children & Youth Services Review. 2020;113:N.PAG-N.PAG.

112. Azorin JM, Adida M, Belzeaux R, Fakra E. [A model of care for first-episode psychosis: the RAISE-ETP project]. L'encephale. 2016;42 Suppl 3:S13-S7.

113. Ba MB, Tettamanti M, Bessero S, Curtis L. Job/education coaching in young adults with mental health needs: Users and staff s evaluations. Early Intervention in Psychiatry. 2014;8(SUPPL. 1):82.

114. Baert S, vanderKlaauw B, vanLomwel G. The effectiveness of medical and vocational interventions for reducing sick leave of self-employed workers. Health Economics (United Kingdom). 2018;27(2):e139-e52.

115. Baker-Ericzén MJ, Fitch MA, Kinnear M, Jenkins MM, Twamley EW, Smith L, et al. Development of the Supported Employment, Comprehensive Cognitive Enhancement, and Social Skills program for adults on the autism spectrum: Results of initial study. Autism: The International Journal Of Research And Practice. 2018;22(1):6-19.

116. Bakker C, Came K. Tetauoko Nga Tangata - Mental health employment social impact bond. Australian and New Zealand Journal of Psychiatry. 2018;52(1 Supplement 1):77.

117. Baksheev GN, Allott K, Jackson HJ, McGorry PD, Killackey E. Predictors of vocational recovery among young people with first-episode psychosis: findings from a randomized controlled trial. Psychiatric Rehabilitation Journal. 2012;35(6):421-7.

118. Bal MI, Roelofs PPDM, Hiberink SR, van Meeteren J, Stam HJ, Roebroeck ME, et al. Entering the labor market: increased employment rates of young adults with chronic physical conditions after a vocational rehabilitation program. Disability And Rehabilitation. 2019:1-8.

119. Bal MI, Roelofs PPDM, Hilberink SR, van Meeteren J, Stam HJ, Roebroeck ME, et al. Entering the labor market: increased employment rates of young adults with chronic physical conditions after a vocational rehabilitation program. Disability And Rehabilitation. 2021;43(14):1965-72.

120. Bal MI, Sattoe JNT, van Schaardenburgh NR, Floothuis MCSG, Roebroeck ME, Miedema HS. A vocational rehabilitation intervention for young adults with physical disabilities: participants' perception of beneficial attributes. Child: Care, Health And Development. 2017;43(1):114-25.

121. Balcazar FE, Oberoi A, Keel JM. Predictors of Employment and College Attendance Outcomes for Youth in Transition: Implications for Policy and Practice. Journal of Applied Rehabilitation Counseling. 2013;44(1):38-45.

122. Balcazar FE, Oberoi AK, Suarez-Balcazar Y, Alvarado F. Predictors of Outcomes for African Americans in a Rehabilitation State Agency: Implications for National Policy and Practice. Rehabilitation Research, Policy & Education. 2012;26(1):43-54.

123. Balcazar FE, Taylor-Ritzler T. Perspectives of vocational rehabilitation counselors on the factors related to employment outcomes of racial and ethnic minorities with disabilities. Journal Of Social Work In Disability & Rehabilitation. 2009;8(3-4):102-16.

124. Baldwin C, Brusco NK. The Effect of Vocational Rehabilitation on Return-to-Work Rates Post Stroke: A Systematic Review. Topics in Stroke Rehabilitation. 2011;18(5):562-72.

125. Baldwin C, Brusco NK, Baker BB-SBCDH-MJJjKKKLLMMMMSSSS-PSTVWZ. The effect of vocational rehabilitation on return-to-work rates post stroke: A systematic review. Topics in Stroke Rehabilitation. 2011;18(5):562-72.

126. Baller JB, Blyler CR, Bronnikov S, Xie H, Bond GR, Filion K, et al. Long-Term Follow-Up of a Randomized Trial of Supported Employment for SSDI Beneficiaries With Mental Illness. Psychiatric Services (Washington, DC). 2019:appips201800554-appips.

127. Baller JB, Blyler CR, Bronnikov S, Xie H, Bond GR, Filion K, et al. Long-Term Follow-Up of a Randomized Trial of Supported Employment for SSDI Beneficiaries With Mental Illness. Psychiatric Services (Washington, DC). 2020;71(3):243-9.

128. Ballou M, Balogun O, Gittens G, Atsushi M, Sanchez W. Trauma and Returning to Work: Women's Lived Experiences and its Implications for Vocational Rehabilitation Counseling. Journal of Applied Rehabilitation Counseling. 2015;46(1):25-33.

129. Baloch NA, Jennings WG. Offender vocational rehabilitation services and postrelease employment: A case for inmates with disabilities. Journal of Offender Rehabilitation. 2018:No-Specified.

130. Balogun-Mwangi O, Rogers ES, Maru M, Magee C. Vocational Peer Support: Results of a Qualitative Study. The Journal Of Behavioral Health Services & Research. 2019;46(3):450-63.

131. Bangen R, Gobes K. [Vocational rehabilitation: first place, then train--contra]. Psychiatrische Praxis. 2014;41(6):295-6.

132. Banks P, Jahoda A, Dagnan D, Kemp J, Williams V. Supported employment for people with intellectual disability: the effects of job breakdown on psychological well-being. Journal of Applied Research in Intellectual Disabilities. 2010;23(4):344-54.

133. Banks P, Jahoda A, Dagnan D, Kemp J, Williams V, Beyer BBC-RCCDFF-JGH-MJJKKKMPRSTVW. Supported employment for people with intellectual disability: The effects of job breakdown on psychological well-being. Journal of Applied Research in Intellectual Disabilities. 2010;23(4):344-54.

134. Barclay L, Lalor A, Migliorini C, Robins L. A comparative examination of models of service delivery intended to support community integration in the immediate period following inpatient rehabilitation for spinal cord injury. Spinal Cord. 2019.

135. Barreira PJ, Tepper MC, Gold PB, Holley D, Macias C. Adapting evidence-based interventions to fit usual practice: staff roles and consumer choice in psychiatric rehabilitation. The Psychiatric Quarterly. 2010;81(2):139-55.

136. Barreira PJ, Tepper MC, Gold PB, Holley D, Macias C. Social value of supported employment for psychosocial program participants. The Psychiatric Quarterly. 2011;82(1):69-84.

137. Bartels SJ, Pratt SI. Psychosocial rehabilitation and quality of life for older adults with serious mental illness: recent findings and future research directions. Current Opinion In Psychiatry. 2009;22(4):381-5.

138. Bassett E. Early intervention for young adult veterans with psychosis: Recovery-oriented care within the San Francisco VA health care system. Early Intervention in Psychiatry. 2018;12(Supplement 1):213.

139. Bassey E, Ellison C, Walker R. Perception of blind rehabilitation services among adults with acquired blindness in Nigeria: Attention to functional goals. British Journal of Visual Impairment. 2019;37(1):6-16.

140. Bates-Maves JK, O'Sullivan D. Making the Case for Specialized Caseloads Among Vocational Rehabilitation Counselors Working With Ex-Offenders: A Pilot Study. Rehabilitation Research, Policy & Education. 2017;31(2):121-34.

141. Bates-Maves JK, O'Sullivan D, Angerer BBBCCCCCCCCCCCDFGHHHHHHHHJKKKL. Making the case for specialized caseloads among vocational rehabilitation counselors working with ex-offenders: A pilot study. Rehabilitation Research, Policy and Education. 2017;31(2):121-34.

142. Baum N, Neuberger T, Ahrens ABBBBBBCCCCCDDDDDDGGGHHHJJKKKL. The contributions of persons in the work environment to the self-identity of persons with mental health problems: A study in Israel. Health & Social Care in the Community. 2014;22(3):308-16.

143. Baxter C, Prior S, Forsyth K, Maciver D, Meiklejohn A, Irvine L, et al. Mental health vocational rehabilitation -occupational therapists' perceptions of individual placement and support. International Journal of Therapy & Rehabilitation. 2012;19(4):217-25.

144. Beach DT, Anthony ABBBCCCFFHHLLMTWWW. Predicting employment outcomes of consumers of state-operated comprehensive rehabilitation centers. Rehabilitation Counseling Bulletin. 2009;52(3):147-55.

145. Bechi M, Spangaro M, Pigoni A, Ripamonti E, Buonocore M, Cocchi F, et al. Exploring predictors of work competence in schizophrenia: The role of theory of mind. Neuropsychological Rehabilitation. 2019;29(5):691-703.

146. Beck C, Wernham C. Improving access to competitive employment for service users in forensic psychiatric units. BMJ Quality Improvement Reports. 2014;3(1).

147. Becker DR. Hiring peers as vocational specialists. Psychiatric Services. 2015;66(4):337.

148. Becker DR, Bond GR. Commentary on special issue on individual placement and support (IPS) international. Psychiatric Rehabilitation Journal. 2020;43(1):79-82.

149. Becker DR, Drake RE, Bond GR. Benchmark Outcomes in Supported Employment...[corrected][published erratum appears in AM J PSYCHIATR REHABIL 2012 Apr; 15(2): 238]. American Journal of Psychiatric Rehabilitation. 2011;14(3):230-6.

150. Becker DR, Drake RE, Bond GR. "Benchmark outcomes in supported emplacement": Corrigendum. American Journal of Psychiatric Rehabilitation. 2012;15(2):238.

151. Becker DR, Drake RE, Bond GR. The IPS supported employment learning collaborative. Psychiatric Rehabilitation Journal. 2014;37(2):79-85.

152. Becker DR, Drake RE, Bond GR. The IPS supported employment learning collaborative...individual placement and support. Psychiatric Rehabilitation Journal. 2014;37(2):79-85.

153. Becker DR, Drake RE, Bond GR, Becker BBBBBBBDFGKKKRT. Benchmark outcomes in supported employment. American Journal of Psychiatric Rehabilitation. 2011;14(3):230-6.

154. Becker DR, Drake RE, Bond GR, Nawaz S, Haslett WR, Martinez RA. Best practices: A national mental health learning collaborative on supported employment. Psychiatric Services (Washington, DC). 2011;62(7):704-6.

155. Becker DR, Drake RE, Bond GR, Nawaz S, Haslett WR, Martinez RA, et al. A national mental health learning collaborative on supported employment. Psychiatric Services. 2011;62(7):704-6.

156. Becker T, Guehne U, Riedel-Heller S. Evidence-based psychosocial measures in rehabilitation. European Psychiatry. 2016;33(SUPPL.):S6.

157. Becker T, Kösters M. Psychosocial interventions in people with severe mental illness: a Bleulerian perspective. Neuropsychobiology. 2012;66(1):70-5.

158. Becker T, Kosters M, Alwan BBBBBBBBBBCCCCDDDFGHJJJKLMMMNR. Psychosocial interventions in people with severe mental illness: A Bleulerian perspective. Special Issue: 100 years of schizophrenia: Symposium '100 Years of Schizophrenia', Marburg, May 21, 2011. 2012;66(1):70-5.

159. Bedekar Y. Vocational rehabilitation: A model for stroke. International Journal of Stroke. 2009;4(SUPPL. 2):7.

160. Bedggood J, Csiernik R. Appointment choice and outcome at a supported employment agency. Journal Of Evidence-Based Social Work. 2009;6(4):390-400.

161. Beemster TT, van Velzen JM, van Bennekom CAM, Frings-Dresen MHW, Reneman MF. Cost-effectiveness of 40-hour versus 100-hour vocational rehabilitation on work participation for workers on sick leave due to subacute or chronic musculoskeletal pain: study protocol for a randomized controlled trial. Trials. 2015;16:317-.

162. Bégin É, Corbière M. Les compétences perçues de la personne ayant un trouble mental grave: un facteur significatif de maintien en emploi. Canadian Journal of Community Mental Health. 2012;31(2):35-50.

163. Begin E, Corbiere M, Auerbach BBBBBBCCCCCCCCDDDDDDDDDEFGGGHH. The perceived competence of persons with severe mental illness: A significant factor in job retention. Les competences percues de la personne ayant un trouble mental grave: Un facteur significatif de maintien en emploi. 2012;31(2):35-50.

164. Behan C, Masterson S, Roche E, Renwick L, McDonough C, Waddington J, et al. Cost-effectiveness of early intervention in psychosis in comparison to treatment as usual. Journal of Mental Health Policy and Economics. 2015;18(SUPPL. 1):S3-S4.

165. Beiler J. [Is "place and train" the Right Way?]. Die Rehabilitation. 2013;52(4):280-3.

166. Beiler J. [Employability as a problem of the vocational rehabilitation of the disabled]. Die Rehabilitation. 2013;52(5):352-6.

167. Beiler J. Is "place and traino" the right way?: Notes to a new approach of the RehaFutur-project. Rehabilitation (Germany). 2013;52(4):280-3.

168. Beimers D, Biege DE, Guo S, Stevenson LD, Allison BBBBBBBBBBCCCCCC-RDDDDDFGGHKLLL. Employment entry through supported employment: Influential factors for consumers with co-occurring mental and substance disorders. Best Practices in Mental Health: An International Journal. 2010;6(2):85-102.

169. Beimers D, Gatlin E, Aarons ABBBBBBBBBBBBBCCDDDDFGGGGGHHHH. Supported employment in rural areas: Implications for mental health practice. Journal of Rural Mental Health. 2011;35(2):3-11.

170. Bejerholm U, Areberg C, Hofgren C, Sandlund M, Rinaldi M. Individual placement and support in Sweden - a randomized controlled trial. Nordic Journal Of Psychiatry. 2015;69(1):57-66.

171. Bejerholm U, Björkman T. Empowerment in supported employment research and practice: is it relevant? The International Journal Of Social Psychiatry. 2011;57(6):588-95.

172. Bejerholm U, Bjorkman T, Bejerholm BBBBBBBBCCCCCDEHKLLLLLMNOPRRSS. Empowerment in supported employment research and practice: Is it relevant? International Journal of Social Psychiatry. 2011;57(6):588-95.

173. Bejerholm U, Larsson L, Hofgren C. Individual placement and support illustrated in the Swedish welfare system: A case study. Journal of Vocational Rehabilitation. 2011;35(1):59-72.

174. Bejerholm U, Larsson ME, Johanson S. Supported employment adapted for people with affective disorders-A randomized controlled trial. Journal Of Affective Disorders. 2017;207:212-20.

175. Bejerholm U, Larsson ME, Johanson S, Adler AAAABBBBBBBBBBBBBBBCCCCCCCCDDV. Supported employment adapted for people with affective disorders-A randomized controlled trial. Journal of Affective Disorders. 2017;207:212-20.

176. Bell E. Competitive employment for consumers who are legally blind: a 10-year retrospective study. Journal Of Rehabilitation Research And Development. 2010;47(2):109-16.

177. Bell MD. Cognitive remediation and supported employment: Neurocognitive enhancement therapy. Schizophrenia Bulletin. 2013;39(SUPPL. 1):S281.

178. Bell MD. Cognitive training to enhance work program outcomes: Preliminary findings. Schizophrenia Bulletin. 2015;41(SUPPL. 1):S302.

179. Bell MD, Choi K-H, Dyer C, Wexler BE. Benefits of cognitive remediation and supported employment for schizophrenia patients with poor community functioning. Psychiatric Services (Washington, DC). 2014;65(4):469-75.

180. Bell MD, Corbera S, Wexler B. Cognitive remediation and supported employment: Moderators and mediators of vocational outcomes. Schizophrenia Bulletin. 2013;39(SUPPL. 1):S281-S2.

181. Bell MD, Corbera S, Wexler BE. Cognitive remediation and competitive employment: Differential benefits for schizophrenia patients with poor community function. Schizophrenia Research. 2012;136(SUPPL. 1):S78.

182. Bell MD, Laws H, Pittman B, Johannesen JK. Comparison of focused cognitive training and portable "brain-games" on functional outcomes for vocational rehabilitation participants. Scientific Reports. 2018;8(1):1779-.

183. Bennett K, Brady MP, Scott J, Dukes C, Frain M. The effects of covert audio coaching on the job performance of supported employees. Focus on Autism & Other Developmental Disabilities. 2010;25(3):173-85.

184. Bennett K, Brady MP, Scott J, Dukes C, Frain M, Baum BBBBBBBCCDGGHHJKKKKLLLMMOPPPRR. The effects of covert audio coaching on the job performance of supported employees. Focus on Autism and Other Developmental Disabilities. 2010;25(3):173-85.

185. Ben-Shalom Y, Mamun AA. Return-to-Work Outcomes Among Social Security Disability Insurance Program Beneficiaries. Journal of Disability Policy Studies. 2015;26(2):100-10.

186. Benstead S. Predicting policy performance: Can the Work and Health Programme work for chronically ill or disabled people? Critical Social Policy. 2019;39(4):643-62.

187. Berglund E, Anderzén I, Andersén Å, Carlsson L, Gustavsson C, Wallman T, et al. Multidisciplinary Intervention and Acceptance and Commitment Therapy for Return-to-Work and Increased Employability among Patients with Mental Illness and/or Chronic Pain: A Randomized Controlled Trial. International Journal Of Environmental Research And Public Health. 2018;15(11).

188. Bergmark M, Bejerholm U, Markstrom U. Implementation of evidence-based interventions: Analyzing critical components for sustainability in community mental health services. Social Work in Mental Health. 2018:No-Specified.

189. Bergmark M, Bejerholm U, Markström U. <italic>Critical Components in Implementing Evidence</italic>‐<italic>based Practice: A Multiple Case Study of Individual Placement and Support for People with Psychiatric Disabilities</italic>. Social Policy & Administration. 2018;52(3):790-808.

190. Bergmark M, Bejerholm U, Markström U. Implementation of evidence-based interventions: analyzing critical components for sustainability in community mental health services. Social Work in Mental Health. 2019;17(2):129-48.

191. Bergstrom G, Bergstrom C, Hagberg J, Bodin L, Jensen I, Aronsson ABBBBBBGHJJKMNRRSTTTTvdHvdHVK. A 7-year follow-up of multidisciplinary rehabilitation among chronic neck and back pain patients. Is sick leave outcome dependent on psychologically derived patient groups? European Journal of Pain. 2010;14(4):426-33.

192. Bernacchio C, McReynolds C, Falvo D, Stevens J, Cimera R, Hogan E, et al. Psychosocial characteristics of aging persons with psychiatric disabilities seeking VR. Special Issue: Psychiatric rehabilitation: Demographic subpopulations. 2009;40(2):5-12.

193. Berry C. Quantity over quality: a political economy of ‘active labour market policy’ in the UK. Policy Studies. 2014;35(6):592-610.

194. Berry HG, Caplan LJ, Benz BBDHHHHHRRRSWW. Employment and earnings growth among transition-age supplemental security income program participants. Journal of Disability Policy Studies. 2010;21(3):152-9.

195. Bertoni D. VA VOCATIONAL REHABILITATION AND EMPLOYMENT PROGRAM: Independent Living Services and Supports Require Stronger Oversight. GAO Reports. 2013:1-15.

196. Bertoni D. Improved Oversight of Independent Living Services and Supports Is Needed. GAO Reports. 2013:1-84.

197. Bertoni D. SUPPLEMENTAL SECURITY INCOME: SSA Could Strengthen Its Efforts to Encourage Employment for Transition-Age Youth. GAO Reports. 2017:1-53.

198. Bertram M. Mental health, social inclusion and the development of vocational services in the NHS – what can be learnt? Mental Health Review Journal. 2019;24(2):133-43.

199. Besse C, Poremski D, Laliberté V, Latimer E. Changes in the nature and intensity of stress following employment among people with severe mental illness receiving individual placement and support services: an exploratory qualitative study. Journal Of Mental Health (Abingdon, England). 2017;26(4):312-7.

200. Besse C, Poremski D, Laliberté V, Latimer E. The meaning and experience of stress among supported employment clients with mental health problems. Health & Social Care In The Community. 2018;26(3):383-92.

201. Besse C, Silva B, Dutoit M, Bonsack C. Individual placement and support (IPS) for people with mental illness: A retrospective naturalistic study. Soutien individuel a l'emploi (IPS) vaudois. 2016;167(7):215-21.

202. Betancourt OA, Herrera MM. Community rehabilitation of the schizophrenic patient. South African Journal of Psychiatry. 2010;16(3):84.

203. Bethge M. [Work-Related Medical Rehabilitation]. Die Rehabilitation. 2017;56(1):14-21.

204. Bethge M, Markus M, Streibelt M, Gerlich C, Schuler M. Effects of nationwide implementation of work-related medical rehabilitation in Germany: propensity score matched analysis. Occupational And Environmental Medicine. 2019;76(12):913-9.

205. Beveridge S, Leconte P, Shaine MD, Del Loro C, Penrod JC. Application of the Knowledge Validation Inventory-Revised to Assess Current Training Needs of State-Federal Rehabilitation Counselors. Rehabilitation Research, Policy & Education. 2015;29(3):241-60.

206. Beyer S. The impact of agency organisation and natural support on supported employment outcomes. Journal of Vocational Rehabilitation. 2012;36(2):109-19.

207. Beyer S. The progress towards integrated employment in the UK. Journal of Vocational Rehabilitation. 2012;37(3):185-94.

208. Beyer S, Andrews BBBBBBBBBBDDEF-JHHHJJKMMMMPRRRR. The progress towards integrated employment in the UK. Journal of Vocational Rehabilitation. 2012;37(3):185-94.

209. Beyer S, Brown T, Akandi R, Rapley M. A comparison of quality of life outcomes for people with intellectual disabilities in supported employment, day services and employment enterprises. Journal of Applied Research in Intellectual Disabilities. 2010;23(3):290-5.

210. Beyer S, de Borja Jordan de Urries F, Verdugo MA, Beyer BBBBBC-RGHJdUJdUJdUJdUKLMNOBPRS. A comparative study of the situation of supported employment in Europe. Journal of Policy and Practice in Intellectual Disabilities. 2010;7(2):130-6.

211. Beyer S, Jordán de Urríes FBJ, Verdugo MA. A comparative study of the situation of supported employment in Europe. Journal of Policy & Practice in Intellectual Disabilities. 2010;7(2):130-6.

212. Beyer S, Kaehne A, Gould S, Carr J. Outcomes and cost: Benefit of transition from college to employment: The ROSE Project. Journal of Intellectual Disability Research. 2012;56(7-8):713.

213. Beyer S, Lynch M, Vigna E, Meek A. Supported employment for people with intellectual disabilities and autism spectrum conditions: What's working? Journal of Intellectual Disability Research. 2019;63(7):638.

214. Beyer S, Meek A, Davies A. Supported work experience: Job coach and employer assessments of worker performance. Journal of Intellectual Disability Research. 2016;60(7-8):637.

215. Beyer S, Meek A, Davies A, Baer BBBBBBCCCDGHHHHHHHKKKLMMMPPRRR. Supported work experience and its impact on young people with intellectual disabilities, their families and employers. Advances in Mental Health and Intellectual Disabilities. 2016;10(3):207-20.

216. Beyer S, Pozner DMMMMRBKMTFKSRWCCVBBBBBBLR. The impact of agency organisation and natural support on supported employment outcomes. Journal of Vocational Rehabilitation. 2012;36(2):109-19.

217. Beyer S, Vigna E, Meek A. Employment outcomes from different patterns of job coach inputs- the experience of engage to change. Journal of Intellectual Disability Research. 2019;63(7):855.

218. Bhat CS. Assisting unemployed adults find suitable work: a group intervention embedded in community and grounded in social action. Journal for Specialists in Group Work. 2010;35(3):246-54.

219. Biegel DE, Beimers D, Stevenson LD, Ronis RJ, Boyle P. Predictors of referral to Supported Employment among consumers with co-occurring mental and substance use disorders. Community Mental Health Journal. 2009;45(6):427-38.

220. Biegel DE, Kola LA, Meeks D, Stevenson L, Beimers D. A university-agency mental health research collaboration: a case example. Care Management Journals: Journal Of Case Management ; The Journal Of Long Term Home Health Care. 2010;11(2):83-90.

221. Biegel DE, Stevenson LD, Beimers D, Ronis RJ, Boyle P. Predictors of competitive employment among consumers with co-occurring mental and substance use disorders. Research on Social Work Practice. 2010;20(2):191-201.

222. Bigby C, Wilson NJ, Balandin S, Stancliffe RJ. Disconnected expectations: staff, family, and supported employee perspectives about retirement. Journal Of Intellectual & Developmental Disability. 2011;36(3):167-74.

223. Biggs D, Hovey N, Tyson PJ, MacDonald S. Employer and employment agency attitudes towards employing individuals with mental health needs. Journal Of Mental Health (Abingdon, England). 2010;19(6):505-16.

224. Biljon Hv, Casteleijn D, Toit SHJd, Rabothata S. An Action Research Approach to Profile an Occupational Therapy Vocational Rehabilitation Service in Public Healthcare. South African Journal of Occupational Therapy. 2015;45(3):40-7.

225. Bio DS, Gattaz WF, Adad ABBBBBBBBCEFFGGGGGHHHKKKLLLMMM. Vocational rehabilitation improves cognition and negative symptoms in schizophrenia. Schizophrenia Research. 2011;126(1-3):265-9.

226. Bio DS, Gattaz WF, Bio DS, Gattaz WF. Vocational rehabilitation improves cognition and negative symptoms in schizophrenia. Schizophrenia Research. 2011;126(1-3):265-9.

227. Björk Brämberg E, Holmgren K, Bültmann U, Gyllensten H, Hagberg J, Sandman L, et al. Increasing return-to-work among people on sick leave due to common mental disorders: design of a cluster-randomized controlled trial of a problem-solving intervention versus care-as-usual conducted in the Swedish primary health care system (PROSA). BMC Public Health. 2018;18(1):889-.

228. Björkdahl A. The return to work after a neuropsychological programme and prognostic factors for success. Brain Injury. 2010;24(9):1061-9.

229. Bjorkdahl A, Benton BBBBBCCDDFFFGHHIJKK-MKKKLLMMM-CO. The return to work after a neuropsychological programme and prognostic factors for success. Brain Injury. 2010;24(9):1061-9.

230. Bjørkedal STB, Eplov LF, Møller T. The missing link-participants' perspectives on transfer from psychosocial interventional contexts to everyday community life: a qualitative synthesis of interventional studies. BMC Psychology. 2021;9(1):62.

231. Blamires K. A summary of government initiatives relating to employment for people with learning disabilities in England. Tizard Learning Disability Review. 2015;20(3):151-65.

232. Blanc A, Blanc BB-LCDEEFHPST. The professional insertion of the workers handicapped in France: Principles and realities. L'insertion professionnelle des travailleurs handicapes en france : Principes et realites. 2010;16(1):3-19.

233. Blank A, Harries P, Reynolds F. Mental health service users' perspectives of work: a review of the literature. British Journal of Occupational Therapy. 2011;74(4):191-9.

234. Blank A, Hayward M. The role of work in recovery. British Journal of Occupational Therapy. 2009;72(7):324-6.

235. Blick RN, Litz KS, Thornhill MG, Goreczny AJ. Do inclusive work environments matter? Effects of community-integrated employment on quality of life for individuals with intellectual disabilities. Research In Developmental Disabilities. 2016;53-54:358-66.

236. Blick RN, Litz KS, Thornhill MG, Goreczny AJ. Do inclusive work environments matter? Effects of community-integrated employment on quality of life for individuals with intellectual disabilities. Research in Developmental Disabilities. 2016;53:358-66.

237. Blonk L, Huijben T, Bredewold F, Tonkens E. Balancing care and work: A case study of recognition in a social enterprise. Journal of Intellectual Disability Research. 2019;63(7):717-8.

238. Bloom J, Dorsett P, McLennan V. Occupational bonding after spinal cord injury: A review and narrative synthesis. Journal of Vocational Rehabilitation. 2019;50(1):109-20.

239. Bloom J, Dorsett P, McLennan V. Investigating employment following spinal cord injury: outcomes, methods, and population demographics. Disability & Rehabilitation. 2019;41(20):2359-68.

240. Bloom J, McLennan V, Dorsett P, Bloom BBCCCC-HCCDDEFFGGGHH-SHHHJJKKKKK. Occupational bonding after spinal cord injury: A review and narrative synthesis. Journal of Vocational Rehabilitation. 2019;50(1):109-20.

241. Boardman J, Beard BBBBBBBBBBBBBBBBCCCCCDDFFGHHHK. Long-term mental health care for people with severe mental disorders - The importance of workplace rehabilitation. Die Psychiatrie: Grundlagen & Perspektiven. 2013;10(2):87-94.

242. Boardman J, Rinaldi M. Difficulties in implementing supported employment for people with severe mental health problems. The British Journal Of Psychiatry: The Journal Of Mental Science. 2013;203(3):247-9.

243. Boeltzig H. State Vocational Rehabilitation Counselors' Perceptions of Internet Use in VR, Types of Internet Applications Used, and Types of Rehabilitation Activities Conducted Online. Journal of Rehabilitation. 2011;77(4):23-30.

244. Boeltzig H, Pilling D, Timmons JC, Johnson R. Disability specialist staff in US one-stop career centers and British Jobcentre Plus offices: roles, responsibilities, and evidence of their effectiveness. Journal of Disability Policy Studies. 2010;21(2):101-15.

245. Boeltzig H, Timmons JC, Butterworth J. Gender differences in employment outcomes of individuals with developmental disabilities. Journal of Vocational Rehabilitation. 2009;31(1):29-38.

246. Boeltzig-Brown H, Fleming AR, Heyman M, Gauthier M, Cully J, Foley SM. A Systematic Review of State Vocational Rehabilitation Agency-Based Literature. Rehabilitation Research, Policy & Education. 2017;31(4):352-71.

247. Boeltzig-Brown H, Sashida C, Nagase O, Kiernan WE, Foley SM. The vocational rehabilitation service system in Japan. Journal of Vocational Rehabilitation. 2013;38(3):169-83.

248. Bogenschutz MD, Hewitt A, Nord D, Hepperlen R. Direct support workforce supporting individuals with IDD: current wages, benefits, and stability. Intellectual And Developmental Disabilities. 2014;52(5):317-29.

249. Bohm M, Stiglbauer B. Psychosocial vocational rehabilitation in a world of work 4.0-Between demands and needs. Neuropsychiatrie. 2019.

250. Bohman TM, Wallisch L, Christensen K, Stoner D, Pittman A, Reed B, et al. Working Well - The Texas Demonstration to Maintain Independence and Employment: 18-month outcomes. Journal of Vocational Rehabilitation. 2011;34(2):97-106.

251. Bolo C, Sareen J, Patten S, Schmitz N, Currie S, Wang J. Receiving workplace mental health accommodations and the outcome of mental disorders in employees with a depressive and/or anxiety disorder. Journal Of Occupational And Environmental Medicine. 2013;55(11):1293-9.

252. Bond G, Drake RE, Campbell K. The effectiveness of the individual placement and support model of supported employment for young adults: Results from four randomized controlled trials. Early Intervention in Psychiatry. 2012;6(SUPPL.1):30.

253. Bond GR, Becker DR, Drake RE, Aarons BBBBBBBBBBBBBBBBBBBBBBBBBBBBBB. Measurement of fidelity of implementation of evidence-based practices: Case example of the IPS Fidelity Scale. Clinical Psychology: Science and Practice. 2011;18(2):126-41.

254. Bond GR, Campbell K, Becker DR. A test of the occupational matching hypothesis for rehabilitation clients with severe mental illness. Journal Of Occupational Rehabilitation. 2013;23(2):261-9.

255. Bond GR, Campbell K, Drake RE. Standardizing measures in four domains of employment outcomes for individual placement and support. Psychiatric Services (Washington, DC). 2012;63(8):751-7.

256. Bond GR, Drake RE. Making the case for IPS supported employment. Administration And Policy In Mental Health. 2014;41(1):69-73.

257. Bond GR, Drake RE, Bailey BBBBBBBBBBBBBBBBBCCCCDDDDDDDEF. Making the case for IPS supported employment. Administration and Policy in Mental Health and Mental Health Services Research. 2014;41(1):69-73.

258. Bond GR, Drake RE, Becker DR. Beyond evidence-based practice: nine ideal features of a mental health intervention. Research on Social Work Practice. 2010;20(5):493-501.

259. Bond GR, Drake RE, Becker DR. Implementation of IPS supported employment around the world: Planned vs. unplanned dissemination. A commentary on Menear et al. Social Science & Medicine. 2011;72(7):1036-8.

260. Bond GR, Drake RE, Becker DR. Generalizability of the Individual Placement and Support (IPS) model of supported employment outside the US. World Psychiatry: Official Journal Of The World Psychiatric Association (WPA). 2012;11(1):32-9.

261. Bond GR, Drake RE, Becker DR. An update on Individual Placement and Support. World psychiatry : official journal of the World Psychiatric Association (WPA). 2020;19(3):390-1.

262. Bond GR, Drake RE, Becker DR, Anthony ABBBBBBBBBBBBBBBBBBBBBBBBBBBBB. Beyond evidence-based practice: Nine ideal features of a mental health intervention. Research on Social Work Practice. 2010;20(5):493-501.

263. Bond GR, Drake RE, Becker DR, Noel V. Sustaining Individual Placement and Support (IPS) services: the IPS Learning Community. World Psychiatry: Official Journal Of The World Psychiatric Association (WPA). 2016;15(1):81-3.

264. Bond GR, Drake RE, Becker DR, Noel VA. The IPS Learning Community: A Longitudinal Study of Sustainment, Quality, and Outcome. Psychiatric Services (Washington, DC). 2016;67(8):864-9.

265. Bond GR, Drake RE, Campbell K. Effectiveness of individual placement and support supported employment for young adults. Early Intervention In Psychiatry. 2016;10(4):300-7.

266. Bond GR, Drake RE, Luciano A. Employment and educational outcomes in early intervention programmes for early psychosis: A systematic review. Epidemiology and Psychiatric Sciences. 2014;1673(1).

267. Bond GR, Drake RE, Luciano A. Employment and educational outcomes in early intervention programmes for early psychosis: A systematic review. Epidemiology and Psychiatric Sciences. 2014;24(5):446-57.

268. Bond GR, Drake RE, Luciano A. Employment and educational outcomes in early intervention programmes for early psychosis: a systematic review. Epidemiology And Psychiatric Sciences. 2015;24(5):446-57.

269. Bond GR, Drake RE, Luciano A, Abdel-Baki AABBBBBBBBBBB-MBCCCCCCDDDDEFFF. Employment and educational outcomes in early intervention programmes for early psychosis: A systematic review. Epidemiology and Psychiatric Sciences. 2015;24(5):446-57.

270. Bond GR, Drake RE, Pogue JA. Expanding Individual Placement and Support to Populations With Conditions and Disorders Other Than Serious Mental Illness. Psychiatric Services (Washington, DC). 2019;70(6):488-98.

271. Bond GR, Drake RE, Pogue JA. Facilitating Competitive Employment for People with Disabilities: Expanding Implementation of IPS Supported Employment to New Populations. Handbook of Disability, Work and Health. 2020:1-18.

272. Bond GR, Drake RE, Skinner JS, Goldman HH. Economic impact of supported employment for early psychosis on US social security disability programs. Early Intervention in Psychiatry. 2010;4(SUPPL. 1):5.

273. Bond GR, Johnson-Kwochka AV, Becker DR, Drake RE, Greene MA. Sustaining and Expanding Evidence-Based Supported Employment: The Role of State Leaders Participating in a Learning Community. Administration And Policy In Mental Health. 2017;44(3):320-30.

274. Bond GR, Johnson-Kwochka AV, Pogue JA, Langfitt Reese S, Becker DR, Drake RE. A Tale of Four States: Factors Influencing the Statewide Adoption of IPS. Administration And Policy In Mental Health. 2021;48(3):528-38.

275. Bond GR, Johnson-Kwochka AV, Pogue JA, Langfitt Reese S, Becker DR, Drake RE, et al. A tale of four states: Factors influencing the statewide adoption of ips. Administration and Policy in Mental Health and Mental Health Services Research. 2020:No-Specified.

276. Bond GR, Kim SJ, Becker DR, Swanson SJ, Drake RE, Krzos IM, et al. A Controlled Trial of Supported Employment for People With Severe Mental Illness and Justice Involvement. Psychiatric Services (Washington, DC). 2015;66(10):1027-34.

277. Bond GR, Kukla M. Impact of follow-along support on job tenure in the individual placement and support model. The Journal Of Nervous And Mental Disease. 2011;199(3):150-5.

278. Bond GR, Kukla M. Is job tenure brief in individual placement and support (IPS) employment programs? Psychiatric Services (Washington, DC). 2011;62(8):950-3.

279. Bond GR, Kukla M, Anderson BBBBBBBBBBBCHJLM-WMMMMMMRSSTWZ. Impact of follow-along support on job tenure in the individual placement and support model. Journal of Nervous and Mental Disease. 2011;199(3):150-5.

280. Bond GR, Lockett H, van Weeghel J. International growth of individual placement and support. Epidemiology And Psychiatric Sciences. 2020;29:e183.

281. Bond GR, Peterson AE, Becker DR, Drake RE. Validation of the Revised Individual Placement and Support Fidelity Scale (IPS-25). Psychiatric Services (Washington, DC). 2012;63(8):758-63.

282. Bonfils IS, Hansen H, Dalum HS, Eplov LF. Implementation of the individual placement and support approach – facilitators and barriers. Scandinavian Journal of Disability Research. 2017;19(4):318-33.

283. Bönisch A, Dorn M, Ehlebracht-König I. 'Vocational Perspective' - Short-Term Efficacy of a Group Treatment for Patients with Extensive Work-Related Problems during Medical Rehabilitation]. Rehabilitation. 2012;51(1):39-51.

284. Borger C, Marrow J, Drake RE, Taylor J. Characteristics of Enrollees in the Supported Employment Demonstration. Psychiatric Services (Washington, DC). 2021:appips202000826.

285. Boutin DL. Occupational outcomes for vocational rehabilitation consumers with hearing impairments. Journal of Rehabilitation. 2010;76(3):40-6.

286. Boutin DL. Where vocational rehabilitation consumers work according to the standard occupational classification system. Journal of Rehabilitation. 2010;76(3):32-9.

287. Boutin DL, Accordino MP. Importance of Collegiate Training for Vocational Rehabilitation Consumers with Psychiatric Disabilities. American Journal of Psychiatric Rehabilitation. 2011;14(1):76-95.

288. Boutin DL, Accordino MP, Agresti BBBBCCCCDF-NHHHKLLLLMMMMMMMMMMN. Exploring postsecondary education and competitive employment for people with mental illness. Special Issue: Psychiatric rehabilitation: Demographic subpopulations. 2009;40(2):13-21.

289. Boutin DL, Agresti BBBBBBCCCDDFGGHHHKLMMMMMMMMNOR. Effective vocational rehabilitation services for military veterans. Journal of Applied Rehabilitation Counseling. 2011;42(2):24-32.

290. Boutin DL, Agresti BCCCCF-NGHHHHKLMMMMMMMMNRRSSSTW. The impact of college training and vocational rehabilitation services on employment for consumers with hearing loss. Journal of the American Deafness and Rehabilitation Association. 2009;42(2):73-89.

291. Boutin DL, Wilson K. An analysis of vocational rehabilitation services for consumers with hearing impairments who received college or university training. Rehabilitation Counseling Bulletin. 2009;52(3):156-66.

292. Boutin DL, Wilson K, Agresti BBBCCCF-NHHHHK-RMMMMMMMMMMMMMNNN. An analysis of vocational rehabilitation services for consumers with hearing impairments who received college or university training. Rehabilitation Counseling Bulletin. 2009;52(3):156-66.

293. Boutin DL, Wilson KB. Professional jobs and hearing loss: a comparison of deaf and hard of hearing consumers. Journal of Rehabilitation. 2009;75(1):36-40.

294. Bowie CR, Grossman M, Gupta M, Holshausen K, Best MW. Action-Based Cognitive Remediation for Individuals With Serious Mental Illnesses: Effects of Real-World Simulations and Goal Setting on Functional and Vocational Outcomes. Psychiatric Rehabilitation Journal. 2017;40(1):53-60.

295. Boycott N, Akhtar A, Schneider J. "Work is good for me": views of mental health service users seeking work during the UK recession, a qualitative analysis. Journal Of Mental Health (Abingdon, England). 2015;24(2):93-7.

296. Boycott N, Schneider J, McMurran M. Additional interventions to enhance the effectiveness of individual placement and support: a rapid evidence assessment. Rehabilitation Research And Practice. 2012;2012:382420-.

297. Boycott N, Schneider J, McMurran M. Evaluation of a cognitive-behavioural intervention augmenting individual placement and support. Mental Health & Social Inclusion. 2016;20(2):119-25.

298. Boycott N, Schneider J, Osborne M. Creating a culture of employability in mental health. Mental Health & Social Inclusion. 2014;18(1):29-34.

299. Boyle CL, Nott MT, Baguley IJ, Ranka JL. Contextual influences on employment of people with dual diagnosis: spinal cord injury and traumatic brain injury. Australian Occupational Therapy Journal. 2014;61(5):335-43.

300. Bradley CF, Ebener DJ, Geyer PD. Contributors to Successful VR Outcomes among Non-Latino (Caucasian) and Latino Consumers with Hearing Loss. Journal of Rehabilitation. 2013;79(2):24-33.

301. Brady MP, Frain M, Duffy ML, Bucholz J. Evaluating work performance and support needs in supported employment training programs: correspondence between teachers' ratings and students' self ratings? Journal of Rehabilitation. 2010;76(3):24-31.

302. Brantschen E, Kawohl W, Rössler W, Bärtsch B, Nordt C. Supported employment - improving competitive employment for people with mental illness: The role of motivation and social network. Journal of Vocational Rehabilitation. 2014;40(1):41-7.

303. Brantschen E, Landolt K, Kawohl W, Rössler W, Bärtsch B, Nordt C. Two types of expectancies concerning competitive employment among people with mental illness in supported employment. Journal of Vocational Rehabilitation. 2017;46(2):195-202.

304. Brasure M, Lamberty GJ, Sayer NA, Nelson NW, MacDonald R, Ouellette J, et al. Participation After Multidisciplinary Rehabilitation for Moderate to Severe Traumatic Brain Injury in Adults: A Systematic Review. Archives of Physical Medicine & Rehabilitation. 2013;94(7):1398-420.

305. Brattig V, Antonovsky BBBEFHHLNNNRRRSSSSSZ. Inclusion instead of integration in vocational rehabilitation with particular attention to (young) people with psychological problems. Inklusion statt Integration in der beruflichen Rehabilitation? Unter vorrangiger Beachtung von (jungen) Menschen mit psychischen Problemen. 2012;44(4):837-49.

306. Brenninkmeijer V, Blonk RWB. The effectiveness of the JOBS program among the long-term unemployed: a randomized experiment in the Netherlands. Health Promotion International. 2012;27(2):220-9.

307. Brewer D, Erickson W, Karpur A, Unger D, Sukyeong P, Malzer V. Evaluation of a Multi-site Transition to Adulthood Program for Youth with Disabilities. Journal of Rehabilitation. 2011;77(3):3-13.

308. Brewin D, Papageorgis S, Collins K. Reducing clients' reliance on long-term sickness benefits. Mental Health Practice. 2011;15(4):20-5.

309. Brice JGH, Swarbrick MA, Gill KJ. Promoting the Wellness of Peer Providers Through Coaching. Journal of Psychosocial Nursing & Mental Health Services. 2014;52(1):41-5.

310. Brickham D, Jeong Han K, Gonzalez R, Rosenthal D. Vocational outcomes of people with alcohol abuse/dependence who received state vocational rehabilitation services. Journal of Vocational Rehabilitation. 2016;45(3):267-79.

311. Brieger P, Hoffmann H. [How can the mentally ill achieve sustained employment? Supported employment versus pre-vocational training]. Der Nervenarzt. 2012;83(7):840-6.

312. Brieger P, Hoffmann H, Angermeyer BBBBBBBBCCCCHHHHHJKMRSTWWW. How can the mentally ill achieve sustained employment? Supported employment versus pre-vocational training. Was bringt psychisch kranke nachhaltig in arbeit?: supported employment" vs pre-vocational training". 2012;83(7):840-6.

313. Briem N, Jonasson H, Aevarsdottir H, Jonsdottir H. Individual placement and support in ICELAND: Collaboration between landspitali university hospital and VIRK vocational rehabilitation fund. Early Intervention in Psychiatry. 2018;12(Supplement 1):18.

314. Briem N, Olafsson M, Viisdottir HF. Early Intervention in Psychosis: The Icelandic model. Early Intervention in Psychiatry. 2014;8(SUPPL. 1):140.

315. Brinchmann B, Lorentsen O. Early intervention: Integrating vocational and medical rehabilitation during admittance in a mental health institution. A study in a rural part of North-Norway. Early Intervention in Psychiatry. 2012;6(SUPPL.1):111.

316. Brinchmann B, Widding-Havneraas T, Modini M, Rinaldi M, Moe CF, McDaid D, et al. A meta-regression of the impact of policy on the efficacy of individual placement and support. Acta Psychiatrica Scandinavica. 2019.

317. Brinchmann B, Widding-Havneraas T, Modini M, Rinaldi M, Moe CF, McDaid D, et al. A meta-regression of the impact of policy on the efficacy of individual placement and support. Acta Psychiatrica Scandinavica. 2020;141(3):206-20.

318. Brock ME, Cannella-Malone HI, Schaefer JM, Page EJ, Andzik NR, Seaman RL. Efficacy of training job coaches to implement evidence-based instructional strategies. Journal of Vocational Rehabilitation. 2016;45(3):351-64.

319. Bronowski P, Sawicka M, Rozya P. Who are shelter housing users and what do they expect? Postepy Psychiatrii i Neurologii. 2016;25(1):32-41.

320. Brooke V, Brooke AM, Schall C, Wehman P, McDonough J, Thompson K, et al. Employees with Autism Spectrum Disorder Achieving Long-Term Employment Success: A Retrospective Review of Employment Retention and Intervention. Research & Practice for Persons with Severe Disabilities. 2018;43(3):181-93.

321. Broulíková HM, Winkler P, Páv M, Kondrátová L. Costs of Mental Health Services in Czechia: Facilitating an Evidence-Based Reform of Psychiatric Care. Applied Health Economics And Health Policy. 2019.

322. Broulíková HM, Winkler P, Páv M, Kondrátová L. Costs of Mental Health Services in Czechia: Facilitating an Evidence-Based Reform of Psychiatric Care. Applied Health Economics And Health Policy. 2020;18(2):287-98.

323. Brown J, Katikireddi SV, Leyland AH, McQuaid RW, Frank J, Macdonald EB. Age, health and other factors associated with return to work for those engaging with a welfare-to-work initiative: a cohort study of administrative data from the UK's Work Programme. BMJ Open. 2018;8(10):e024938-e.

324. Brown J, Neary J, Katikireddi SV, Thomson H, McQuaid RW, Leyland AH, et al. Protocol for a mixed-methods longitudinal study to identify factors influencing return to work in the over 50s participating in the UK Work Programme: Supporting Older People into Employment (SOPIE). BMJ Open. 2015;5(12):e010525-e.

325. Brown M, Mihelicova M, Collins K, Ponce A, Blank BBBBBCCCDHKKLMMPPPRST. Predictors of employment outcomes in a comprehensive service program for individuals experiencing chronic homelessness. American Journal of Orthopsychiatry. 2019;89(2):279-86.

326. Brown P, Johnson K. Aging with a disability and state vocational rehabilitation services. Work (Reading, Mass). 2014;48(3):441-51.

327. Brown P, Johnson K, Adkins ABCCDEFFGGJJJJKKKKKLMMMMMMMMNO. Aging with a disability and state vocational rehabilitation services. Work: Journal of Prevention, Assessment & Rehabilitation. 2014;48(3):441-51.

328. Browne DJ, Stephenson A, Wright J, Waghorn G. Developing high performing employment services for people with mental illness...including commentary by Sherring J. International Journal of Therapy & Rehabilitation. 2009;16(9):502-11.

329. Browne DJ, Waghorn G. Employment services as an early intervention for young people with mental illness. Early Intervention In Psychiatry. 2010;4(4):327-35.

330. Brucker DL, Botticello A, O'Neill J, Kutlik A. Variations in social capital among vocational rehabilitation applicants. Journal of Vocational Rehabilitation. 2017;46(2):187-94.

331. Brunette MF. Facilitators and barriers to implementation of coordinated specialty care in u.s. community mental health clinic. Schizophrenia Bulletin. 2015;41(SUPPL. 1):S304.

332. Bua-Iam P, Bias TK. Economic Impacts of West Virginia Division of Rehabilitation Services on Consumers with Significant Disabilities: Realistic Return-on-Investment Models for State-Federal VR Programs. Journal of Rehabilitation. 2011;77(3):25-30.

333. Buizza C, Pioli R, Lecchi S, Bonetto C, Bartoli A, Taglietti R, et al. Mental disorders and work integration: A retrospective study in a northern Italian town. Clinical Practice and Epidemiology in Mental Health. 2014;10.

334. Bull H, Lystad JU, Auerbach BBBBBBBCCCCDDE-KFGGHHHHKLLLLLLM. The importance of work for people with schizophrenia. Betydningen av arbeid for personer med schizofreni. 2011;48(8):733-8.

335. Bull H, Ueland T, Lystad J, Evensen S, Falkum E, Gaebel W, et al. The impact of apathy on vocational outcome on vocational rehabiltation. European Archives of Psychiatry and Clinical Neuroscience. 2015;265(1 SUPPL. 1):S99.

336. Bull H, Ueland T, Lystad JU, Evensen S, Falkum E. Can work history at baseline predict work behavior with in the first four weeks of vocational rehabilitation? Schizophrenia Research. 2014;153(SUPPL. 1):S279-S80.

337. Bull H, Ueland T, Lystad JU, Evensen S, Friis S, Martinsen EW, et al. Validation of the Work Behavior Inventory. Nordic Journal of Psychiatry. 2015;69(4):300-6.

338. Bull H, Ueland T, Ullevoldsæter Lystad J, Evensen S, Wilhelm Martinsen E, Falkum E, et al. Vocational Functioning in Schizophrenia Spectrum Disorders: Does Apathy Matter? Journal of Nervous & Mental Disease. 2016;204(8):599-605.

339. Bull H, Ueland T, Ullevoldsaeter Lystad J, Falkum E. Can work history at baseline predict work behavior within the first 4 weeks of vocational rehabilitation? European Archives of Psychiatry and Clinical Neuroscience. 2013;263(1 SUPPL. 1):S98.

340. Burcea ŞG. The Social Enterprise - Viable Mechanism of Social Integration for Romanian Vulnerable Groups. Review of International Comparative Management / Revista de Management Comparat International. 2014;15(5):613-25.

341. Burgess J, Yeeles K, Burns T. Refining Individual Placement and Support (IPS) and establishing its effectiveness: A pragmatic, non-inferiority RCT (IPS-LITE trial). Psychiatrische Praxis. 2011;38(SUPPL. 1).

342. Burgess S, Cimera RE. Employment outcomes of transition-aged adults with autism spectrum disorders: a state of the States report. American Journal on Intellectual & Developmental Disabilities. 2014;119(1):64-83.

343. Burke-Miller J, Razzano LA, Grey DD, Blyler CR, Cook JA. Supported employment outcomes for transition age youth and young adults. Psychiatric Rehabilitation Journal. 2012;35(3):171-9.

344. Burkhardt E, Leopold K, Laier S, Jackel D, Kallenbach M, Muller H, et al. What to do before it starts? FRITZ-The early intervention and therapy centre in Berlin. Was tun, bevor es losgeht? FRITZ-Das Fruhinterventions- und Therapiezentrum in Berlin. 2017;65(2):105-12.

345. Burns T, Catty J, White S, Becker T, Koletsi M, Fioritti A, et al. The impact of supported employment and working on clinical and social functioning: results of an international study of individual placement and support. Schizophrenia Bulletin. 2009;35(5):949-58.

346. Burns T, Yeeles K, Langford O, Montes MV, Burgess J, Anderson C. A randomised controlled trial of time-limited individual placement and support: IPS-LITE trial. The British Journal Of Psychiatry: The Journal Of Mental Science. 2015;207(4):351-6.

347. Burstrom B, Nylen L, Clayton S, Whitehead M. How equitable is vocational rehabilitation in Sweden? A review of evidence on the implementation of a national policy framework. Disability And Rehabilitation. 2011;33(6):453-66.

348. Burt MR. Impact of housing and work supports on outcomes for chronically homeless adults with mental illness: LA's HOPE. Psychiatric Services (Washington, DC). 2012;63(3):209-15.

349. Burton CZ, Vella L, Twamley EW. Prospective memory, level of disability, and return to work in severe mental illness. The Clinical Neuropsychologist. 2019;33(3):594-605.

350. Burton JD. Moving towards value-based supported employment programs. Psychiatric Rehabilitation Journal. 2009;32(4):257-8.

351. Burton JD, Drake RE, Bond GR. Moving towards value-supported employment programs...Bond GR et al. Implementing supported employment as an evidence-based practice. Psychiatric Services 2001;52(3):313-322. Psychiatric Rehabilitation Journal. 2009;32(4):257-60.

352. Butler G, Howard L, Choi S, Thornicroft G, Barkow BB-MBCCCDEGHHKMMMNSVDWW. Characteristics of people with severe mental illness who obtain employment. The Psychiatrist. 2010;34(2):47-50.

353. Butterworth J, Hiersteiner D, Engler J, Bershadsky J, Bradley V. National Core Indicators©: Data on the current state of employment of adults with IDD and suggestions for policy development. Journal of Vocational Rehabilitation. 2015;42(3):209-20.

354. Butterworth J, Migliore A, Nord D, Gelb A. Improving the Employment Outcomes of Job Seekers with Intellectual and Developmental Disabilities: A Training and Mentoring Intervention for Employment Consultants. Journal of Rehabilitation. 2012;78(2):20-9.

355. Butterworth J, Migliore A, Nye-Lengerman K, Lyons O, Gunty A, Eastman J, et al. Using data-enabled performance feedback and guidance to assist employment consultants in their work with job seekers: An experimental study. Journal of Vocational Rehabilitation. 2020;53(2):189-203.

356. Buys N, Matthews LR, Randall C. Contemporary vocational rehabilitation in Australia. Disability And Rehabilitation. 2015;37(9):820-4.

357. Caldwell K, Parker Harris S, Renko M. Inclusive management for social entrepreneurs with intellectual disabilities: "how they act". Journal Of Applied Research In Intellectual Disabilities: JARID. 2019.

358. Calkins M, Schmidt L, McCurry L, Patton B, Tang S, Moberg P, et al. F130. SAMHSA SUPPORTED COORDINATED SPECIALTY CARE FOR FIRST-EPISODE PSYCHOSIS: THE PENN PSYCHOSIS EVALUATION AND RECOVERY CENTER (PERC)...2019 Congress of the Schizophrenia International Research Society, 10-14 April 2019, Orlando, Florida. Schizophrenia Bulletin. 2019;45:S303-S.

359. Calkins M, Schmidt L, McCurry LT, Patton B, Tang S, Moberg P, et al. Samhsa supported coordinated specialty care for first-episode psychosis: The penn psychosis evaluation and recovery center (PERC). Schizophrenia Bulletin. 2019;45(Supplement 2):S303.

360. Calkins M, Schmidt L, Patton B, Franco O, Huque Z, Payne E, et al. Further Evidence Supporting Coordinated Specialty Care (CSC) for Early Psychosis: Findings From the SAMHSA Supported Penn Psychosis Evaluation and Recovery Center (PERC). Biological Psychiatry. 2020;87(9 Supplement):S291.

361. Calkins M, Westfall M, Hurford I. Pennsylvania first-episode program evaluation of coordinated specialty care: Six-and 12-month outcomes. Schizophrenia Bulletin. 2020;46(Supplement 1):S228-S9.

362. Campbell K. Employment rates for people with severe mental illness in the UK not improved by 1 year's individual placement and support. Evidence-Based Mental Health. 2010;13(4):114-.

363. Campbell K, Bond GR, Drake RE. Who benefits from supported employment: a meta-analytic study. Schizophrenia Bulletin. 2011;37(2):370-80.

364. Campbell K, Bond GR, Drake RE, McHugo GJ, Xie H. Client predictors of employment outcomes in high-fidelity supported employment: a regression analysis. The Journal Of Nervous And Mental Disease. 2010;198(8):556-63.

365. Canady VA. Illinois employment model serves as an example for others. Mental Health Weekly. 2020;30(44):6-7.

366. Canal P, Cruz RM, Bartilotti BBBBCDEGGHHHHLLMMMPRRRR-PSSSSSS. Psychological aspects and vocational rehabilitation: A literature review. Aspectos psicologicos e reabilitacao profissional: Revisao de literatura. 2013;30(4):593-601.

367. Cantora A, Berman BCCCCDFGHHLVLLLLLLLMM-KNOBOBPPR. Navigating the job search after incarceration: The experiences of work-release participants. Criminal Justice Studies: A Critical Journal of Crime, Law & Society. 2015;28(2):141-60.

368. Capella McDonnall M, Crudden A. Building Relationships with Businesses: Recommendations from Employers Concerning Persons who are Blind/Visually Impaired. Journal of Rehabilitation. 2015;81(3):43-50.

369. Cappa C, Castignoli V, Gazzola S, Pedrini P, Scottini P, Zorza V, et al. Application of IPS within the STAND-UP Program for the early treatment of onset of psychosis in DSM-DP Ausl in Piacenza. Early Intervention in Psychiatry. 2016;10(Supplement 1):248.

370. Cappa C, Figoli M, Rossi P. Network of services facilitating and supporting job placement for people with autism spectrum disorders. The experience of the ASL Piacenza, Italy. Annali dell'Istituto superiore di sanita. 2020;56(2):241-6.

371. Carlier BE, Schuring M, Burdorf A. Influence of an Interdisciplinary Re-employment Programme Among Unemployed Persons with Mental Health Problems on Health, Social Participation and Paid Employment. Journal of Occupational Rehabilitation. 2018;28(1):147-57.

372. Carlos Arango-Lasprilla J, da Silva Cardoso E, Wilson LM, Romero MG, Fong C, Sung C. Vocational Rehabilitation Service Patterns and Employment Outcomes for Hispanics with Spinal Cord Injuries. Rehabilitation Research, Policy & Education. 2011;25(3/4):149-62.

373. Carlson KF, Pogoda TK, Gilbert TA, Resnick SG, Twamley EW, O'Neil ME, et al. Supported Employment for Veterans With Traumatic Brain Injury: Patient Perspectives. Archives Of Physical Medicine And Rehabilitation. 2018;99(2S):S4-S13.e1.

374. Carlson L, Rapp CA, Eichler MS. The experts rate: supervisory behaviors that impact the implementation of evidence-based practices. Community Mental Health Journal. 2012;48(2):179-86.

375. Carlson L, Smith G, Mariscal ES, Rapp CA, Holter MC, Ko E, et al. The comparative effectiveness of a model of job development versus treatment as usual. Best Practices in Mental Health: An International Journal. 2018;14(2):21-31.

376. Carlson SR, Morningstar ME, Munandar V. Workplace supports for employees with intellectual disability: A systematic review of the intervention literature. Journal of Vocational Rehabilitation. 2020;52(3):251-65.

377. Carmona VR, Gómez-Benito J, Huedo-Medina TB, Rojo JE. Employment outcomes for people with schizophrenia spectrum disorder: A meta-analysis of randomized controlled trials. International Journal Of Occupational Medicine And Environmental Health. 2017;30(3):345-66.

378. Carmona VR, Gómez-Benito J, Rojo-Rodes JE. Employment Support Needs of People with Schizophrenia: A Scoping Study. Journal Of Occupational Rehabilitation. 2019;29(1):1-10.

379. Carpenter C, Daly J. Creating an Employment Collaborative. Journal of Vocational Rehabilitation. 2019;50(3):331-8.

380. Carta MG, Sancassiani F, Lecca ME, Pintus E, Pintus M, Pisano E, et al. Coping with crisis: People with severe mental disorders acting for social change through sustainable energy. Clinical Practice and Epidemiology in Mental Health. 2013;9.

381. Carter EW, Bendetson S, Guiden CH. Family Perspectives on the Appeals of and Alternatives to Sheltered Employment for Individuals with Severe Disabilities. Research & Practice for Persons with Severe Disabilities. 2018;43(3):145-64.

382. Carter EW, McMillan E, Willis W. The TennesseeWorks Partnership: Elevating employment outcomes for people with intellectual and developmental disabilities. Journal of Vocational Rehabilitation. 2017;47(3):365-78.

383. Cartwright KA, Lecomte T, Corbière M, Lysaker P. Narrative development and supported employment of persons with severe mental illness. Journal Of Mental Health (Abingdon, England). 2017:1-8.

384. Cartwright KA, Lecomte T, Corbière M, Lysaker P. Narrative development and supported employment of persons with severe mental illness. Journal Of Mental Health (Abingdon, England). 2020;29(1):12-9.

385. Caruana E, Cotton S, Killackey E, Allott K. The relationship between cognition, job complexity, and employment duration in first-episode psychosis. Psychiatric Rehabilitation Journal. 2015;38(3):210-7.

386. Case-Smith J, Cleary D, Persch A. Current Practices in Job Matching for Individuals With Intellectual and Developmental Disabilities...AOTA/NBCOT National Student Conclave Dearborn Michigan November 18-19 2016. American Journal of Occupational Therapy. 2015;69:290-3.

387. Cassinello K, Bramley S. Keeley's journey: from service user to service provider. Work (Reading, Mass). 2012;43(1):91-7.

388. Castelein S, Swart M, Wolters G, Bruggeman R, Knegtering H. Does routine outcome monitoring in schizophrenia improve the implementation of evidence based care? Schizophrenia Research. 2014;153(SUPPL. 1):S185.

389. Castle D, Crosse C, Morgain D, McDowell C, Rossell S, Thomas N, et al. Helping people with a mental illness obtain work: the Health Optimisation Program for Employment. Australasian Psychiatry: Bulletin Of Royal Australian And New Zealand College Of Psychiatrists. 2016;24(4):337-41.

390. Catty J, Koletsi M, White S, Becker T, Fioritti A, Kalkan R, et al. Therapeutic relationships: their specificity in predicting outcomes for people with psychosis using clinical and vocational services. Social Psychiatry and Psychiatric Epidemiology. 2009:1-7.

391. Catty J, Koletsi M, White S, Becker T, Fioritti A, Kalkan R, et al. Therapeutic relationships: their specificity in predicting outcomes for people with psychosis using clinical and vocational services. Social Psychiatry And Psychiatric Epidemiology. 2010;45(12):1187-93.

392. Catty J, White S, Koletsi M, Becker T, Fioritti A, Kalkan R, et al. Therapeutic relationships in vocational rehabilitation: predicting good relationships for people with psychosis. Psychiatry Research. 2011;187(1-2):68-73.

393. Cavenaugh B, Giesen JM. A Systematic Review of Transition Interventions Affecting the Employability of Youths with Visual Impairments. Journal of Visual Impairment & Blindness. 2012;106(7):400-13.

394. Celona D, Garino D, Bertossi F, Impagnatiello M, Botter V, Sandri F, et al. Multidimensional approach in persons with schizophrenia spectrum disorders. European Psychiatry. 2015;30(SUPPL. 1):868.

395. Ceolta‐Smith J, Salway S, Tod AM. A Review of Health-related Support Provision within the UK Work Programme - What's on the Menu? Social Policy & Administration. 2015;49(2):254-76.

396. Ceolta‐Smith J, Salway S, Tod AM. Experiences from the frontline: An exploration of personal advisers’ practice with claimants who have health‐related needs within UK welfare‐to‐work provision. Health & Social Care in the Community. 2018;26(4):e598-e608.

397. Chambless CE, Julnes G, McCormick ST, Reither A. Supporting Work Effort of SSDI Beneficiaries: Implementation of Benefit Offset Pilot Demonstration. Journal of Disability Policy Studies. 2011;22(3):179-88.

398. Chan ASM, Tsang HWH, Li SMY. Case report of integrated supported employment for a person with severe mental illness. The American Journal Of Occupational Therapy: Official Publication Of The American Occupational Therapy Association. 2009;63(3):238-44.

399. Chan F, Chiu CY, Strauser D. Tailoring vocational services to survivor's employment status. Psycho-Oncology. 2013;22(SUPPL. 3):110.

400. Chan F, Sung C. Effect of state vocational rehabilitation services on employment outcome of people with epilepsy. Epilepsy Currents. 2012;12(1 SUPPL. 1).

401. Chan JYC, Hirai HW, Tsoi KKF. Can computer-assisted cognitive remediation improve employment and productivity outcomes of patients with severe mental illness? A meta-analysis of prospective controlled trials. Journal of Psychiatric Research. 2015;68:293-300.

402. Chan JYC, Hirai HW, Tsoi KKF, Anaya BBBBBCCDEEEEFFGGHHKLLLLMMMMMMM. Can computer-assisted cognitive remediation improve employment and productivity outcomes of patients with severe mental illness? A meta-analysis of prospective controlled trials. Journal of Psychiatric Research. 2015;68:293-300.

403. Chan LG, Goh SE, Chua K. Prevalence and pattern of psychiatric disorders after traumatic brain injury and its association with long-term psychosocial outcomes in a Singaporean sample. Annals of the Academy of Medicine Singapore. 2013;42(9 SUPPL. 1):S157.

404. Chanen AM, Nicol K, Betts JK, Bond GR, Mihalopoulos C, Jackson HJ, et al. INdividual Vocational and Educational Support Trial (INVEST) for young people with borderline personality disorder: study protocol for a randomised controlled trial. Trials. 2020;21(1):583.

405. Chang L, Douglas N, Scanlan JN, Still M. Implementation of the enhanced intersectoral links approach to support increased employment outcomes for consumers of a large metropolitan mental health service. British Journal of Occupational Therapy. 2016;79(11):643-50.

406. Chang Y-J, Wang T-Y, Chen Y-R. A location-based prompting system to transition autonomously through vocational tasks for individuals with cognitive impairments. Research In Developmental Disabilities. 2011;32(6):2669-73.

407. Chapman C, Rosewall M. High supported employment & education engagement in NAVIGATE patients. Early Intervention in Psychiatry. 2018;12(Supplement 1):199.

408. Charette-Dussault E, Corbiere M. An Integrative Review of the Barriers to Job Acquisition for People With Severe Mental Illnesses. Journal of Nervous and Mental Disease. 2019;207(7):523-37.

409. Charles C, Miller B, Kerrigan A, Edwards J. The effectiveness of evidence based principles with traumatic brain injury veteran's successful community integration through employment. Journal of Head Trauma Rehabilitation. 2013;28(5):E42.

410. Charzyńska K, Kucharska K, Mortimer A. Does employment promote the process of recovery from schizophrenia? A review of the existing evidence. International Journal Of Occupational Medicine And Environmental Health. 2015;28(3):407-18.

411. Charzynska K, Kucharska K, Mortimer A, Addington AAABBBBBBBBBCCDDSDEEGGHHHK-PK-PL. Does employment promote the process of recovery from schizophrenia? A review of the existing evidence. International Journal of Occupational Medicine and Environmental Health. 2015;28(3):407-18.

412. Chase B. Good fences make good neighbors: Collaborating with families to create employment opportunity. Journal of Vocational Rehabilitation. 2021;54(3):279-83.

413. Chen N, Lal S. Stakeholder Perspectives on IPS for Employment: A Scoping Review. Canadian Journal Of Occupational Therapy Revue Canadienne D'ergotherapie. 2020;87(4):307-18.

414. Chen N, Lal S, Alverson AAAABBBBBBBBBBBCCDPEGGGHHJKKKK. Stakeholder perspectives on IPS for employment: A scoping review. Canadian Journal of Occupational Therapy / Revue Canadienne D'Ergotherapie. 2020;87(4):307-18.

415. Cheng ASK, Chiu FPF, Fung MSM, Au RWC. A review of supported employment services for people with mental disabilities in Hong Kong. Journal of Vocational Rehabilitation. 2015;42(1):75-83.

416. Cheng C, Oakman J, Bigby C, Fossey E, Cavanagh J, Meacham H, et al. What constitutes effective support in obtaining and maintaining employment for individuals with intellectual disability? A scoping review. Journal of Intellectual & Developmental Disability. 2018;43(3):317-27.

417. Cheng K-Y, Chen S-Y, Lin C-Y. Mortality among patients with schizophrenia and vocational rehabilitation program services under Taiwan's psychiatric care reform. International Journal Of Mental Health Systems. 2016;10:32-.

418. Cheng K-Y, Chen S-Y, Lin C-Y, Akiyama BBBCCCCDADFGGGHJJKLLLMMMMOPRRR. Mortality among patients with schizophrenia and vocational rehabilitation program services under Taiwan's psychiatric care reform. International Journal of Mental Health Systems. 2016;10.

419. Cheng K-Y, Yen C-F, Balogun-Mwangi BCCDJJKL-ELMMMMRRRRSSSSTVWY. The social support, mental health, psychiatric symptoms, and functioning of persons with schizophrenia participating in peer co-delivered vocational rehabilitation: A pilot study in Taiwan. BMC Psychiatry. 2021;21.

420. Chereau I, Fiorito N, Herouin F, Llorca PM, Blanc O. Employment support: A new approach towards inclusion. Annales Medico-Psychologiques. 2021;179(4):370-3.

421. Chinnery G. More than a job for today: career focused IPS and young people with psychosis. Early Intervention in Psychiatry. 2014;8(SUPPL. 1):8.

422. Chiu C, Chan F, Strauser D, Feuerstein M. Differential vocational rehabilitation services needs between employed and unemployed cancer survivors. Psycho-Oncology. 2010;19(SUPPL. 1):S75-S6.

423. Chiu C-Y, Chan F, Bishop M, da Silva Cardoso E, O'Neill J. State vocational rehabilitation services and employment in multiple sclerosis. Multiple Sclerosis (Houndmills, Basingstoke, England). 2013;19(12):1655-64.

424. Chiu C-Y, Chan F, Strauser D, Feuerstein M, Ditchman N, Cardoso E, et al. State rehabilitation services tailored to employment status among cancer survivors. Journal Of Occupational Rehabilitation. 2014;24(1):89-99.

425. Chiu C-Y, Sharp S, Pfaller J, Rumrill P, Cheing G, Sanchez J, et al. Differential vocational rehabilitation service patterns related to the job retention and job placement needs of people with diabetes. Journal of Vocational Rehabilitation. 2015;42(2):177-85.

426. Chong HK. Research of independent living-supporting service for developmental disabilities in era of convergence - focusing on supporting developmental disabilities in Japan. Research Journal of Pharmacy and Technology. 2019;12(4):1979-85.

427. Chouinard V. Women with disabilities' experiences of government employment assistance in Canada. Disability And Rehabilitation. 2010;32(2):148-58.

428. Chouinard V, Allaire BBCCFHJJJKPPRSWW. Women with disabilities' experiences of government employment assistance in Canada. Disability and Rehabilitation: An International, Multidisciplinary Journal. 2010;32(2):148-58.

429. Chow CM, Cichocki B. Predictors of Job Accommodations for Individuals With Psychiatric Disabilities. Rehabilitation Counseling Bulletin. 2016;59(3):172-84.

430. Chow CM, Cichocki B, Croft B. The impact of job accommodations on employment outcomes among individuals with psychiatric disabilities. Psychiatric Services (Washington, DC). 2014;65(9):1126-32.

431. Christensen JJ, Hetherington S, Daston M, Riehle E. Longitudinal outcomes of Project SEARCH in upstate New York. Journal of Vocational Rehabilitation. 2015;42(3):247-55.

432. Christensen JJ, Richardson K. Project SEARCH workshop to work: Participant reflections on the journey through career discovery. Journal of Vocational Rehabilitation. 2017;46(3):341-54.

433. Christensen JJ, Richardson K, Hetherington S. New York State Partnerships in Employment. Journal of Vocational Rehabilitation. 2017;47(3):351-63.

434. Christensen TN, Eplov L, Nordentoft M. The effectiveness of IPS enhanced with cognitive remediation and social skills training for people with severe mental illness in Denmark: A randomized controlled trial. Early Intervention in Psychiatry. 2012;6(SUPPL.1):97.

435. Christensen TN, Gammelgaard I, Stenager E, Nordentoft M, Eplov LF. Individual placement and support (IPS) supplemented with cognitive remediation and work-related social skills training in Denmark: A randomized clinical trial. Early Intervention in Psychiatry. 2014;8(SUPPL. 1):44.

436. Christensen TN, Nielsen IG, Nordentoft M, Eplov LF. Vocational outcomes of individual placement and support, cognitive remediation and work-related social skills training for people with severe mental illness in Denmark: Results from a randomized controlled trial. Early Intervention in Psychiatry. 2018;12(Supplement 1):51.

437. Christensen TN, Nielsen IG, Stenager E, Morthorst BR, Lindschou J, Nordentoft M, et al. Individual Placement and Support supplemented with cognitive remediation and work-related social skills training in Denmark: study protocol for a randomized controlled trial. Trials. 2015;16:280-.

438. Christensen TN, Nordentoft M. The effectiveness of IPS enhanced with cognitive remediation and social skills training for people with severe mental illness in Denmark: A randomised controlled trial. Schizophrenia Bulletin. 2013;39(SUPPL. 1):S285.

439. Christensen TN, Nordentoft M, Eplov LF. Limited Conclusions Can Be Reached From Danish Randomized Clinical Trial of Supported Employment-Reply. JAMA Psychiatry. 2019.

440. Christensen TN, Nordentoft M, Eplov LF. Limited Conclusions Can Be Reached From Danish Randomized Clinical Trial of Supported Employment-Reply. JAMA Psychiatry. 2020;77(3):327-8.

441. Christensen TN, Wallstrøm IG, Bojesen AB, Nordentoft M, Eplov LF. Predictors of work and education among people with severe mental illness who participated in the Danish individual placement and support study: findings from a randomized clinical trial. Social Psychiatry And Psychiatric Epidemiology. 2021.

442. Christensen TN, Wallstrøm IG, Stenager E, Bojesen AB, Gluud C, Nordentoft M, et al. Effects of Individual Placement and Support Supplemented With Cognitive Remediation and Work-Focused Social Skills Training for People With Severe Mental Illness: A Randomized Clinical Trial. JAMA Psychiatry. 2019.

443. Church HR, Seewald PM, Clark JMR, Jak AJ, Twamley EW. Predictors of work outcomes following supported employment in veterans with a history of traumatic brain injury. Neurorehabilitation. 2019;44(3):333-9.

444. Cichocki B. The alliance in psychiatric rehabilitation: Client characteristics associated with the initial alliance in a supported employment program. Work (Reading, Mass). 2015;52(4):811-24.

445. Cichocki B, Ackerman A-DAABBBCCCCCCCCCCC-CDDW-VODDDDDD. The alliance in psychiatric rehabilitation: Client characteristics associated with the initial alliance in a supported employment program. Work: Journal of Prevention, Assessment & Rehabilitation. 2015;52(4):811-24.

446. Cimera RE. The monetary benefits and costs of hiring supported employees: a pilot study. Journal of Vocational Rehabilitation. 2009;30(2):111-9.

447. Cimera RE. The economics of supported employment: What new data tell us. Journal of Vocational Rehabilitation. 2012;37(2):109-17.

448. Cimera RE. The outcomes achieved by previously placed supported employees with intellectual disabilities: Second verse same as the first? Journal of Vocational Rehabilitation. 2012;36(1):65-71.

449. Cimera RE. Reducing the Cost of Providing Supported Employment Services: A Preliminary Study. Journal of Rehabilitation. 2014;80(3):4-10.

450. Cimera RE. The Percentage of Supported Employees With Significant Disabilities Who Would Earn More in Sheltered Workshops. Research & Practice for Persons with Severe Disabilities. 2017;42(2):108-20.

451. Cimera RE, Anthony BBBBBCCCCCCDGHHJKLLLLLLNU. The outcomes and costs of public vocational rehabilitation consumers with mental illness. Special Issue: Psychiatric rehabilitation: Demographic subpopulations. 2009;40(2):28-33.

452. Cimera RE, Baer BBCCCCCCCCCCCCHHHHJKKLLLMMNRRR. National cost efficiency of supported employees with intellectual disabilities: 2002 to 2007. American Journal on Intellectual and Developmental Disabilities. 2010;115(1):19-29.

453. Cimera RE, Boardman BBBCCCCCCCCCCCCCCCCCCCCHHHJJKK. The economics of supported employment: What new data tell us. Journal of Vocational Rehabilitation. 2012;37(2):109-17.

454. Cimera RE, Bond CCCCCCCCHJKLLLLRRTTWZ. A comparison of the cost-ineffectiveness of supported employment versus sheltered work services by state and demographics of program participants. Journal of Vocational Rehabilitation. 2016;45(3):281-94.

455. Cimera RE, Burgess S. Do adults with autism benefit monetarily from working in their communities? Journal of Vocational Rehabilitation. 2011;34(3):173-80.

456. Cimera RE, Cowan RJ. The costs of services and employment outcomes achieved by adults with autism in the US. Autism: The International Journal Of Research And Practice. 2009;13(3):285-302.

457. Cimera RE, Cowan RJ. The costs of services and employment outcomes achieved by adults with autism in the United States. Autism: The International Journal of Research & Practice. 2009;13(3):285-302.

458. Cimera RE, Halpern MMSWWBDDMHGRRGBBBKNDHSIBTRWMMH. Does being in sheltered workshops improve the employment outcomes of supported employees with intellectual disabilities? Journal of Vocational Rehabilitation. 2011;35(1):21-7.

459. Cimera RE, Lam LCCRRBBBLKKTLNSTHHHWBCCCCCCCCC. The national costs of supported employment to vocational rehabilitation: 2002 to 2006. Journal of Vocational Rehabilitation. 2009;30(1):57-66.

460. Cimera RE, Oswald G. An exploration of the costs of services funded by vocational rehabilitation. Journal of Rehabilitation. 2009;75(1):18-26.

461. Cimera RE, Rumrill PD, Chan F, Kaya C, Bezyak J, Adams BCEGHHKLL-CMMMPRRSSSW. Vocational rehabilitation services and outcomes for transition-age youth with visual impairments and blindness. Journal of Vocational Rehabilitation. 2015;43(2):103-11.

462. Cimera RE, Super BLBRKNHHWCCCCCCWCCL. The outcomes achieved by previously placed supported employees with intellectual disabilities: Second verse same as the first? Journal of Vocational Rehabilitation. 2012;36(1):65-71.

463. Ciniselli G, Dighera R, Quarenghi A, Cantoni S. [Employment of psychiatrically disabled people. The Lavoro LiberaMente project]. La Medicina Del Lavoro. 2009;100(6):408-16.

464. Cissen-van Heugten WHJM, van der Heijden PT, Slaats I, Lagerveld SE. [Faster recovery of work participation through collaboration between specialist mental health care and Employee Insurance Agency (UWV): a pilot study]. Tijdschrift Voor Psychiatrie. 2019;61(5):326-34.

465. Clayton S, Bambra C, Gosling R, Povall S, Misso K, Whitehead M. Assembling the evidence jigsaw: insights from a systematic review of UK studies of individual-focused return to work initiatives for disabled and long-term ill people. BMC Public Health. 2011;11:170-.

466. Clayton S, Barr B, Nylen L, Burström B, Thielen K, Diderichsen F, et al. Effectiveness of return-to-work interventions for disabled people: a systematic review of government initiatives focused on changing the behaviour of employers. European Journal Of Public Health. 2012;22(3):434-9.

467. Clayton S, Gosling R, Povall S, Misso K, Bambra C, Whitehead M. 015 Pathways to work? Insights from a systematic review of the UK's return to work initiatives for disabled and chronically ill people. Journal of Epidemiology & Community Health. 2010;64:A6-A.

468. Cobb J, Wittenburg DC, Stepanczuk C. POSSIBLE STATE INTERVENTION OPTIONS TO SERVE TRANSITION-AGE YOUTHS: LESSONS FROM THE WEST VIRGINIA YOUTH WORKS DEMONSTRATION PROJECT. Social Security Bulletin. 2018;78(3):43-58.

469. Cocks E. Response to 'Measuring the fidelity of supported employment for people with severe mental illness'. Australian Occupational Therapy Journal. 2009;56(5):369-.

470. Cocks E. Response to 'Measuring the fidelity of supported employment for people with severe mental illness'...Waghorn 2009. Australian Occupational Therapy Journal, 56, 365-366. Australian Occupational Therapy Journal. 2009;56(5):369-.

471. Cocks E, Boaden R. Evaluation of an employment program for people with mental illness using the Supported Employment Fidelity Scale. Australian Occupational Therapy Journal. 2009;56(5):300-6.

472. Cocks E, Boaden R, Becker BBBBCCCCDJLM-WMMRWSWWW. Evaluation of an employment program for people with mental illness using the Supported Employment Fidelity Scale. Australian Occupational Therapy Journal. 2009;56(5):300-6.

473. Cocks E, Lewis G, Thoresen S. Economic and social outcomes of completing traineeships and apprenticeships for Australians with disabilities. Journal of Intellectual Disability Research. 2012;56(7-8):710.

474. Cocks E, Thoresen SH, Lee EAL, Ball BBCCCCCCCCDEEEFFGHIJJKKLLMMNRS. Pathways to employment and quality of life for apprenticeship and traineeship graduates with disabilities. International Journal of Disability, Development and Education. 2015;62(4):422-37.

475. Cocks E, Thoresen SH, Lim Lee EA, Bagshaw BBCCCCCEFGGGHHJJJKKLLLLLMNW. Employment and related economic outcomes for Australian apprenticeship and traineeship graduates with disabilities: Baseline findings from a national three-year longitudinal study. Journal of Vocational Rehabilitation. 2013;39(3):205-17.

476. Cogné M, Wiart L, Simion A, Dehail P, Mazaux JM. Five-year follow-up of persons with brain injury entering the French vocational and social rehabilitation programme UEROS: Return-to-work, life satisfaction, psychosocial and community integration. Brain Injury. 2017;31(5):655-66.

477. Cogne M, Wiart L, Simion A, Dehail P, Mazaux JM, Alaszewski AAA-LBBBBCCDDDD-CFFFFFLGGGGGHHHH. Five-year follow-up of persons with brain injury entering the French vocational and social rehabilitation programme UEROS: Return-to-work, life satisfaction, psychosocial and community integration. Brain Injury. 2017;31(5):655-66.

478. Cohen DA, Klodnick VV, Stevens L, Fagan MA, Spencer ES. Implementing Adapted Individual Placement and Support (IPS) Supported Employment for Transition-Age Youth in Texas. Community Mental Health Journal. 2019.

479. Cohen DA, Klodnick VV, Stevens L, Fagan MA, Spencer ES. Implementing Adapted Individual Placement and Support (IPS) Supported Employment for Transition-Age Youth in Texas. Community Mental Health Journal. 2020;56(3):513-23.

480. Cohen MJ, Becker DR. Family advocacy for the IPS supported employment project: accomplishments and challenges. Psychiatric Rehabilitation Journal. 2014;37(2):148-50.

481. Cohen MJ, Becker DR. Family advocacy for the IPS supported employment project: Accomplishments and challenges...Individual Placement and Support. Psychiatric Rehabilitation Journal. 2014;37(2):148-50.

482. Colleran A, Fannon D, Cox M, Murray G. Vision for change in practice: a pilot study of recovery oriented mental health services. International Journal of Integrated Care (IJIC). 2017;17:1-2.

483. Conley R, Conroy JW. The Florida Freedom Initiative on employment of people with significant disabilities: lessons to be learned. Journal of Vocational Rehabilitation. 2009;31(1):19-27.

484. Connell M, King R, Crowe T. Can employment positively affect the recovery of people with psychiatric disabilities? Psychiatric Rehabilitation Journal. 2011;35(1):59-63.

485. Conroy BE, Milani F, Levine M, Stein J, Zorowitz R, Harvey R, et al. Vocational rehabilitation after a stroke. 2009:735-46.

486. Conroy JW, Ferris CS, Irvine R, Blanck BCCCCHMNRR. Microenterprise options for people with intellectual and developmental disabilities: An outcome evaluation. Journal of Policy and Practice in Intellectual Disabilities. 2010;7(4):269-77.

487. Contreras N, Rossell SL, Castle DJ, Fossey E, Morgan D, Crosse C, et al. Enhancing work-focused supports for people with severe mental illnesses in australia. Rehabilitation Research And Practice. 2012;2012:863203-.

488. Conyers L, Boomer KB. Examining the role of vocational rehabilitation on access to care and public health outcomes for people living with HIV/AIDS. Disability & Rehabilitation. 2014;36(14):1203-10.

489. Conyers L, Boomer KB, Agresti ABBCCCCCGGHHJKM-KMMMNRRRRSSVOW. Evaluating the relative impact of State Vocational Rehabilitation and American Job Centers on contributing to the goals of the National HIV/AIDS Strategy. Journal of Vocational Rehabilitation. 2017;47(2):135-47.

490. Cook JA, Burke-Miller JK. Reasons for job separations in a cohort of workers with psychiatric disabilities. Journal Of Rehabilitation Research And Development. 2015;52(4):371-84.

491. Cook JA, Burke-Miller JK, Roessel E. Long-Term Effects of Evidence-Based Supported Employment on Earnings and on SSI and SSDI Participation Among Individuals With Psychiatric Disabilities. The American Journal Of Psychiatry. 2016;173(10):1007-14.

492. Coombes K, Haracz K, Robson E, James C. Pushing through: Mental health consumers' experiences of an individual placement and support employment programme. British Journal of Occupational Therapy. 2016;79(11):651-9.

493. Cooney K. The promise and pitfalls of employer-linked job training for disadvantaged workers. Administration in Social Work. 2010;34(1):27-48.

494. Cooper S, Powell VB, So R, Fisher M, Vinogradov S. Neuroplasticity-based cognitive training in schizophrenia in a "real world" setting. Schizophrenia Bulletin. 2011;37(SUPPL. 1):241.

495. Corbière M, Brouwers E, Lanctôt N, van Weeghel J. Employment specialist competencies for supported employment programs. Journal Of Occupational Rehabilitation. 2014;24(3):484-97.

496. Corbière M, Lanctôt N, Lecomte T, Latimer E, Goering P, Kirsh B, et al. A pan-Canadian evaluation of supported employment programs dedicated to people with severe mental disorders. Community Mental Health Journal. 2010;46(1):44-55.

497. Corbiere M, Lecomte T. Vocational services offered to people with severe mental illness. Journal of Mental Health. 2009;18(1):38-50.

498. Corbiere M, Lecomte T, Alden ABBBBBBBBBBBBBBBBBCCCCCCCCCCDD. Vocational services offered to people with severe mental illness. Journal of Mental Health. 2009;18(1):38-50.

499. Corbiere M, Lecomte T, Lysaker P. A group cognitive behavioral intervention for people registered in supported employment programs: CBT-SE. Early Intervention in Psychiatry. 2014;8(SUPPL. 1):83.

500. Corbière M, Lecomte T, Reinharz D, Kirsh B, Goering P, Menear M, et al. Predictors of Acquisition of Competitive Employment for People Enrolled in Supported Employment Programs. The Journal Of Nervous And Mental Disease. 2017;205(4):275-82.

501. Corbière M, Villotti P, Lecomte T, Bond GR, Lesage A, Goldner EM. Work accommodations and natural supports for maintaining employment. Psychiatric Rehabilitation Journal. 2014;37(2):90-8.

502. Corbiere M, Villotti P, Toth K, Waghorn G. Disclosure of a mental disorder in the workplace and work accommodations: Two factors associated with job tenure of people with severe mental disorders. Encephale. 2014;40(SUPPL. 2):S91-S102.

503. Corbière M, Villotti P, Toth K, Waghorn G. [Disclosure of a mental disorder in the workplace and work accommodations: two factors associated with job tenure of people with severe mental disorders]. L'encephale. 2014;40 Suppl 2:S91-S102.

504. Corbiere M, Villotti P, Toth K, Waghorn G, Ajzen ABBBBBBBB-MBCCCCCCCCCCCCCCCDEFG. Disclosure of a mental disorder in the workplace and work accommodations: Two factors associated with job tenure of people with severe mental disorders. La divulgation du trouble mental et les mesures d'accommodements de travail: Deux facteurs du maintien en emploi des personnes aux prises avec un trouble mental grave. 2014;40(Suppl 2):S91-S102.

505. Corbière M, Zaniboni S, Lecomte T, Bond G, Gilles P-Y, Lesage A, et al. Job acquisition for people with severe mental illness enrolled in supported employment programs: a theoretically grounded empirical study. Journal Of Occupational Rehabilitation. 2011;21(3):342-54.

506. Cornes M, Manthorpe J, Joly L, O'Halloran S. Reconciling recovery, personalisation and Housing First: integrating practice and outcome in the field of multiple exclusion homelessness. Health & Social Care In The Community. 2014;22(2):134-43.

507. Correll CU, Galling B, Pawar A, Krivko A, Bonetto C, Ruggeri M, et al. Comparison of early intervention services vs treatment as usual for early-phase psychosis: A systematic review, meta-analysis, and meta-regression. JAMA Psychiatry. 2018;75(6):555-65.

508. Cosottile DW, DeFulio A. The Compatibility of Employment-Based Contingency Management and Vocational Services at the Department of Veterans Affairs. Psychology of Addictive Behaviors. 2019.

509. Cosottile DW, DeFulio A, Bonn-Miller BBCDDDDDDDDEHJJKKKLLLOPPPPPRR. The compatibility of employment-based contingency management and vocational services at the Department of Veterans Affairs. Psychology of Addictive Behaviors. 2019:No-Specified.

510. Costa M, Baker M, Davidson L, Giard J, Guillorn L, Ibáñez AG, et al. Provider perspectives on employment for people with serious mental illness. The International Journal Of Social Psychiatry. 2017;63(7):632-40.

511. Cotner B, Ottomanelli L, Arriola N, Toscano R, Carlomagno J. Tools for a Working Life: Engaging Providers in Toolkit Development to Improve Rehabilitation Outcomes. Archives of Physical Medicine and Rehabilitation. 2019;100(10):e135.

512. Cotner B, Ottomanelli L, O'Connor DR, Njoh E, Jones V. Delivering vocational services to rural veterans with disabilities through video telerehabilitation. Archives of Physical Medicine and Rehabilitation. 2016;97(10):e95.

513. Cotner BA, Njoh EN, Trainor JK, O'Connor DR, Barnett SD, Ottomanelli L. Facilitators and barriers to employment among veterans with spinal cord injury receiving 12 months of evidence-based supported employment services. Topics In Spinal Cord Injury Rehabilitation. 2015;21(1):20-30.

514. Cotner BA, Njoh EN, Trainor JK, O'Connor DR, Barnett SD, Ottomanelli L. Facilitators and Barriers to Employment Among Veterans with Spinal Cora Injury Receiving 12 Months or Evidence-Based Supported Employment Services. Topics in Spinal Cord Injury Rehabilitation. 2015;21(1):20-30.

515. Cotner BA, Ottomanelli L, Keleher V, Dirk L. Scoping review of resources for integrating evidence-based supported employment into spinal cord injury rehabilitation. Disability And Rehabilitation. 2019;41(14):1719-26.

516. Cotner BA, Ottomanelli L, O'Connor DR, Njoh EN, Barnett SD, Miech EJ. Quality of Life Outcomes for Veterans With Spinal Cord Injury Receiving Individual Placement and Support (IPS). Topics In Spinal Cord Injury Rehabilitation. 2018;24(4):325-35.

517. Cotner BA, Ottomanelli L, O'Connor DR, Trainor JK. Provider-identified barriers and facilitators to implementing a supported employment program in spinal cord injury. Disability And Rehabilitation. 2018;40(11):1273-9.

518. Cotton S, Allot K, Chinnery G, Jackson H, Killackey E. The relationship between vocational functioning and quality of life in patients with first episode psychosis. Early Intervention in Psychiatry. 2012;6(SUPPL.1):98.

519. Cotton S, Allott K, Chinnery G, Jackson H, Killackey E. The relationship between vocational functioning and quality of life in patients with first episode psychosis. Early Intervention in Psychiatry. 2014;8(SUPPL. 1):81.

520. Covell NH, Margolies PJ, Myers RW, Ruderman D, Fazio ML, McNabb LM, et al. Scaling up evidence-based behavioral health care practices in New York state. Psychiatric Services (Washington, DC). 2014;65(6):713-5.

521. Covell NH, Margolies PJ, Myers RW, Ruderman D, Fazio ML, McNabb LM, et al. State mental health policy: scaling up evidence-based behavioral health care practices in new york state. Psychiatric Services. 2014;65(6):713-5.

522. Cox A, Simmons H, Painter G, Philipson P, Hill R, Chester V. Real Work Opportunities: establishing an accessible vocational rehabilitation programme within a forensic intellectual disability service. Journal of Intellectual Disabilities & Offending Behaviour. 2014;5(4):160-6.

523. Cox ME, Land KA. The power of the employment specialist: Skills that impact outcomes. Journal of Vocational Rehabilitation. 2019;50(3):273-8.

524. Craig T, Shepherd G, Rinaldi M, Smith J, Carr S, Preston F, et al. Vocational rehabilitation in early psychosis: cluster randomised trial. The British Journal Of Psychiatry: The Journal Of Mental Science. 2014;205(2):145-50.

525. Crain M, Penhale C, Newstead C, Thomson L, Heah T, Barclay K. The contribution of IPS to recovery from serious mental illness: a case study. Work (Reading, Mass). 2009;33(4):459-64.

526. Crain M, Penhale C, Newstead C, Thomson L, Heah T, Barclay K. The contribution of IPS to recovery from serious mental illness: a case study...Individual Placement and Support. Work. 2009;33(4):459-64.

527. Cramm JM, Finkenflügel H, Kuijsten R, van Exel NJA. How employment support and social integration programmes are viewed by the intellectually disabled. Journal Of Intellectual Disability Research: JIDR. 2009;53(6):512-20.

528. Crespo-Facorro B. What we talk about when we talk about specialized early intervention programs: 15 years of PAFIP (Cantabria, Spain). Early Intervention in Psychiatry. 2016;10(Supplement 1):45.

529. Crotty G. People with intellectual disabilities moving into adulthood. Learning Disability Practice. 2016;19(5):32-7.

530. Cubillos L, Muñoz J, Caballero J, Mendoza M, Pulido A, Carpio K, et al. Addressing Severe Mental Illness Rehabilitation in Colombia, Costa Rica, and Peru. Psychiatric Services (Washington, DC). 2020:appips201900306-appips.

531. Cudd PA, Keijer U, Breding J. Work life, new technology and employment of disabled people: A twenty year programme. Technology & Disability. 2012;24(3):211-8.

532. Cuetoa B, Rodriguez V. Is sheltered employment more stable than ordinary employment? Work. 2016;55(1):51-61.

533. Cunningham J. Learning on the Job for People with Disabilities. NCSL Legisbrief. 2018;26(12):1-2.

534. Curran N. What can we learn from supported employment? Community Living. 2013;27(1):22-3.

535. D. Herron J. Recovery Lessons Learned from Individuals with Psychiatric Disabilities. Journal of Policy Practice. 2016;15(1/2):116-32.

536. Dague B. Sheltered employment, sheltered lives: Family perspectives of conversion to community-based employment. Journal of Vocational Rehabilitation. 2012;37(1):1-11.

537. Dague B, Migliore LNGMDDRBBBBBPMGJWGMPRRWWWWWWTF. Sheltered employment, sheltered lives: Family perspectives of conversion to community-based employment. Journal of Vocational Rehabilitation. 2012;37(1):1-11.

538. Dalto GB. A needs assessment of employment services for people with serious mental illness in northwestern Nevada. Dissertation Abstracts International: Section B: The Sciences and Engineering. 2021;82(3-B):No-Specified.

539. Darensbourg BL. Predictors of competitive employment of VR consumers with blindness or visual impairments. Journal of Vocational Rehabilitation. 2013;38(1):29-34.

540. Darus A. Rehabilitation paradigm: Return to work from disability. Occupational and Environmental Medicine. 2018;75(Supplement 2):A238.

541. Davies H. Supported employment works -- so let's have a workable strategy. Community Living. 2013;27(2):18-9.

542. Davis G, Sylvester JT. Vacational Rehabilitation. Montana Business Quarterly. 2012;50(4):14-7.

543. Davis LL, Blansett CM, Mumba MN, MacVicar D, Toscano R, Pilkinton P, et al. The methods and baseline characteristics of a VA randomized controlled study evaluating supported employment provided in primary care patient aligned care teams. BMC Medical Research Methodology. 2020;20(1):33.

544. Davis LL, Kyriakides TC, Suris A, Ottomanelli L, Drake RE, Parker PE, et al. Veterans individual placement and support towards advancing recovery: Methods and baseline clinical characteristics of a multisite study. Psychiatric Rehabilitation Journal. 2018;41(1):55-66.

545. Davis LL, Kyriakides TC, Suris AM, Ottomanelli LA, Mueller L, Parker PE, et al. Effect of Evidence-Based Supported Employment vs Transitional Work on Achieving Steady Work Among Veterans With Posttraumatic Stress Disorder: A Randomized Clinical Trial. JAMA Psychiatry. 2018;75(4):316-24.

546. Davis LL, Leon AC, Toscano R, Drebing CE, Ward LC, Parker PE, et al. A randomized controlled trial of supported employment among veterans with posttraumatic stress disorder. Psychiatric Services (Washington, DC). 2012;63(5):464-70.

547. Davis LL, Pilkinton P, Poddar S, Blansett C, Toscano R, Parker PE. Impact of social challenges on gaining employment for veterans with posttraumatic stress disorder: an exploratory moderator analysis. Psychiatric Rehabilitation Journal. 2014;37(2):107-9.

548. Davis LW, Lysaker PH, Eicher AC. Effects of amindfulness interventionon work outcomes for adults with schizophrenia. Schizophrenia Bulletin. 2011;37(SUPPL. 1):262-3.

549. Davis LW, Lysaker PH, Kristeller JL, Salyers MP, Kovach AC, Woller S. Effect of mindfulness on vocational rehabilitation outcomes in stable phase schizophrenia. Psychological Services. 2015;12(3):303-12.

550. Davis M, Sheidow AJ, McCart MR, Perrault RT. Vocational Coaches for Justice-Involved Emerging Adults. Psychiatric Rehabilitation Journal. 2018;41(4):266-76.

551. Dawson S, Muller J, Renigers V, Varona L, Kernot J. Consumer, health professional and employment specialist experiences of an individual placement and support programme. Scandinavian Journal Of Occupational Therapy. 2020:1-13.

552. De Boer AGM, Taskila T, Tamminga SJ, Frings-Dresen MHW, Feuerstein M, Verbeek JH. Interventions to enhance return-to-work for cancer patients. Cochrane Database of Systematic Reviews. 2009(1):CD007569.

553. de Bruyn M, Wright J. Joining the Dots: Theoretically Connecting the Vona du Toit Model of Creative Ability (VdTMoCA) with Supported Employment. South African Journal of Occupational Therapy. 2017;47(2):49-52.

554. de Graaf-Zijl M, Spijkerman M, Zwinkels W. Long-Term Effects of Individual Placement and Support Services for Disability Benefits Recipients with Severe Mental Illnesses. IZA Discussion Paper series. 2020;IZA DP No. 13772.

555. De Greef V. Analysis of barriers and facilitators of existing programs in Belgium for the purpose of implementing individual placement and support (IPS). Psychiatric Rehabilitation Journal. 2019.

556. De Greef V. Analysis of barriers and facilitators of existing programs in Belgium for the purpose of implementing individual placement and support (IPS). Psychiatric Rehabilitation Journal. 2020;43(1):18-23.

557. De Greef V, Bond BCCCDGFKMMNSSTTVCVCVCV. Analysis of barriers and facilitators of existing programs in Belgium for the purpose of implementing individual placement and support (IPS). Psychiatric Rehabilitation Journal. 2019:No-Specified.

558. De Greef V, Bond BCCCDGFKMMNSSTTVCVCVCV. Analysis of barriers and facilitators of existing programs in Belgium for the purpose of implementing individual placement and support (IPS). Special Issue: International Implementation of Individual Placement and Support. 2020;43(1):18-23.

559. De La Cour FLD, Rasmussen MA, Foged EM, Jensen LS, Schow T. Vocational rehabilitation in mild traumatic brain injury: Supporting return to work and daily life functioning. Frontiers in Neurology. 2019;10(FEB):103.

560. de Malmanche J, Robertson L. The Experience of KAI MAHI, an Employment Initiative for People with an Experience of Mental Illness, as Told by Zarna, Zeus, Lulu, Mary, Paul, and Hemi. Community Mental Health Journal. 2015;51(8):880-7.

561. de Pierrefeu I, Charbonneau C. [Two "hybrid" vocational services for people with a severe mental illness in France and Quebec]. L'encephale. 2014;40 Suppl 2:S66-S74.

562. de Pierrefeu I, Charbonneau C, Baptiste BBBCCCCCCdPdPDDDDLMPPPQRVVW. Two "hybrid" vocational services for people with a severe mental illness in France and Quebec. Deux structures d'insertion professionnelle de format hybride pour personnes avec un trouble mental : Les ESAT de transition de Messidor (France) et Acces-Cible SMT (Quebec). 2014;40(Suppl 2):S66-S74.

563. de Pierrefeu I, Corbière M, Pachoud B. [Supervisors and Employment Counselors in Messidor Transitional Social Firms Supporting People with Psychiatric Disability to Gain Competitive Employment]. Sante Mentale Au Quebec. 2017;42(2):155-71.

564. de Pierrefeu I, Corbière M, Pachoud B. Vocational Counselors in France: Comparison to Competencies of Employment Specialists Working in Canadian IPS Programs. Community Mental Health Journal. 2017;53(7):871-7.

565. de Pierrefeu I, Corbière M, Pachoud B. Les accompagnants à l'insertion professionnelle en milieu ordinaire pour les personnes en situation de handicap psychique au sein des ESAT de transition Messidor. Sante Mentale au Quebec. 2017;42(2):155-71.

566. de Winter L, Couwenbergh C, van Weeghel J, Bergmans C, Bond GR. Fidelity and IPS: does quality of implementation predict vocational outcomes over time for organizations treating persons with severe mental illness in the Netherlands? Social Psychiatry And Psychiatric Epidemiology. 2020;55(12):1607-17.

567. Dean D, Pepper J, Schmidt R, Stern S. THE EFFECTS OF VOCATIONAL REHABILITATION FOR PEOPLE WITH COGNITIVE IMPAIRMENTS. International Economic Review. 2015;56(2):399-426.

568. Dean D, Pepper JV, Schmidt R, Stern S. The Effects of Vocational Rehabilitation Services for People with Mental Illness. Journal of Human Resources. 2017;52(3):826-58.

569. Dean D, Schmidt R, Pepper J, Stern S. The Effects of Vocational Rehabilitation for People with Physical Disabilities. Journal of Human Capital. 2018;12(1):1-37.

570. Deforche B, Mommen J, Hublet A, De Roover W, Huys N, Clays E, et al. Evaluation of a Brief Intervention for Promoting Mental Health among Employees in Social Enterprises: A Cluster Randomized Controlled Trial. International Journal Of Environmental Research And Public Health. 2018;15(10).

571. Del Valle R, Leahy MJ, Sherman S, Anderson CA, Tansey T, Schoen B. Promising best practices that lead to employment in vocational rehabilitation: Findings from a four-state multiple case study. Journal of Vocational Rehabilitation. 2014;41(2):99-113.

572. Del Valle RJ. Rehabilitation counselor self-efficacy and work environment factors that promote the use of evidence-based practices in vocational rehabilitation service delivery. 2015(Ph.D.):122 p- p.

573. Demou E, Hanson M, Bakhshi A, Kennedy M, Macdonald EB. Working Health Services Scotland: a 4-year evaluation. Occupational Medicine (Oxford, England). 2018;68(1):38-45.

574. Dempsey I, Ford J. Employment for people with intellectual disability in Australia and the United Kingdom. Journal of Disability Policy Studies. 2009;19(4):233-43.

575. Dempsey I, Ford J, Albrecht ABBBBBBFHHHKKLMOBOBPRSSWY. Employment for people with intellectual disability in Australia and the United Kingdom. Journal of Disability Policy Studies. 2009;19(4):233-43.

576. Denning J, Hunter N. Breast cancer and return to work: A pilot. Breast Cancer Research and Treatment. 2016;159(1):196.

577. Denning J, Hunter N, Seaton B. Supporting cancer survivors returning to work: A pilot study of a vocational rehabilitation service. Psycho-Oncology. 2016;25(Supplement 1):17.

578. Denning J, Wiseman T. Return to work support for post-transplant patients. Psycho-Oncology. 2018;27(Supplement 2):26.

579. Denny-Brown N, O'Day B, McLeod S, Chow GHHLM-WMSSY. Staying employed: Services and supports for workers with disabilities. Journal of Disability Policy Studies. 2015;26(2):124-31.

580. DeTore NR, Hintz K, Khare C, Mueser KT. Disclosure of mental illness to prospective employers: Clinical, psychosocial, and work correlates in persons receiving supported employment. Psychiatry Research. 2019;273:312-7.

581. Deuchert E, Kauer L, Meisen Zannol F. Would you train me with my mental illness? Evidence from a discrete choice experiment. University of St. Gallen, School of Economics and Political Science; 2011.

582. Deuchert E, Kauer L, Meisen Zannol F. Would you train me with my mental illness? Evidence from a discrete choice experiment. The Journal Of Mental Health Policy And Economics. 2013;16(2):67-80.

583. Deuchert E, Kauer L, Zannol FM. Would you train me with my mental illness? Evidence from a Discrete Choice Experiment. Journal of Mental Health Policy and Economics. 2011;14(SUPPL. 1):S5.

584. Dewa CS, Hoch JS, Loong D, Trojanowski L, Bonato S. Evidence for the Cost-Effectiveness of Return-to-Work Interventions for Mental Illness Related Sickness Absences: A Systematic Literature Review. Journal Of Occupational Rehabilitation. 2021;31(1):26-40.

585. Dewa CS, Loong D, Trojanowski L, Bonato S. The effectiveness of augmented versus standard individual placement and support programs in terms of employment: a systematic literature review. Journal Of Mental Health (Abingdon, England). 2018;27(2):174-83.

586. Di Sarro R, Varrucciu N, Di Santantonio A, Bonsi II. The Integrated and Disability Health Program of AUSL Bologna. The Alstom experience for employment access in high functioning autism spectrum disorders. Annali dell'Istituto superiore di sanita. 2020;56(2):247-50.

587. Diallo A, Chia V, Rivas B, Aguirre A, Flowers C, Ngai K. The Use of Employment/Vocational Rehabilitation Services for Persons with HIV/AIDS and Substance Abuse: A potential Health Benefit. Journal of Applied Rehabilitation Counseling. 2017;48(4):28-37.

588. Dibben P, Wood G, O’Hara R. Do return to work interventions for workers with disabilities and health conditions achieve employment outcomes and are they cost effective? A systematic narrative review. Employee Relations. 2018;40(6):999-1014.

589. Dickson E, Taylor J. Supporting service users into employment: a literature review. Mental Health Practice. 2012;16(4):15-8.

590. Dijkers M. Rebuttal to Ottomanelli et al. Methods of a multisite randomized clinical trial of supported employment among veterans with spinal cord injury. J Rehabil Res Dev. 2009;46(7):919–30. Journal Of Rehabilitation Research And Development. 2010;47(5):vii.

591. Dillahunt-Aspillaga C, Powell-Cope G, Yanson J. An exploration of resource facilitation services for veterans with traumatic brain injury. Archives of Physical Medicine and Rehabilitation. 2017;98(10):e32.

592. Dillahunt-Aspillaga T, Menchetti B, Bruce T. Project RESULTS: Florida's collaborative approach to employing survivors of traumatic brain injury. Journal of Head Trauma Rehabilitation. 2010;25(5):389-90.

593. Dillahunt-Aspillaga T, Price P. Community capacity building and vocational models of service delivery for individuals with traumatic brain injury in the State of Florida. Brain Injury. 2010;24(3):442-3.

594. Dillo W, Lampen S, Neumann A, Steinmuller S, Wellmann B. The ressource orientated integration of chronic mentally ill into regular employment via "one-euro-jobs" - Initial experiences of a model project. Fortschritte der Neurologie Psychiatrie. 2010;78(5):288-93.

595. Dillo W, Lampen S, Neumann A, Steinmüller S, Wellmann B. [The resource orientated integration of chronic mentally ill into regular employment via "One-Euro-Jobs"--initial experiences of a model project]. Fortschritte Der Neurologie-Psychiatrie. 2010;78(5):288-93.

596. Dillo W, Lampen S, Neumann A, Steinmuller S, Wellmann B, Albrecht ABBBBBBBCCDHHHHKKKLLMMMMMPPRRR. The resource oriented integration of chronic mentally ill into regular employment via "One-Euro-Jobs"-Initial experiences in a model project. Die ressourcenorientierte Integration chronisch psychisch Kranker in den Arbeitsmarkt uber Ein-Euro- Jobs-Erste Erfahrungen eines Modellprojekts. 2010;78(5):288-93.

597. Ditchman NM, Miller JL, Easton AB. Vocational Rehabilitation Service Patterns: An Application of Social Network Analysis to Examine Employment Outcomes of Transition-Age Individuals With Autism. Rehabilitation Counseling Bulletin. 2018;61(3):143-53.

598. Dixon L, Lieberman J, Addington CHLMMMMM. Early psychosis intervention services: A work in progress. Schizophrenia Bulletin. 2015;41(1):23-4.

599. Dixon LB, Dickerson F, Bellack AS, Bennett M, Dickinson D, Goldberg RW, et al. The 2009 schizophrenia PORT psychosocial treatment recommendations and summary statements. Schizophrenia Bulletin. 2010;36(1):48-70.

600. Dixon LB, Goldman HH, Srihari VH, Kane JM. Transforming the Treatment of Schizophrenia in the United States: The RAISE Initiative. Annual Review of Clinical Psychology. 2018;14:237-58.

601. Dolce JN, Waynor WR. Effect of Psychiatric Symptoms on Employment Outcomes for Individuals Receiving Supported Employment Services: A Preliminary Study. Journal Of Psychosocial Nursing And Mental Health Services. 2018;56(3):33-8.

602. Dolce JN, Waynor WR, Blustein BBBBBCDDDDDFGGGGHKLLLMMMMMRRRR. Effect of psychiatric symptoms on employment outcomes for individuals receiving supported employment services: A preliminary study. Journal of Psychosocial Nursing and Mental Health Services. 2018;56(3):33-8.

603. Domin D, Butterworth J. The role of community rehabilitation providers in employment for persons with intellectual and developmental disabilities: results of the 2010-2011 National Survey. Intellectual And Developmental Disabilities. 2013;51(4):215-25.

604. Dominy M, Hayward-Butcher T. 'Is work good for you?' Does paid employment produce positive social capital returns for people with severe and enduring mental health conditions? Mental Health & Social Inclusion. 2012;16(1):14-25.

605. Donelly M, Given F. Employment programs and professionals with a disability. Work (Reading, Mass). 2010;36(2):217-25.

606. Donelly M, Given F, Condeluci CKGGBDDHLHHRGHCTLAMNSMSRRMSLBB. Employment programs and professionals with a disability. Special Issue: Participation in Work in Australia. 2010;36(2):217-25.

607. Donker-Cools BHPM, Daams JG, Wind H, Frings-Dresen MHW. Effective return-to-work interventions after acquired brain injury: A systematic review. Brain Injury. 2016;30(2):113-31.

608. Doose S. Supported employment in Germany. Journal of Vocational Rehabilitation. 2012;37(3):195-202.

609. dos Santos Rodrigues P, Luecking RG, Glat R, Daquer AFC. Improving workforce outcomes among persons with disabilities in Brazil through youth apprenticeships and customized employment. Journal of Vocational Rehabilitation. 2013;38(3):185-94.

610. Douglas G, Nadel S. Software Provider Finds Untapped Talent In Individuals on the Autism Spectrum. HR Focus. 2015;92(1):1-3.

611. Dowler DL, Walls RT. A Review of Supported Employment Services for People with Disabilities: Competitive Employment, Earnings, and Service Costs. Journal of Rehabilitation. 2014;80(1):11-21.

612. Drake RE. Employment and Schizophrenia: Three Innovative Research Approaches. Schizophrenia Bulletin. 2018;44(1):20-1.

613. Drake RE. Introduction to the special issue on Individual Placement and Support (IPS) International. Psychiatric Rehabilitation Journal. 2020;43(1):1.

614. Drake RE. Introduction to supported employment. Epidemiology And Psychiatric Sciences. 2020;29:e185.

615. Drake RE, Anthony BBBDDDDHJ-KKLMMMMSSW. Employment and schizophrenia: Three innovative research approaches. Schizophrenia Bulletin. 2018;44(1):20-1.

616. Drake RE, Becker D, Bush P, Xie H. The long-term impact of employment on mental health costs. Early Intervention in Psychiatry. 2010;4(SUPPL. 1):5.

617. Drake RE, Becker DR. Why not implement supported employment? Psychiatric Services (Washington, DC). 2011;62(11):1251-.

618. Drake RE, Becker DR. Robert Liberman's Influence on Supported Employment. American Journal of Psychiatric Rehabilitation. 2014;17(3):193-6.

619. Drake RE, Becker DR, Bond GR. Introducing Individual Placement and Support (IPS) supported employment in Japan. Psychiatry And Clinical Neurosciences. 2019;73(2):47-9.

620. Drake RE, Becker DR, Bond GR. Growth and Sustainment of Individual Placement and Support. Psychiatric Services (Washington, DC). 2020;71(10):1075-7.

621. Drake RE, Bond GR. IPS Support Employment: A 20-Year Update. American Journal of Psychiatric Rehabilitation. 2011;14(3):155-64.

622. Drake RE, Bond GR. Introduction to the special issue on individual placement and support. Psychiatric Rehabilitation Journal. 2014;37(2):76-8.

623. Drake RE, Bond GR, Alverson BBBBBBBBBBBCCCDDDDDDFGHHJKKKMM. IPS support employment: A 20-year update. American Journal of Psychiatric Rehabilitation. 2011;14(3):155-64.

624. Drake RE, Bond GR, Becker BBBBBBCCDDDDDDFGHHHKKLLLMMMMNN. Introduction to the special issue on individual placement and support. Special Issue: Individual Placement and Support (IPS) Model of Supported Employment. 2014;37(2):76-8.

625. Drake RE, Bond GR, Goldman HH, Hogan MF, Karakus M. Individual Placement And Support Services Boost Employment For People With Serious Mental Illnesses, But Funding Is Lacking. Health Affairs (Project Hope). 2016;35(6):1098-105.

626. Drake RE, Bond GR, Thornicroft G, Knapp M, Goldman HH, Adam A-SABBBBBBBBBBBBBBBBBCCCCCCCCCC. Mental health disability: An international perspective. Journal of Disability Policy Studies. 2012;23(2):110-20.

627. Drake RE, Frey W, Bond GR, Goldman HH, Salkever D, Miller A, et al. Assisting Social Security Disability Insurance beneficiaries with schizophrenia, bipolar disorder, or major depression in returning to work. The American Journal Of Psychiatry. 2013;170(12):1433-41.

628. Drake RE, Frey W, Karakus M, Salkever D, Bond GR, Goldman HH. Policy Implications of the Mental Health Treatment Study. Psychiatric Services (Washington, DC). 2016;67(10):1139-41.

629. Drake RE, Riley J, Goldman HH, Becker DR, Bond GR, Bennici FJ, et al. Supporting a Working Life When Disability Is Not Permanent. Psychiatric Services (Washington, DC). 2020:appips201900478-appips.

630. Drake RE, Sederer LI, Becker DR, Bond GR. COVID-19, Unemployment, and Behavioral Health Conditions: The Need for Supported Employment. Administration And Policy In Mental Health. 2021;48(3):388-92.

631. Drake RE, Strickler DC, Bond GR, Beers BCDDDGHHLMMRSSVWWW. Psychiatric rehabilitation in residential treatment settings. Psychiatric Annals. 2015;45(3):114-9.

632. Drake RE, Wallach MA. Employment is a critical mental health intervention. Epidemiology And Psychiatric Sciences. 2020;29:e178.

633. Drake RE, Whitley R, Anthony BBB-ZBBBCCCDDDDDDDDDDDFFGHHHHJJ. Recovery and severe mental illness: Description and analysis. The Canadian Journal of Psychiatry / La Revue canadienne de psychiatrie. 2014;59(5):236-42.

634. Drake RE, Xie H, Bond GR, McHugo GJ, Caton CLM. Early psychosis and employment. Schizophrenia Research. 2013;146(1-3):111-7.

635. Drebing CE, Bell M, Campinell EA, Fraser R, Malec J, Penk W, et al. Vocational services research: recommendations for next stage of work. Journal Of Rehabilitation Research And Development. 2012;49(1):101-19.

636. Dreher KC, Bond GR, Becker DR. Creation of a measure to assess knowledge of the individual placement and support model. Psychiatric Rehabilitation Journal. 2010;33(3):181-9.

637. Dreisinger BAZ. “PLEASE DON’T MIND OUR TATTOOS”: SINGAPORE’S RE-ENTRY PROGRAMS FOR THE FORMERLY INCARCERATED. World Policy Journal. 2017;34(2):43-54.

638. Dresser K, Clark HB, Deschênes N. Implementation of a Positive Development, Evidence-Supported Practice for Emerging Adults with Serious Mental Health Conditions: The Transition to Independence Process (TIP) Model. The Journal Of Behavioral Health Services & Research. 2015;42(2):223-37.

639. Drosos N, Theodoroulakis M. Employment as an integral part of social inclusion: The case of mental health service users in Greece. Journal of Intellectual Disability Research. 2019;63(7):862.

640. Druss BG. Supported employment over the long term: from effectiveness to sustainability. The American Journal Of Psychiatry. 2014;171(11):1142-4.

641. Druss BG, Anthony BBDDFHHHHKKKMMS. Supported employment over the long term: From effectiveness to sustainability. The American Journal of Psychiatry. 2014;171(11):1142-4.

642. Du Sert OP, Joober R, Lepage M, Malla A, Dama M, Groff M. Early trajectories of positive symptoms remission in first episode-psychosis: A 2-year follow-up study. Schizophrenia Bulletin. 2020;46(Supplement 1):S7-S8.

643. Dubreucq J. Recovery-oriented interventions in schizophrenia spectrum disorder (SSD). Annales Medico-Psychologiques. 2021;179(4):363-9.

644. Dudley R, Nicholson M, Stott P, Spoors G. Improving vocational outcomes of service users in an Early Intervention in Psychosis service. Early Intervention In Psychiatry. 2014;8(1):98-102.

645. Dunstan DA, Falconer AK, Price IR. The relationship between hope, social inclusion and mental wellbeing in supported work integration. Occupational and Environmental Medicine. 2018;75(Supplement 2):A562.

646. Dunstan DA, Falconer AK, Price IR, Andresen ABBBCCCCDDFGGHIIJJKKKLBMMMMPRS. The relationship between hope, social inclusion, and mental wellbeing in supported employment. Australian Journal of Rehabilitation Counselling. 2017;23(1):37-51.

647. Durbin J, Selick A, Langill G, Cheng C, Archie S, Butt S, et al. Using fidelity measurement to assess quality of early psychosis intervention services in Ontario. Psychiatric Services. 2019;70(9):840-4.

648. Durcan G. Beyond the gate: supporting the employment aspirations of offenders with mental health conditions. Mental Health & Social Inclusion. 2012;16(4):188-93.

649. Dutoit M, Besse C, Hausmann P, Spagnoli D, Bonsack C. [A supported employment experience in western Switzerland for persons with mental health disorders]. Revue Medicale Suisse. 2014;10(442):1711-4.

650. Duwe G, Akers BCHHLMMMNPPPPRRRSSSSTUUVW. The benefits of keeping idle hands busy: An outcome evaluation of a prisoner reentry employment program. Crime & Delinquency. 2015;61(4):559-86.

651. Eack SM, Hogarty GE, Greenwald DP, Hogarty SS, Keshavan MS, Anderson B-YB-YBBBBBBBBCCCDEEEEFFGGGHHHHH. Effects of cognitive enhancement therapy on employment outcomes in early schizophrenia: Results from a 2-year randomized trial. Research on Social Work Practice. 2011;21(1):32-42.

652. Easterly L, McCallion P, Bart BBBBBCCCDDEEEEGHKKLLLMMPPPPRSS. Applying corporate citizenship theory to the operation of affirmative businesses. Journal of Policy and Practice in Intellectual Disabilities. 2010;7(4):261-8.

653. Ebuenyi ID, Syurina EV, Bunders JFG, Regeer BJ. Barriers to and facilitators of employment for people with psychiatric disabilities in Africa: a scoping review. Global Health Action. 2018;11(1):1463658-.

654. Echarti N, Schüring E, O'Donoghue C. Effects of Vocational Re-training on Employment Outcomes Among Persons with Disabilities in Germany: A Quasi-Experiment. Journal Of Occupational Rehabilitation. 2020;30(2):221-34.

655. Eichert HC. Ambulant vocational rehabilitation of persons with mental illness]. Rehabilitation. 2012;51(3):181-8.

656. Eisen SV, Mueller LN, Chang BH, Resnick SG, Schultz MR, Clark JA. Mental health and quality of life among veterans employed as peer and vocational rehabilitation specialists. Psychiatric Services (Washington, DC). 2015;66(4):381-8.

657. El Aissati D, Jackson C. Birmingham early intervention service redesign: Improving recovery and social outcomes for young people with psychosis. Early Intervention in Psychiatry. 2012;6(SUPPL.1):86.

658. Ellenkamp JJH, Brouwers EPM, Embregts PJCM, Joosen MCW, van Weeghel J. Work Environment-Related Factors in Obtaining and Maintaining Work in a Competitive Employment Setting for Employees with Intellectual Disabilities: A Systematic Review. Journal Of Occupational Rehabilitation. 2016;26(1):56-69.

659. Elliott B, Konet R. The Connections Place: A Job Preparedness Program for Individuals with Borderline Personality Disorder. Community Mental Health Journal. 2014;50(1):41-5.

660. Ellison ML, Huckabee SS, Stone RA, Sabella K, Mullen MG. Career services for young adults with serious mental health conditions: Innovations in the field. The Journal of Behavioral Health Services & Research. 2018:No-Specified.

661. Ellison ML, Klodnick VV, Bond GR, Krzos IM, Kaiser SM, Fagan MA, et al. Adapting supported employment for emerging adults with serious mental health conditions. The Journal Of Behavioral Health Services & Research. 2015;42(2):206-22.

662. Ellison ML, Reilly ED, Mueller L, Schultz MR, Drebing CE. A supported education service pilot for returning veterans with posttraumatic stress disorder. Psychological Services. 2018;15(2):200-7.

663. Ellison ML, Rogers ES, Lyass A, Massaro J, Wewiorski NJ, Hsu S-T, et al. Statewide Initiative of Intensive Psychiatric Rehabilitation: Outcomes and Relationship to Other Mental Health Service Use. Psychiatric Rehabilitation Journal. 2011;35(1):9-19.

664. Enayati H, Shaw LA, Golden TP, Anderson CA. Bounding the return on investment and projecting the costs of expanding PROMISE services and activities: Initial insights from PROMISE for policymakers. Journal of Vocational Rehabilitation. 2019;51(2):263-73.

665. Engelbrecht M, van Niekerk L, Coetzee Z, Hajwani Z. Supported Employment for people with mental disabilities in South Africa: cost calculation of service utilisation. South African Journal of Occupational Therapy. 2017;47(2):11-6.

666. Erickson D, Whitehurst D, Roes M, Digiacomo A. "Individual placement and support" provides advantages for early psychosis clients. Early Intervention in Psychiatry. 2018;12(Supplement 1):209.

667. Erickson D, Whitehurst DGT. Individual placement and support provides modest advantages in first six months. Early Intervention in Psychiatry. 2016;10(Supplement 1):154-5.

668. Erickson DH, Roes MM, DiGiacomo A, Burns A. "individual placement and support" boosts employment for early psychosis clients, even when baseline rates are high. Early Intervention in Psychiatry. 2020:No-Specified.

669. Erickson DH, Roes MM, DiGiacomo A, Burns A. "Individual Placement and Support" boosts employment for early psychosis clients, even when baseline rates are high. Early Intervention In Psychiatry. 2021;15(3):662-8.

670. Erickson M, Jaafari N, Lysaker P. Insight and negative symptoms as predictors of functioning in a work setting in patients with schizophrenia. Psychiatry Research. 2011;189(2):161-5.

671. Erlandsson L-K. The Redesigning Daily Occupations (ReDO)-Program: Supporting Women With Stress-Related Disorders to Return to Work—Knowledge Base, Structure, and Content. Occupational Therapy in Mental Health. 2013;29(1):85-101.

672. Essen C. Does individual placement and support really 'reflect client goals'? Journal Of Psychiatric And Mental Health Nursing. 2012;19(3):231-40.

673. Essen C, Alverson BBBBBBBBBBBCCCDDDFGGHKLMNNOHPP. Does individual placement and support really 'reflect client goals'? Journal of Psychiatric and Mental Health Nursing. 2012;19(3):231-40.

674. Eva G, Playford D, Radford K, Burton C. The development of a vocational rehabilitation intervention (the rejoin intervention) to support people with cancer to remain in work. Psycho-Oncology. 2013;22(SUPPL. 3):108-9.

675. Evans E, Hser Y-I, Huang D. Employment Services Utilization and Outcomes among Substance Abusing Offenders Participating in California’s Proposition 36 Drug Treatment Initiative. Journal of Behavioral Health Services & Research. 2010;37(4):461-76.

676. Evensen S, Ueland T, Lystad JU, Bull H, Falkum E. Cost effectiveness of the jobmanagement program (JUMP). Schizophrenia Research. 2014;153(SUPPL. 1):S240-S1.

677. Evensen S, Ueland T, Lystad JU, Bull H, Klungsoyr O, Martinsen EW, et al. Employment outcome and predictors of competitive employment at 2-year follow-up of a vocational rehabilitation programme for individuals with schizophrenia in a high-income welfare society. Nordic Journal of Psychiatry. 2017;71(3):180-7.

678. Evensen S, Wisloff T, Lystad J, Bull H, Martinsen E, Ueland T, et al. Cost-effectiveness of a vocational rehabilitation program for individuals with schizophrenia in a high-income society. European Archives of Psychiatry and Clinical Neuroscience. 2017;267(1 Supplement 1):S10.

679. Evert Cimera R, Gonda J, Vaschak J. Are high schools referring transition-age youth with intellectual disabilities to vocational rehabilitation? A state-by-state analysis. Journal of Vocational Rehabilitation. 2015;42(3):263-70.

680. Fadyl JK, Anstiss D, Reed K, Khoronzhevych M, Levack WMM. Effectiveness of vocational interventions for gaining paid work for people living with mild to moderate mental health conditions: systematic review and meta-analysis. BMJ Open. 2020;10(10):e039699.

681. Fadyl JK, McPherson KM. Approaches to vocational rehabilitation after traumatic brain injury: a review of the evidence. The Journal Of Head Trauma Rehabilitation. 2009;24(3):195-212.

682. Fadyl JK, McPherson KM, Babineau B-YBBCCCCEEF-OGGGGHHHJJJJKKKKKLL. Approaches to vocational rehabilitation after traumatic brain injury: A review of the evidence. The Journal of Head Trauma Rehabilitation. 2009;24(3):195-212.

683. Fagundo E. Older vocational rehabilitation consumers with gainful employment outcomes: correlation of VR services, demographics, and disability characteristics. Dissertation Abstracts International: Section B: The Sciences and Engineering. 2021;82(7-B):No-Specified.

684. Falkum E. Vocational rehabilitation for individuals with schizophrenia-should competitive work be the only goal. European Archives of Psychiatry and Clinical Neuroscience. 2017;267(1 Supplement 1):S10-S1.

685. Falkum E, Klungsøyr O, Lystad JU, Bull HC, Evensen S, Martinsen EW, et al. Vocational rehabilitation for adults with psychotic disorders in a Scandinavian welfare society. BMC Psychiatry. 2017;17(1):24-.

686. Falkum E, Lystad J, Bull H, Evensen S, Ueland T. Helping people with psychotic disorders back to work: The JUMP study. European Archives of Psychiatry and Clinical Neuroscience. 2013;263(1 SUPPL. 1):S65.

687. Falkum E, Lystad JU, Bull H, Evensen S, Klungsoyr O, Ueland T. Vocational rehabilitation for persons with psychotic disorders - The jump study. Schizophrenia Bulletin. 2015;41(SUPPL. 1):S311.

688. Falkum E, Lystad JU, Bull H, Evensen S, Ueland T. Helping people with psychotic disorders back to work the sjump study. Schizophrenia Research. 2014;153(SUPPL. 1):S280.

689. Farabee D, Zhang SX, Wright B, Archibald BBBDFFFJLLLLMMOBPPRRSSTTVVW. An experimental evaluation of a nationally recognized employment-focused offender reentry program. Journal of Experimental Criminology. 2014;10(3):309-22.

690. Fasching H. Vocational education and training and transitions into the labour market of persons with intellectual disabilities. European Journal of Special Needs Education. 2014;29(4):505-20.

691. Fasching H, Baer CDDEEFFGGGGHKKLLLPPPRRRSSSSTWW. Vocational education and training and transitions into the labour market of persons with intellectual disabilities. European Journal of Special Needs Education. 2014;29(4):505-20.

692. Favre C, Spagnoli D, Adewuya BBBCCCDDHHLLLMST. Cognitive and behavioural therapy for management of functional disabilities. Gestion des limitations fonctionnelles par therapie cognitive et comportementale. 2016;26(3):103-10.

693. Favre C, Spagnoli D, Pomini V. Support programme of employment for psychiatric patients: A retrospective study Goal. Schweizer Archiv fur Neurologie und Psychiatrie. 2014;165(7):258-64.

694. Favre C, Spagnoli D, Pomini V, Adewuya BBBBCCCCDDFHHLLMMNSST. Support programme of employment for psychiatric patients: A retrospective study. Dispositif de soutien a l'emploi pour patients psychiatriques: Evaluation retrospective. 2014;165(7):258-64.

695. Featherston LW. Wage differences among closure 26 status competitively employed women and men with disabilities who received VR services from RSA in 2006. 2009(Ph.D.):208 p- p.

696. Ferguson K, Xie B, Glynn S. Adapting the Individual Placement and Support Model with Homeless Young Adults. Child & Youth Care Forum. 2012;41(3):277-94.

697. Ferguson KM. Merging the fields of mental health and social enterprise: lessons from abroad and cumulative findings from research with homeless youths. Community Mental Health Journal. 2012;48(4):490-502.

698. Ferguson KM. Using the Social Enterprise Intervention (SEI) and Individual Placement and Support (IPS) models to improve employment and clinical outcomes of homeless youth with mental illness. Social Work In Mental Health. 2013;11(5).

699. Ferguson KM. From Victims of Market Forces to Entrepreneurs: Rethinking the Role of Supported Employment and Social Entrepreneurship in Behavioral Health Interventions. Human Service Organizations: Management, Leadership & Governance. 2016;40(4):397-409.

700. Ferguson KM. Employment Outcomes From a Randomized Controlled Trial of Two Employment Interventions With Homeless Youth. Journal Of The Society For Social Work And Research. 2018;9(1):1-21.

701. Ferguson KM. Nonvocational Outcomes From a Randomized Controlled Trial of Two Employment Interventions for Homeless Youth. Research on Social Work Practice. 2018;28(5):603-18.

702. Ferguson KM, Achenbach ABBBBBBBBBCCCCCCCDRDDDDDDDEFFF. Merging the fields of mental health and social enterprise: Lessons from abroad and cumulative findings from research with homeless youths. Community Mental Health Journal. 2012;48(4):490-502.

703. Ferguson KM, Achenbach BBBCCCCCDDDDEFFFFFFFFGGGGHKKLL. Using the Social Enterprise Intervention (SEI) and Individual Placement and Support (IPS) models to improve employment and clinical outcomes of homeless youth with mental illness. Social Work in Mental Health. 2013;11(5):473-95.

704. Ferguson KM, Altena AAAB-ABBBBBBBBCCCCDDRDDFFFFFFGG. Employment outcomes from a randomized controlled trial of two employment interventions with homeless youth. Journal of the Society for Social Work and Research. 2018;9(1):1-21.

705. Fernandez HG, Molla CL. What do people with intellectual disabilities think about their career development? Journal of Intellectual Disability Research. 2019;63(7):874.

706. Ferrara M, Guloksuz S, Burke S, Baccari F, Miselli M, Saponaro A, et al. Implementation of a program for early intervention in psychosis onset: The experience of regione Emilia Romagna, Northern Italy. Schizophrenia Bulletin. 2018;44(Supplement 1):S426-S7.

707. Ficarelli ML, Vignali E, Artoni S, Franzini MC, Montanaro S, Andreoli MV, et al. Implementing individual and placement support for patients with severe mental illness: Findings from the real world. Journal of Psychopathology. 2021;27(2):71-80.

708. Fingard J, Rutherford J. Deinstitutionalization and vocational rehabilitation for mental health consumers in Nova Scotia since the 1950s. Histoire Sociale. 2011;44(88):385-408.

709. Fioritti A, Burns T, Hilarion P, van Weeghel J, Cappa C, Suñol R, et al. Individual placement and support in Europe. Psychiatric Rehabilitation Journal. 2014;37(2):123-8.

710. Fioritti A, D'Alema M, Barone R, Bruschetta S. Social enterprises, vocational rehabilitation, supported employment: working on work in Italy. The Journal Of Nervous And Mental Disease. 2014;202(6):498-500.

711. Fioritti A, Peloso PF, Percudani M. “We Can Work It Out”: The Place of Work in Italian Psychosocial Rehabilitation. International Journal of Mental Health. 2016;45(1):51-8.

712. Fitzgerald S, Kimmel K, Locust A, Miller S. Uncovering the early vocational recovery phases for persons with psychiatric disabilities participating in a supported employment program. Journal of Vocational Rehabilitation. 2018;48(1):27-36.

713. Fleming AR, Del Valle R, Kim M, Leahy MJ. Best Practice Models of Effective Vocational Rehabilitation Service Delivery in the Public Rehabilitation Program: A Review and Synthesis of the Empirical Literature. Rehabilitation Counseling Bulletin. 2013;56(3):146-59.

714. Fleming AR, Phillips BN, Kaseroff A, Huck GE. A Qualitative Study of Job Placement Provider Decisions in Vocational Rehabilitation. Rehabilitation Counseling Bulletin. 2014;58(1):7-19.

715. Fleming C, Curtis R, Davis Martin E, Kraska M, Shippen M, Varda K. Perceptions and practices of mental health professionals regarding the employment of people with serious mental illness. Journal of Vocational Rehabilitation. 2019;50(1):39-48.

716. Fleming C, Martin ED, Curtis R, Varda K. Social Role Valorization and Employment of People with the Most Significant Disabilities. Journal of Rehabilitation. 2019;85(3):14-21.

717. Fleury M-J, Grenier G, Bamvita J-M. Predictors of frequent recourse to health professionals by people with severe mental disorders. Canadian Journal Of Psychiatry Revue Canadienne De Psychiatrie. 2015;60(2):77-86.

718. Flippo K, Butterworth J. Community Conversations and Transition Systems Change. Journal of Disability Policy Studies. 2018;29(1):7-11.

719. Flippo KF, Gardner JF, Eidelman. Employment: It is everybody's business. Special Issue: Employment First: An essential strategy. 2011;49(4):305-9.

720. Flores N, Jenaro C, Begoña Orgaz M, Victoria Martín M. Understanding Quality of Working Life of Workers with Intellectual Disabilities. Journal of Applied Research in Intellectual Disabilities. 2011;24(2):133-41.

721. Flyckt L, Msghina M. [Participation is the foundation of successful treatment of psychotic disorders]. Lakartidningen. 2015;112.

722. Foley K, Pallas D, Forcehimes A, Houck J, Bogenschutz M, Keyser-Marcus L, et al. Effect of job skills training on employment and job seeking behaviors in an American Indian substance abuse treatment sample. Journal of Vocational Rehabilitation. 2010;33(3):181-92.

723. Foley SM, Haines K, Mock L, Foley S, Sevak P. Phase II of the SGA Project: Development of the coordinated team approach intervention. Journal of Vocational Rehabilitation. 2020;53(3):273-85.

724. Fong C, Chia-Chiang W, Fitzgerald S, Muller V, Ditchman N, Menz F. Personal, environmental, and service-delivery determinants of employment quality for state vocational rehabilitation consumers: A multilevel analysis. Journal of Vocational Rehabilitation. 2016;45(1):5-18.

725. Fong CJ, Murphy KM, Westbrook JD, Markle MM, Arnold BBBBCCCdBdBFFFFFGGGG-BHHHHHHKLL. Psychological interventions to facilitate employment outcomes for cancer survivors: A systematic review and meta-analysis. Research on Social Work Practice. 2018;28(1):84-98.

726. Forslund MV, Roe C, Arango-Lasprilla JC, Sigurdardottir S, Andelic N. Impact of personal and environmental factors on employment outcome two years after moderate-to-severe traumatic brain injury. Journal of rehabilitation medicine. 2013;45(8):801-7.

727. Fortin G, Corbiere M, Lecomte T. Profiles and correlates of well-being among people with severe mental illness receiving supported employment services : Does getting a job matter? Schizophrenia Bulletin. 2011;37(SUPPL. 1):264.

728. Fortin G, Lecomte T, Corbiere M. Personality traits of people with severe mental illness registered in supported employment programs and their relation to baseline motivation to find a job and job acquisition at 3-month follow-up: Preliminary results. Schizophrenia Research. 2012;136(SUPPL. 1):S269.

729. Fortin G, Lecomte T, Corbière M. Does personality influence job acquisition and tenure in people with severe mental illness enrolled in supported employment programs? Journal Of Mental Health (Abingdon, England). 2017;26(3):248-56.

730. Fossey E, Harvey C, Farhall J, Wiggins A, Ennals P. From evidence to realities: Psychosocial intervention provision in Australian routine community mental health practice. Australian and New Zealand Journal of Psychiatry. 2016;50(Supplement 1):101.

731. Foster C. MITIE: helping long-term unemployed disabled people into work. Equal Opportunities Review. 2010(203):13-5.

732. Fowler D, Hodgekins J, Berry C, Clarke T, Palmier-Claus J, Sacadura C, et al. Social recovery therapy: a treatment manual. Psychosis. 2019;11(3):261-72.

733. Fowler D, Hodgekins J, Wilson J, Birchwood M. Early intervention service non-responders: The case of longstanding social disability. Early Intervention in Psychiatry. 2012;6(SUPPL.1):16.

734. Fraker T, Rangarajan A. The Social Security Administration's youth transition demonstration projects. Journal of Vocational Rehabilitation. 2009;30(3):223-40.

735. Fraker TM, Crane KT, Honeycutt TC, Luecking RG, Mamun AA, O'Day BL. The youth transition demonstration project in Miami, Florida: Design, implementation, and three-year impacts. Journal of Vocational Rehabilitation. 2018;48(1):79-91.

736. Fraker TM, Luecking RG, Mamun AA, Martinez JM, Reed DS, Wittenburg DC, et al. An analysis of 1-year impacts of youth transition demonstration projects. Career Development and Transition for Exceptional Individuals. 2016;39(1):34-46.

737. Franck N. [Cognitive remediation and work outcome in schizophrenia]. L'encephale. 2014;40 Suppl 2:S75-S80.

738. Franck N. Cognitive remediation and work outcome in schizophrenia. Encephale. 2014;40(SUPPL. 2):S75-S80.

739. Franck N, Addington ABBBBBCCdADFFFFFGGHKKLLMMMMMMM. Cognitive remediation and work outcome in schizophrenia. Remediation cognitive et insertion professionnelle dans la schizophrenie. 2014;40(Suppl 2):S75-S80.

740. Frank A. Navigating the health-work interface-vocational rehabilitation in the UK. Occupational Medicine (Oxford, England). 2018;68(1):2-4.

741. Frank RG. Supported employment: evidence on economic impacts. Psychiatric Services (Washington, DC). 2013;64(2):103-.

742. Frank RG. Helping (Some) SSDI beneficiaries with severe mental illness return to work. American Journal of Psychiatry. 2013;170(12):1379-81.

743. Fraser RT, Weber PB, Laxer KD, Post T, Fraser. Considering developing a specialized epilepsy employment program? The PEP Jobs Program paradigm. Epilepsy & Behavior. 2018;82:194-5.

744. Frederick DE, VanderWeele TJ. Supported employment: Meta-analysis and review of randomized controlled trials of individual placement and support. Plos One. 2019;14(2):e0212208-e.

745. Freeman N, Paris T. Employment after brain injury. Journal of Head Trauma Rehabilitation. 2010;25(5):397.

746. Freeze S, Hall AC, Collins S, Shumate D, Thomas C, Brent B. Different states, common issues: Moving mountains one service at a time. Journal of Vocational Rehabilitation. 2017;46(3):265-71.

747. Friedman C, Rizzolo MC. "Get us real jobs:" Supported employment services for people with intellectual and developmental disabilities in Medicaid Home and Community Based Services Waivers. Journal of Vocational Rehabilitation. 2017;46(1):107-16.

748. Friedman C, Rizzolo MC, Bagenstos BBBBCCEEEFFFFFFFFFFFGHJKLMMNNP. "Get us real jobs:" Supported employment services for people with intellectual and developmental disabilities in Medicaid Home and Community Based Services Waivers. Journal of Vocational Rehabilitation. 2017;46(1):107-16.

749. Froud R, Amundsen PA, Bartys S, Battie M, Burton K, Foster NE, et al. Opportunities and challenges around adapting supported employment interventions for people with chronic low back pain: modified nominal group technique. Disability And Rehabilitation. 2020:1-8.

750. Froud R, Grant M, Burton K, Foss J, Ellard DR, Seers K, et al. Development and feasibility of an intervention featuring individual supported work placements to aid return to work for unemployed people living with chronic pain. Pilot And Feasibility Studies. 2020;6:49.

751. Frounfelker R, Teachout A, Bond GR, Drake RE. Criminal justice involvement of individuals with severe mental illness and supported employment outcomes. Community Mental Health Journal. 2011;47(6):737-41.

752. Frounfelker RL, Glover CM, Teachout A, Wilkniss SM, Whitley R. Access to supported employment for consumers with criminal justice involvement. Psychiatric Rehabilitation Journal. 2010;34(1):49-56.

753. Frounfelker RL, Wilkniss SM, Bond GR, Devitt TS, Drake RE. Enrollment in supported employment services for clients with a co-occurring disorder. Psychiatric Services (Washington, DC). 2011;62(5):545-7.

754. Frøyland K. Applicability of IPS principles to job inclusion of vulnerable youth. Journal of Vocational Rehabilitation. 2016;45(3):249-65.

755. Frøyland K. Vital tasks and roles of frontline workers facilitating job inclusion of vulnerable youth. European Journal of Social Work. 2019;22(4):563-74.

756. Fure S, Howe E, Roe C, Hellstrom T, Nordenmark TH, Ugelstad H, et al. Combined Cognitive and Vocational Intervention After Mild-To-Moderate TBI: Baseline Characteristics. Archives of Physical Medicine and Rehabilitation. 2019;100(10):e18.

757. Fure SCR, Howe EI, Andelic N, Brunborg C, Sveen U, Røe C, et al. Cognitive and vocational rehabilitation after mild-to-moderate traumatic brain injury: A randomised controlled trial. Annals Of Physical And Rehabilitation Medicine. 2021;64(5):101538.

758. Furukawa S, Fujieda Y, Shimizu K, Ishibashi A, Eguchi S. [About mental health outreach services in Japan]. Nihon rinsho Japanese journal of clinical medicine. 2013;71(4):718-24.

759. Fyhn T, Ludvigsen K, Reme SE, Schaafsma F. A structured mixed method process evaluation of a randomized controlled trial of Individual Placement and Support (IPS). Implementation science communications. 2020;1:95.

760. Fyhn T, Øverland S, Reme SE. Predictors of employment in people with moderate to severe mental illness participating in a randomized controlled trial of Individual Placement and Support (IPS). The International Journal Of Social Psychiatry. 2021;67(2):150-7.

761. Gabbay M, Taylor L, Sheppard L, Hillage J, Bambra C, Ford F, et al. NICE guidance on long-term sickness and incapacity. British Journal of General Practice. 2011;61(584):e118-e24.

762. Gaillard A, Sultan-Taieb H, Sylvain C, Durand M-J. Economic evaluations of mental health interventions: A systematic review of interventions with work-focused components. Safety Science. 2020;132:104982.

763. Gal G, Shadmi E, Hoter-Ishay G, Gelkopf M, Roe D. Comparing outcome measures of persons with severe mental illness in vocational rehabilitation programs: a dual perspective of consumers and providers. International journal for quality in health care : journal of the International Society for Quality in Health Care. 2021;33(1).

764. Gammelgaard I, Christensen TN, Eplov LF, Jensen SB, Stenager E, Petersen KS. 'I have potential': Experiences of recovery in the individual placement and support intervention. The International Journal Of Social Psychiatry. 2017;63(5):400-6.

765. Gao N, Dolce J, Rio J, Heitzmann C, Loving S. In-vivo job development training among peer providers of homeless veterans supported employment programs. Psychiatric Rehabilitation Journal. 2016;39(2):191-2.

766. Gao N, Dolce JN. A case illustration of strategies to improve employment outcomes among individuals receiving ACT services. American Journal of Psychiatric Rehabilitation. 2010;13(2):94-104.

767. Gao N, Dolce JN, Becker BBDHKKMMRTW. A case illustration of strategies to improve employment outcomes among individuals receiving ACT services. American Journal of Psychiatric Rehabilitation. 2010;13(2):94-104.

768. Gao N, Gill KJ, Schmidt LT, Pratt CW, Abadie LAMBASKRBBBHTEB-MTNMMJDDDABWACR. The application of human capital theory in vocational rehabilitation for individuals with mental illness. Journal of Vocational Rehabilitation. 2010;32(1):25-33.

769. Gao N, Waynor WR, O'Donnell S. Creating organizational commitment to change: key to consumer employment success in a supportive housing agency. Journal of Vocational Rehabilitation. 2009;31(1):45-50.

770. Garcia C, Golay P, Favrod J, Bonsack C. French Translation and Validation of Three Scales Evaluating Stigma in Mental Health. Frontiers In Psychiatry. 2017;8:290-.

771. Garrels V, Sigstad HMH. Employment for persons with intellectual disability in the Nordic countries: A scoping review. Journal of applied research in intellectual disabilities : JARID. 2021;34(4):993-1007.

772. Gehl L. Employment Power. Stanford Social Innovation Review. 2011;9(1):61-2.

773. Gentry T, Kriner R, Sima A, McDonough J, Wehman P. Reducing the Need for Personal Supports Among Workers with Autism Using an iPod Touch as an Assistive Technology: Delayed Randomized Control Trial. Journal of Autism & Developmental Disorders. 2015;45(3):669-84.

774. Gerber LH, Bush H, Cai X, Morse L, Worobey L, Garfinkel S. Scoping review of peer reviewed publications addressing rehabilitation for people sustaining traumatic spinal cord injury. Journal of Spinal Cord Medicine. 2019.

775. Germundsson P, Gustafsson J, Lind M, Danermark B. Disability and supported employment: impact on employment, income, and allowances. International Journal Of Rehabilitation Research Internationale Zeitschrift Fur Rehabilitationsforschung Revue Internationale De Recherches De Readaptation. 2012;35(3):263-9.

776. Gervey R, Gao N, Tillman D, Dickel K, Kneubuehl J. Person-centered employment planning teams: a demonstration project to enhance employment and training outcomes for persons with disabilities accessing the One-Stop Career Center system. Journal of Rehabilitation. 2009;75(2):43-9.

777. Gewurtz RE. Instituting market-based principles within social services for people living with mental illness: The case of the revised ODSP Employment Supports policy. 2011(Ph.D.):278 p- p.

778. Gewurtz RE, Cott C, Rush B, Kirsh B. The shift to rapid job placement for people living with mental illness: an analysis of consequences. Psychiatric Rehabilitation Journal. 2012;35(6):428-34.

779. Gewurtz RE, Cott C, Rush B, Kirsh B. How is unemployment among people with mental illness conceptualized within social policy? A case study of the Ontario Disability Support Program. Work (Reading, Mass). 2015;51(1):121-33.

780. Gewurtz RE, Cott C, Rush B, Kirsh B. How does outcome-based funding affect service delivery? An analysis of consequences within employment services for people living with serious mental illness. Administration And Policy In Mental Health. 2015;42(1):19-28.

781. Giesen JM, Lang AH. Predictors of Earnings Enabling Likely Roll Departure for SSDI Beneficiaries With Visual Impairments in Vocational Rehabilitation. Journal of Disability Policy Studies. 2018;29(3):166-77.

782. Gilbert E, Marwaha S, Milton A, Johnson S, Morant N, Parsons N, et al. Social firms' provision of employment opportunities for people with mental ill health: a UK survey. Mental Health Today. 2013:24-7.

783. Gilman K. Lessons from the trenches: How to make self-employment work for people with disabilities. Work: Journal of Prevention, Assessment & Rehabilitation. 2014;48(1):137-40.

784. Gladh L, Sjölund A. The Validation Process: A useful tool to visualize abilities and enhance the possibilities of paid employment for people with ASD. Journal of Vocational Rehabilitation. 2014;41(1):71-6.

785. Gladman B, Waghorn G. Personal experiences of people with serious mental illness when seeking, obtaining and maintaining competitive employment in Queensland, Australia. Work. 2016;53(4):835-43.

786. Gladman B, Wishart L, Waghorn G, Dias S. Reliability of Health Professionals' Perceptions of Employment for People with Severe Mental Illness. Journal of Rehabilitation. 2015;81(1):3-8.

787. Glover CM, Frounfelker RL. Competencies of Employment Specialists for Effective Job Development. American Journal of Psychiatric Rehabilitation. 2011;14(3):198-211.

788. Glover CM, Frounfelker RL. Competencies of more and less successful employment specialists. Community Mental Health Journal. 2013;49(3):311-6.

789. Glynn K, Schaller J. Predictors of employment outcomes for transition-age state-federal vocational rehabilitation consumers with attention-deficit/hyperactivity disorder. Journal of Vocational Rehabilitation. 2017;47(2):159-74.

790. Glynn K, Schaller J, Alston ABBBB-BBBCCDDF-PFFGGG-KGGHHIJKKKK. Predictors of employment outcomes for transition-age state-federal vocational rehabilitation consumers with attention-deficit/hyperactivity disorder. Journal of Vocational Rehabilitation. 2017;47(2):159-74.

791. Glynn SM, Marder SR, Noordsy DL, O'Keefe C, Becker DR, Drake RE, et al. An RCT Evaluating the Effects of Skills Training and Medication Type on Work Outcomes Among Patients With Schizophrenia. Psychiatric Services (Washington, DC). 2017;68(3):271-7.

792. Gmitroski T, Bradley C, Heinemann L, Liu G, Blanchard P, Beck C, et al. Barriers and facilitators to employment for young adults with mental illness: a scoping review. BMJ Open. 2018;8(12):e024487-e.

793. Gödecker-Geenen N, Riedel HP, Keck T. [Developing the integration-oriented participation into working life]. Die Rehabilitation. 2013;52(2):126-31.

794. Godwin K. Coaching people with learning disabilities into work. Equal Opportunities Review. 2010(203):10-2.

795. Goetz LL, Ottomanelli LA, Barnett SD. Multicenter trial of supported employment for veterans with spinal cord injury. Topics in Spinal Cord Injury Rehabilitation. 2013;19(1):15.

796. Gold J. A-Visions: a Scripps supported employment program. Journal of Vocational Rehabilitation. 2010;32(1):47-59.

797. Gold J, Lopez DBCPKDK. A-Visions: A Scripps supported employment program. Journal of Vocational Rehabilitation. 2010;32(1):47-59.

798. Gold PB. Quality of life and competitive work among adults with severe mental illness: moderating effects of family contact. Psychiatric Services (Washington, DC). 2013;64(12):1218-24.

799. Gold PB, Aschbrenner BBBCCCCCDFGGGHKKKLLLMMMRSTTVW. Quality of life and competitive work among adults with severe mental illness: Moderating effects of family contact. Psychiatric Services. 2013;64(12):1218-24.

800. Gold PB, Fabian ES, Luecking RG. Job Acquisition by Urban Youth With Disabilities Transitioning From School to Work. Rehabilitation Counseling Bulletin. 2013;57(1):31-45.

801. Gold PB, Macias C, Barreira PJ, Tepper M, Frey J. Viability of Using Employment Rates from Randomized Trials as Benchmarks for Supported Employment Program Performance. Administration and Policy in Mental Health and Mental Health Services Research. 2009:1-6.

802. Gold PB, Macias C, Barreira PJ, Tepper M, Frey J. Viability of using employment rates from randomized trials as benchmarks for supported employment program performance. Administration And Policy In Mental Health. 2010;37(5):427-32.

803. Gold PB, Macias C, Rodican CF. Does Competitive Work Improve Quality of Life for Adults with Severe Mental Illness? Evidence from a Randomized Trial of Supported Employment. The Journal Of Behavioral Health Services & Research. 2016;43(2):155-71.

804. Goldberg RW, Resnick SG. US Department of Veterans Affairs (VA) efforts to promote psychosocial rehabilitation and recovery. Psychiatric Rehabilitation Journal. 2010;33(4):255-8.

805. Goldman HH, Frey WD, Riley JK. Social Security and Disability Due to Mental Impairment in Adults. Annual Review Of Clinical Psychology. 2018;14:453-69.

806. Goods N, Millsteed J. Understanding retirement for ageing adults with a disability in supported employment. British Journal of Occupational Therapy. 2016;79(11):713-21.

807. Gore N, Forrester-Jones R, Young R. Supported Employment: Not re-inventing the wheel, just moving it in a realistic direction. Journal of Intellectual Disability Research. 2012;56(7-8):711.

808. Gore NJ, Forrester-Jones R, Young R. Staff experiences of supported employment with the Sustainable Hub of Innovative Employment for people with Complex needs. British Journal of Learning Disabilities. 2014;42(3):228-35.

809. Graffam J, Shinkfield AJ, Lavelle B. Recidivism among participants of an employment assistance program for prisoners and offenders. International Journal of Offender Therapy & Comparative Criminology. 2014;58(3):348-63.

810. Gray HM, Nelson SE, Shaffer HJ, Stebbins P, Farina AR, Chen CCDDEFFGHHKLMNRRSST. How do homeless adults change their lives after completing an intensive job-skills program? A prospective study. Journal of Community Psychology. 2017;45(7):888-905.

811. Greenberg D, Walter J, Knight G. A cost-benefit analysis of the random assignment UK Employment Retention and Advancement Demonstration. Applied Economics. 2013;45(31):4335-54.

812. Grigal M, Hart D, Migliore A, Agran ABBBBBBCCCdDDDGGGGGGGHHHHKKKLM. Comparing the transition planning, postsecondary education, and employment outcomes of students with intellectual and other disabilities. Career Development for Exceptional Individuals. 2011;34(1):4-17.

813. Grigorovich A, Stergiou-Kita M, Damianakis T, Le Dorze G, Lemsky C, Hebert D. Persons with brain injury and employment supports: Long-term employment outcomes and use of community-based services. Brain Injury. 2017;31(5):607-19.

814. Grove B. International employment schemes for people with mental health problems. Bjpsych International. 2015;12(4):97-9.

815. Gruman C, Shugrue NA, Koppelman J, Schimmel J, Porter A, Robison JT. The Impact of Benefits Counseling and Vocational Rehabilitation on Employment and Earnings. Journal of Rehabilitation. 2014;80(3):21-9.

816. Guevara E, Valois M, Winkelmann I, Malla A, Joober R, Goldberg K, et al. Housing for recovery. Early Intervention in Psychiatry. 2016;10(Supplement 1):141.

817. Guhne U, Hoffmann H, Stengler K, Becker T, Riedel-Heller SG, Areberg ABBBBBBCCCDFFFGGHHHIKiKKLLMMMM. Supported Employment in severe mental illness. Further developments. Supported Employment bei schweren psychischen Erkrankungen: Neuentwicklungen im Feld. 2015;12(4):220-6.

818. Gühne U, Weinmann S, Arnold K, Becker T, Riedel-Heller SG. S3 guideline on psychosocial therapies in severe mental illness: evidence and recommendations. European Archives Of Psychiatry And Clinical Neuroscience. 2015;265(3):173-88.

819. Guhne U, Weinmann S, Arnold K, Becker T, Riedel-Heller SG, Albus AABBBBBBBBBBBBBBBCCCCCCCCDHDDD. S3 guideline on psychosocial therapies in severe mental illness: Evidence and recommendations. European Archives of Psychiatry and Clinical Neuroscience. 2015;265(3):173-88.

820. Guhne U, Weinmann S, Riedel-Heller SG, Becker T, Alvarez-Jimenez BBBBBBBBCCCCCCCCCCDDDFFGF-S-MFF. Psychosocial therapies in severe mental illness: Update on evidence and recommendations. Current Opinion in Psychiatry. 2020;33(4):414-21.

821. Gustafsson J, Peralta J, Danermark B. Supported Employment and Social Inclusion - Experiences of Workers with Disabilities in Wage Subsidized Employment in Sweden. Scandinavian Journal of Disability Research. 2018;20(1):26-36.

822. Gustafsson J, Peralta JP, Danermark B. The employer's perspective on Supported employment for people with disabilities: Successful approaches of Supported employment organizations. Journal of Vocational Rehabilitation. 2013;38(2):99-111.

823. Gutman SA, Brown T. A Bibliometric Analysis of the Quantitative Mental Health Literature in Occupational Therapy. Occupational Therapy in Mental Health. 2018;34(4):305-46.

824. Guy EJ. Factors associated with both successful and unsuccessful vocational rehabilitation case closures of Navajo people with disabilities: a qualitative study. 2009(Ph.D.):114 p- p.

825. Hackman A. RA1SE Connection: A First-Episode Intervention. Journal of the American Academy of Child and Adolescent Psychiatry. 2018;57(10 Supplement):S76-S7.

826. Hagglund P. Experimental evidence from active placement efforts among unemployed in Sweden. Evaluation review. 2014;38(3):191-216.

827. Hagner D, Dague B, Phillips K, Attridge BBCFFFGGGGHHHIKKMMNPSWZ. Including employees with disabilities in workplace cultures: Strategies and barriers. Rehabilitation Counseling Bulletin. 2015;58(4):195-202.

828. Hagner D, Dague B, Phillips KJ. Implementation of an Employment Consultation Model of Job Support Following Online Training. Journal of Rehabilitation. 2014;80(4):19-27.

829. Hall AC, Butterworth J, Winsor J, Kramer J, Nye-Lengerman K, Timmons J. Building an Evidence-Based, Holistic Approach to Advancing Integrated Employment. Research & Practice for Persons with Severe Disabilities. 2018;43(3):207-18.

830. Hall AC, Freeze S, Butterworth J, Hoff D. Employment funding for intellectual/developmental disability systems. Journal of Vocational Rehabilitation. 2011;34(1):1-15.

831. Halling G, Isernhagen SJ. Back to Work: Important Roles for Rehab. Rehab Management: The Interdisciplinary Journal of Rehabilitation. 2013;26(1):30-5.

832. Ham W, McDonough J, Molinelli A, Schall C, Wehman P. Employment supports for young adults with autism spectrum disorder: Two case studies. Journal of Vocational Rehabilitation. 2014;40(2):117-24.

833. Hamilton AB, Cohen AN, Glover DL, Whelan F, Chemerinski E, McNagny KP, et al. Implementation of evidence-based employment services in specialty mental health. Health Services Research. 2013;48(6 Pt 2):2224-44.

834. Hamilton IS, Schneider J, Kane E, Jordan M. Employment of ex-prisoners with mental health problems, a realistic evaluation protocol. BMC Psychiatry. 2015;15:185-.

835. Hammel Y, Kilian H, Schafer F, Bond HHTWW. Vocational rehabilitation for mentally disabled persons. Berufliche Rehabilitation psychisch Kranker. 2013;32(6):358-62.

836. Hanisch SE, Wrynne C, Weigl M. Perceived and actual barriers to work for people with mental illness. Journal of Vocational Rehabilitation. 2017;46(1):19-30.

837. Hardonk S, Halldórsdóttir S. Work Inclusion through Supported Employment? Perspectives of Job Counsellors in Iceland. Scandinavian Journal of Disability Research. 2021;23(1):39-49.

838. Harker B, Desenberg-Wines A. Iowa Coalition for Integrated Employment. Journal of Vocational Rehabilitation. 2017;47(3):317-26.

839. Harley DA, Abdullah ABBCCCCCDDDEEFFFFGGGGHHHHHJKKK. Adult ex-offender population and employment: A synthesis of the literature on recommendations and best practices. Journal of Applied Rehabilitation Counseling. 2014;45(3):10-21.

840. Harris AW, Kosic T, Xu J, Walker C, Gye W, Redoblado Hodge A. Web-Based Cognitive Remediation Improves Supported Employment Outcomes in Severe Mental Illness: Randomized Controlled Trial. JMIR Mental Health. 2017;4(3):e30-e.

841. Harris AWF, Kosic T, Walker C, Gye W, Redoblado-Hodge A. Internet based cognitive remediation can assist people with severe mental illness to gain and retain employment - The cogrem study. Schizophrenia Bulletin. 2015;41(SUPPL. 1):S314.

842. Harris AWF, Marsh PJ. Personalizing social and cognitive remediation-is the whole greater than the sum of the parts? Schizophrenia Research. 2012;136(SUPPL. 1):S38.

843. Harris C, Switzer E, Gower WS. The Diversity Partners Project: Multi-systemic knowledge translation and business engagement strategies to improve employment of people with disabilities. Journal of Vocational Rehabilitation. 2017;46(3):273-85.

844. Harris LM, Matthews LR, Penrose-Wall J, Alam A, Jaworski A, Ades ABBBBBCCCCCC-RDDDEFFGHH-GHKLLLLL. Perspectives on barriers to employment for job seekers with mental illness and additional substance-use problems. Health & Social Care in the Community. 2014;22(1):67-77.

845. Harris M, Cleary C, King J, Waghorn G. Development and preliminary evaluation of an employment resource for mental health service consumers, families and carers, and clinicians. Australian e-Journal for the Advancement of Mental Health. 2009;8(1):1-15.

846. Harris SP, Owen R, Jones R, Caldwell K. Does workfare policy in the United States promote the rights of people with disabilities? Journal of Vocational Rehabilitation. 2013;39(1):61-73.

847. Harris SP, Renko M, Caldwell K. Accessing social entrepreneurship: Perspectives of people with disabilities and key stakeholders. Journal of Vocational Rehabilitation. 2013;38(1):35-48.

848. Harrison J, Krieger MJ, Johnson HA. Review of Individual Placement and Support Employment Intervention for Persons with Substance Use Disorder. Substance Use & Misuse. 2019:1-8.

849. Harrison J, Krieger MJ, Johnson HA. Review of Individual Placement and Support Employment Intervention for Persons with Substance Use Disorder. Substance Use & Misuse. 2020;55(4):636-43.

850. Harrison J, Krieger MJ, Johnson HA, Aklin BBBBBBBBBBCCCCDDDDDEFFGHHJKLLL. Review of individual placement and support employment intervention for persons with substance use disorder. Substance Use & Misuse. 2019:No-Specified.

851. Harrison J, Krieger MJ, Johnson HA, Aklin BBBBBBBBBBCCCCDDDDDEFFGHHJKLLL. Review of individual placement and support employment intervention for persons with substance use disorder. Substance Use & Misuse. 2020;55(4):636-43.

852. Hart T, Dijkers M, Whyte J, Braden C, Trott CT, Fraser R. Vocational interventions and supports following job placement for persons with traumatic brain injury. Journal of Vocational Rehabilitation. 2010;32(3):135-50.

853. Hart T, Dijkers M, Whyte J, Braden C, Trott CT, Fraser R, et al. Vocational interventions and supports following job placement for persons with traumatic brain injury. Journal of Vocational Rehabilitation. 2010;32(3):135-50.

854. Hartman E, Schlegelmilch A, Roskowski M, Anderson CA, Tansey TN, Golden TP. Early findings from the Wisconsin PROMISE project: Implications for policy and practice. Journal of Vocational Rehabilitation. 2019;51(2):167-81.

855. Hartnett HP, Thurman H, Cordingly K. Individuals' perceptions of employment accommodation decisions and solutions: Lessons for social workers. Journal of Social Work in Disability and Rehabilitation. 2010;9(1):53-68.

856. Harvey C, Farhall J, Lewis J, Stain H. Evidence-based psychosocial interventions for australians living with psychoses: Epidemiological data on receipt, targeting and explanatory factors. Australian and New Zealand Journal of Psychiatry. 2016;50(Supplement 1):101.

857. Harvey C, Lewis J, Farhall J. Receipt and targeting of evidence-based psychosocial interventions for people living with psychoses: findings from the second Australian national survey of psychosis. Epidemiology and Psychiatric Sciences. 2018:1-17.

858. Harvey C, Lewis J, Farhall J. Receipt and targeting of evidence-based psychosocial interventions for people living with psychoses: findings from the second Australian national survey of psychosis. Epidemiology And Psychiatric Sciences. 2019;28(6):613-29.

859. Harvey J, Szoc R, Dela Rosa M, Pohl M, Jenkins J. Understanding the competencies needed to customize jobs: A competency model for customized employment. Journal of Vocational Rehabilitation. 2013;38(2):77-89.

860. Harvey SB, Henderson M. Occupational psychiatry. Psychiatry. 2009;8(5):174-8.

861. Harvey SB, Modini M, Christensen H, Glozier N. Severe mental illness and work: what can we do to maximise the employment opportunities for individuals with psychosis? The Australian And New Zealand Journal Of Psychiatry. 2013;47(5):421-4.

862. Haslett WR, Drake RE, Bond GR, Becker DR, McHugo GJ. Individual Placement and Support: Does Rurality Matter? American Journal of Psychiatric Rehabilitation. 2011;14(3):237-44.

863. Haslett WR, McHugo GJ, Bond GR, Drake RE. Use of Software for Tablet Computers to Promote Engagement With Supported Employment: Results From an RCT. Psychiatric Services (Washington, DC). 2014;65(7):954-6.

864. Hasson H. Systematic evaluation of implementation fidelity of complex interventions in health and social care. Implementation Science: IS. 2010;5:67-.

865. Hasson H, Andersson M, Bejerholm U. Barriers in implementation of evidence-based practice: Supported employment in Swedish context. Journal Of Health Organization And Management. 2011;25(3):332-45.

866. Hasson-Ohayon I, Roe D, Yanos PT, Lysaker PH. The trees and the forest: mixed methods in the assessment of recovery based interventions' processes and outcomes in mental health. Journal Of Mental Health (Abingdon, England). 2016;25(6):543-9.

867. Hatton C. Paid employment amongst adults with learning disabilities receiving social care in England: trends over time and geographical variation. Tizard Learning Disability Review. 2018;23(2):117-22.

868. Haugli L, Øyeflaten I. The nature of the working alliance: building understanding in all fields...Waghorn G, De Souza T, Rampton N, et al [including commentary by Watzke S]. The working alliance in supported employment for people with severe mental health problems. Int J Ther Rehabil. 2009 Jun;16(6):315-23. International Journal of Therapy & Rehabilitation. 2009;16(7):402-.

869. Hayashi T, Yamaguchi S, Sato S. Implementing the individual placement and support model of supported employment in Japan: Barriers and strategies. Psychiatric Rehabilitation Journal. 2019.

870. Hayashi T, Yamaguchi S, Sato S. Implementing the individual placement and support model of supported employment in Japan: Barriers and strategies. Psychiatric Rehabilitation Journal. 2020;43(1):53-9.

871. Hayes L, Brophy L, Harvey C, Tellez JJ, Herrman H, Killackey E. Enabling choice, recovery and participation: evidence-based early intervention support for psychosocial disability in the National Disability Insurance Scheme. Australasian Psychiatry: Bulletin Of Royal Australian And New Zealand College Of Psychiatrists. 2018;26(6):578-85.

872. Hay-Smith EJC, Dickson B, Nunnerley J, Anne Sinnott K. 'The final piece of the puzzle to fit in': an interpretative phenomenological analysis of the return to employment in New Zealand after spinal cord injury. Disability & Rehabilitation. 2013;35(17):1436-46.

873. Heath KL, Reed DL, Baker CCGHIKLODPRSSSSSVWWW. Industry-driven support (IDS) model to build social capital and business skills of low-income entrepreneurs with disabilities. Journal of Vocational Rehabilitation. 2013;38(2):139-48.

874. Heath KL, Ward KM, Reed DL, Arnold CCCGGGMPRSWY. Customized self-employment and the use of Discovery for entrepreneurs with disabilities. Journal of Vocational Rehabilitation. 2013;39(1):23-7.

875. Hedger S. Wage assessment tools for Australian Disability Enterprises and supported employees. Keeping Good Companies (14447614). 2012;64(6):354-6.

876. Hedley D, Bury S, Uljarevic M, Dissanayake C. Predictors of mental health and well-being in recently employed adults with autism spectrum disorder. Journal of Intellectual Disability Research. 2019;63(7):652.

877. Hedley D, Cai R, Uljarevic M, Wilmot M, Spoor JR, Richdale A, et al. Transition to work: Perspectives from the autism spectrum. Autism: The International Journal Of Research And Practice. 2018;22(5):528-41.

878. Hedley D, Dissanayake C, Richdale A, Spoor J, Uljarevic M. Long-term benefits of supported employment for adults with autism spectrum disorder. Journal of Intellectual Disability Research. 2016;60(7-8):679.

879. Hedley D, Spoor JR, Uljarevich M, Wilmot M, Cai R, Moss S, et al. Work Experiences and Well-being in Employees with Autism Spectrum Disorder. Academy of Management Annual Meeting Proceedings. 2017;2017(1):1-.

880. Hedley D, Uljarević M, Bury SM, Dissanayake C. Predictors of mental health and well-being in employed adults with autism spectrum disorder at 12-month follow-up. Autism Research: Official Journal Of The International Society For Autism Research. 2019;12(3):482-94.

881. Hedley D, Uljarević M, Cameron L, Halder S, Richdale A, Dissanayake C. Employment programmes and interventions targeting adults with autism spectrum disorder: A systematic review of the literature. Autism: The International Journal Of Research And Practice. 2017;21(8):929-41.

882. Heffernan J, Pilkington P. Supported employment for persons with mental illness: systematic review of the effectiveness of individual placement and support in the UK. Journal Of Mental Health (Abingdon, England). 2011;20(4):368-80.

883. Hegelstad WTV, Hatloy K, Heitmann L, Johannessen JO. Earning and learning: Two sides of the same coin. Testing of an integrated IPS fidelity scale for education and employment in Norway. Early Intervention in Psychiatry. 2016;10(Supplement 1):56.

884. Hegelstad WTV, Joa I, Heitmann L, Johannessen JO, Langeveld J. Job- and schoolprescription: A local adaptation to individual placement and support for first episode psychosis. Early Intervention In Psychiatry. 2019;13(4):859-66.

885. Heinemann AW, Moore D, Lazowski LE, Huber M, Semik P. Benefits of Substance Use Disorder Screening on Employment Outcomes in State–Federal Vocational Rehabilitation Programs. Rehabilitation Counseling Bulletin. 2014;57(3):144-58.

886. Heitmann L, Grini I, Fjeldeoye L, Ten Velden Hegelstad W, Langeveld J. Job-Prescription: A vocational rehabilitation program for people with severe mental illness in South-West Norway. Early Intervention in Psychiatry. 2010;4(SUPPL. 1):147.

887. Heitmann L, Oye LF, Steinsland SS, Olsson LR, Ten Velden Hegelstad W. Job-Prescription: A vocational rehabilitation program for young people with severe mental illness in south-west Norway. Early Intervention in Psychiatry. 2012;6(SUPPL.1):90.

888. Hellström L, Bech P, Hjorthøj C, Nordentoft M, Lindschou J, Eplov LF. Effect on return to work or education of Individual Placement and Support modified for people with mood and anxiety disorders: results of a randomised clinical trial. Occupational And Environmental Medicine. 2017;74(10):717-25.

889. Hellström L, Bech P, Nordentoft M, Lindschou J, Eplov LF. The effect of IPS-modified, an early intervention for people with mood and anxiety disorders: study protocol for a randomised clinical superiority trial. Trials. 2013;14:442-.

890. Hellström L, Madsen T, Nordentoft M, Bech P, Eplov LF. Trajectories of Return to Work Among People on Sick Leave with Mood or Anxiety Disorders: Secondary Analysis from a Randomized Controlled Trial. Journal Of Occupational Rehabilitation. 2018;28(4):666-77.

891. Hellström L, Pedersen P, Christensen TN, Wallstroem IG, Bojesen AB, Stenager E, et al. Vocational Outcomes of the Individual Placement and Support Model in Subgroups of Diagnoses, Substance Abuse, and Forensic Conditions: A Systematic Review and Analysis of Pooled Original Data. Journal Of Occupational Rehabilitation. 2021.

892. Hemmeter J, Donovan M, Cobb J, Asbury T. Long term earnings and disability program participation outcomes of the Bridges transition program. Journal of Vocational Rehabilitation. 2015;42(1):1-15.

893. Hemphill E, Kulik CT, Allen BBBBBBBBCCCCCCDDFFFGGGGHHHJJJK. Help wanted: People with disabilities and recruitment advertising. Journal of Employment Counseling. 2016;53(2):71-85.

894. Henry AD, Hashemi L, Zhang J. Evaluation of a statewide implementation of supported employment in Massachusetts. Psychiatric Rehabilitation Journal. 2014;37(4):284-8.

895. Herbig B, Glaser J, Angerer P. Old, sick, unemployed, without a chance? Results of a randomised controlled trial of the effects of a combined health and employment promotion program for the older long-term unemployed (AmigA-M). Bundesgesundheitsblatt - Gesundheitsforschung - Gesundheitsschutz. 2012;55(8):970-9.

896. Hergenrather KC, Gitlin DJ, Rhodes SD. Consumers with a Bipolar Disorder: A Theory-based Approach to Explore Beliefs Impacting Job Placement. Journal of Rehabilitation. 2011;77(3):14-24.

897. Hergenrather KC, Haase E, Zeglin RJ, Rhodes SD. Consumers With Major Depressive Disorder: Factors Influencing Job Placement. Rehabilitation Research, Policy & Education. 2013;27(2):108-23.

898. Hergenrather KC, Rhodes SD, Haase E. Consumers with Major Depressive Disorder and Employment Outcomes: Application of the Theory of Planned Behavior. Journal of Rehabilitation. 2013;79(1):19-29.

899. Herring S. The westend coop: Integrating a therapeutic small business model into outpatient cognitive and behavioral rehabilitation. Journal of Head Trauma Rehabilitation. 2011;26(5):418-9.

900. Herron JD, Gioia D, Dohrn B. A social enterprise model for employment. Psychiatric Services (Washington, DC). 2009;60(8):1140-.

901. Heugten HCV, Lagerveld S, Van Der Heijden P, Groenendaal E, Van De Pol P. Individual placement and support (IPS). Tijdschrift voor Psychiatrie. 2019;61(9):664-5.

902. Hielscher E, Waghorn G. Managing disclosure of personal information: An opportunity to enhance supported employment. Psychiatric Rehabilitation Journal. 2015;38(4):306-13.

903. Hilarión P, Koatz D, Bonet P, Cid J, Pinar I, Otín JM, et al. Implementation of the individual placement and support pilot program in Spain. Psychiatric Rehabilitation Journal. 2020.

904. Hill DA, Belcher L, Brigman HE, Renner S, Stephens B, Beard BCCCDDJDDFFFGGGGHHHJKLLLMMMMRR. The Apple iPad TM as an innovative employment support for young adults with autism spectrum disorder and other developmental disabilities. Journal of Applied Rehabilitation Counseling. 2013;44(1):28-37.

905. Hill JC. Zebras in the workplace: Vocational rehabilitation considerations for individuals with Ehlers-Danlos Syndrome. Journal of Vocational Rehabilitation. 2017;47(2):197-206.

906. Hill JC, Barlow BBBBBCCCDKKKKLLMRRRST. Zebras in the workplace: Vocational rehabilitation considerations for individuals with Ehlers-Danlos syndrome. Journal of Vocational Rehabilitation. 2017;47(2):197-206.

907. Hilton G, Unsworth CA, Murphy GC, Browne M, Olver J. Longitudinal employment outcomes of an early intervention vocational rehabilitation service for people admitted to rehabilitation with a traumatic spinal cord injury. Spinal Cord. 2017;55(8):743-52.

908. Hoch JS, Dewa CS. Advantages of the net benefit regression framework for economic evaluations of interventions in the workplace: a case study of the cost-effectiveness of a collaborative mental health care program for people receiving short-term disability benefits for psychiatric disorders. Journal Of Occupational And Environmental Medicine. 2014;56(4):441-5.

909. Hoeffding LK, Nielsen MH, Rasmussen MA, Norup A, Arango-Lasprilla JC, Kjær UK, et al. A manual-based vocational rehabilitation program for patients with an acquired brain injury: study protocol of a pragmatic randomized controlled trial (RCT). Trials. 2017;18(1):371-.

910. Hoeffding LK, Nielsen MH, Rasmussen MA, Norup A, Arango-Lasprilla JC, Kjær UK, et al. A manual-based vocational rehabilitation program for patients with an acquired brain injury: study protocol of a pragmatic randomized controlled trial (RCT). Trials. 2017;19:1-9.

911. Hoffman D, Hemmeter J, Bailey MS. The relationship between youth services and adult outcomes among former child SSI recipients. Journal of Vocational Rehabilitation. 2018;48(2):233-47.

912. Hoffmann H. [Vocational rehabilitation: first place, then train--pro]. Psychiatrische Praxis. 2014;41(6):293-4.

913. Hoffmann H, Becker BBBBBBBBCCDDDGHHHHHJJKKLLMMNTW. What makes supported employment so superior? Was macht Supported Employment so uberlegen? 2013;10(2):95-101.

914. Hoffmann H, Jäckel D, Glauser S, Kupper Z. A randomised controlled trial of the efficacy of supported employment. Acta Psychiatrica Scandinavica. 2012;125(2):157-67.

915. Hoffmann H, Richter D. Supported Employment-and Switzerland. Supported employment und die Schweiz. 2018;169(6):181-2.

916. Hoffmann H, Richter D. Supported employment in Switzerland-Are we on track? Psychiatric Rehabilitation Journal. 2019.

917. Hoffmann H, Richter D. Supported employment in Switzerland-Are we on track? Psychiatric Rehabilitation Journal. 2020;43(1):72-5.

918. Höhl W. Jobcoaching in der Ergotherapie. Ergotherapie & Rehabilitation. 2017;56(1):20-4.

919. Höhl W, Kornwinkel S. New possibilities for work integration through OT OT-assisted work opportunities for the disabled with reference to ALGII. Ergotherapie & Rehabilitation. 2010;49(9):16-21.

920. Holmas TH, Monstad K, Reme SE, Abadie BBCCCCDDEGGGGHKLMMRRRSS. Regular employment for people with mental illness-An evaluation of the individual placement and support programme. Social Science & Medicine. 2021;270.

921. Holmes MM, Stanescu SC, Linaker C, Price C, Maguire N, Fraser S, et al. Exploring the views of stakeholders about the feasibility of carrying out a randomised controlled trial of Individual Placement and Support for people unemployed with chronic pain based in primary care (the InSTEP study). Pilot And Feasibility Studies. 2020;6:44.

922. Holmes MM, Stanescu SC, Linaker C, Price C, Maguire N, Fraser S, et al. Individualised placement support as an employment intervention for individuals with chronic pain: a qualitative exploration of stakeholder views. BJGP open. 2020;4(3).

923. Holmes-Langstone D. Cefndy: a great example of 'supported employment'. Equipment Services. 2011:90-1.

924. Holttum S. Research watch. Mental Health & Social Inclusion. 2011;15(2):49-53.

925. Holttum S. Improving social inclusion for young people diagnosed with 'first episode psychosis': employment, education and online support. Mental Health & Social Inclusion. 2013;17(3):112-7.

926. Holtyn AF, Toegel F, Novak MD, Silverman K. Factors associated with obtaining employment among opioid use disorder patients enrolled in a therapeutic workplace intervention. Drug and Alcohol Dependence. 2021;226:108907.

927. Holtyn AF, Toegel F, Subramaniam S, Arellano M, Leoutsakos J-M, Fingerhood M, et al. Financial incentives promote engagement in employment services for unemployed adults in treatment for opioid use disorder. Drug And Alcohol Dependence. 2020;212:107982.

928. Holtyn AF, Toegel F, Subramaniam S, Jarvis BP, Leoutsakos J-M, Fingerhood M, et al. Abstinence-contingent wage supplements to promote drug abstinence and employment: a randomised controlled trial. Journal of epidemiology and community health. 2020;74(5):445-52.

929. Holwerda A, Fokkens AS, Engbers C, Brouwer S. Collaboration between mental health and employment services to support employment of individuals with mental disorders. Disability And Rehabilitation. 2016;38(13):1250-6.

930. Hönig A. [Job placement for people with severe mental illness. The group experience and employment support in a community mental health institution in Buenos Aires]. Vertex (Buenos Aires, Argentina). 2015;26(124):435-40.

931. Hornik-Lurie T, Zilber N, Lerner Y. Trends in the use of rehabilitation services in the community by people with mental disabilities in Israel; the factors involved. Israel Journal of Health Policy Research. 2012;1(1):24.

932. Hoste T, Jurcak SE. Pathway to employment-The successful vocational rehabilitation process. Brain Injury. 2014;28(5-6):759.

933. Hou WH, Chi CC, Lo HL, Chou YY, Kuo KN, Chuang HY. Vocational rehabilitation for enhancing return-to-work in workers with traumatic upper limb injuries. Cochrane Database of Systematic Reviews. 2017;2017(12):CD010002.

934. Houtenville AJ, Brucker DL, Aaron BBBBBBBBCEGHHKLLLMMMSSSSSWWWWW. Participation in safety-net programs and the utilization of employment services among working-age persons with disabilities. Journal of Disability Policy Studies. 2014;25(2):91-105.

935. Hoven H, Ford R, Willmot A, Hagan S, Siegrist J. Job Coaching and Success in Gaining and Sustaining Employment Among Homeless People. Research on Social Work Practice. 2016;26(6):668-74.

936. Howard L, de Salis I, Tomlin Z, Thornicroft G, Donovan J. Why is recruitment to trials difficult? An investigation into recruitment difficulties in an RCT of supported employment in patients with severe mental illness. Contemporary Clinical Trials. 2009;30(1):40-6.

937. Howard-Wilsher S, Irvine L, Fan H, Shakespeare T, Suhrcke M, Horton S, et al. Systematic overview of economic evaluations of health-related rehabilitation. Disability And Health Journal. 2016;9(1):11-25.

938. Howe EI, Fure SCR, Løvstad M, Enehaug H, Sagstad K, Hellstrøm T, et al. Effectiveness of Combining Compensatory Cognitive Training and Vocational Intervention vs. Treatment as Usual on Return to Work Following Mild-to-Moderate Traumatic Brain Injury: Interim Analysis at 3 and 6 Month Follow-Up. Frontiers in neurology. 2020;11:561400.

939. Howe EI, Langlo K-PS, Terjesen HCA, Røe C, Schanke A-K, Søberg HL, et al. Combined cognitive and vocational interventions after mild to moderate traumatic brain injury: study protocol for a randomized controlled trial. Trials. 2017;18(1):483-.

940. Hsiu-shan Y, Wan IL. Disability employment services under new public management: A comparison of Australia and Taiwan. International Social Work. 2018;61(3):437-50.

941. Hsu T-H, Huang Y-T, Liu Y-H, Ososkie J, Fried J, Bezyak J. Taiwanese attitudes and affective reactions toward individuals and coworkers who have intellectual disabilities. American Journal On Intellectual And Developmental Disabilities. 2015;120(2):110-24.

942. Huang IC, Holzbauer JJ, Lee E-J, Chronister J, Chan F, O'Neil J. Vocational rehabilitation services and employment outcomes for adults with cerebral palsy in the United States. Developmental Medicine And Child Neurology. 2013;55(11):1000-8.

943. Huang IC, Wang Y-T, Chan F. Employment outcomes of adults with cerebral palsy in Taiwan. Disability And Rehabilitation. 2013;35(3):228-35.

944. Huang IC, Wang Y-T, Chan F, Andersson BBBBBBDHHIJKKLLLLM-EMMMMNNNOOPP. Employment outcomes of adults with cerebral palsy in Taiwan. Disability and Rehabilitation: An International, Multidisciplinary Journal. 2013;35(3):228-35.

945. Hughes Jr C. Mississippi Partnerships for Employment: Collaborating for systems change. Journal of Vocational Rehabilitation. 2017;47(3):327-35.

946. Hult M, Lappalainen K, Saaranen TK, Räsänen K, Vanroelen C, Burdorf A. Health-improving interventions for obtaining employment in unemployed job seekers. The Cochrane Database Of Systematic Reviews. 2020;1:CD013152.

947. Humber L. Finding employment and inclusion in society for people with learning disabilities. Learning Disability Today. 2013;13(1):22-4.

948. Humber LA, Alcott AAABBBBBBBCCEFFFFGGGGGGGGKMMOC. Social inclusion through employment: The marketisation of employment support for people with learning disabilities in the United Kingdom. Disability & Society. 2014;29(2):275-89.

949. Humensky J, Turner L, Dixon L, Nuechterlein K. Time required by supported education and supported employment services for individuals in early psychosis treatment. Early Intervention in Psychiatry. 2018;12(Supplement 1):58.

950. Humensky JL, Essock SM, Dixon LB. Characteristics associated with the pursuit of work and school among participants in a treatment program for first episode of psychosis. Psychiatric Rehabilitation Journal. 2017;40(1):108-12.

951. Humensky JL, Nossel I, Bello I, Dixon LB. Supported Education and Employment Services for Young People with Early Psychosis in OnTrackNY. The Journal Of Mental Health Policy And Economics. 2019;22(3):95-108.

952. Humensky JL, Turner LR, Dixon LB, Drake RE, Becker DR, Subotnik KL, et al. Personnel time required for supported employment and education services for individuals in a recent-onset psychosis treatment program. Early Intervention In Psychiatry. 2021;15(2):402-5.

953. Humm LB, Olsen D, Be M, Fleming M, Smith M, Huffcutt BSGI. Simulated job interview improves skills for adults with serious mental illnesses. Special Issue: Positive change: Connecting the virtual and the real. 2014;12:50-4.

954. Humm LB, Olsen D, Bell M, Fleming M, Smith M. Simulated job interview improves skills for adults with serious mental illnesses. Journal of Cyber Therapy and Rehabilitation. 2014;7(1):12-4.

955. Hurford I, Calkins M, Villa P, Kohler C. Comparison of 2 first-episode psychosis programs in philadelphia. Schizophrenia Bulletin. 2017;43(Supplement 1):S159-S60.

956. Hutchinson J, Gilbert D, Papworth R, Boardman J. Implementing Supported Employment. Lessons from the Making IPS Work Project. International Journal Of Environmental Research And Public Health. 2018;15(7).

957. Hynes PJ, Harb A. Practices and roles of Irish occupational therapists' with adults with intellectual disabilities who access supported employment services. Irish Journal of Occupational Therapy. 2017;45(2):78-91.

958. Iancu S, Zweekhorst M, Veltman D, Balkom A, Bunders J. Outsourcing Mental Health Care Services? The Practice and Potential of Community-Based Farms in Psychiatric Rehabilitation. Community Mental Health Journal. 2015;51(2):175-84.

959. Iancu SC, Bunders JFG, van Balkom AJLM. Bridging the gap: Using farms to enhance social inclusion of people with chronic mental disorders. Acta Psychiatrica Scandinavica. 2013;128(4):318-9.

960. Ikebuchi E, Sato S, Yamaguchi S, Shimodaira M, Taneda A, Hatsuse N, et al. Does improvement of cognitive functioning by cognitive remediation therapy effect work outcomes in severe mental illness? A secondary analysis of a randomized controlled trial. Psychiatry And Clinical Neurosciences. 2017;71(5):301-8.

961. Ineson R. Exploring paid employment options with a person with severe learning disabilities and high support needs: An exploratory case study. British Journal of Occupational Therapy. 2015;78(1):58-65.

962. Ineson R, Barnes BBBBBBBBEHKKLMMMRRRSSSSSTWWYZ. Exploring paid employment options with a person with severe learning disabilities and high support needs: An exploratory case study. The British Journal of Occupational Therapy. 2015;78(1):58-65.

963. Inge KJ, Cimera RE, Revell WG, Wehman PH, Seward HE. Employment outcomes for individuals with spinal cord injuries: 2011-2013. Journal of Vocational Rehabilitation. 2015;42(1):85-96.

964. Inge KJ, Cimera RE, Revell WG, Wehman PH, Seward HE. "Employment outcomes for individuals with spinal cord injuries: 2011-2013": Erratum. Special Issue: Vocational rehabilitation issues and outcomes for people with chronic illnesses. 2015;42(2):189.

965. Inge KJ, Cimera RE, Rumrill Jr PD, Revell WG. Employment Outcomes for Individuals with Multiple Sclerosis: 2011-2013. Journal of Rehabilitation. 2016;82(4):28-37.

966. Inge KJ, Graham CW, Brooks-Lane N, Wehman P, Griffin C, Inge, et al. Defining customized employment as an evidence-based practice: The results of a focus group study. Journal of Vocational Rehabilitation. 2018;48(2):155-66.

967. Inge KJ, Graham CW, Erickson D, Sima A, West M, Cimera RE. Improving the employment outcomes of individuals with traumatic brain injury: The effectiveness of knowledge translation strategies to impact the use of evidence-based practices by vocational rehabilitation counselors. Journal of Vocational Rehabilitation. 2016;45(1):107-15.

968. Inge KJ, Wehman P, Revell G, Erickson D, Butterworth J, Gilmore D. Survey results from a national survey of community rehabilitation providers holding special wage certificates. Journal of Vocational Rehabilitation. 2009;30(2):67-85.

969. Insel TR. RAISE-ing Our Expectations for First-Episode Psychosis. The American Journal Of Psychiatry. 2016;173(4):311-2.

970. Ipsen C, Goe R, Bliss S. Vocational Rehabilitation (VR) funding of job development and placement services: Implications for rural reach. Journal of Vocational Rehabilitation. 2019;51(3):313-24.

971. Ipsen C, Kurth N, Hall J, McCormick S, Chambless C. Exploring the PROMISE of transition services for youth with disabilities receiving SSI. Journal of Vocational Rehabilitation. 2019;50(1):95-108.

972. Ipsen C, Kurth N, McCormick S, Hall JP, Chambless C, Golden TP, et al. Engaging SSI youth and families with ASPIRE services. Journal of Vocational Rehabilitation. 2019;51(2):211-24.

973. Ipsen C, Swicegood G. Rural and urban vocational rehabilitation self-employment outcomes. Journal of Vocational Rehabilitation. 2017;46(1):97-105.

974. Ireys HT, Wehman P. The Evaluation of the Demonstration to Maintain Independence and Employment. Journal of Vocational Rehabilitation. 2011;34(2):67-9.

975. Isherwood L, Moskos M, King D, Walker R, Brown L. Multiple disadvantage, service delivery and client outcomes in a strengths-based employment program. Australian Bulletin of Labour. 2017;43(1):1-20.

976. Ito J, Oshima I, Nishio M, Kuno E. Initiative to build a community-based mental health system including assertive community treatment for people with severe mental illness in Japan. American Journal of Psychiatric Rehabilitation. 2009;12(3):247-60.

977. Ito J, Oshima I, Nishio M, Kuno E. "Initiative to build a community-based mental health system including assertive community treatment for people with severe mental illness in Japan": Corrigendum. American Journal of Psychiatric Rehabilitation. 2015;18(3):302.

978. Iudici A, Renzi C. The configuration of job placement for people with disabilities in the current economic contingencies in Italy: Social and clinical implications for health. Disability And Health Journal. 2015;8(4):586-93.

979. Iwane T, Yoshida A, Kono M, Hashimoto H, Yamamoto S. Work support for persons with mental disabilities in Japan. Work (Reading, Mass). 2013;45(2):253-60.

980. Jäckel D, Kupper Z, Glauser S, Mueser KT, Hoffmann H. Effects of Sustained Competitive Employment on Psychiatric Hospitalizations and Quality of Life. Psychiatric Services (Washington, DC). 2017;68(6):603-9.

981. Jackson Y, Kelland J, Cosco TD, McNeil DC, Reddon JR. Nonvocational outcomes of vocational rehabilitation: reduction in health services utilization. Work. 2009;33(4):381-7.

982. Jacob A, Scott M, Falkmer M, Falkmer T. The Costs and Benefits of Employing an Adult with Autism Spectrum Disorder: A Systematic Review. Plos One. 2015;10(10):e0139896-e.

983. Jagannathan A, Harish N, Venkatalakshmi C, Kumar CN, Thirthallli J, Kumar D, et al. Supported employment programme for persons with severe mental disorders in India: A feasibility study. The International Journal Of Social Psychiatry. 2020;66(6):607-13.

984. Jäger M, Paras S, Nordt C, Warnke I, Bärtsch B, Rössler W, et al. [How sustainable is supported employment? A follow-up investigation]. Neuropsychiatrie: Klinik, Diagnostik, Therapie Und Rehabilitation: Organ Der Gesellschaft Osterreichischer Nervenarzte Und Psychiater. 2013;27(4):196-201.

985. Jaleel F, Nirmala BP, Thirthalli J. Supported employment initiative for persons with mental illness in Indian setting: an expanded case report. Asian Journal Of Psychiatry. 2014;9:91-2.

986. Janaway BM, Kripalani M. Response letter to the article by Killackey et al (2019) 'Individual placement and support for vocational recovery in first-episode psychosis: randomised controlled trial'. The British journal of psychiatry : the journal of mental science. 2020;216(3):167.

987. Jang Y, Wang Y-T, Lin M-H. Factors affecting employment outcomes for people with disabilities who received Disability Employment Services in Taiwan. Journal Of Occupational Rehabilitation. 2014;24(1):11-21.

988. Jang Y, Wang Y-T, Lin M-H, Beach BBBBC-MCCCCCCDFF-JHJJJLMMMMOPRSS. Factors affecting employment outcomes for people with disabilities who received disability employment services in Taiwan. Journal of Occupational Rehabilitation. 2014;24(1):11-21.

989. Jennings R, Brown K, Wrigley M, Gartland N. Developing an integrated approach across various government departments' for service users with First Episode Psychosis to achieve employment and educational goals using Individual Placement Support model. International Journal of Integrated Care (IJIC). 2017;17:1-2.

990. Jetha A, Bowring J, Furrie A, Smith F, Breslin C. Accommodations best served soft: Supporting the needs of disabled young adults in the workplace. Occupational and Environmental Medicine. 2018;75(Supplement 2):A570.

991. Jetha A, Shaw R, Sinden AR, Mahood Q, Gignac MA, McColl MA, et al. Work-focused interventions that promote the labour market transition of young adults with chronic disabling health conditions: a systematic review. Occupational And Environmental Medicine. 2019;76(3):189-98.

992. Ji E, Schaller J, Pazey B, Glynn K, Alston ABB-BBCDF-PFGG-KGHIKKK-BLLMNNNRSSS. Education and employment outcomes from the RSA data file for transition-age African American, White, and Hispanic youth with learning disabilities. Journal of Applied Rehabilitation Counseling. 2015;46(3):15-24.

993. Johanson S, Markström U, Bejerholm U. Enabling the return-to-work process among people with affective disorders: A multiple-case study. Scandinavian Journal Of Occupational Therapy. 2019;26(3):205-18.

994. Johanson S, Markström U, Larsson ME, Bejerholm U. Implementation of a novel return-to-work approach for persons with affective disorders in a traditional vocational rehabilitation context: a case study. International Journal Of Mental Health Systems. 2020;14:22.

995. Johnson ET, Kaya C, Fong C, Dutta A, Yaghmaian R, Kundu M, et al. Vocational rehabilitation services for African-American women with HIV/AIDS: An examination of acceptance rates and employment outcomes. Journal of Vocational Rehabilitation. 2017;47(2):235-45.

996. Johnson JE, Moore CL, Aref F, Washington AL, Ward C, Webb K. National Survey of State Vocational Rehabilitation Agency and Veterans Affairs Interagency Collaborations: An Emerging Conceptual Framework for Co-Serving Veterans of Color with Disabilities. Journal of Applied Rehabilitation Counseling. 2017;48(4):54-64.

997. Johnson RL, Floyd M, Pilling D, Boyce MJ, Grove B, Secker J, et al. Service users' perceptions of the effective ingredients in supported employment. Journal of Mental Health. 2009;18(2):121-8.

998. Johnson-Kwochka A, Bond GR, Becker DR, Drake RE, Greene MA. Prevalence and Quality of Individual Placement and Support (IPS) Supported Employment in the United States. Administration And Policy In Mental Health. 2017;44(3):311-9.

999. Jones-Parkin T, Thomas F, Hess K, Snyder A. Employment First and transition: Utah school-to-work initiative. Journal of Vocational Rehabilitation. 2021;54(3):265-71.

1000. Jordán de Urríes FB, Verdugo MA. Evaluation and follow up of supported employment initiatives in Spain from 1995 to 2008. Journal of Vocational Rehabilitation. 2010;33(1):39-49.

1001. Jordán de Urríes FB, Verdugo MA. Are we Walking Towards Integrated Employment? A Perspective from Europe and Australia. Journal of Vocational Rehabilitation. 2012;37(3):143-5.

1002. Jordán de Urríes FB, Verdugo MA. Open employment in Spain. Why have we still not taken the decisive step? Journal of Vocational Rehabilitation. 2012;37(3):147-54.

1003. Jorge-Monteiro MF, Ornelas JH. "What's Wrong with the Seed?" A Comparative Examination of an Empowering Community-Centered Approach to Recovery in Community Mental Health. Community Mental Health Journal. 2016;52(7):821-33.

1004. Jorge-Monteiro MF, Ornelas JH, Aubry BBBBBBBCCCCCCCCCCDDDDDDDDDEEEF. "What's wrong with the seed?" A comparative examination of an empowering community-centered approach to recovery in community mental health. Community Mental Health Journal. 2016;52(7):821-33.

1005. Jorgensen Smith T, Dillahunt-Aspillaga C, Kenney C. Integrating customized employment practices within the vocational rehabilitation system. Journal of Vocational Rehabilitation. 2015;42(3):201-8.

1006. Jung Y, Bellini JL. Predictors of Employment Outcomes for Vocational Rehabilitation Consumers With HIV/AIDS: 2002–2007. Rehabilitation Counseling Bulletin. 2011;54(3):142-53.

1007. Jung Y, Bellini JL, Beach BBBCCCCCDE-HFHHIJKMMMMPTTWW. Predictors of employment outcomes for vocational rehabilitation consumers with HIV/AIDS: 2002-2007. Rehabilitation Counseling Bulletin. 2011;54(3):142-53.

1008. Jung Y, Schaller J, Bellini J. Predictors of employment outcomes for state-federal vocational rehabilitation consumers with HIV/AIDS. Rehabilitation Counseling Bulletin. 2010;53(3):175-89.

1009. Jung Y, Schaller J, Bellini J, Allison ABBBBBBBBBCCCCCCCDEFFGGHHHHHHK. Predictors of employment outcomes for state-federal vocational rehabilitation consumers with HIV/AIDS. Rehabilitation Counseling Bulletin. 2010;53(3):175-85.

1010. Jurcak SE, Wright R. Successful transition for adults with ABI from rehabilitation to employment via a non-traditional sheltered workshop. Brain Injury. 2016;30(5-6):741.

1011. Juurlink TT, Lamers F, van Marle HJF, Michon H, van Busschbach JT, Beekman ATF, et al. Employment in Personality Disorders and the Effectiveness of Individual Placement and Support: Outcomes from a Secondary Data Analysis. Journal Of Occupational Rehabilitation. 2019.

1012. Juurlink TT, Lamers F, van Marle HJF, Michon H, van Busschbach JT, Beekman ATF, et al. Employment in Personality Disorders and the Effectiveness of Individual Placement and Support: Outcomes from a Secondary Data Analysis. Journal Of Occupational Rehabilitation. 2020;30(2):255-62.

1013. Kaehne A. Project SEARCH - a new model of supported employment? Learning Disability Today. 2015;15(1):22-4.

1014. Kaehne A. Project SEARCH UK - Evaluating Its Employment Outcomes. Journal Of Applied Research In Intellectual Disabilities: JARID. 2016;29(6):519-30.

1015. Kaehne A, Baer BBBBBCCCDDEEEEEF-JGGGGHHHJKKKKK. Project SEARCH UK-Evaluating its employment outcomes. Journal of Applied Research in Intellectual Disabilities. 2016;29(6):519-30.

1016. Kaehne A, Beyer S. Supported employment for young people with intellectual disabilities facilitated through peer support: a pilot study. Journal Of Intellectual Disabilities: JOID. 2013;17(3):236-51.

1017. Kaehne A, Beyer S, Abbott ABBBBCCCDEFF-JGHHHHHHHJJJKKKKKK. Supported employment for young people with intellectual disabilities facilitated through peer support: A pilot study. Journal of Intellectual Disabilities. 2013;17(3):236-51.

1018. Kalef L, Barrera M, Heymann J. Developing inclusive employment: Lessons from Telenor Open Mind. Work. 2014;48(3):423-34.

1019. Kaletta JP, Binks DJ, Robinson R. Creating an Inclusive Workplace: Integrating Employees With Disabilities Into a Distribution Center Environment. Professional Safety. 2012;57(6):62-71.

1020. Kalkan R, Dorn W, Ehiosun U, Kilian R, Becker T. The effects of obtaining work on the quality of life of persons with severe mental illness. Psychiatrische Praxis. 2011;38(SUPPL. 1).

1021. Kamau C. Is the NHS Mental Health Service Preparing Clients to Resume Employment? Psychiatric Services (Washington, DC). 2016;67(5):578-9.

1022. Kamp M. Supported Employment in The Netherlands: New challenges for integrated employment. Journal of Vocational Rehabilitation. 2012;37(3):155-62.

1023. Kane J, Schooler N. New findings from the NIMH RAISE early treatment program. Early Intervention in Psychiatry. 2016;10(Supplement 1):13.

1024. Kane JM, Robinson DG, Schooler NR, Mueser KT, Penn DL, Rosenheck RA, et al. Comprehensive Versus Usual Community Care for First-Episode Psychosis: 2-Year Outcomes From the NIMH RAISE Early Treatment Program. The American Journal Of Psychiatry. 2016;173(4):362-72.

1025. Kantartzis S, Ammeraal M, Breedveld S, Mattijs L, Geert, Leonardos, et al. "Doing" social inclusion with ELSiTO: empowering learning for social inclusion through occupation. Work (Reading, Mass). 2012;41(4):447-54.

1026. Karakus M, Riley J, Goldman H. Federal Policies and Programs to Expand Employment Services Among Individuals with Serious Mental Illnesses. Administration And Policy In Mental Health. 2017;44(3):339-44.

1027. Karakus M, Salkever D, Goldman H, Frey W. Therapeutic effects of employment among people with serious mental illnesses: Evidence from US mental health treatment study. Journal of Mental Health Policy and Economics. 2013;16(SUPPL. 1):S17.

1028. Karambelas GJ, Cotton SM, Farhall J, Killackey E, Allott KA. Contribution of neurocognition to 18-month employment outcomes in first-episode psychosis. Early Intervention in Psychiatry. 2017:No-Specified.

1029. Karambelas GJ, Cotton SM, Farhall J, Killackey E, Allott KA. Contribution of neurocognition to 18-month employment outcomes in first-episode psychosis. Early Intervention In Psychiatry. 2019;13(3):453-60.

1030. Karen R, Ellie. Partnerships: Creating Vocational Options for People with Mental Illness. Work. 2012;43(1):1-3.

1031. Kärkkäinen R, Saaranen T, Räsänen K. Occupational health care return-to-work practices for workers with job burnout. Scandinavian Journal Of Occupational Therapy. 2019;26(3):194-204.

1032. Kashyap S, Shivalkar R, Nagpal K, Kansal M, Kapoor G, Ghoshal A. Health benefits of supported employment in Patients With Chronic Schizophrenia. Indian Journal of Psychiatry. 2019;61(9 Supplement 3):S570.

1033. Katz EE. Social enterprise businesses: A strategy for creating good jobs for people with disabilities. Journal of Vocational Rehabilitation. 2014;40(2):137-42.

1034. Katz N, Gilad Izhaky S, Ziv O, Revach A. "Coffee Stands": a vocational rehabilitation project in the community for people coping with mental disorders. Work (Reading, Mass). 2013;44(4):481-90.

1035. Kawohl W. Employment and mental health. European Psychiatry. 2015;30(SUPPL. 1):96.

1036. Kawohl W, Bakshecv BBBB-MBBCCDDFHHHKKLNNTTVV. New ways in psychiatric vocational rehabilitation. Neue Formen der psychiatrischen Arbeitsrehabilitation. 2015;12(3):156-61.

1037. Kawohl W, Moock J, Heuchert S, Rössler W. Job Maintenance by Supported Employment: An Overview of the "Supported Employment Plus" Trial. Frontiers In Public Health. 2015;3:140-.

1038. Kaya C. Demographic Variables, Vocational Rehabilitation Services, and Employment Outcomes for Transition‐Age Youth With Intellectual Disabilities. Journal of Policy & Practice in Intellectual Disabilities. 2018;15(3):226-36.

1039. Kaya C, Fong C. Vocational Rehabilitation Services and Outcomes for Working Age People with Depression and Other Mood Disorders. Journal of Rehabilitation. 2017;83(3):44-52.

1040. Kaya C, Fong C, Rumrill P, Hartman E, Wehman P, Kanako I, et al. Vocational rehabilitation services and competitive employment for transition-age youth with autism spectrum disorders. Journal of Vocational Rehabilitation. 2016;45(1):73-83.

1041. Kaya C, Hanley-Maxwell C, Chan F, Tansey T. Differential vocational rehabilitation service patterns and outcomes for transition-age youth with autism. Journal Of Applied Research In Intellectual Disabilities: JARID. 2018;31(5):862-72.

1042. Kaya C, Hsu S, Rumrill PD, Hanley-Maxwell C, Chan F. Differential vocational rehabilitation service patterns and outcomes for transition-age youth with specific learning disabilities: Implications in the COVID-19 era. Journal of Vocational Rehabilitation. 2021;54(1):59-70.

1043. Kaya C, Leslie M, McDaniels B, Cuevas S, Wu H, Rumrill P, et al. Vocational rehabilitation factors associated with successful return to work outcomes for clients with Parkinson's disease. Journal of Vocational Rehabilitation. 2020;52(2):145-56.

1044. Keefe K, Styron T, O'Connell M, Mattias K, Davidson L, Costa M. Understanding family perspectives on supported employment. The International Journal Of Social Psychiatry. 2020;66(1):76-83.

1045. Kehn M, Honeycutt T, Foley S, Sevak P. Implementation and impacts of the Substantial Gainful Activity Project demonstration in Minnesota. Journal of Vocational Rehabilitation. 2020;53(3):307-17.

1046. Kendall KM, Karns GL. The business case for hiring people with disabilities. Social Business. 2018;8(3):277-92.

1047. Kern RS, Smith K, Mitchell S, Gibson C, Gharapetian L, Green MF. Errorless learning and supported employment in schizophrenia: Addressing cognitive impairments as rate-limiting factors to employment success. Schizophrenia Bulletin. 2011;37(SUPPL. 1):269-70.

1048. Kern RS, Smith KM, Mitchell SS, Iglesias J, Dolinsky M, Reddy LF, et al. The effects of errorless learning on competitive and transitional employment in a VA supported employment program. Schizophrenia Bulletin. 2013;39(SUPPL. 1):S294.

1049. Kern RS, Zarate R, Glynn SM, Smith KM, Reddy F, Mitchell SS, et al. The effects of errorless learning on work outcome in schizophrenia. Schizophrenia Bulletin. 2015;41(SUPPL. 1):S178.

1050. Kern RS, Zarate R, Glynn SM, Turner LR, Smith KM, Mitchell SS, et al. A demonstration project involving peers as providers of evidence-based, supported employment services. Psychiatric Rehabilitation Journal. 2013;36(2):99-107.

1051. Kern RS, Zarate R, Glynn SM, Turner LR, Smith KM, Mitchell SS, et al. Improving Work Outcome in Supported Employment for Serious Mental Illness: Results From 2 Independent Studies of Errorless Learning. Schizophrenia Bulletin. 2018;44(1):38-45.

1052. Khalifa G, Sharif Z, Sultan M, Di Rezze B. Workplace accommodations for adults with autism spectrum disorder: a scoping review. Disability And Rehabilitation. 2019:1-16.

1053. Khalifa N, Hadfield S, Thomson L, Talbot E, Bird Y, Schneider J, et al. Barriers and facilitators to the implementation of individual placement and support (IPS) for patients with offending histories in the community: The United Kingdom experience. British Journal of Occupational Therapy. 2020;83(3):179-90.

1054. Khalifa N, Talbot E, Barber S, Schneider J, Bird Y, Attfield J, et al. A Feasibility Cluster Randomized Controlled Trial of Individual Placement and Support (IPS) for Patients With Offending Histories. Frontiers In Psychiatry. 2020;10:952.

1055. Khalifa N, Talbot E, Schneider J, Walker DM, Bates P, Bird Y, et al. Individual placement and support (IPS) for patients with offending histories: the IPSOH feasibility cluster randomised trial protocol. BMJ Open. 2016;6(7):e012710-e.

1056. Khan F, Ng L, Turner-Stokes L. Effectiveness of vocational rehabilitation intervention on the return to work and employment of persons with multiple sclerosis. The Cochrane Database Of Systematic Reviews. 2009(1):CD007256.

1057. Khoronzhevych M, Maximova-Mentzoni T, Gubrium E, Muller AE. Participant Engagement in Supported Employment: A Systematic Scoping Review. Journal Of Occupational Rehabilitation. 2021.

1058. Kiernan WE, Mank DM. Introduction to Employment First: An essential strategy. Special Issue: Employment First: An essential strategy. 2011;49(4):ii.

1059. Kilian H. [Supported Employment - A Wrong Policy for Germany? - Pro]. Psychiatrische Praxis. 2016;43(5):242-3.

1060. Kilian R, Lauber C, Kalkan R, Dorn W, Rössler W, Wiersma D, et al. The relationships between employment, clinical status, and psychiatric hospitalisation in patients with schizophrenia receiving either IPS or a conventional vocational rehabilitation programme. Social Psychiatry And Psychiatric Epidemiology. 2012;47(9):1381-9.

1061. Killackey E. IPS in early psychosis: The Australian experience. European Archives of Psychiatry and Clinical Neuroscience. 2009;259(SUPPL. 1):S10.

1062. Killackey E. Psychosocial and psychological interventions in early psychosis: Essential elements for recovery. Early Intervention in Psychiatry. 2009;3(SUPPL. 1):S17-S21.

1063. Killackey E. Functional recovery in early psychosis: Something old somewhere new. Australian and New Zealand Journal of Psychiatry. 2010;44(SUPPL. 1):A5.

1064. Killackey E. Individual placement and support in early psychosis: Evidence of benefit and future directions. Early Intervention in Psychiatry. 2010;4(SUPPL. 1):6.

1065. Killackey E. Bolstering work: Potential benefits of cognitive interventions to employment interventions for people with early psychosis. Australian and New Zealand Journal of Psychiatry. 2011;45(O1):A21-A2.

1066. Killackey E. Individual placement and support research and development in early psychosis in Australia. Australian and New Zealand Journal of Psychiatry. 2012;46(SUPPL. 1):47.

1067. Killackey E. Not dreaming anymore: IPS employment and education in a national early psychosis system in Australia. Early Intervention in Psychiatry. 2014;8(SUPPL. 1):7.

1068. Killackey E. Resignation not accepted: Employment, education and training in early intervention, past, present and future. Early Intervention in Psychiatry. 2015;9(6):429-32.

1069. Killackey E. Starting fires, not filling buckets: The importance of education in the vocational recovery of young people with first episodes psychosis. Early Intervention in Psychiatry. 2016;10(Supplement 1):56.

1070. Killackey E, Allott K. Utilising Individual Placement and Support to address unemployment and low education rates among individuals with psychotic disorders. The Australian And New Zealand Journal Of Psychiatry. 2013;47(6):521-3.

1071. Killackey E, Allott K, Cotton S, Chinnery G, Jackson H. Baseline to 18 months: Main results from a randomized controlled trial of individual placement and support for young people with first-episode psychosis. Early Intervention in Psychiatry. 2014;8(SUPPL. 1):152.

1072. Killackey E, Allott K, Cotton SM, Chinnery G, Jackson HJ, Baksheev G. The role of neurocognition within a randomised controlled trial of Individual Placement and Support for first-episode psychosis. Schizophrenia Bulletin. 2013;39(SUPPL. 1):S264-S5.

1073. Killackey E, Allott K, Cotton SM, Jackson H, Scutella R, Tseng Y-P, et al. A randomized controlled trial of vocational intervention for young people with first-episode psychosis: method. Early Intervention In Psychiatry. 2013;7(3):329-37.

1074. Killackey E, Allott K, Jackson HJ, Scutella R, Tseng Y-P, Borland J, et al. Individual placement and support for vocational recovery in first-episode psychosis: randomised controlled trial. The British Journal Of Psychiatry: The Journal Of Mental Science. 2019;214(2):76-82.

1075. Killackey E, Allott K, Woodhead G, Connor S, Dragon S, Ring J. Individual placement and support, supported education in young people with mental illness: an exploratory feasibility study. Early Intervention In Psychiatry. 2017;11(6):526-31.

1076. Killackey E, Alvarez-Jimenez BBBBDDDDDFIIJKKLMMMORRRRSSSTW. Resignation not accepted: Employment, education and training in early intervention, past, present and future. Early Intervention in Psychiatry. 2015;9(6):429-32.

1077. Killackey E, Alvarez-Jimenez M, Allott K, Bendall S, McGorry P. Community rehabilitation and psychosocial interventions for psychotic disorders in youth. Child And Adolescent Psychiatric Clinics Of North America. 2013;22(4):745-58.

1078. Killackey E, Becker BBBCKKMNPRRRSWWW. All in a day's work: Opportunities and challenges for vocational interventions in early intervention settings. Early Intervention in Psychiatry. 2010;4(4):267-9.

1079. Killackey E, Cotton S, Allott K. Learning and earning get you more than a job: Impact of employment and education on mental health and other functional variables. Early Intervention in Psychiatry. 2014;8(SUPPL. 1):117.

1080. Killackey E, Cotton S, Allott K. Employment and education outcomes from a rct of individual placement and support for young people with first-episode psychosis. Schizophrenia Bulletin. 2017;43(Supplement 1):S50-S1.

1081. Killackey E, DeLisi LE, Nasrallah HA. Vocational recovery in first episode psychosis: International evidence for early intervention. Schizophrenia Research. 2010;117(2-3):133.

1082. Killackey E, DeLisi LE, Nasrallah HA. Lighting fires not filling buckets: Meaningful vocational recovery in first episode psychosis. Schizophrenia Research. 2010;117(2-3):134.

1083. Killackey E, Jackson H, Allott K, Cotton S. Vocational recovery in first episode psychosis: Preliminary results from a large randomised controlled trial of individual placement and support. Schizophrenia Research. 2014;153(SUPPL. 1):S282.

1084. Killackey EJ, Allott KA, Cotton SM, Chinnery GL, Sun P, Collins Z, et al. Vocational recovery in first-episode psychosis: First results from a large randomized controlled trial of IPS. Early Intervention in Psychiatry. 2012;6(SUPPL.1):13.

1085. Kim H, Bayley M, Dawson D, Mollayeva T, Colantonio A. Characteristics and functional outcomes of brain injury caused by physical assault in Canada: a population-based study from an inpatient rehabilitation setting. Disability and Rehabilitation. 2013;35(26):2213-20.

1086. Kim WH, Nam JK. Corporations May Hire More People With Disabilities Through Public Employment Support Services: Propensity Score Matching Analysis. Rehabilitation Counseling Bulletin. 2019;62(4):243-52.

1087. King DA. Notice of Proposed Information Collection Requests; State Plan for Vocational Rehabilitation Services and Supplement for Supported Employment Services. Federal Register (National Archives & Records Service, Office of the Federal Register). 2012;77(191):60116-.

1088. King DA. Notice of Proposed Information Collection Requests; Office of Special Education and Rehabilitative Services; Annual Vocational Rehabilitation Program/Cost Report (Rehabilitation Services Administration (RSA)-2). Federal Register (National Archives & Records Service, Office of the Federal Register). 2012;77(127):39224-.

1089. King J, Cleary C, Harris MG, Lloyd C, Waghorn G. Employment-related information for clients receiving mental health services and clinicians. Work (Reading, Mass). 2011;39(3):291-303.

1090. King J, Waghorn G. How Higher Performing Employment Specialists Support Job-seekers with Psychiatric Disabilities Retain Employment. Journal of Rehabilitation. 2018;84(4):22-8.

1091. Kinoshita Y, Furukawa TA, Kinoshita K, Honyashiki M, Omori IM, Marshall M, et al. Supported employment for adults with severe mental illness. The Cochrane Database Of Systematic Reviews. 2013(9):CD008297.

1092. Kinoshita Y, Furukawa TA, Omori IM, Watanabe N, Marshall M, Bond GR, et al. Supported employment for adults with severe mental illness. The Cochrane Database Of Systematic Reviews. 2010;2010(1).

1093. Kirsh B. Client, Contextual and Program Elements Influencing Supported Employment: A Literature Review. Community Mental Health Journal. 2016;52(7):809-20.

1094. Kirsh B, Alverson ABBBBBBBBBBBBBB-MBCCCCCCCCCCCCC. Client, contextual and program elements influencing supported employment: A literature review. Community Mental Health Journal. 2016;52(7):809-20.

1095. Kirsh B, Krupa T, Cockburn L, Gewurtz R, Behney BCCCCCDDDEHKKKKMOPRSSW. A Canadian model of work integration for persons with mental illnesses. Disability and Rehabilitation: An International, Multidisciplinary Journal. 2010;32(22):1833-46.

1096. Kirsh B, Stergiou-Kita M, Gewurtz R, Dawson D, Krupa T, Lysaght R, et al. From margins to mainstream: what do we know about work integration for persons with brain injury, mental illness and intellectual disability? Work (Reading, Mass). 2009;32(4):391-405.

1097. Kitayama M. On the support for the employment of psychiatric patients. Seishin shinkeigaku zasshi = Psychiatria et neurologia Japonica. 2009;111(9):1082-6.

1098. Kivekas T, Ahola K, Nevala N, Martimo KP. Competitive work and schizophrenia: Design of a project aiming at obtaining and keeping a job through supported employment and multifaceted collaboration. European Archives of Psychiatry and Clinical Neuroscience. 2013;263(1 SUPPL. 1):S101.

1099. Klayman D, Coughlin J. Summative evaluation of the Employment First State Leadership Mentoring Project 2015 program year. Journal of Vocational Rehabilitation. 2017;47(1):25-45.

1100. Kline E, Keshavan M. Innovations in first episode psychosis interventions: The case for a "RAISE-Plus" approach. Schizophrenia Research. 2017;182:2-3.

1101. Klodnick VV, Sabella K, Brenner CJ, Krzos IM, Ellison ML, Kaiser SM, et al. Perspectives of Young Emerging Adults With Serious Mental Health Conditions on Vocational Peer Mentors. Journal of Emotional & Behavioral Disorders. 2015;23(4):226-37.

1102. Klungsoyr O, Lystad JU, Ueland T, Bull H, Evensen S, Falkum E. G-estimation of causal pathways in vocational rehabilitation for adults with psychotic disorders - a secondary analysis of a randomized trial. BMC Psychiatry. 2021;21(1):370.

1103. Knaeps J, DeSmet A, Van Audenhove C. Supported employment fidelity in Flemish vocational programs. Psychiatrische Praxis. 2011;38(SUPPL. 1).

1104. Knaeps J, DeSmet A, Van Audenhove C. The IPS fidelity scale as a guideline to implement Supported Employment. Journal of Vocational Rehabilitation. 2012;37(1):13-23.

1105. Knaeps J, DeSmet A, Van Audenhove C, Migliore OBRRMRVABMSVLBBBBWGLCTTTWBBBBB. The IPS Fidelity Scale as a guideline to implement supported employment. Journal of Vocational Rehabilitation. 2012;37(1):13-23.

1106. Knaeps J, Neyens I, Donceel P, van Weeghel J, Van Audenhove C. Beliefs of Vocational Rehabilitation Counselors About Competitive Employment for People With Severe Mental Illness in Belgium. Rehabilitation Counseling Bulletin. 2015;58(3):176-88.

1107. Knaeps J, Neyens I, van Weeghel J, Van Audenhove C. Counsellors' focus on competitive employment for people with severe mental illness: an application of the theory of planned behaviour in vocational rehabilitation programmes. British Journal of Guidance & Counselling. 2016;44(1):57-71.

1108. Knaeps J, Neyens I, van Weeghel J, Van Audenhove C, Abraham AAAAABBBBBCCCDFFFFGGGGHHHHHHHK. Counsellors' focus on competitive employment for people with severe mental illness: An application of the theory of planned behaviour in vocational rehabilitation programmes. British Journal of Guidance & Counselling. 2016;44(1):57-71.

1109. Knapp M, McDaid D, Park A, Anthony BE-GE-LE-LFFGHJKKKLLMMMNPPRRSSSTZ. Recovery and economics. Die Psychiatrie: Grundlagen & Perspektiven. 2015;12(3):162-6.

1110. Knijn T, Van Wel F. Better at work: Activation of partially disabled workers in the Netherlands. Alter. 2014;8(4):282-94.

1111. Koatz D, Hilarión P, Bonet P, Suñol R. Integrated care for mental health social inclusion through job placement: Implementing IPS in Spain. International Journal of Integrated Care (IJIC). 2016;16(6):1-2.

1112. Koh PPW. Piloting a transition programme for employment for young adults with stroke or spinal cord injury. Physiotherapy (United Kingdom). 2015;101(SUPPL. 1):eS777-eS8.

1113. Kohler C, Schmidt L, Patton B, Franco O, Huque Z, Calkins M, et al. Further evidence supporting coordinated specialty care for early psychosis: Findings from the samhsa supported penn psychosis evaluation and recovery center (PERC). Schizophrenia Bulletin. 2020;46(Supplement 1):S325-S6.

1114. Koletsi M, Niersman A, van Busschbach JT, Catty J, Becker T, Burns T, et al. Working with mental health problems: clients' experiences of IPS, vocational rehabilitation and employment. Social Psychiatry And Psychiatric Epidemiology. 2009;44(11):961-70.

1115. Kondratova L, Winkler P. The supported employment of people with severe mental illness-The Czech and international experience: A narrative synthesis. Podporovane zamestnavani osob s vaznym dusevnim onemocnenim-Zahranicni a ceska zkusenost: Narativni synteza. 2017;113(3):132-9.

1116. Kostick KM, Whitley R, Bush PW. Client-centeredness in supported employment: specialist and supervisor perspectives. Journal Of Mental Health (Abingdon, England). 2010;19(6):523-31.

1117. Kowlakowsky-Hayner SA, Kolakowsky-Hayner SA, Wright J, Shem K, Medel R, Duong T. An effective community-based mentoring program for return to work and school after brain and spinal cord injury. NeuroRehabilitation. 2012;31(1):63-73.

1118. Kowlakowsky-Hayner SA, Tyerman A. Vocational rehabilitation after traumatic brain injury: Models and services. NeuroRehabilitation. 2012;31(1):51-62.

1119. Kramer J, Bose J, Shepard J, Winsor J. Engaging Families in Employment: Individuals and Families' Retrospective Transition Experiences With Employment Services. Intellectual And Developmental Disabilities. 2020;58(4):314-27.

1120. Kregel J. Work incentives planning and assistance: assisting beneficiaries to obtain employment and reduce dependence on SSA benefits. Journal of Vocational Rehabilitation. 2009;31(1):1-9.

1121. Kregel J, O'Mara S. Work Incentive Counseling as a workplace support. Journal of Vocational Rehabilitation. 2011;35(2):73-83.

1122. Kristman VL, Corbiere M, Shaw WS, Harlos K, Cernigoj M. Factors associated with supervisor support of job accommodations for mental health disorders in the workplace. Occupational and Environmental Medicine. 2018;75(Supplement 2):A560-A1.

1123. Krowatschek G, Kromer S, Stahl F, Rossler W, Kawohl W. Inpatient and Outpatient Treatment in a Crisis Service: Who Uses What? Psychiat Prax. 2011;39(1):21-5.

1124. Krowatschek G, Krömer S, Stahl F, Rössler W, Kawohl W. [Inpatient and outpatient treatment in a crisis service: who uses what?]. Psychiatrische Praxis. 2012;39(1):21-5.

1125. Krupa T, Chen SP. Psychiatric/psychosocial rehabilitation (PSR) in relation to vocational and educational environments: Work and learning. Current Psychiatry Reviews. 2013;9(3):195-206.

1126. Krupa T, Chen S-P, Baron BBBBBBBBCCCCCCCCCCDDDDDDEEGGGG. Psychiatric/psychosocial rehabilitation (PSR) in relation to vocational and educational environments: Work and learning. Current Psychiatry Reviews. 2013;9(3):195-206.

1127. Krysta J, Krysta K, Zawada K, Cubala WJ, Wiglusz MS, Jakuszkowiak-Wojten K, et al. Development of vocational training systems for patients with intellectual disability in Poland. Psychiatria Danubina. 2015;27(Suppl 1):S401-S7.

1128. Kukla M, Bond G. Job Match and Job Tenure in Persons with Severe Mental Illness. Journal of Rehabilitation. 2012;78(1):11-5.

1129. Kukla M, Bond GR. The working alliance and employment outcomes for people with severe mental illness enrolled in vocational programs. Rehabilitation Psychology. 2009;54(2):157-63.

1130. Kukla M, Bond GR. Follow along support and job tenure for persons with severe mental illness in supported employment. Schizophrenia Bulletin. 2011;37(SUPPL. 1):270.

1131. Kukla M, Bond GR. A randomized controlled trial of evidence-based supported employment: Nonvocational outcomes. Journal of Vocational Rehabilitation. 2013;38(2):91-8.

1132. Kukla M, Bond GR, Angell BBBBBBBDDDEFHHKKLMMMMNSTT. The working alliance and employment outcomes for people with severe mental illness enrolled in vocational programs. Rehabilitation Psychology. 2009;54(2):157-63.

1133. Kukla M, Bond GR, Xie H. A prospective investigation of work and nonvocational outcomes in adults with severe mental illness. The Journal Of Nervous And Mental Disease. 2012;200(3):214-22.

1134. Kukla M, McGuire AB, Salyers MP. Rural and urban supported employment programs in the Veterans Health Administration: Comparison of barriers and facilitators to vocational achievement for veterans experiencing mental illnesses. Psychiatric Rehabilitation Journal. 2016;39(2):129-36.

1135. Kukla M, McGuire AB, Salyers MP. Barriers and Facilitators Related to Work Success for Veterans in Supported Employment: A Nationwide Provider Survey. Psychiatric Services (Washington, DC). 2016;67(4):412-7.

1136. Kukla M, McGuire AB, Strasburger AM, Belanger E, Bakken SK. Helping veterans achieve work: A Veterans Health Administration nationwide survey examining effective job development practices in the community. Psychiatric Rehabilitation Journal. 2018;41(2):103-8.

1137. Kukla M, Salyers MP, Strasburger AM, Johnson-Kwochka A, Amador E, Lysaker PH. Work-Focused Cognitive Behavioral Therapy to Complement Vocational Services for People With Mental Illness: Pilot Study Outcomes Across a 6-Month Posttreatment Follow-Up. Psychiatric Rehabilitation Journal. 2019;42(4):366-71.

1138. Kukla M, Strasburger AM, Lysaker PH. A CBT Intervention Targeting Competitive Work Outcomes for Persons With Mental Illness. Psychiatric Services. 2016;67(6):697-.

1139. Kukla M, Strasburger AM, Salyers MP, Rollins AL, Lysaker PH. A Pilot Test of Group Based Cognitive Behavioral Therapy to Augment Vocational Services for Persons With Serious Mental Illness: Feasibility and Competitive Work Outcomes. Journal of Nervous & Mental Disease. 2018;206(5):310-5.

1140. Kukla M, Strasburger AM, Salyers MP, Rollins AL, Lysaker PH. Psychosocial outcomes of a pilot study of work-tailored cognitive behavioral therapy intervention for adults with serious mental illness. Journal Of Clinical Psychology. 2021;77(3):488-95.

1141. Kulkarni M, Scullion H. Talent management activities of disability training and placement agencies in India. International Journal of Human Resource Management. 2015;26(9):1169-81.

1142. Kumar S, Mohanty S. Impact of remunerative jobs on psychopathology of hospitalized chronic schizophrenic patients. Indian Journal of Psychiatry. 2010;52(SUPPL. 1):S46.

1143. Kumar S, Mohanty S. Contribution of paid work assignments in work behaviour of hospitalized chronic schizophrenic patients. Indian Journal of Psychiatry. 2011;53(5 SUPPL. 1):S80.

1144. Kumar S, Mohanty S. Effects of paid work assignments on psychosocial functioning of hospitalized chronic schizophrenic patients. Indian Journal of Psychiatry. 2013;55(SUPPL.1):S120.

1145. Kurtz MM, Bandura BBBBBDDDFGGGGGHKKKLLLLLMMMMMMM. Neurocognition as a predictor of response to evidence-based psychosocial interventions in schizophrenia: What is the state of the evidence? Clinical Psychology Review. 2011;31(4):663-72.

1146. Kurzban S, Davis L, Brekke JS, Kurzban S, Davis L, Brekke JS. Vocational, social, and cognitive rehabilitation for individuals diagnosed with schizophrenia: a review of recent research and trends. Current Psychiatry Reports. 2010;12(4):345-55.

1147. Kuznetsova Y, Yalcin B, Aakvik AABBCCDDDDDEFFFFGGHHHHHHHHHHHH. Inclusion of persons with disabilities in mainstream employment: Is it really all about the money? A case study of four large companies in Norway and Sweden. Disability & Society. 2017;32(2):233-53.

1148. Kymalainen JA, Henze KT, Deluca M, Mitton TA, Walton HM, Duffy P, et al. Are we there yet? The four-year impact of a VA fellowship program on the recovery orientation of rehabilitation programs. Psychiatric rehabilitation journal. 2010;33(4):320-7.

1149. Labriola M, Thielen K, Eplov LF, Nielsen CV. [Vocational rehabilitation]. Ugeskrift For Laeger. 2014;176(10).

1150. Lahey PM, Kirsch B, MacDermid J, Tompa E, Gewurtz RE. Active labour market policies for people with disabilities in receipt of public income benefits: A scoping review. Work. 2019;64(2):229-47.

1151. Lammerts L, Schaafsma FG, Bonefaas-Groenewoud K, van Mechelen W, Anema J. Effectiveness of a return-to-work program for workers without an employment contract, sick-listed due to common mental disorders. Scandinavian Journal Of Work, Environment & Health. 2016;42(6):469-80.

1152. Lammerts L, van Dongen JM, Schaafsma FG, van Mechelen W, Anema JR. A participatory supportive return to work program for workers without an employment contract, sick-listed due to a common mental disorder: an economic evaluation alongside a randomized controlled trial. BMC Public Health. 2017;17(1):162-.

1153. Lamore K, Dubois T, Rothe U, Leonardi M, Girard I, Manuwald U, et al. Return to Work Interventions for Cancer Survivors: A Systematic Review and a Methodological Critique. International Journal Of Environmental Research And Public Health. 2019;16(8).

1154. Lanctôt N, Bergeron-Brossard P, Sanquirgo N, Corbière M. Causal attributions of job loss among people with psychiatric disabilities. Psychiatric Rehabilitation Journal. 2013;36(3):146-52.

1155. Landauer L. JOB COACH HELPS BUILD PRIDE & PROFESSIONALISM. Exceptional Parent. 2017;47(10):28-30.

1156. Landolt K, Brantschen E, Nordt C, Bärtsch B, Kawohl W, Rössler W. Association of Supported Employment With Cognitive Functioning and Employment Outcomes. Psychiatric Services (Washington, DC). 2016;67(11):1257-61.

1157. Langi FLFG, Balcazar FE, Suarez-Balcazar Y. Analysis of Time in Rehabilitation and Incidence of Successful Rehabilitation Within Individuals with Disabilities Receiving Occupational/Vocational Training. Journal Of Occupational Rehabilitation. 2018;28(4):701-10.

1158. Langi FLFG, Oberoi A, Balcazar FE, Awsumb J. Vocational Rehabilitation of Transition-Age Youth with Disabilities: A Propensity-Score Matched Study. Journal Of Occupational Rehabilitation. 2017;27(1):15-23.

1159. Larson JE, Sheehan L, Ryan C, Lemp S, Drandorff L. Practitioner perspectives on Individual Placement and Support (IPS) for individuals with serious mental illness. Journal of Vocational Rehabilitation. 2014;41(3):225-35.

1160. Latimer E. An effective intervention delivered at sub-therapeutic dose becomes an ineffective intervention. The British Journal Of Psychiatry: The Journal Of Mental Science. 2010;196(5):341-2.

1161. Latimer E. Extending the Quality and Reach of IPS. Administration And Policy In Mental Health. 2017;44(3):374-7.

1162. Latimer E, Becker BBBCCDDHJ-KKKLMMNNOPPSSW. Extending the quality and reach of IPS. Administration and Policy in Mental Health and Mental Health Services Research. 2017;44(3):374-7.

1163. Latimer E, Becker BBDFGHLLLLR. An effective intervention delivered at sub-therapeutic dose becomes an ineffective intervention. The British Journal of Psychiatry. 2010;196(5):341-2.

1164. Latimer E, Bordeleau F, Méthot C, Barrie T, Ferkranus A, Lurie S, et al. Implementation of supported employment in the context of a national Canadian program: Facilitators, barriers and strategies. Psychiatric Rehabilitation Journal. 2019.

1165. Latimer E, Bordeleau F, Méthot C, Barrie T, Ferkranus A, Lurie S, et al. Implementation of supported employment in the context of a national Canadian program: Facilitators, barriers and strategies. Psychiatric Rehabilitation Journal. 2020;43(1):2-8.

1166. Latimer EA, Bond GR, Drake RE. Economic approaches to improving access to evidence-based and recovery-oriented services for people with severe mental illness. Can J Psychiatry. 2011;56(9):523-9.

1167. Lauber C. Individual placement and support improves employment of young people with first episode psychosis. Evidence-Based Mental Health. 2009;12(2):53-.

1168. Lauber C. Is supported employment replacing traditional vocational rehabilitation services? European Psychiatry. 2009;24(SUPPL. 1):S241.

1169. Lauber C. Vocational rehabilitation in people with severe mental illness. European Psychiatry. 2011;26(SUPPL. 1).

1170. Laun L, Thoursie PS. Does privatisation of vocational rehabilitation improve labour market opportunities? Evidence from a field experiment in Sweden. Journal of Health Economics. 2014;34:59-72.

1171. Laverman A. PAVING the WAY. PN. 2015;69(10):20-4.

1172. Lawer L, Brusilovskiy E, Salzer MS, Mandell DS. Use of vocational rehabilitative services among adults with autism. Journal Of Autism And Developmental Disorders. 2009;39(3):487-94.

1173. Lawlor ER, Donnelly M. Use of an employment advisory service by cancer survivors. Journal Of Psychosocial Oncology. 2015;33(3):219-31.

1174. Lawlor ER, Donnelly M, Amir BBBBBdBdBGGGGHHHKLMMMMMPRRRSSS. Use of an employment advisory service by cancer survivors. Journal of Psychosocial Oncology. 2015;33(3):219-31.

1175. Le Roux N, Marcellini A. The integration of disabled students into the work place in France. A review and a theoritical discussion. Alter. 2011;5(4):281-96.

1176. Lecomte T, Corbière M, Giguère C-E, Titone D, Lysaker P. Group cognitive behaviour therapy for supported employment - Results of a randomized controlled cohort trial. Schizophrenia Research. 2019.

1177. Lecomte T, Corbière M, Giguère C-E, Titone D, Lysaker P. Group cognitive behaviour therapy for supported employment - Results of a randomized controlled cohort trial. Schizophrenia Research. 2020;215:126-33.

1178. Lecomte T, Corbière M, Leclerc C. [Evidence-based interventions in psychiatric rehabilitation: which ones should we prioritized and why?]. Canadian Journal Of Psychiatry Revue Canadienne De Psychiatrie. 2014;59(4):196-202.

1179. Lecomte T, Corbière M, Leclerc C. Les interventions basées sur les résultats probants en réadaptation psychiatrique : auxquelles accorder la priorité et pourquoi? Canadian Journal of Psychiatry. 2014;59(4):196-202.

1180. Lecomte T, Corbiere M, Leclerc C, Addington ABBBBBCCCCDDDDDFFGGGKKLLLLLLLL. Evidence-based interventions in psychiatric rehabilitation: Which ones should we prioritize and why? Les interventions basees sur les resultats probants en readaptation psychiatrique: Auxquelles accorder la priorite et pourquoi? 2014;59(4):196-202.

1181. Lecomte T, Corbière M, Lysaker PH. [A group cognitive behavioral intervention for people registered in supported employment programs: CBT-SE]. L'encephale. 2014;40 Suppl 2:S81-S90.

1182. Lecomte T, Corbiere M, Lysaker PH, Addington BBBBBCCCCCCCCCDKLLLLLLLLLLLMMM. A group cognitive behavioral intervention for people registered in supported employment programs: CBT-SE. Une intervention cognitive comportementale de groupe pour les personnes suivies dans le cadre d'un programme de soutien en emploi (TCC-SE). 2014;40(Suppl 2):S81-S90.

1183. Lecomte T, Corbiere M, Lysaker PH, Titone D. A group cognitive behavioral intervention for people registered in supported employment programs: CBT-SE. Schizophrenia Bulletin. 2015;41(SUPPL. 1):S180.

1184. Lecomte T, Corbière M, Simard S, Leclerc C. Merging evidence-based psychosocial interventions in schizophrenia. Behavioral Sciences (Basel, Switzerland). 2014;4(4):437-47.

1185. Leddy M, Stefanovics E, Rosenheck R. Health and well-being of homeless veterans participating in transitional and supported employment: Six-month outcomes. Journal Of Rehabilitation Research And Development. 2014;51(1):161-75.

1186. Lee EJ, Park J, Chun J, Pi S. State Vocational Rehabilitation Services and Employment Outcomes for Asian Americans with Psychiatric Disabilities. Community mental health journal. 2020.

1187. Lee E-J, Park J, Chun J, Pi S, Alegria ABBBBBBBCDDHHIKKK-RKLLLLM-WMMOPP. State vocational rehabilitation services and employment outcomes for asian americans with psychiatric disabilities. Community Mental Health Journal. 2020:No-Specified.

1188. Lee SJ, Crowther E, Keating C, Kulkarni J. What is needed to deliver collaborative care to address comorbidity more effectively for adults with a severe mental illness? Australian & New Zealand Journal of Psychiatry. 2013;47(4):333-46.

1189. Lee SM, Chung HY, Hong M. The report on current state and effectiveness of occupational therapy with mentally disabled people in Korea. European Neuropsychopharmacology. 2017;27(Supplement 4):S971-S2.

1190. Legard S, Halrynjo S, Danielsen Floer KE. Is transition support a hurdle to getting a job? Scandinavian Journal of Disability Research. 2016;18(1):17-31.

1191. Leickly E, McDonell M, Monroe-DeVita M, Peterson R, Oluwoye O, Hughes M, et al. Washington state new journeys program for young adults experiencing first episode psychosis: Pilot results. Schizophrenia Bulletin. 2017;43(Supplement 1):S258.

1192. Leinonen T, Viikari-Juntura E, Husgafvel-Pursiainen K, Juvonen-Posti P, Laaksonen M, Solovieva S. The effectiveness of vocational rehabilitation on work participation: a propensity score matched analysis using nationwide register data. Scandinavian Journal Of Work, Environment & Health. 2019;45(6):651-60.

1193. Leonardi M, Scaratti C, Cabello M, Olaya B, Vlachou A, Burger H, et al. Reintegration in the workplace of people with chronic diseases: The EU pathways project and the key role of rehabilitation. Neurorehabilitation and Neural Repair. 2018;32(4-5):348-9.

1194. Leopold A, Elias E, Rumrill P, Stauffer C, Jacobs K, Nardone A, et al. Project career: Development of an inter-professional program to support the transition of students with TBI from postsecondary education to employment. Journal of Head Trauma Rehabilitation. 2015;30(3):E65.

1195. Leopold K, Becker T, Förstl J, Kiefer F, de Millas W, Janetzky W, et al. [Early Intervention in Schizophrenia - an Update]. Fortschritte Der Neurologie-Psychiatrie. 2020;88(6):387-97.

1196. LePage J, Ottomanelli L, Barnett SD, Njoh EN. Spinal cord injury combined with felony history: effect on supported employment for Veterans. Journal Of Rehabilitation Research And Development. 2014;51(10):1497-504.

1197. LePage JP, Crawford AM, Cipher DJ, Anderson K, Rock A, Johnson JAP, et al. Blending Traditional Vocational Services and Individualized Placement and Support for Formerly Incarcerated Veterans. Psychiatric Services (Washington, DC). 2020;71(8):816-23.

1198. LePage JP, Crawford AM, Martin WB, Ottomanelli L, Cipher D, Rock A, et al. The association between time incarcerated and employment success: Comparing traditional vocational services with a hybrid supported employment program for veterans. Psychiatric Rehabilitation Journal. 2021;44(2):142-7.

1199. LePage JP, Lewis AA, Crawford AM, Parish JA, Ottomanelli L, Washington EL, et al. Incorporating Individualized Placement and Support Principles Into Vocational Rehabilitation for Formerly Incarcerated Veterans. Psychiatric Services (Washington, DC). 2016;67(7):735-42.

1200. LePage JP, Lewis AA, Crawford AM, Washington EL, Parish-Johnson JA, Cipher DJ, et al. Vocational rehabilitation for veterans with felony histories and mental illness: 12-month outcomes. Psychological Services. 2018;15(1):56-64.

1201. LePage JP, Martin WB, Crawford AM, Rock A, Parish Johnson JA, Washington EL. Individualized Placement and Support Supported Employment for Justice-involved Homeless and Unemployed Veterans. Medical Care. 2021;59(Suppl 2):S195-S8.

1202. LePage JP, Washington EL, Lewis AA, Johnson KE, Garcia-Rea EA. Effects of structured vocational services on job-search success in ex-offender veterans with mental illness: 3-month follow-up. Journal of Rehabilitation Research & Development. 2011;48(3):277-86.

1203. Leverich AE, Acke S, Verbrugghe M, Schmickler MN, De Brouwer C. Efficiency of vocational rehabilitation programs for workers with schizophrenia: A systematic literature review according to the prisma guidelines. Occupational and Environmental Medicine. 2018;75(Supplement 2):A612-A3.

1204. Lewis G, Thoresen SH, Cocks E. Successful approaches to placing and supporting apprentices and trainees with disability in Australia. Journal of Vocational Rehabilitation. 2011;34(3):181-9.

1205. Lewis G, Thoresen SH, Cocks E. Post-course outcomes of apprenticeships and traineeships for people with disability in Western Australia. Journal of Vocational Rehabilitation. 2011;35(2):107-16.

1206. Lewis R, Dobbs L, Biddle P. ‘If this wasn’t here I probably wouldn’t be’: disabled workers’ views of employment support. Disability & Society. 2013;28(8):1089-103.

1207. Lewis R, Dobbs L, Biddle P, Abberley AABBBBBCDDDSFGGGGGGHHHHPPPPPPR. 'If this wasn't here I probably wouldn't be': Disabled workers' views of employment support. Disability & Society. 2013;28(8):1089-103.

1208. Lexén A, Bejerholm U. Exploring communication and interaction skills at work among participants in individual placement and support. Scandinavian Journal Of Occupational Therapy. 2016;23(4):314-9.

1209. Lexén A, Emmelin M, Bejerholm U. Individual Placement and Support is the keyhole: Employer experiences of supporting persons with mental illness. Journal of Vocational Rehabilitation. 2016;44(2):135-47.

1210. Lexén A, Hofgren C, Bejerholm U. Support and process in individual placement and support: a multiple case study. Work (Reading, Mass). 2013;44(4):435-48.

1211. Lexén A, Hofgren C, Bejerholm U. Reclaiming the worker role: perceptions of people with mental illness participating in IPS. Scandinavian Journal Of Occupational Therapy. 2013;20(1):54-63.

1212. Li Q, Li D, Tsui MCM, Zhang G. Is Mainland China ready to implement vocational rehabilitation to re-engage its people with schizophrenia in meaningful worker roles?...Tse, S., Logistic regression analysis of psychosocial correlates associated with recovery from schizophrenia in a Chinese community. International Journal of Social Psychiatry. Mar 2015. International Journal of Social Psychiatry. 2015;61(2):205-6.

1213. Li Q, Li D, Tsui MCM, Zhang G, Anthony KLLMTTY. Is Mainland China ready to implement vocational rehabilitation to re-engage its people with schizophrenia in meaningful worker roles? International Journal of Social Psychiatry. 2015;61(2):205-6.

1214. Lidén E, Alstersjö K, Gurné FL, Fransson S, Bergbom I. Combining garden therapy and supported employment - a method for preparing women on long-term sick leave for working life. Scandinavian Journal Of Caring Sciences. 2016;30(2):411-8.

1215. Lidz CW, Smith LM. Employment specialists’ perspectives on implementing supported employment with young adults. American Journal of Psychiatric Rehabilitation. 2016;19(4):339-52.

1216. Lidz CW, Smith LM, Arnett BBB-MCCDDDDEHJJM-WMMNNPQRRSST-STW. Employment specialists' perspectives on implementing supported employment with young adults. American Journal of Psychiatric Rehabilitation. 2016;19(4):339-52.

1217. Liffck E, Mehdiyoun NF, Vohs JL, Francis MM, Radnovich AJ, Breier A. Development of coordinated specialty care teams for early psychosis at parc. Schizophrenia Bulletin. 2015;41(SUPPL. 1):S180-S1.

1218. Liljeholm U, Argentzell E, Bejerholm U. An integrated mental health and vocational intervention: A longitudinal study on mental health changes among young adults. Nursing Open. 2020;7(6):1755-65.

1219. Lim Y, Millington M, Mpofu E. The Evidentiary Basis for Supported Employment Practice for Workers with Schizophrenia: A Thematic Analysis. American Journal of Psychiatric Rehabilitation. 2013;16(1):93-113.

1220. Lim Y, Millington M, Mpofu E. The Evidentiary Basis for Supported Employment Practice for Workers with Schizophrenia: A Thematic Analysis. American Journal of Psychiatric Rehabilitation. 2014;17(2):93-113.

1221. Lim Y, Millington M, Mpofu E, Anthony AABBBBBBBBBBBCCCDDDEGGH-MHHHKMM. The evidentiary basis for supported employment practice for workers with schizophrenia: A thematic analysis. American Journal of Psychiatric Rehabilitation. 2014;17(2):93-113.

1222. Lin Y-J, Huang IC, Wang Y-T. Outcomes of home-based employment service programs for people with disabilities and their related factors--a preliminary study in Taiwan. Disability And Rehabilitation. 2014;36(17):1457-63.

1223. Linaker C, Coggon D, Cooper C, Ntani G, Walker-Bone K, Fraser S, et al. Individualised placement and support programme for people unemployed because of chronic pain: A feasibility study and the InSTEP pilot RCT. Health Technology Assessment. 2021;25(5).

1224. Lindsay S, Cagliostro E, Albarico M, Mortaji N, Karon L. A Systematic Review of the Benefits of Hiring People with Disabilities. Journal Of Occupational Rehabilitation. 2018;28(4):634-55.

1225. Lindsay S, Cagliostro E, Carafa G, Allaire AABBBBBBBBBCCCDDDEFFGGGGGGHHHH. A systematic review of workplace disclosure and accommodation requests among youth and young adults with disabilities. Disability and Rehabilitation: An International, Multidisciplinary Journal. 2018;40(25):2971-86.

1226. Lindsay S, McDougall C, Menna-Dack D, Sanford R, Adams T. An ecological approach to understanding barriers to employment for youth with disabilities compared to their typically developing peers: views of youth, employers, and job counselors. Disability And Rehabilitation. 2015;37(8):701-11.

1227. Linkins KW, Brya JJ, Oelschlaeger A, Simonson B, Lahiri S, McFeeters J, et al. Influencing the disability trajectory for workers with serious mental illness: Lessons from Minnesota's Demonstration to Maintain Independence and Employment. Journal of Vocational Rehabilitation. 2011;34(2):107-18.

1228. Linnemørken LTB, Sveinsdottir V, Knutzen T, Rødevand L, Hernæs KH, Reme SE. Protocol for the Individual Placement and Support (IPS) in Pain Trial: A randomized controlled trial investigating the effectiveness of IPS for patients with chronic pain. BMC Musculoskeletal Disorders. 2018;19(1):47-.

1229. Linton KF, Alter BBBBCDGGGHKKMNORRRST. Developing a social enterprise as a social worker. Administration in Social Work. 2013;37(5):458-70.

1230. Liu KPY, Wong D, Chung ACY, Kwok N, Lam MKY, Yuen CMC, et al. Effectiveness of a Workplace Training Programme in Improving Social, Communication and Emotional Skills for Adults with Autism and Intellectual Disability in Hong Kong - A Pilot Study. Occupational Therapy International. 2013;20(4):198-204.

1231. Liu Q, Wilson LH. Adding a vocational focus to Asian mental health services in New Zealand: occupational therapy opportunities. New Zealand Journal of Occupational Therapy. 2009;56(1):40-5.

1232. Llerena K, Reddy LF, Kern RS. The role of experiential and expressive negative symptoms on job obtainment and work outcome in individuals with schizophrenia. Schizophrenia Research. 2018;192:148-53.

1233. Llerena K, Reddy LF, Kern RS, Andreasen AABBBBBBBBCCCCCDDDEEEFFFGGGGHK. The role of experiential and expressive negative symptoms on job obtainment and work outcome in individuals with schizophrenia. Schizophrenia Research. 2018;192:148-53.

1234. Lloyd C, King R. Implementation of supported employment: What are the implications for clinical services? Journal of Rehabilitation. 2012;78(1):25-9.

1235. Lloyd-Evans B, Johnson S. An evaluation of the implementation of an IPS employment support service in a UK catchment area: The WISE study. Psychiatrische Praxis. 2011;38(SUPPL. 1).

1236. Lockett H, Bensemann C. Employment as a health intervention – the role of psychiatry in bridging the evidence to practice gap. Australian & New Zealand Journal of Psychiatry. 2013;47(5):417-20.

1237. Lockett H, Priest R, Grove B. Implementing what works: The impact of the individual placement and support regional trainer. Australian and New Zealand Journal of Psychiatry. 2012;46(SUPPL. 1):47.

1238. Lockett H, Waghorn G, Kydd R. Policy barriers to evidence-based practices in vocational rehabilitation for people with psychiatric disabilities in New Zealand. Work (Reading, Mass). 2018;60(3):421-35.

1239. Lockett H, Waghorn G, Kydd R. The importance of process and outcome measurement in the effective implementation of evidence-based practices in vocational rehabilitation. Australian and New Zealand Journal of Psychiatry. 2018;52(1 Supplement 1):116.

1240. Lockett H, Waghorn G, Kydd R, Chant D. Predictive validity of evidence-based practices in supported employment: a systematic review and meta-analysis. Mental Health Review Journal. 2016;21(4):261-81.

1241. Lombardi L. The 'fondazione San sebastiano': Rehabilitation and care service provision for people with intellectual disabilities and/or mental health problems. Journal of Intellectual Disability Research. 2015;59(SUPPL. 1):57-8.

1242. Lones CE, Bond GR, McGovern MP, Carr K, Leckron-Myers T, Hartnett T, et al. Individual Placement and Support (IPS) for Methadone Maintenance Therapy Patients: A Pilot Randomized Controlled Trial. Administration And Policy In Mental Health. 2017;44(3):359-64.

1243. Lord SE, McGurk SR, Nicholson J, Carpenter-Song EA, Tauscher JS, Becker DR, et al. The potential of technology for enhancing individual placement and support supported employment. Psychiatric Rehabilitation Journal. 2014;37(2):99-106.

1244. Lorenc T, Rodgers M, Marshall D, Melton H, Rees R, Wright K, et al. Support for adults with autism spectrum disorder without intellectual impairment: Systematic review. Autism: The International Journal Of Research And Practice. 2018;22(6):654-68.

1245. Lövgren V, Markström U, Sauer L. Towards Employment: What Research Says About Support-to-Work in Relation to Psychiatric and Intellectual Disabilities. Journal Of Social Work In Disability & Rehabilitation. 2017;16(1):14-37.

1246. Lovstad M, Ugelstad H, Spjelkavik O, Roe C, Schanke AK, Terjesen HCA, et al. The effect evaluation of combined cognitive and vocational interventions after mild-to-moderate traumatic brain injury: A randomized controlled trial and qualitative process evaluation-presentation of a study protocol. Brain Injury. 2017;31(6-7):799-800.

1247. Løvvik C, Øverland S, Hysing M, Broadbent E, Reme SE. Association between illness perceptions and return-to-work expectations in workers with common mental health symptoms. Journal Of Occupational Rehabilitation. 2014;24(1):160-70.

1248. Løvvik C, Shaw W, Overland S, Reme SE. Expectations and illness perceptions as predictors of benefit recipiency among workers with common mental disorders: secondary analysis from a randomised controlled trial. BMJ Open. 2014;4(3):e004321-e.

1249. Lowery LA. Perceived Benefits of Common Educational and Employment Supports for Adults With Autism Spectrum Disorder...2018 AOTA Annual Conference & Expo, April 19-22, 2018, Salt Lake City, Utah. American Journal of Occupational Therapy. 2018;72:1-.

1250. Lu W, Bates FM, Waynor WR, Bazan C, Gao CE, Yanos PT. I feel frozen: Client perceptions of how posttraumatic stress disorder impacts employment. Psychiatric Rehabilitation Journal. 2021.

1251. Luciano A, Bond GR, Drake RE, Becker DR. Is high fidelity to supported employment equally attainable in small and large communities? Community Mental Health Journal. 2014;50(1):46-50.

1252. Luciano A, Drake RE, Bond GR, Becker DR, Carpenter-Song E, Lord S, et al. Evidence-based supported employment for people with severe mental illness: Past, current, and future research. Journal of Vocational Rehabilitation. 2014;40(1):1-13.

1253. Luecking RG. Connecting employers with people who have intellectual disability. Intellectual And Developmental Disabilities. 2011;49(4):261-73.

1254. Luecking RG. International Perspectives on Integrated Employment. Journal of Vocational Rehabilitation. 2013;38(3):163-4.

1255. Lukyanova V. Employment outcomes for people with disabilities across age and disability groups. Archives of Physical Medicine and Rehabilitation. 2013;94(10):e1.

1256. Lukyanova V, Suarez-Balcazar Y, Balcazar F, Oberoi A. A case file review of employment outcomes from Community Rehabilitation Providers. Journal of Vocational Rehabilitation. 2015;43(1):33-40.

1257. Lukyanova VV, Balcazar FE, Oberoi AK, Suarez-Balcazar Y. Employment outcomes among African Americans and Whites with mental illness. Work. 2014;48(3):319-28.

1258. Luo X, Law SF, Wang X, Shi J, Zeng W, Ma X, et al. Effectiveness of an Assertive Community Treatment program for people with severe schizophrenia in mainland China – a 12-month randomized controlled trial. Psychological Medicine. 2019;49(6):969-79.

1259. Lusk SL. Predictors of Successful Vocational Rehabilitation Closure among Individuals with Substance and Alcohol Use Disorders: An Analysis of Rehabilitation Services Administration Data 2010-2014. Alcoholism Treatment Quarterly. 2018;36(2):224-37.

1260. Lusk SL, Drew EHKLLLMMOPPRSSWW. Predictors of successful vocational rehabilitation closure among individuals with substance and alcohol use disorders: An analysis of rehabilitation services administration data 2010-2014. Alcoholism Treatment Quarterly. 2018;36(2):224-37.

1261. Lusk SL, Veale FRB. Increasing Successful Vocational Rehabilitation Outcomes for Individuals with Substance Use Disorders. Journal of Applied Rehabilitation Counseling. 2018;49(1):4-10.

1262. Lynas L. Project ABLE (Autism: Building Links to Employment): A specialist employment service for young people and adults with an autism spectrum condition. Journal of Vocational Rehabilitation. 2014;41(1):13-21.

1263. Lynas L, Cederlund DHHHKLSST. Project ABLE (Autism: Building Links to Employment): A specialist employment service for young people and adults with an autism spectrum condition. Journal of Vocational Rehabilitation. 2014;41(1):13-21.

1264. Lysaght R. Inclusive employment: Will we know it when we see it? Journal of Intellectual Disability Research. 2012;56(7-8):715.

1265. Lysaght R, Cobigo V, Hamilton K, Banks BBBBBBBBCCCCCCCCDDFFFFFGHHHHHJ. Inclusion as a focus of employment-related research in intellectual disability from 2000 to 2010: A scoping review. Disability and Rehabilitation: An International, Multidisciplinary Journal. 2012;34(16):1339-50.

1266. Lysaght R, Jakobsen K, Granhaug B. Social firms: a means for building employment skills and community integration. Work (Reading, Mass). 2012;41(4):455-63.

1267. Lysaght R, Krupa T, Bouchard M, Bond BCCCCEGHKKKKKLLLLLLOCQSSWWY. The role of social enterprise in creating work options for people with intellectual and developmental disabilities. Journal on Developmental Disabilities. 2018;23(3):18-30.

1268. Lysaker PH, Davis LW, Bryson GJ, Bell MD, Lysaker PH, Davis LW, et al. Effects of cognitive behavioral therapy on work outcomes in vocational rehabilitation for participants with schizophrenia spectrum disorders. Schizophrenia Research. 2009;107(2/3):186-91.

1269. Lystad JU, Bull HC, Ueland T, Falkum E. A randomized clinical trial of cognitive remediation versus cognitive behavioral therapy techniques in vocational rehabilitation for adults with schizophrenia spectrum disorders-sample characteristics of the first 40 participants in each intervention. Schizophrenia Bulletin. 2013;39(SUPPL. 1):S297.

1270. Lystad JU, Falkum E, Haaland VØ, Bull H, Evensen S, Bell MD, et al. Neurocognition and occupational functioning in schizophrenia spectrum disorders: The MATRICS Consensus Cognitive Battery (MCCB) and workplace assessments. Schizophrenia Research. 2016;170(1):143-9.

1271. Lystad JU, Falkum E, Haaland VØ, Bull H, Evensen S, McGurk SR, et al. Cognitive remediation and occupational outcome in schizophrenia spectrum disorders: A 2year follow-up study. Schizophrenia Research. 2017;185:122-9.

1272. Lystad JU, Falkum E, Haaland VO, Bull H, Evensen S, Ueland T. Neurocognition and occupational functioning in schizophrenia spectrum disorders. Schizophrenia Bulletin. 2015;41(SUPPL. 1):S54.

1273. Ma Z, Dhir P, Perrier L, Bayley M, Munce S. The Impact of Vocational Interventions on Vocational Outcomes, Quality of Life, and Community Integration in Adults with Childhood Onset Disabilities: A Systematic Review. Journal Of Occupational Rehabilitation. 2019.

1274. Macaden AS, Chandler BJ, Chandler C, Berry A. Sustaining employment after vocational rehabilitation in acquired brain injury. Disability & Rehabilitation. 2010;32(14):1140-7.

1275. Macaden AS, Chandler BJ, Chandler C, Berry A, Abrams CDGGLMOBOPRRRSSTTTVVWY. Sustaining employment after vocational rehabilitation in acquired brain injury. Disability and Rehabilitation: An International, Multidisciplinary Journal. 2010;32(14):1140-7.

1276. MacEachen E, Du B, Bartel E, Ekberg K, Tompa E, Kosny A, et al. Scoping review of work disability policies and programs. International Journal of Disability Management. 2017;12.

1277. Machingura T, Lloyd C. Mental health occupational therapy and supported employment. Irish Journal of Occupational Therapy. 2017;45(1):52-7.

1278. Macias C, Aronson E, Hargreaves W, Weary G, Barreira PJ, Harvey J, et al. Transforming Dissatisfaction with Services into Self-Determination: A Social Psychological Perspective on Community Program Effectiveness. Journal Of Applied Social Psychology. 2009;39(8):1835-59.

1279. Macias C, Gold PB, Hargreaves WA, Aronson E, Bickman L, Barreira PJ, et al. Preference in random assignment: implications for the interpretation of randomized trials. Administration And Policy In Mental Health. 2009;36(5):331-42.

1280. Madsen KP, Cleal B, Olesen K, Hagelund L, Willaing I. Willingness to pay for flexibility at the workplace for people with diabetes and chronic disease: a discrete choice experiment in a population of workers in Denmark. BMC Public Health. 2019;19(1):584-.

1281. Magura S, Marshall T. The Effectiveness of Interventions Intended to Improve Employment Outcomes for Persons with Substance Use Disorder: An Updated Systematic Review. Substance Use & Misuse. 2020;55(13):2230-6.

1282. Magura S, Marshall T, Aklin BBCDDEHJKLLLLMMMMMMMPPRSSSSSWZ. The effectiveness of interventions intended to improve employment outcomes for persons with substance use disorder: An updated systematic review. Substance Use & Misuse. 2020:No-Specified.

1283. Mahmood Z, Keller AV, Burton CZ, Vella L, Matt GE, McGurk SR, et al. Modifiable Predictors of Supported Employment Outcomes Among People With Severe Mental Illness. Psychiatric Services (Washington, DC). 2019;70(9):782-92.

1284. Major B, DeLisi LE, Nasrallah HA. Modifying individual placement & support for an early intervention in psychosis cohort; results of a naturalistic UK study. Schizophrenia Research. 2010;117(2-3):133-4.

1285. Major B, Hinton M, Flint A, Chalmers-Brown A, McLoughlin K, Johnson S. IPS in early psychosis: UK studies. European Archives of Psychiatry and Clinical Neuroscience. 2009;259(SUPPL. 1):S10.

1286. Major BS, Hinton MF, Flint A, Chalmers-Brown A, McLoughlin K, Johnson S. Evidence of the effectiveness of a specialist vocational intervention following first episode psychosis: a naturalistic prospective cohort study. Social Psychiatry And Psychiatric Epidemiology. 2010;45(1):1-8.

1287. Major BS, Hinton MF, Flint A, Chalmers-Brown A, McLoughlin K, Johnson S. Supported employment in inner city London; results of a naturalistic evaluation in a first-episode psychosis cohort. Early Intervention in Psychiatry. 2010;4(SUPPL. 1):6.

1288. Makaay E. Genuine shared collaboration: The role of the vocational worker. Early Intervention in Psychiatry. 2010;4(SUPPL. 1):146.

1289. Malmberg-Heimonen IE, Behncke BBCDDEAFFGHHIJKLLLM-HM-HM-HMMMONS. Social workers' training evaluated by a cluster-randomized study: Reemployment for welfare recipients? Research on Social Work Practice. 2015;25(6):643-53.

1290. Mamboleo G, Bengtson K, Jia-Rung W, Kaya C, Johnson ET, Xiaolei T, et al. Vocational Rehabilitation Services Tailored to Employment Status of Prime Working-Age Adults with HIV/AIDS. Journal of Rehabilitation. 2017;83(3):28-36.

1291. Mamboleo G, Kaya C, Meyer L, Kamnetz B, Bezyak J, Chan F. Vocational rehabilitation services and outcomes for individuals with arthritis in the United States. Journal of Vocational Rehabilitation. 2015;42(2):131-9.

1292. Man DW, Law KM, Chung RC. Cognitive training for Hong Kong Chinese with schizophrenia in vocational rehabilitation. Hong Kong medical journal = Xianggang yi xue za zhi / Hong Kong Academy of Medicine. 2012;18 Suppl 6:18-22.

1293. Man DWK, Law KM, Chung RCK. Cognitive training for Hong Kong Chinese with schizophrenia in vocational rehabilitation. Hong Kong Medical Journal. 2012;18(6 Supplement 6):S18-S22.

1294. Mandal R, Ose SO. Social responsibility at company level and inclusion of disabled persons: the case of Norway. Scandinavian Journal of Disability Research. 2015;17(2):167-87.

1295. Mandal R, Ose SO. Managing absence and dropout in vocational rehabilitation - a mixed-methods analysis of practices and perspectives among vocational rehabilitation companies in Norway. Disability and Rehabilitation. 2019:1-11.

1296. Mandiberg JM, Edwards M. Business Incubation for People with Severe Mental Illness Histories: The Experience of One Model. Journal of Policy Practice. 2016;15(1/2):82-101.

1297. Mann DR, Croake S, Rumrill PD, Koch LC. Learning from State Vocational Rehabilitation Agencies on the eve of WIOA: State differences in service receipt and employment outcomes by applicant employment status. Journal of Vocational Rehabilitation. 2018;48(3):369-86.

1298. Manokara V. Evaluating the impact of different disability support services in Singapore on quality of life (QOL) of persons with intellectual and developmental disabilities (IDD). Journal of Intellectual Disability Research. 2019;63(7):842-3.

1299. Manthey T. Using Motivational Interviewing to Increase Retention in Supported Education. American Journal of Psychiatric Rehabilitation. 2011;14(2):120-36.

1300. Manthey TJ, Rapp CA, Carlson L, Holter MC, Davis JK. The Perceived Importance of Integrated Supported Education and Employment Services. Journal of Rehabilitation. 2012;78(1):16-24.

1301. Margolies PJ, Broadway-Wilson K, Gregory R, Jewell TC, Scannevin G, Jr., Myers RW, et al. Use of learning collaboratives by the center for practice innovations to bring IPS to scale in New York State. Psychiatric Services (Washington, DC). 2015;66(1):4-6.

1302. Margolies PJ, Humensky JL, Chiang IC, Covell NH, Broadway-Wilson K, Gregory R, et al. Is There a Role for Fidelity Self-Assessment in the Individual Placement and Support Model of Supported Employment? Psychiatric Services (Washington, DC). 2017;68(9):975-8.

1303. Margolies PJ, Humensky JL, Chiang IC, Covell NH, Jewell TC, Broadway-Wilson K, et al. Relationship Between Self-Assessed Fidelity and Self-Reported Employment in the Individual Placement and Support Model of Supported Employment. Psychiatric Services (Washington, DC). 2018;69(5):609-12.

1304. Marino L, Nossel I, Choi JC, Nuechterlein K, Wang Y, Essock S, et al. The RAISE Connection Program for Early Psychosis: Secondary Outcomes and Mediators and Moderators of Improvement. Journal of Nervous & Mental Disease. 2015;203(5):365-71.

1305. Marino LA, Dixon LB. An update on supported employment for people with severe mental illness. Current Opinion In Psychiatry. 2014;27(3):210-5.

1306. Marino LA, Dixon LB, Allott ABBBBBBCDDDDFGGGHHHKKKKKKLMNNS. An update on supported employment for people with severe mental illness. Current Opinion in Psychiatry. 2014;27(3):210-5.

1307. Markström U, Svensson B, Bergmark M, Hansson L, Bejerholm U. What influences a sustainable implementation of evidence-based interventions in community mental health services? Development and pilot testing of a tool for mapping core components. Journal Of Mental Health (Abingdon, England). 2018;27(5):395-401.

1308. Markussen S, Røed K. The impacts of vocational rehabilitation. Labour Economics. 2014;31:1-13.

1309. Marrone J, Burns R, Taylor S. Vocational rehabilitation and mental health employment services: True love or marriage of convenience? Journal of Vocational Rehabilitation. 2014;40(2):149-54.

1310. Marsden J, Anders P, Clark H, Colocassis K, Eastwood B, Knight J, et al. Protocol for a multi-centre, definitive randomised controlled trial of the effectiveness of Individual Placement and Support for employment support among people with alcohol and drug dependence. Trials. 2020;21(1):167.

1311. Marshall T, Goldberg RW, Braude L, Dougherty RH, Daniels AS, Ghose SS, et al. Supported employment: assessing the evidence. Psychiatric Services (Washington, DC). 2014;65(1):16-23.

1312. Martin F, Sevak P, Foley S. Implementation and impacts of the Substantial Gainful Activity Project demonstration in Kentucky. Journal of Vocational Rehabilitation. 2020;53(3):297-305.

1313. Martin FH, Walls RT, Brodwin MG, Parker RM, Siu FW, Kurata E. Competitive employment outcomes of vocational rehabilitation. Journal of Applied Rehabilitation Counseling. 2012;43(1):3-10.

1314. Martin M. CESP Impact: Comparing supported employment & sign language interpreting. Journal of Vocational Rehabilitation. 2014;40(2):175-9.

1315. Martin V, Lanovaz MJ. Program evaluation of a community organization offering supported employment services for adults with autism. Research in Autism Spectrum Disorders. 2021;82:101741.

1316. Martinez K. Integrated employment, EmploymentFirst, and U.S. federal policy. Journal of Vocational Rehabilitation. 2013;38(3):165-8.

1317. Martinis JG, Blanck BCDFFHHHKKKLMMMMMMMMPPRSSSTUWW. "The Right to Make Choices": How vocational rehabilitation can help young adults with disabilities increase self-determination and avoid guardianship. Journal of Vocational Rehabilitation. 2015;42(3):221-7.

1318. Maru M, Rogers ES, Hutchinson D, Shappell H. An Integrated Supported Employment and Education Model: Exploratory Study of an Innovative Approach Designed to Better Meet the Needs of Young Adults with Psychiatric Conditions. The Journal Of Behavioral Health Services & Research. 2018;45(3):489-98.

1319. Maru M, Rogers ES, Nicolellis D, Legere L, Placencio-Castro M, Magee C, et al. Vocational peer support for adults with psychiatric disabilities: Results of a randomized trial. Psychiatric Rehabilitation Journal. 2021:No-Specified.

1320. Marwaha S, Gilbert E, Flanagan S. Implementation of an employment intervention in mental health teams: a naturalistic 1-year employment outcome study in people with severe mental illness. Journal of Mental Health. 2014;23(3):135-9.

1321. Matérne M, Lundqvist L-O, Strandberg T. Support Persons’ Perceptions of Giving Vocational Rehabilitation Support to Clients With Acquired Brain Injury in Sweden. Journal of Social Work in Disability & Rehabilitation. 2016;15(3/4):351-69.

1322. Matsuda Y, Sato S, Iwata K, Furukawa S, Hatsuse N, Watanabe Y, et al. Effects of risperidone and aripiprazole on neurocognitive rehabilitation for schizophrenia. Psychiatry And Clinical Neurosciences. 2014;68(6):425-31.

1323. Matsui R, Hata TS. Employment opportunities of persons with disabilities and the special subsidiary company scheme in Japan. Work: Journal of Prevention, Assessment & Rehabilitation. 2013;45(2):261-5.

1324. Mattei G, Venturi G, Alfieri S, Colombini N, Ferrari S, Rigatelli M, et al. Clinical and Socio-demographic Variables Associated with the Outcome of Vocational Rehabilitation Programs: A Community-Based Italian Study. Community Mental Health Journal. 2020;56(7):1380-90.

1325. Matthews LR, Harris LM, Jaworski A, Alam A, Bozdag G. Function, health and psychosocial needs in job-seekers with anxiety, mood, and psychotic disorders who access disability employment services. Work (Reading, Mass). 2014;49(2):271-9.

1326. Mattila-Holappa P, Joensuu M, Ahola K, Koskinen A, Tuisku K, Ervasti J, et al. Psychotherapeutic and work-oriented interventions: employment outcomes among young adults with work disability due to a mental disorder. International Journal Of Mental Health Systems. 2016;10:68-.

1327. Mauro V, Biggeri M, Deepak S, Trani J-F. The effectiveness of community-based rehabilitation programmes: an impact evaluation of a quasi-randomised trial. Journal Of Epidemiology And Community Health. 2014;68(11):1102-8.

1328. Mawn L, Oliver EJ, Akhter N, Bambra CL, Torgerson C, Bridle C, et al. Are we failing young people not in employment, education or training (NEETs)? A systematic review and meta-analysis of re-engagement interventions. Systematic Reviews. 2017;6(1):16-.

1329. Maybee M, Swain JH. The need for employment supports for persons with intellectual and developmental disabilities in North Carolina. North Carolina Medical Journal. 2009;70(6):548-51.

1330. McCluskey A, Arblaster K, Urlic K. Organisational changes leading to use of Assertive Community Treatment and supported employment improve outcomes for people with severe mental illness. Australian Occupational Therapy Journal. 2009;56(5):362-4.

1331. McConkey R, Kelly F, Craig S, Keogh F. A longitudinal study of post-school provision for Irish school-leavers with intellectual disability. British Journal of Learning Disabilities. 2017;45(3):166-71.

1332. McConkey R, Kelly F, Craig S, Keogh F. Changes in the Provision of Day Services in Ireland to Adult Persons With Intellectual Disability. Journal of Policy & Practice in Intellectual Disabilities. 2019;16(1):13-20.

1333. McCormack B, Farrell M, Adams B-BBBCCCDGGHJKKLPPSSSSV. Translating quality of life into service action: Use of personal outcome measures in the Republic of Ireland. British Journal of Learning Disabilities. 2009;37(4):300-7.

1334. McDermott S, Edwards R. Enabling self-determination for older workers with intellectual disabilities in supported employment in Australia. Journal Of Applied Research In Intellectual Disabilities: JARID. 2012;25(5):423-32.

1335. McDonagh MS, Dana T, Selph S, Devine EB, Cantor A, Bougatsos C, et al. Treatments for Schizophrenia in Adults: A Systematic Review. 2017.

1336. McDonald S, Bertram M. Job creation through income generation: an evaluation of Re-Cover, a decorating project developed with forensic mental health service users. Journal of Mental Health Training, Education & Practice. 2018;13(3):148-56.

1337. McDonnall MC, Cmar J. Employment Outcomes and Job Quality of Vocational Rehabilitation Consumers With Deaf-Blindness. Rehabilitation Counseling Bulletin. 2019;63(1):13-24.

1338. McDonnall MC, Cmar JL. Services for Consumers Who Are Deafblind: Vocational Rehabilitation Agency Service Models Utilized and Their Effectiveness. Journal of Visual Impairment & Blindness. 2019;113(1):19-31.

1339. McDonnall MC, Crudden A, Avila BBBC-MDFHHJLLNRRRSSSTWWWW. Factors affecting the successful employment of transition-age youths with visual impairments. Journal of Visual Impairment & Blindness. 2009;103(6):329-41.

1340. McDonough JT, Revell G. Accessing employment supports in the adult system for transitioning youth with autism spectrum disorders. Journal of Vocational Rehabilitation. 2010;32(2):89-100.

1341. McDonough JT, Revell G, Hiller BDHOBWKPLMWB-LB-LB-LHTWWCCGB. Accessing employment supports in the adult system for transitioning youth with autism spectrum disorders. Special Issue: Autism Spectrum Disorders: Transition and Employment. 2010;32(2):89-100.

1342. McDowell C, Ennals P, Fossey E. Vocational Service Models and Approaches to Improve Job Tenure of People With Severe and Enduring Mental Illness: A Narrative Review. Frontiers In Psychiatry. 2021;12:668716.

1343. McDowell C, Fossey E. Workplace accommodations for people with mental illness: a scoping review. Journal Of Occupational Rehabilitation. 2015;25(1):197-206.

1344. McDowell C, Fossey E, Banks BCCDRDFGGGGHKLM-WM-WMMMMSSSSTUWW. Workplace accommodations for people with mental illness: A scoping review. Journal of Occupational Rehabilitation. 2015;25(1):197-206.

1345. McFarlane WR, Lynch S, Melton R. Family psychoeducation in clinical high risk and first-episode psychosis. Adolescent Psychiatry. 2012;2(2):182-94.

1346. McFarlane WR, Lynch S, Melton R, Addington AAAABBBBBBBBBBBBCCCDDDDEFFFFFF. Family psychoeducation in clinical high risk and first-episode psychosis. Special Issue: Youth at risk for psychosis. 2012;2(2):182-94.

1347. McGahey E, Waghorn G, Lloyd C, Morrissey S, Williams PL. Formal plan for self-disclosure enhances supported employment outcomes among young people with severe mental illness. Early Intervention In Psychiatry. 2016;10(2):178-85.

1348. McGeough C. Compensated work therapy program provides an evidence-based model of supported employment. Contemporary Rehab. 2009;65(5):19-20.

1349. McGinty EE, Kennedy-Hendricks A, Linden S, Choksy S, Stone E, Daumit GL. An innovative model to coordinate healthcare and social services for people with serious mental illness: A mixed-methods case study of Maryland's Medicaid health home program. General Hospital Psychiatry. 2018;51:54-62.

1350. McGrew JH, Danner M. Evaluation of an intensive case management program for transition age youth and its transition to assertive community treatment. American Journal of Psychiatric Rehabilitation. 2009;12(3):278-94.

1351. McGuire AB, Bond GR, Clendenning DR, Kukla M. Service intensity as a predictor of competitive employment in an individual placement and support model. Psychiatric Services (Washington, DC). 2011;62(9):1066-72.

1352. McGuire AB, Bond GR, Clendenning DR, Kukla M, Becker BBBBBBBBBBBCCFFHHJKLM-WMMMMRT. Service intensity as a predictor of competitive employee in an individual placement and support model. Psychiatric Services. 2011;62(9):1066-72.

1353. McGurk SR. The role of coaching strategies in maximizing benefit from cognitive remediation when combined with supported employment. Schizophrenia Bulletin. 2015;41(SUPPL. 1):S183.

1354. McGurk SR, Drake RE, Xie H, Riley J, Milfort R, Hale TW, et al. Cognitive Predictors of Work Among Social Security Disability Insurance Beneficiaries With Psychiatric Disorders Enrolled in IPS Supported Employment. Schizophrenia Bulletin. 2018;44(1):32-7.

1355. McGurk SR, Mueser KT. Cognitive remediation and supported employment: The thinking skills for work program. Schizophrenia Bulletin. 2013;39(SUPPL. 1):S343-S4.

1356. McGurk SR, Mueser KT. Sustaining the Long-Term Effects of Supported Employment for Persons With Psychiatric Disabilities. The American Journal Of Psychiatry. 2016;173(10):953-5.

1357. McGurk SR, Mueser KT, DeRosa TJ, Wolfe R. Work, recovery, and comorbidity in schizophrenia: a randomized controlled trial of cognitive remediation. Schizophrenia Bulletin. 2009;35(2):319-35.

1358. McGurk SR, Mueser KT, DeRosa TJ, Wolfe R, Addington AAAABBBBBBBBBBBBBBBBBBBBBBBCCC. Work, recovery, and comorbidity in schizophrenia: A randomized controlled trial of cognitive remediation. Schizophrenia Bulletin. 2009;35(2):319-35.

1359. McGurk SR, Mueser KT, Xie H, Feldman K, Shaya Y, Klein L, et al. Cognitive remediation for vocational rehabilitation nonresponders. Schizophrenia Research. 2016;175(1-3):48-56.

1360. McGurk SR, Mueser KT, Xie H, Welsh J, Kaiser S, Drake RE, et al. Cognitive Enhancement Treatment for People With Mental Illness Who Do Not Respond to Supported Employment: A Randomized Controlled Trial. The American Journal Of Psychiatry. 2015;172(9):852-61.

1361. McGurk SR, Schiano D, Mueser KT, Wolfe R. Implementation of the thinking skills for work program in a psychosocial clubhouse. Psychiatric Rehabilitation Journal. 2010;33(3):190-9.

1362. McLaren J, Lichtenstein JD, Lynch D, Becker D, Drake R. Individual Placement and Support for People with Autism Spectrum Disorders: A Pilot Program. Administration And Policy In Mental Health. 2017;44(3):365-73.

1363. McManus E, Sach TH, Radford K. The importance of perspective when evaluating the economic value of vocational rehabilitation. Value in Health. 2017;20(9):A738.
[truncated: 192,250 more chars]
